# Enhanced succinic acid production by *Mannheimia* employing optimal malate dehydrogenase

**DOI:** 10.1038/s41467-020-15839-z

**Published:** 2020-04-23

**Authors:** Jung Ho Ahn, Hogyun Seo, Woojin Park, Jihye Seok, Jong An Lee, Won Jun Kim, Gi Bae Kim, Kyung-Jin Kim, Sang Yup Lee

**Affiliations:** 1 Metabolic and Biomolecular Engineering National Research Laboratory, Department of Chemical and Biomolecular Engineering (BK21 Plus Program), Institute for the BioCentury, Korea Advanced Institute of Science and Technology (KAIST), Daejeon, 34141 Republic of Korea; 2 Systems Metabolic Engineering and Systems Healthcare Cross-Generation Collaborative Laboratory, KAIST, Daejeon, 34141 Republic of Korea; 3 Bioinformatics Research Center and BioProcess Engineering Research Center KAIST, Daejeon, 34141 Republic of Korea; 4 School of Life Sciences, KNU Creative BioResearch Group, Kyungpook National University, Daegu, 41566 Republic of Korea; 5 Pohang Accelerator Laboratory, Pohang University of Science and Technology, Pohang, Republic of Korea; 6 KNU Institute for Microorganisms, Kyungpook National University, Daegu, 41566 Republic of Korea

**Keywords:** Protein design, Metabolic engineering, Applied microbiology

## Abstract

Succinic acid (SA), a dicarboxylic acid of industrial importance, can be efficiently produced by metabolically engineered *Mannheimia succiniciproducens*. Malate dehydrogenase (MDH) is one of the key enzymes for SA production, but has not been well characterized. Here we report biochemical and structural analyses of various MDHs and development of hyper-SA producing *M. succiniciproducens* by introducing the best MDH. *Corynebacterium glutamicum* MDH (*Cg*MDH) shows the highest specific activity and least substrate inhibition, whereas *M. succiniciproducens* MDH (*Ms*MDH) shows low specific activity at physiological pH and strong uncompetitive inhibition toward oxaloacetate (*ki* of 67.4 and 588.9 μM for *Ms*MDH and *Cg*MDH, respectively). Structural comparison of the two MDHs reveals a key residue influencing the specific activity and susceptibility to substrate inhibition. A high-inoculum fed-batch fermentation of the final strain expressing *cgmdh* produces 134.25 g L^−1^ of SA with the maximum productivity of 21.3 g L^−1^ h^−1^, demonstrating the importance of enzyme optimization in strain development.

## Abstract (web summary)

Malate dehydrogenase (MDH) is one of the key enzymes for succinic acid (SA) bioproduction. Here, the authors report biochemical and structural analyses of various MDHs to reveal amino acids influencing the specific activity and susceptibility to substrate inhibition, and achieve industrial-level SA production.

## Introduction

Bio-based production of industrial chemicals from renewable non-food biomass has become increasingly important as a sustainable substitute for conventional petroleum-based production processes relying on fossil resources. Among many chemicals that can be produced biologically, succinic acid (SA), a four-carbon dicarboxylic acid, is one of the most promising platform chemicals serving as a precursor for industrially important chemicals, such as 1,4-butanediol, γ-butyrolactone, tetrahydrofuran, and as a monomer to manufacture various polymers^1^. Recognizing its importance, much effort has been exerted to metabolically engineer various microorganisms toward development of efficient SA production bioprocesses^1^. Moreover, companies such as Ajinomoto^2^, Myriant^3^, Reverdia^4^, and Succinity^5^ have successfully established demo plant-scale bio-based SA production, seeking its commercialization^1^.

Various microorganisms, including *Actinobacillus succinogenes*^6^, *Corynebacterium glutamicum*^7^, *Escherichia coli*^8^, *Mannheimia succiniciproducens*^9^ (and *Basfia succiniciproducens*^5^, which is almost identical to *M. succiniciproducens* in genome sequence), *Saccharomyces cerevisiae*^10^, and *Yarrowia lipolytica*^11^ have been studied and engineered to develop SA production bioprocesses. Although significant progress has been made on SA production using *C. glutamicum*, *E. coli*, and yeast strains, these non-natural SA producers produce SA with relatively low overall production indices (titer, yield, and productivity) and often rely on dual phase fermentation process comprising aerobic cultivation of cells to a high density followed by switching to anaerobic condition to produce SA^7,12^. On the other hand, natural SA producers such as *A. succinogenes* and *M. succiniciproducens* (*B. succiniciproducens*) that are capnophilic, Gram-negative, and facultative anaerobic bacteria can efficiently produce SA as a major fermentation product^1^. Thus, these natural SA producers have been suggested as good candidate strains for industrial-scale SA production^5,6,9^.

Among these SA producing microorganisms, *M. succiniciproducens* has been proven to be one of the best strains for SA production. In our previous studies, energy balance^13^, byproduct pathways elimination^14^, and flux re-routing^9^ based on in silico genome-scale metabolic analyses were performed to enhance SA production in *M. succiniciproducens*. The *M. succiniciproducens* PALK strain, which was developed by deleting lactate dehydrogenase (LDHA), phosphate acetyltransferase (PTA), and acetate kinase (ACKA) to prevent byproduct formation, produced nearly homo-SA with high productivity^15^. Moreover, various studies including the use of inexpensive carbon sources^16^, membrane engineering for strain robustness^17^, and development of effective fermentation^9^ and downstream processes^15^ were carried out. However, there had been no effort exerted to improve the enzymes to further improve SA production.

**Fig. 1:**
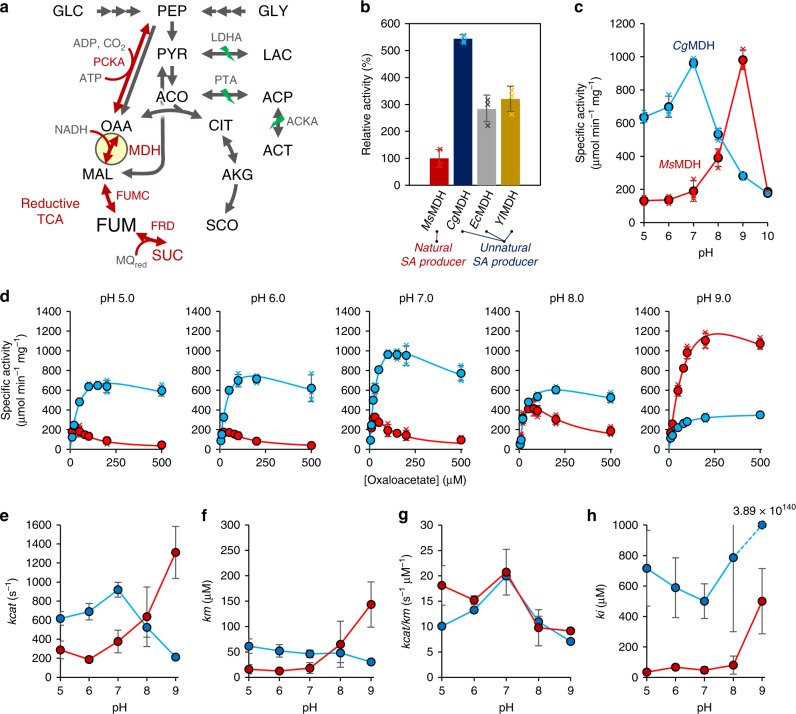
Comparison of MDH activities. **a** SA biosynthetic pathway in the *M. succiniciproducens* PALK strain. Deleted genes are indicated as green thunder symbol. Key enzymes in SA production are indicated as red arrows. GLC glucose, PEP phosphoenolpyruvate, GOL glycerol, PYR pyruvate, LAC lactate, ACO acetyl-CoA, ACP acetyl-phosphate, ACT acetate, OAA oxaloacetate, MAL malate, FUM fumarate, SUC succinate, SCO succinyl-CoA, AKG alpha-ketoglutarate, CIT citrate, PCKA phosphoenolpyruvate carboxylase, LDHA lactate dehydrogenase, PTA phosphate acetyltransferase, ACKA acetate kinase, MDH malate dehydrogenase, FUMC fumarate hydratase, FRD fumarate reductase, MQ_red_ menaquinol. **b** The relative activities of four MDHs from various SA producers, including *M. succiniciproducens*, *C. glutamicum*, *E. coli*, and *Y. lipolytica*, in comparison with the activity of *Ms*MDH (*n* = 3 independent experiments). Data are presented as mean values ± standard deviation. **c** Optimal pH of *Ms*MDH and *Cg*MDH (*n* = 3 independent experiments). Data are presented as mean values ± standard deviation. The specific activities at pH 10.0 were determined from a single data. **d** Catalytic performances of *Ms*MDH and *Cg*MDH at different pH (*n* = 3 independent experiments). Data are presented as mean values ± standard deviation. **e** *kcat*, **f** *km*, **g** *kcat/km*, and **h** *ki* values of *Ms*MDH and *Cg*MDH at different pH. Red and blue lines represent *Ms*MDH and *Cg*MDH, respectively. The *ki* value of *Cg*MDH at pH 9.0 is shown in number because its value is significantly higher than the rest of the *ki* values. Data **e**–**h** are presented as parameters ± standard error. The standard errors from determining the kinetic parameters using OriginPro 2019 software (*n* = the number of mean velocity data at specific pH) are shown as bars.

In *M. succiniciproducens*, SA is produced through the reductive branch of tricarboxylic acid (TCA) cycle. The key enzymes involved in SA production were identified to be phosphoenolpyruvate (PEP) carboxykinase (PCKA) that converts PEP to oxaloacetate (OAA) while producing ATP, malate dehydrogenase (MDH) that reduces OAA to malate using NADH as a cofactor, fumarase (FUMC) that converts malate to fumarate, and fumarate reductase (FRD) that reduces fumarate to SA using menaquinol as a cofactor (Supplementary Fig. 1 and Supplementary Table 1). Among these key enzymes, the NAD^+^/NADH-dependent MDH was predicted to be important for further engineering as it converts OAA to malate, which is a committed step toward SA biosynthesis (Fig. 1a)^18^. Considering the importance of MDH, there had been studies on the amplification of a heterologous MDH in *E. coli*^19,20^ and controlled MDH localization in *S. cerevisiae*^21,22^. However, there had been no studies on systematically characterizing various MDHs. Also, there had been no studies on the role of MDH in SA production by rumen bacteria, the most efficient SA producers.

Phylogenetic analysis of MDH from previous study classified this enzyme into two categories: cytosolic and mitochondrial MDHs, as they were found to be localized at different cellular compartments and showed distinctive characteristics^23^. In general, the cytosolic MDHs preferentially participate in malate–aspartate shuttle for NADH transfer, whereas the mitochondrial MDHs participate in oxidative TCA cycle^24,25^. Thus, the cytosolic MDH seems to be more appropriate for OAA reduction compared with the mitochondrial MDH^26^. However, the MDHs from the major microorganisms employed for SA overproduction, such as *A. succinogenes, E. coli, M. succiniciproducens, S. cerevisiae*, and *Y. lipolytica* are clustered together with the mitochondrial MDH family, whereas the *C. glutamicum* MDH belongs to the cytosolic MDH family (Supplementary Fig. 2 and Supplementary Data 1). These intriguing results led us to comparatively examine the structural and catalytic characteristics of MDHs.

In this study, we report the results of detailed studies on various MDHs, selection of the best MDH and subsequent metabolic engineering of *M. succiniciproducens*, and development of fermentation processes for highly efficient SA production. This strategy of integrating systems metabolic engineering with enzyme engineering will be useful for developing high performance strains for the production of industrially competitive bio-based chemicals.

## Results

### Kinetic characteristics of various MDHs

In order to find an optimal MDH for OAA to malate conversion, eight different MDHs from the most studied and/or industrially employed natural and non-natural SA producers including *A. succinogenes* (*As*MDH), *C. glutamicum* (*Cg*MDH), *E. coli* (*Ec*MDH), *M. succiniciproducens* (*Ms*MDH), *S. cerevisiae* (mitochondrial *Sc*MDH1, cytosolic *Sc*MDH2, and glyoxysomal *Sc*MDH3), and *Y. lipolytica* (cytosolic *Yl*MDH)^27^ were evaluated. This was because there would be a better chance of finding an optimal MDH from these microorganisms, which potentially bears desirable properties for enhanced SA production in *M. succiniciproducens*, rather than from other organisms not capable of efficiently producing SA. The physiological roles of these selected MDHs are summarized in Supplementary Note 1. Out of the eight MDHs expressed in *E. coli*, only four MDHs, including *Ms*MDH, *Cg*MDH, *Ec*MDH, and *Yl*MDH, were expressed in soluble form and successfully purified. The OAA reduction activities of four MDHs at pH 7.0 were measured using the purified enzymes. Considering that *M. succiniciproducens* is the most efficient SA producer, it was our surprise to find that *Ms*MDH exhibited the lowest activity among the four MDHs compared (Fig. 1b). *Cg*MDH*, Ec*MDH, and *Yl*MDH showed 4.4, 1.8, and 2.2-fold higher activities than *Ms*MDH, respectively (Fig. 1b). These results were intriguing as *M. succiniciproducens* has been thought to possess a very efficient reductive branch of TCA cycle as it produces SA with high productivity^18^.

Detailed kinetic analyses were performed on *Ms*MDH and *Cg*MDH (the MDH possessing the highest activity) to compare their characteristics. The kinetic analyses were performed using OAA and NADH as substrates, to focus on the thermodynamically favored reaction, which is the reaction necessary for SA production. First, enzyme activities of *Ms*MDH and *Cg*MDH were measured at various pH of 5.0–10.0 because the activities of MDHs have shown to be strongly influenced by pH^28^ (Fig. 1c). *Cg*MDH showed high activity at relatively acidic pH and the highest activity at pH 7.0, whereas *Ms*MDH showed the highest activity at pH 9.0 and dramatically reduced activity at acidic pH. As the optimal pH for growth of *M. succinicproducens* is pH 6.5–7.2^29,30^, fermentation has typically been performed at pH 6.5, which is the lowest pH for SA production without sacrificing cell growth. Furthermore, the intracellular pH of *M. succiniciproducens* PALK strain was found to be 6.86 ± 0.21 (Supplementary Fig. 3 and Supplementary Table 2). In another study, *Cg*MDH was identified to be a major enzyme for OAA reduction in *C. glutamicum* under both aerobic and anaerobic conditions, whereas the membrane-associated malate:quinone oxidoreductase (MQO) in *C. glutamicum* has a major role in malate oxidation to OAA instead of *Cg*MDH^31^. Thus, the above results on MDH activities suggest that *Cg*MDH will likely be more effective than *Ms*MDH for SA production in *M. succinicproducens*.

Quasi-steady-state kinetic analyses of *Ms*MDH and *Cg*MDH were also performed at various pH to determine their kinetic parameters (Fig. 1d–h and Table 1). At pH 5.0–7.0, *Cg*MDH exhibited significantly higher *kcat* compared with *Ms*MDH, which is consistent with the results of the optimal pH study above (Fig. 1c and Table 1). However, the *km* values of *Ms*MDH were lower than those of *Cg*MDH at pH 5.0–7.0, resulting in similar catalytic efficiencies (*kcat*/*km)* between *Ms*MDH and *Cg*MDH (Fig. 1e–g and Table 1). It was notable that both *Ms*MDH and *Cg*MDH were inhibited by OAA (Fig. 1d); these characteristics are similar to those reported for other MDHs^32–35^. In particular, *Ms*MDH activity was significantly reduced by substrate inhibition under acidic and neutral pH, showing much lower *ki* values than *Cg*MDH throughout the entire pH range (Fig. 1d, h, and Table 1). The intracellular OAA concentration in *M. succiniciproducens* PALK strain was 26.11 ± 4.47 μM, confirming that substrate inhibition of *Ms*MDH occurred in the cell (Fig. 1h, Supplementary Figs. 4, 5, Table 1, and Supplementary Tables 3–5). On the other hand, *Cg*MDH activity was reduced only to a small degree due to relatively mild substrate inhibition as reflected by its high *ki* values over the entire pH range (Fig. 1d, h, and Table 1). It is notable that substrate inhibition of *Ms*MDH tends to be alleviated with increasing pH, showing a pattern similar to its pH-dependent activity (Fig. 1d–h and Table 1). Taken together, *Ms*MDH showing low *kcat* and *ki* values at pH 5.0–7.0 is not likely the best MDH for the production of SA by *M. succinicproducens* PALK strain having a physiological pH of 6.5–7.0 and an intracellular OAA concentration of 26.11 ± 4.47 μM. Instead, *Cg*MDH showing high *kcat* and *ki* values at pH 5.0–7.0 is a better MDH for enhanced SA production in *M. succiniciproducens*.

**Table 1:** Kinetic parameters of *Ms*MDH, *Ms*MDH^G11Q^, and *Cg*MDH toward OAA.

	*Ms*MDH				*Cg*MDH				*Ms*MDH^G11Q^			
pH	*kcat* (s^−1^)	*km* (μM)	*kcat*/*km* (s^−1^ μM^−1^)	*ki* (μM)	*kcat* (s^−1^)	*km* (μM)	*kcat/km* (s^−1^ μM^−1^)	*ki* (μM)	*kcat* (s^−1^)	*km* (μM)	*kcat/km* (s^−1^ μM^−1^)	*ki* (μM)
5	287.4 ± 93.5	15.9 ± 9.0	18.1 ± 3.9	34.7 ± 15.9	617.8 ± 72.8	61.4 ± 14.3	10.1 ± 0.3	716.1 ± 248.1	664.9 ± 62.8	62.0 ± 9.1	10.7 ± 0.2	125.0 ± 19.1
6	187.0 ± 29.6	12.3 ± 3.6	15.2 ± 0.9	67.4 ± 18.6	689.1 ± 86.0	52.0 ± 12.3	13.3 ± 0.5	588.9 ± 196.2	762.4 ± 204.6	99.0 ± 39.0	7.7 ± 0.9	188.1 ± 80.1
7	376.1 ± 119.1	18.2 ± 10.5	20.7 ± 4.5	47.3 ± 22.1	919.8 ± 77.0	46.1 ± 7.3	19.9 ± 0.3	500.0 ± 114.1	927.4 ± 152.4	103.5 ± 25.9	9.0 ± 0.4	302.4 ± 92.7
8	635.8 ± 312.6	65.1 ± 45.3	9.8 ± 3.5	80.3 ± 59.1	524.8 ± 103.4	47.8 ± 18.7	11.0 ± 1.0	786.4 ± 486.1	1005.4 ± 171.6	94.1 ± 29.7	10.7 ± 0.7	914.3 ± 368.4
9	1310.9 ± 273.0	143.3 ± 44.5	9.1 ± 0.6	500.1 ± 214.2	213.2 ± 6.6	30.2 ± 3.5	7.1 ± 0.1	N.A.^a^	1037.9 ± 186.6	207.1 ± 53.2	5.0 ± 0.2	N.A.^a^

Data are presented as parameters ± standard error. The standard errors from determining the kinetic parameters using OriginPro 2019 software (*n* = the number of mean velocity data at specific pH) are indicated after ±.

^a^N.A: Data not provided due to extremely high value.

### Structural and kinetic analyses of *Ms*MDH and *Cg*MDH

**Fig. 2:**
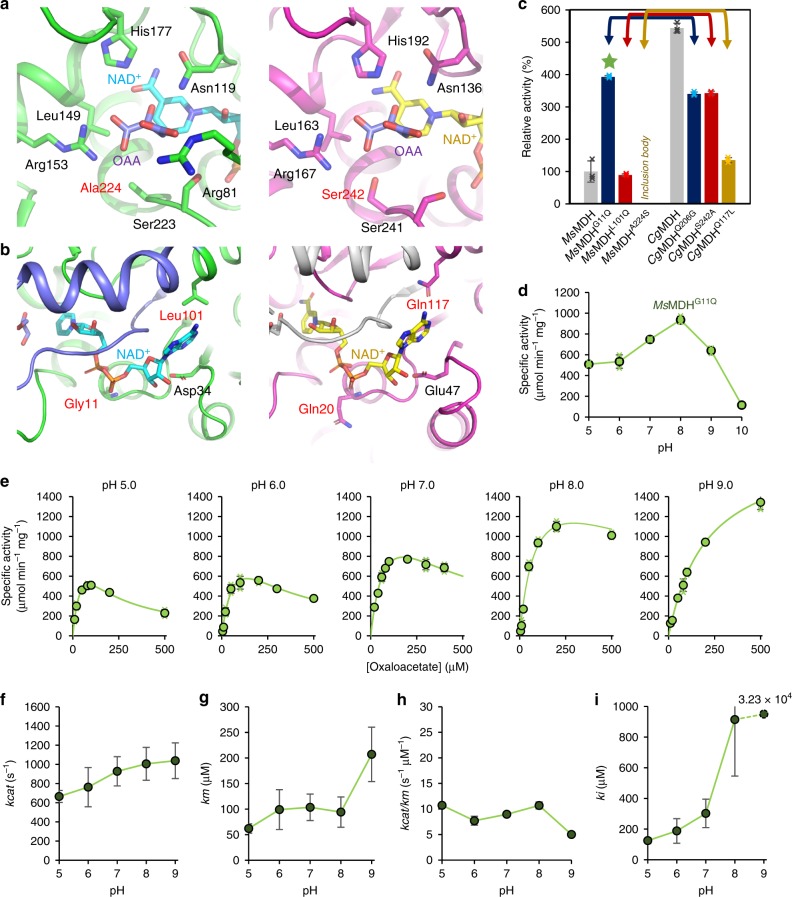
Development of highly efficient *Ms*MDH^G11Q^ variant based on structural comparison between *Ms*MDH and *Cg*MDH. **a** The OAA/malate binding site and **b** NADH/NAD^+^ binding site of the two crystal structures; *Ms*MDH (left, green model) and *Cg*MDH (right, magenta model). The conformation of OAA is obtained from a superimposed structure of *Methylobacterium extorquens* MDH (PDB code 4ROS). The mobile loop is distinguished by different color schemes of light blue (*Ms*MDH) and gray (*Cg*MDH). The observed residual differences are indicated by red color. **c** Site-directed mutagenesis and the relative activities of the *Ms*MDH and *Cg*MDH variants in comparison with the activity of *Ms*MDH (*n* = 3 independent experiments). Data are presented as mean values ± standard deviation. Each of the corresponding variants are indicated by the same color scheme and arrow. The *Ms*MDH^A224S^ variant, which is labeled as ‘Inclusion body’, was expressed insoluble. The *Ms*MDH^G11Q^ variant is indicated by a green star. **d** Optimal pH of the *Ms*MDH^G11Q^ variant (*n* = 3 independent experiments). Data are presented as mean values ± standard deviation. The specific activity at pH 10.0 was determined from a single data. **e** Catalytic performance of the *Ms*MDH^G11Q^ variant at different pH (*n* = 3 independent experiments). Data are presented as mean values ± standard deviation. **f** *kcat*, **g** *km*, **h** *kcat/km*, and **i** *ki* values of the *Ms*MDH^G11Q^ variant at different pH. The *ki* value of the *Ms*MDH^G11Q^ variant at pH 9.0 is shown in number because its value is significantly higher than the rest of the *ki* values. Data **f**–**i** are presented as parameters ± standard error. The standard errors from determining the kinetic parameters using OriginPro 2019 software (*n* = the number of mean velocity data at specific pH) are shown as bars.

The crystal structures of *Ms*MDH and *Cg*MDH were determined (Supplementary Fig. 6 and Supplementary Table 6) and detailed structural comparison was carried out. As enzyme active site is directly related to kinetic properties, the crystal structures of *Ms*MDH and *Cg*MDH were superimposed to find structural differences near the active sites (Supplementary Fig. 7). The two structures, representing the mitochondrial and cytosolic MDHs, were identified to be quite different from each other with a root-mean-square deviation value of 2.77 (Supplementary Fig. 7). Noticeable differences in the substrate (OAA or malate) and cofactor (NADH or NAD^+^) binding sites of *Ms*MDH and *Cg*MDH were found from the conformation of mobile loop^36^ and the lengths of β7-β8, α2-β2, and α7-α8 connecting loops (Supplementary Fig. 8). The β7-β8, α2-β2, and α7-α8 connecting loops include a catalytic histidine residue, the NADH-stabilizing residues, and the OAA-binding residues, respectively (Supplementary Fig. 8). As the connecting loops are directly involved in catalysis and substrate-binding, it was speculated that the structural differences between *Ms*MDH and *Cg*MDH were responsible for significant differences in enzyme kinetics. However, such kinetic discrepancy is an outcome of complex combinatorial effect of the whole enzyme structure. Thus, the reason for such difference in enzyme kinetics cannot be simply identified by enzyme mutagenesis study. Instead, differences of amino acid residues in the two MDHs were analyzed to identify a key residue that affects the performance of OAA reduction (Fig. 2a, b).

Comparative analysis of the OAA and malate binding site (the α7-α8 connecting loop) revealed that Ala224 in *Ms*MDH corresponded to Ser242 in *Cg*MDH (Fig. 2a,b). Also, Leu101 in *Ms*MDH, a residue involved in the binding of adenine moiety of NADH, corresponded to Gln117 in *Cg*MDH (Fig. 2a, b). Presence of a protruding Gln20 in *Cg*MDH was also observed at the pyrophosphate moiety binding site, whereas *Ms*MDH was found to possess Gly11 instead (Fig. 2a, b). Based on three residual differences, each of these three residues differently found in *Ms*MDH and *Cg*MDH were swapped at the corresponding site to construct six mutant MDHs for key residue identification. The relative activities of OAA reduction by six *Ms*MDH and *Cg*MDH variants, except for the *Ms*MDH^A224S^ variant, which formed inclusion bodies in *E. coli*, were analyzed in comparison with the activity of *Ms*MDH at pH 7.0 and compared with those of the wild-type enzymes. One of the variants, *Ms*MDH^G11Q^, showed 2.9-fold higher activity compared with *Ms*MDH, whereas the activity of the *Cg*MDH^Q20G^ variant (the counterpart variant of *Ms*MDH^G11Q^) was reduced compared with *Cg*MDH (Fig. 2c). All the other variants showed reduced activities compared with their corresponding wild-type MDHs (Fig. 2c). These results suggested that the replacement of Gly11 in *Ms*MDH with Gln (e.g., Gln20 of *Cg*MDH) endowed *Ms*MDH with high overall catalytic performance observed with *Cg*MDH (Fig. 2c). A study on kinetic analysis of the MDH from *Phycomyces blakesleeanus*, which is known to have glycine residue at its 11th position, revealed that substrate inhibition prevents NADH binding^34^. This finding is similar to our result in that the change in NADH binding motif (replacement of Gly11 *Ms*MDH with Gln) affected substrate inhibition. However, kinetic studies on *Ms*MDH and the *Ms*MDH^G11Q^ variant toward NADH revealed similar results with those of mitochondrial MDH from *Cryptococcus neoformans*, which exhibited uncompetitive substrate inhibition (Supplementary Fig. 9 and Supplementary Table 7)^35^.

Next, detailed kinetic characteristics of the *Ms*MDH^G11Q^ variant were analyzed at pH 5.0–9.0 (Fig. 2d–i). First, the optimal activity of the *Ms*MDH^G11Q^ variant was observed at pH 8.0 (Fig. 2d), which was reduced below the optimal pH of wild-type *Ms*MDH (Fig. 1c). Second, the *kcat* values were significantly increased for the entire pH range examined (Fig. 2f and Table 1). Third, the *Ms*MDH^G11Q^ variant showed reduced susceptibility to substrate inhibition represented by higher *ki* value (Fig. 2i and Table 1). Fourth, the *km* values were significantly increased and the catalytic efficiency was reduced compared with *Ms*MDH (Fig. 2g, h and Table 1). Although the fourth characteristic of the *Ms*MDH^G11Q^ variant is undesirable, the other three improved characteristics, in particular higher *kcat* and *ki* values, suggest that this variant might be superior to *Ms*MDH. In summary, a key residue for high kinetic efficiency in *Ms*MDH was discovered and the *Ms*MDH^G11Q^ variant with high activity and less substrate inhibition compared with its wild type was developed based on comparative structural analysis of *Ms*MDH and *Cg*MDH.

### SA production by employing *Cg*MDH and *Ms*MDH^G11Q^ variant

Fed-batch fermentations of the engineered strains employing the highly catalytic MDHs, *Cg*MDH or *Ms*MDH^G11Q^ variant, were performed to evaluate the actual SA production performances. It should be mentioned that all fed-batch fermentations were performed in duplicate to confirm reproducibility.

Before testing the effect of replacing *Ms*MDH with *Cg*MDH or *Ms*MDH^G11Q^ variant on SA production in vivo, we first examined whether *msmdh* gene overexpression is beneficial to SA production. In silico simulation suggested that reinforcement of metabolic flux from OAA to malate is advantageous for enhanced SA production (Supplementary Fig. 1 and Supplementary Table 1). Thus, the PALK strain^15^ overexpressing the *msmdh* gene was developed (Supplementary Tables 8, 9). Fed-batch fermentation of the PALK (pMS3-msmdh) strain in a chemically defined medium (CDM) using glucose as a carbon source produced 79.07 g L^−1^ of SA with the yield and productivity of 1.23 mol mol^−1^ glucose and 3.26 g L^−1^ h^−1^, respectively (Supplementary Fig. 10a and Supplementary Data 2). For comparison, fed-batch fermentation of the PALK (pMS3) strain, the PALK strain harboring an empty vector, produced 74.56 g L^−1^ of SA with yield and productivity of 1.11 mol mol^−1^ glucose and 3.03 g L^−1^ h^−1^, respectively, under the same fed-batch culture condition (Supplementary Data 2)^17^. Overexpression of *msmdh* in the PALK strain enhanced SA production as predicted by in silico simulation, but the improvement was not significant. To examine whether expressing the gene encoding one of the two highly efficient MDHs, *Cg*MDH and the *Ms*MDH^G11Q^ variant, allows enhanced SA production, two engineered *M. succiniciproducens* PALK strains expressing the corresponding genes (*cgmdh* and *msmdh*^G11Q^) were constructed (Supplementary Tables 8, 9). Fed-batch culture of the PALK (pMS3-cgmdh) strain produced 87.23 g L^−1^ of SA with yield and productivity of 1.29 mol mol^−1^ glucose and 3.6 g L^−1^ h^−1^, respectively (Supplementary Fig. 10b and Supplementary Data 2); the titer, yield, and productivity all significantly increased by amplifying the *cgmdh* gene. These results suggest that increasing conversion rate of OAA to malate by *Cg*MDH possessing 4.4-fold higher activity than *Ms*MDH (Fig. 1b) as well as much less substrate inhibition (Fig. 1d) at physiological conditions (pH and OAA concentration) was both advantageous for enhanced SA production. The beneficial effect of employing highly efficient *Cg*MDH was also tested in wild-type *C. glutamicum* and *E. coli* strains, which are widely used in the industry for chemicals production. Both engineered strains overexpressing *cgmdh* showed enhanced SA production (Supplementary Table 10), suggesting that the highly active *Cg*MDH is indeed beneficial for enhancing the conversion of OAA to malate regardless of the microbial strain.

Next, two PALK derivative strains expressing the *cgmdh*^Q20G^ (encoding *Cg*MDH^Q20G^) and *msmdh*^G11Q^ genes were constructed (Supplementary Tables 8, 9) to compare the effect of expressing the *msmdh*^G11Q^ gene and its counterpart variant (*cgmdh*^Q20G^) gene on SA production. Fed-batch fermentation of the PALK (pMS3-cgmdh^Q20G^) strain produced 79.39 g L^−1^ of SA with the yield and productivity of 1.0 mol mol^−1^ glucose and 3.27 g L^−1^ h^−1^, respectively (Supplementary Fig. 10c and Supplementary Data 2). The SA production performance had significantly decreased compared with the PALK (pMS3-cgmdh) strain, indicating decreased catalytic performance of substituting Gln20 of *Cg*MDH with glycine (Fig. 2c). On the other hand, fed-batch fermentation of the PALK (pMS3-msmdh^G11Q^) strain produced 84.19 g L^−1^ of SA with the yield and productivity of 1.08 mol mol^−1^ glucose, and 3.48 g L^−1^ h^−1^, respectively (Supplementary Fig. 10d and Supplementary Data 2), which is an improved SA production performance compared with the PALK (pMS3-msmdh) strain (Supplementary Fig. 10a and Supplementary Data 2). This result suggests that the *Ms*MDH^G11Q^ variant allows more efficient in vivo conversion of OAA to malate compared with *Ms*MDH. Taken together, the expression of a gene encoding the superior *Cg*MDH or the *Ms*MDH^G11Q^ variant, instead of *Ms*MDH, in *M. succiniciproducens*, which consequently led to enhanced SA production. Also, the importance of glutamine residue at the position of Gln20 in *Cg*MDH and Gly11 in *Ms*MDH in enhanced conversion of OAA to malate was confirmed in vivo.

Recombinant strains harboring plasmids are not preferred in industrial fermentation due to the possibility of plasmid instability in the absence of antibiotic selection pressure^37^. Thus, we pursued to construct a plasmid-free SA producer by chromosomal integration of the superior *cgmdh* or *msmdh*^G11Q^ gene. The PALKcgmdh and PALKmsmdh^G11Q^ strains were constructed by replacing the *msmdh* gene in the PALK strain genome with the *cgmdh* or *msmdh*^G11Q^ gene under the stronger *frd* promoter (Supplementary Tables 8, 9; see Methods for chromosomal integration)^38^. The SA production performance indices obtained with fed-batch fermentation of the PALKcgmdh (Supplementary Fig. 10e and Supplementary Data 2) and PALKmsmdh^G11Q^ (Supplementary Fig. 10f and Supplementary Data 2) strains in CDM containing glucose were comparable to those achieved with the PALK (pMS3-cgmdh) (Supplementary Fig. 10b) and PALK (pMS3-msmdh^G11Q^) (Supplementary Fig. 10d) strains, respectively, which express the *mdh* genes from the plasmids. It is interesting to observe that chromosomal replacement of the *msmdh* with *cgmdh* or *msmdh*^G11Q^ allowed high SA production similar to those achieved by the PALK (pMS3-cgmdh) and PALK (pMS3-msmdh^G11Q^) strains, even though these latter strains additionally harbor *msmdh* gene in the chromosome. Thus, enhanced SA production by the PALK (pMS3-cgmdh) and PALK (pMS3-msmdh^G11Q^) strains seems primarily due to the introduction of *Cg*MDH or *Ms*MDH^G11Q^, both of which possess higher *kcat* and *ki* values than *Ms*MDH. In addition, fed-batch fermentation of the PALKPfrdmsmdh strain, which was constructed by replacing the native promoter of *msmdh* gene with the *frd* promoter in the PALK strain, showed similar SA production performance with the PALK strain (Supplementary Fig. 11 and Supplementary Data 2), suggesting that the enhanced SA production by replacing *msmdh* with *cgmdh* or *msmdh*^G11Q^ was not due to the use of stronger *frd* promoter, but rather because of the higher MDH activity. Furthermore, specific activity of the cell extract of PALKcgmdh strain was 1.5-fold higher than that of the PALK strain, suggesting that the replacement of *msmdh* gene with the *cgmdh* gene in *M. succiniciproducens* genome is beneficial for SA production (Supplementary Fig. 12).

### Optimizing culture condition to improve SA production

**Fig. 3:**
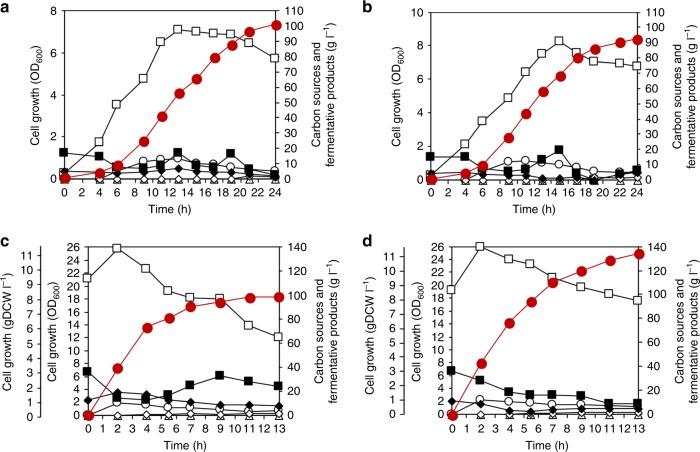
Fed-batch fermentation profiles of various metabolically engineered *M. succiniciproducens* strains. Fed-batch fermentations of the **a** PALKcgmdh and **b** PALKmsmdh^G11Q^ strains were carried out in CDM using glucose and glycerol as dual carbon sources. Fed-batch fermentations of the **c** PALK and **d** PALKcgmdh strains were carried out using glucose and glycerol with increased initial cell mass (OD_600_ = 21.1 and 19.3, respectively). Symbols: White square, cell growth; red circle, SA; black square, glucose; black diamond, glycerol; white circle, pyruvate; white diamond, acetate; white triangle, formate. Fermentations were all performed in duplicate (*n* = 2 independent experiments). The fermentation profile shown here represents the result of one fed-batch culture, whereas the result of another reproduced fed-batch culture is shown in Supplementary Fig. 15. Source data are provided as a Source Data file.

To further improve SA production by fed-batch fermentations of the PALKcgmdh and PALKmsmdh^G11Q^ strains, culture conditions were optimized. Based on our previous studies, glucose and glycerol were used as dual carbon sources to enhance SA production by taking full advantage of a highly efficient *Cg*MDH or *Ms*MDH^G11Q^ through providing more reducing equivalents^9,39^; conversion of glycerol (C3) to PEP (C3) generates twice as much reducing equivalents per six-carbon equivalent mole compared with glucose (C6)^40^. Metabolic fluxes of the PALK strain generated from glucose or glucose plus glycerol were compared using in silico simulation to verify enhanced NADH generation (glycerol 3-phosphate dehydrogenase and glyceraldehyde 3-phosphate dehydrogenase reactions). In silico simulation (Supplementary Fig. 13) and kinetic studies on *Ms*MDH and the *Ms*MDH^G11Q^ variant (Supplementary Fig. 9 and Supplementary Table 7) suggested that the OAA to malate conversion flux increased through the elevated supply of NADH. Fed-batch fermentation of the PALKcgmdh strain in CDM using glucose and glycerol as dual carbon sources produced 101.18 g L^−1^ of SA with the yield and productivity of 1.37 mol mol^−1^ glucose equivalent (mol SA per mol glucose equivalent) and 4.18 g L^−1^ h^−1^, respectively (Fig. 3a and Supplementary Data 2). These results were similar to those obtained by fed-batch fermentation of the plasmid-based PALK (pMS3-cgmdh) strain using dual carbon sources (Supplementary Data 2). On the other hand, fed-batch fermentation of the PALKmsmdh^G11Q^ strain in a CDM using dual carbon sources produced 92.5 g L^−1^ of SA with the yield and productivity of 1.28 mol mol^−1^ glucose equivalent and 3.82 g L^−1^ h^−1^, respectively (Fig. 3b and Supplementary Data 2). Although SA production did not improve as much as the PALKcgmdh strain, the overall SA production indices were significantly increased compared with the previously reported PALK strain cultured under the same condition (Supplementary Data 2)^15^. Taken together, the PALKcgmdh strain showed the best SA production performance, and thus was used for further studies.

One of the fermentation characteristics of *M. succiniciproducens* is its low cell density during the fermentation. In the cases of the PALK (pMS3), PALKPfrdmsmdh, PALK (pMS3-msmdh), PALK (pMS3-cgmdh), PALK (pMS3-msmdh^G11Q^), PALK (pMS3-cgmdh^Q20G^), PALKmsmdh^G11Q^, and PALKcgmdh strains, the maximum cell densities reached during fermentations using glucose were 4.87, 4.56, 4.87, 3.53, 3.21, 3.27, 4.46, and 3.26 gDCW L^−1^, respectively. On the other hand, the specific SA productivities obtained for these strains were 0.62, 0.67, 0.67, 1.02, 1.08, 1.0, 0.76, and 1.17 g gDCW^−1^ h^−1^, respectively (Supplementary Data 2). Due to exceptionally high specific SA productivities of these engineered strains, the overall SA productivities are very high even though cell densities reached were far lower than other SA producers such as *E. coli* and *C. glutamicum* (Discussion). As cells are themselves biofactories, the overall productivity can be further increased if cell density is increased. Thus, higher cell density inoculums (the OD_600_ of 21.1 equivalent to 9.52 gDCW L^−1^ for PALK and the OD_600_ of 19.3 equivalent to 8.7 gDCW L^−1^ for PALKcgmdh) were employed for SA production by fed-batch cultures (Fig. 3c, d). High-inoculum fed-batch culture of the PALK strain produced 98.28 g L^−1^ of SA, with the yield, overall productivity, and maximum productivity of 0.93 mol mol^−1^ glucose, 8.93 and 18.4 g L^−1^ h^−1^ (Supplementary Data 2), respectively, using dual carbon sources. The specific SA productivity obtained with high-inoculum fed-batch culture was 0.94 g gDCW^−1^ h^−1^, similar to that obtained with normal fed-batch culture (1.0 g gDCW^−1^ h^−1^). In the case of the PALKcgmdh strain, the overall and maximum SA productivities were significantly increased to 10.33 and 21.3 g L^−1^ h^−1^, respectively, using dual carbon sources (Supplementary Data 2) due to the strain’s superior specific SA productivity of 1.19 g gDCW^−1^ h^−1^. In addition, the PALKcgmdh strain produced 134.25 g L^−1^ of SA with almost no byproduct resulting in a yield of 1.25 mol mol^−1^ glucose equivalent (Fig. 3d and Supplementary Data 2). This is the best overall performance reported to date for fermentative SA production^1^. Fermentations of metabolically engineered *M. succiniciproducens* strains developed in this study were all performed in duplicate to confirm reproducibility and as a standard way of presentation; the results of one representative fed-batch fermentation are shown in the main figure while those of the other fermentations are provided in Supplementary Data 2 and Supplementary Figs. 10 and 14–16.

## Discussion

For the purpose of increasing SA production, MDH that has important role in directing metabolic flux coming from the anaplerotic pathway toward SA production was investigated. Detailed kinetic analyses of *Ms*MDH elucidated its low activity at pH 5.0–8.0 and its high susceptibility to substrate inhibition suggesting that *Ms*MDH might not be an optimal MDH for enhanced SA production.

The OAA reduction activity of *Cg*MDH was significantly higher than those of other mitochondrial MDH-like enzymes (Fig. 1b and Supplementary Fig. 2). Functional differences between cytosolic and mitochondrial MDHs are well understood in the malate–aspartate shuttle of eukaryotes^26^. In the shuttle system across mitochondrial inner membrane, whereas OAA reduction activity exists in both cytosolic and mitochondrial MDHs, the cytosolic MDH is known to have higher preference for converting OAA to malate by NADH oxidation relative to the mitochondrial MDH^24,25^, which is consistent with what we observed in this study (Fig. 1a and Supplementary Fig. 2). Moreover, the PALK strain expressing *Arabidopsis thaliana* cytosolic MDH (*At*MDHc1) produced higher amount of SA from fed-batch fermentation using glucose compared to the PALK strain expressing *A. thaliana* mitochondrial MDH (*At*MDHm1) suggesting once again that cytosolic MDH might be a better choice for SA production than mitochondrial MDH (Supplementary Fig. 16 and Supplementary Data 2). However, *Cg*MDH was still the best MDH for highest SA production performance in *M. succiniciproducens*.

Structural analyses of *Cg*MDH and *Ms*MDH provided basis to enhance *kcat* and to alleviate substrate inhibition of *Ms*MDH. The Gly11 of *Ms*MDH and Gln20 of *Cg*MDH, which take part in the binding of pyrophosphate moiety of NADH/NAD^+^, were identified as a key residue that determines the OAA reduction activity and susceptibility to substrate inhibition (Fig. 1a, b and Table 1). The *Ms*MDH^G11Q^ variant developed based on structural analyses exhibited significantly increased *kcat* and higher *ki* values compared with its wild type (Table 1). Reduction or elimination of substrate inhibition of several dehydrogenases by single mutation in the active site has been reported^41^.

Replacement of *Ms*MDH with highly efficient *Cg*MDH in the *M. succiniciproducens* PALK strain resulted in the highest overall SA production titer, yield, and productivity reported to date (Supplementary Data 2). Moreover, a bioprocess engineering approach of employing high cell density inoculum fed-batch culture was taken to further increase the overall SA production indices (titer: 134.25 g L^−1^, yield: 1.25 mol mol^−1^ glucose equivalent, productivity: 10.33 g L^−1^ h^−1^; Supplementary Data 2). SA production by other microorganisms, such as *E. coli* and *C. glutamicum*, often employs dual phase fermentation technique, which involves aerobic growth phase to reach high cell density first followed by anaerobic production phase to produce SA. The *E. coli* AFP111 strain expressing the *Rhizobium etli* pyruvate carboxylase gene was first aerobically grown up to 10.2 gDCW L^−1^ and then subjected to anaerobic condition to produce 99.2 g L^−1^ of SA with an overall productivity of 1.3 g L^−1^ h^−1^. The specific SA productivity of 0.14 g gDCW^−1^ h^−1^ was obtained^12^. Similarly, *C. glutamicum* BOL-3 strain overexpressing the glyceraldehyde 3-phosphate dehydrogenase gene was cultured until cell density reached 50 gDCW L^−1^, then was further cultured under anaerobic condition to produce 134 g L^−1^ of SA with an overall productivity of 2.48 g L^−1^ h^−1^. The specific SA productivity of 0.05 g gDCW^−1^ h^−1^ was obtained^7^. Thus, it can be clearly seen that *M. succiniciproducens* PALK and MDH amplified PALK strains possess much higher specific SA productivities than these popular SA producers. In addition, introduction of *Cg*MDH into *E. coli* and *C. glutamicum* strains confirmed that the highly active *Cg*MDH is beneficial for enhanced SA production regardless of the microbial strain (Supplementary Table 10). Although MDH was the focus of this study, it is possible that the introduction of more efficient *Cg*MDH into *M. succiniciproducens* might have affected the activities of other enzymes and corresponding reaction fluxes (see Supplementary Note 2 for detailed discussion). As identified from in silico genome-scale metabolic simulation (Supplementary Fig. 1 and Supplementary Table 1), PCKA, FUMC, and FRD can be the next potential candidates to be studied for further enhancing SA production using similar approaches reported here.

In conclusion, we developed a highly efficient SA producing *M. succiniciproducens* strain by integrating the strategies of systems metabolic engineering with enzyme engineering. The use of the best MDH selected from various MDHs through detailed structural and kinetic studies significantly improved SA production. The strategy described in this paper will be useful for developing high performance strains for the production of industrially competitive bio-based chemicals.

## Methods

### Strains and plasmids

Strains, plasmids, and oligonucleotides used in this study are listed in Supplementary Tables 8, 9. The *E. coli* Top10 and *E. coli* W3110 strains were used as a cloning host for gene manipulation and for SA production, respectively. The *E. coli* strains were cultivated at 37 °C in lysogeny broth (LB) medium containing (per L) 10 g Bacto tryptone, 5 g yeast extract, and 10 g NaCl. The *C. glutamicum* strain was also used for SA production and was cultivated under 30 °C in BHIS medium containing (per L) 37 g Bacto Brain Heart Infusion (BHI) medium and 91 g d-sorbitol. The *M. succiniciproducens* strains were cultivated under 39 °C in BHI medium. The LB and BHI plates were prepared by the addition of 1.5% (w/v) of agar. When needed, ampicillin (Ap), kanamycin (Km), and chloramphenicol (Cm) were added to the final concentrations of 50, 25, and 6.8 μg mL^−1^, respectively, for *M. succiniciproducens*, and 50, 25, and 24 μg mL^−1^, respectively, for *E. coli*. In the case of *C. glutamicum*, 25 μg mL^−1^ Km was added to BHIS medium.

To construct pMS3-msmdh, pMS3-msmdh^G11Q^, pMS3-cgmdh, pMS3-cgmdh^Q20G^, pMS3-atmdhc1, and pMS3-atmdhm1, the *msmdh* (primers P1-2), *msmdh*^G11Q^ (primers P1-2), *cgmdh* (primers P3-4), *cgmdh*^Q20G^ (primers P3-4), *atmdhc1* (primers P5-6), and *atmdhm1* (primers P7-8) gene fragments were prepared by polymerase chain reaction (PCR) with primers listed in Supplementary Tables 8, 9 using *M. succiniciproducens* genomic DNA, pET30a*-*msmdh^G11Q^, *C. glutamicum* genomic DNA, pET30a*-*cgmdh^Q20G^, and *A. thaliana* genomic DNA respectively, as templates. Each gene fragments were then inserted into pMS3 digested with *Eco*RI and *Kpn*I using Gibson assembly^42^. To construct p10099A-cgmdh, p10099A was digested with *Eco*RI and *Pst*I and the *cgmdh* gene fragment was prepared by PCR with primers P18-19. The linearized p10099A and the *cgmdh* gene fragment were assembled using Gibson assembly. To construct pEKEx1-cgmdh, pEKEx1 was digested with *Eco*RI and *Pst*I and the *cgmdh* gene fragment was prepared by PCR with primers P20-21. The linearized pEKEx1 and the *cgmdh* gene fragment were assembled using Gibson assembly. Correct construction of all plasmids developed in this study was verified using DNA sequencing. Plasmids pMS3-msmdh, pMS3-msmdh^G11Q^, pMS3-cgmdh, pMS3-cgmdh^Q20G^, pMS3-atmdhc1, and pMS3-atmdhm1 were transformed to PALK strain^43^.

The *M. succiniciproducens* PALKcgmdh strain was constructed by replacing *msmdh* gene in the PALK strain genome with *cgmdh* gene. To construct the *cgmdh* gene integration vector pINcgmdh, the plasmid pSacHR06 containing the *sacB* gene was digested with *Xho*I and *Sac*I. Next, the upstream and downstream homologous regions of the *msmdh* gene were amplified from the *M. succiniciproducens* PALK genomic DNA with primers P9-10 and P16-17, respectively. The *lox66*-*cat*-*lox77* cassette was amplified from pMSmulox with primers P14-15 and the *cgmdh* gene was amplified from pMS3-cgmdh with primers P11 and P13. The linearized pSacHR06, 1 kb fragments of the upstream and downstream homologous regions of the *msmdh* gene, *lox66*-*cat*-*lox77* cassette, and *cgmdh* gene fragments were assembled using Gibson assembly to finally construct pINcgmdh. The *M. succiniciproducens* PALKmsmdh^G11Q^ strain was constructed by replacing *msmdh* gene in the PALK strain with the *msmdh*^G11Q^ gene. The *msmdh*^G11Q^ gene integration vector pINmsmdh^G11Q^ was constructed using the DNA fragments utilized to construct pINcgmdh except for *msmdh*^G11Q^ gene, which was amplified from pMS3-msmdh^G11Q^ with primers P11-12. The *M. succiniciproducens* PALKPfrdmsmdh strain was constructed by replacing the promoter of *msmdh* (P_*mdh*_) in the PALK strain with the promoter of *frd* (P_*frd*_). To construct the P_*frd*_ sequence integration vector pINPfrdmsmdh, the plasmid pSacHR06 containing the *sacB* gene was digested with *Xho*I and *Sac*I. Next, the upstream and downstream homologous regions of the P_*mdh*_ sequence were amplified from the *M. succiniciproducens* PALK genomic DNA with primers P22-23 and P24-25, respectively. The *lox66*-*cat*-*lox77* cassette was amplified from pMSmulox with primers P26-27 and the P_*frd*_ sequence was amplified from pMS3 with primers P28-29. The linearized pSacHR06, 1 kb fragments of the upstream and downstream homologous regions of the P_*mdh*_ sequence, *lox66*-*cat*-*lox77* cassette, and P_*frd*_ sequence fragments were assembled using Gibson assembly to finally construct pINPfrdmsmdh. Heterologous gene integrations into the PALK genome were carried out using the markerless chromosomal integration system^43^.

### In silico analysis

Flux variability scanning based on enforced objective flux (FVSEOF) algorithm^44^ was performed using the *M. succiniciproducens* genome-scale metabolic model that consists 686 metabolic reactions and 519 metabolites^39^ to identify amplification target genes for enhanced SA production in *M. succiniciproducens* (Supplementary Fig. 1a). FVSEOF algorithm searches the changes in metabolic flux solution space (i.e., min and max values of metabolic flux) of intracellular reaction in response to an increased flux toward the target chemical. Based on FVSEOF results, candidate enzymes that would ensure a minimal production rate of target chemical were selected from the slope of minimum flux values (*V*_min_ slope), which was calculated by a linear regression between the enforced SA production rate and the minimal flux values (*V*_min_) of the intracellular reactions (Supplementary Fig. 1b)^45^. The reactions that positively related with SA production rate were selected as target enzymes. Throughout the simulation, the glucose uptake rate was set to 10 mmol gDCW^−1^ h^−1^. Among 686 metabolic reactions in *M. succiniciproducens* genome-scale model, only four reactions showed increased flux patterns of minimum flux values for SA production; FRD, PCKA, MDH, and FUMC (Supplementary Table 1).

In order to compare the amount of NAD^+^/NADH produced and consumed by the PALK strain from utilizing single (glucose) or dual (glucose and glycerol) carbon sources, in silico flux-sum analysis of the entire 515 cytosolic metabolites^46^ were carried out (Supplementary Fig. 13a). Flux-sum is the summation of all fluxes in or out of each metabolite. The cytosolic metabolites were only considered for flux-sum analysis to focus on glucose/glycerol metabolism taking place inside the cell. Parsimonious flux balance analysis (pFBA) was performed to calculate flux distributions generated from single or dual carbon source utilization while maximizing biomass production as an objective^47^ (Supplementary Fig. 13b). The *ldhA*, *pta*, and *ackA* genes were deleted to mimic the genotype of PALK strain. Flux variability analysis (FVA)^48^ was performed to calculate the upper and lower bounds of fluxes under 95% maximal growth rate with loopless solution^49^. To prevent the upper and lower bounds from being unrealistic, additional constraint was given to limit the sum of all absolute fluxes in FVA solution to be no larger than 10% of the sum of all absolute fluxes in pFBA solution. Throughout the simulation where glucose was used as a sole carbon source, the glucose uptake rate was set to 10 mmol gDCW^−1^ h^−1^. For the simulation where glucose and glycerol were utilized as dual carbon sources, the glucose and glycerol uptake rates were set to five and 10 mmol gDCW^−1^ h^−1^, respectively. The uptake rates of dual carbon sources were set different to equate the number of carbons (glucose, six carbons; glycerol, three carbons) entering the metabolic system. All simulations were conducted in Python environment with Gurobi Optimizer 6.0 and GurobiPy package (Gurobi Optimization Inc., Houston, TX, USA). Reading, writing, and manipulation of the COBRA-compliant SBML files were implemented using COBRApy^50^.

### Protein preparations

The genes encoding *Ms*MDH (primers P30-31), *As*MDH (primers P32-33), *Cg*MDH (primers P34-35), *Ec*MDH (primers P36-37), *Yl*MDH (primers P38-39), mitochondrial *Sc*MDH1 (primers P40-41), cytosolic *Sc*MDH2 (primers P42-43), and glyoxysomal *Sc*MDH3 (primers P44-45) from *M. succiniciproducens*, *A. succinogenes*, *C. glutamicum*, *E. coli*, *Y. lipolytica*, and *S. cerevisiae* were amplified from their chromosomal DNAs by PCR with primers listed in Supplementary Tables 8, 9. The PCR products were then inserted into pET30a (Novagen, Madison, WI, USA) with 6×His at the C terminus. The resulting expression vectors were transformed into the *E. coli* BL21 (DE3)^T1R^ strain and were grown in LB medium containing 100 mg L^−1^ Km at 37 °C to an OD_600_ (optical density at 600 nm) of 0.6. After induction with 1.0 mM 1-thio-β-d-galactopyranoside (IPTG), cells were further cultured for 20 h at 18 °C and harvested by centrifugation at 5000×*g* for 15 min at 4 °C. The cell pellet was resuspended in 40 mM Tris–HCl at pH 8.0 and disrupted by ultra-sonication. The cell debris was removed by centrifugation at 11,000×*g* for 1 h and the lysate was bound to Ni-NTA agarose column (Qiagen, Chatsworth, CA, USA). After washing with 40 mM Tris–HCl containing 20 mM imidazole at pH 8.0, the proteins bound to the resin were eluted with 300 mM imidazole in 40 mM Tris–HCl at pH 8.0. Further purification was carried out using HiTrap Q ion exchange chromatography and size exclusion chromatography. The purified proteins were concentrated to 30 g L^−1^ in 40 mM Tris–HCl at pH 8.0 for crystallizations. Site-directed mutagenesis was performed using the QuikChange site-directed mutagenesis kit (Stratagene, La Jolla, CA, USA).

### In vitro malate dehydrogenase activity assay

The activity of MDH was measured using a spectrophotometer at 340 nm by analyzing residual concentration of NADH (extinction coefficient of 6.22 mM^−1^ cm^−1^)^51^. The relative activities of MDHs in comparison with the activity of *Ms*MDH were measured using 0.5 mL of reaction mixture containing 0.1 M Tris–HCl at pH 7.0, 200 μM NADH, 100 μM oxaloacetate, and 3 nM of various MDHs. For measurement of activity at various pH, the same reaction mixture containing 0.1 M BIS-Tris or CHES buffer were used for pH 5.0–6.0 and 9.0–10.0, respectively, instead of 0.1 M Tris–HCl. Based on the plotted kinetic data, the kinetic parameters were determined from non-linear regression analyses based on the modified Briggs-Haldane equation^35,52^ using OriginPro 2019 software (OriginLab, Northampton, MA, USA). All experiments were performed in triplicate at room temperature.

### Cell extract assay

The enzyme activities in the cell extracts of *M. succiniciproducens* PALK and PALKcgmdh strains were measured using a spectrophotometer at 340 nm by analyzing residual concentration of NADH. The cell extract was obtained by collecting the cells (OD_600_ of 50) grown up to late exponential phase using centrifugation at 5566×*g* and 4 °C, washing the cells twice with 0.1 M Tris–HCl at pH 7.0, and sonication in a final volume of 25 mL. The enzyme activity in cell extract was measured at pH 7.0 using 0.5 mL of reaction mixture containing 0.1 M Tris–HCl, 200 μM NADH, 100 μM OAA, and 10 μL of cell extract lysate. The concentrations of the total proteins in the reaction mixtures containing the PALK and PALKcgmdh cell lysates were 8.8 and 7.6 μg mL^−1^, respectively.

### Crystallization and structure determination

Crystallization of the purified proteins was initially performed using the following crystal screening kits: Index and PEG/Ion (Hampton Research) and Wizard I and II (Rigaku) using the hanging-drop vapor-diffusion technique at 20 °C. Drop size was 2 μL, which includes 1 μL of protein solution and 1 μL of reservoir solution, and the drop was equilibrated against 50 μL of the reservoir solution. The *Ms*MDH crystals, co-crystallized with NAD^+^ (molar ratio 1:10) to capture the reaction product inside the *Ms*MDH crystal structure, appeared in 16% (w/v) PEG 3350 and 6% (v/v) tacsimate at pH 6.0. The cryoprotectant solution was a mixture of 16% (w/v) PEG 3350, 6% (v/v) tacsimate at pH 6.0, and 30% (v/v) glycerol. Data were collected under 100 K at Beamline 7A of the Pohang Accelerator Laboratory (Pohang, Republic of Korea)^53^. Subsequently, the data were indexed, integrated, and scaled using the HKL2000 software suite^54^. The *Ms*MDH crystals belonged to a space group P6422 with unit cell parameters of *a* = 80.09 Å, *b* = 80.09 Å, and *c* = 193.15 Å; *α* = *β* = 90° and *γ* = 120°. With one molecule of *Ms*MDH per asymmetric unit, the Matthews coefficient was ~2.58 Å^3^ Da^−1^, which corresponds to a solvent content of ~52.04 %^55^. The structure of *Ms*MDH was determined by molecular replacement with the CCP4 version of MOLREP^56^ using the structure of an MDH from *Haemophilus influenza*e (PDB code 6BAL, 77% sequence identity) as a search model. The model building was performed using the WinCoot program^57^ and the refinement was performed with REFMAC5^58^. The highest quality *Cg*MDH crystals co-crystallized with l-malate and NAD^+^ (molar ratio 1:20 and 1:10) appeared in 20% (w/v) PEG 3350, 0.1 M HEPES at pH 7.5, and 0.2 M MgCl_2_·6H_2_O. The cryoprotectant solution includes 20% (w/v) PEG 3350, 0.1 M HEPES at pH 7.5, 0.2 M MgCl_2_·6H_2_O, and 30% (v/v) glycerol. The *Cg*MDH crystals belonged to the space group C2 with unit cell parameters of *a* = 102.93 Å, *b* = 116.94 Å, and *c* = 66.00 Å; *α* = *γ* = 90° and *β* = 95.31°. Using one molecule of *Cg*MDH per asymmetric unit, the Matthews coefficient was ~2.87 Å^3^ Da^−1^, which corresponds to a solvent content of ~56.77%^55^. The structure of *Cg*MDH was determined by molecular replacement with the CCP4 version of MOLREP using the structure of a MDH from *Mycobacterium tuberculosis* (PDB code 4TVO, 57% sequence identity) as a search model. The model was built following the procedure described above. Statistical analyses of the data are summarized in Supplementary Table 6. The refined models of *Ms*MDH and *Cg*MDH were deposited in the Protein Data Bank with PDB codes 6ITL (10.2210/pdb6ITL/pdb) and 6ITK (10.2210/pdb6ITK/pdb), respectively.

### Maximum likelihood of phylogenetic tree

Iterative searching for MDH-like proteins was performed by Basic Local Alignment Search Tool (BLAST) in National Center for Biotechnology Information server using position-specific iterated BLAST method^59^. Multiple sequences alignment was performed by Clustal omega^60^. The evolutionary history was inferred using the Maximum Likelihood method based on the Le_Gascuel_2008 model^61^. Initial trees for the heuristic search were obtained automatically by applying Neighbor-Join and BioNJ algorithms to a matrix of pairwise distances estimated using a JTT model and then selecting the topology with superior log likelihood value. A discrete Gamma distribution was used to model evolutionary rate differences among sites (5 categories, parameter = 1.0385). The rate variation model allowed 1.33% sites to be evolutionarily invariable. The tree with the highest log likelihood (−58574.72) is shown in Supplementary Fig. 2. The phylogenetic tree is drawn to scale with branch lengths measured in the number of substitutions per site. The analysis involved 343 amino acid sequences. All positions with less than 95% site coverage were eliminated. That is, fewer than 5% alignment gaps, missing data, and ambiguous bases were allowed at any position. There were a total of 263 positions in the final dataset. Evolutionary analyses were conducted using MEGA X^62^.

### SA production using *C. glutamicum* and *E. coli* strains

The wild type and engineered *C. glutamicum* strains were aerobically cultivated in Erlenmeyer flask equipped with CO_2_ gas inlet and outlet ports. Each flasks contained 100 mL BHIS medium. The aerobic cultivation was carried out for 6 h at 30 °C with shaking because *C. glutamicum* strains cannot grow under anaerobic condition^7^. Then, IPTG was added to each flask to a final concentration of 0.5 mM to initiate *Cg*MDH expression. Finally, the flasks were charged with CO_2_ as headspace gas and incubated at 30 °C with shaking for 10 h. The wild type and engineered *E. coli* strains were anaerobically cultivated for 16 h in 100 mL LB medium with 3 g L^−1^ glucose at 37 °C with shaking. The initial cell concentrations of the wild type and engineered *C. glutamicum* and *E. coli* strains were OD_600_ of 0.2–0.25.

### Fermentation and analytical procedures

The *M. succiniciproducens* strains were precultured in a 50 mL tube or a 500 mL Erlenmeyer flask equipped with CO_2_ gas inlet and outlet ports. Each tube and flask contained 20 and 270 mL, respectively, of complex MH5 medium (per L: 2.78 g yeast extract, 2.78 g polypeptone, 0.18 g NaCl, 0.02 g CaCl_2_·2H_2_O, 0.2 g MgCl_2_·6H_2_O, and 8.06 g K_2_HPO_4_, and 9.15 g NaHCO_3_). After adjusting the pH of the complex medium to 7.0 using 5 M NaOH and flushing with CO_2_, it was heat sterilized at 121 °C for 15 min. Carbon sources (glucose and glycerol) were separately sterilized and added into the culture medium to a final concentration of 10 g L^−1^. Then, 2.5 mL of glycerol stock culture (15%, w/v), which was stored in a deep freezer at −70 °C, was inoculated into a 50 mL test tube containing complex medium and carbon source. The test tube charged with CO_2_ as headspace gas was incubated in a static incubator at 39 °C. Next, 5% (v/v) of the cultured cell broth was transferred to the 500 mL Erlenmeyer flask containing complex medium and carbon source for further cultivation using the same condition as test tube culture.

Fed-batch fermentations were carried out in a 6.6 L Bioflo 3000 bioreactor (New Brunswick Scientific Co., Edison, NJ, USA) with a working volume of 2.5 L. The CDM used in this study contained (per L) 1 g NaCl, 0.02 g CaCl_2_·2H_2_O, 2 g (NH_4_)_2_SO_4_, 0.5 g alanine, 0.5 g asparagine, 0.005 g biotin, 0.5 g methionine, 0.005 g Ca-pantothenate, 0.005 g pyridoxine-HCl, 0.005 g thiamine, 0.2 g MgCl_2_·6H_2_O, 1.5 g K_2_HPO_4_, 9.997 g NaHCO_3_, 0.005 g ascorbic acid, 0.5 g aspartic acid, 0.5 g cysteine, 0.005 g nicotinic acid, 0.5 g proline, 0.5 g serine, and 5 mL trace metal solution. The trace metal solution contained (per L) 5 mL HCl, 10 g FeSO_4_·7H_2_O, 2.25 g ZnSO_4_·7H_2_O, 1 g CuSO_4_·5H_2_O, 0.5 g MnSO_4_·5H_2_O, 0.23 g Na_2_B_4_O_7_·10H_2_O, and 0.1 g (NH_4_)_6_Mo_7_O_24_. The CDM was supplemented with 18.02 g L^−1^ (100 mM) glucose and/or 4.60 g L^−1^ (50 mM) glycerol. Antibiotics were added to the following concentration when necessary: Ap (50 mg L^−1^) and Km (25 mg L^−1^). The feeding solution was composed of 900 g L^−1^ glucose and/or glycerol. Fed-batch fermentation was initiated by inoculation of 300 mL of precultured broth, giving the initial OD_600_ of 0.2–0.3. Temperature and agitation speed of four flat blade turbine impellers in the bioreactor were controlled at 39 °C and 200 rpm, respectively. The pH of the fermentation broth was controlled at 6.5 by automatic addition of a mixture of 1.57 M ammonia solution and 6.84 M MgOH_2_ solution. The bioreactor was continuously sparged with industrial-grade CO_2_ gas at a flow rate of 0.2 vvm (CO_2_ volume per working volume per min) by a mass flow controller. Fed-batch fermentations were performed in a semi-continuous feeding mode, which the feeding solution was supplied into the bioreactor via a peristaltic pump, to maintain the carbon source concentration at 5–15 g L^−1^ for substrate inhibition minimization by changing the feeding rate. For higher inoculum fed-batch fermentation, cells were harvested from a 10 L batch fermentation and were resuspended using 200 mL of CDM. The rest of the fermentation procedures were identical to a normal fed-batch fermentation except for the initial glucose and glycerol concentrations, which were 36.4 and 9.2 g L^−1^, respectively.

The concentrations of glucose, glycerol, and fermentative products were immediately monitored using ProStar 210 HPLC (Varian, CA, USA) accompanied with ProStar 320 UV/visible-light (Varian) and Shodex RI-71 refractive index (Shodex, Tokyo, Japan) detectors over the entire period of fermentation. The MetaCarb 87 H column (300 × 7.8 mm; Agilent, CA, USA) was eluted isocratically (flow rate = 0.6 mL min^−1^) using 0.01 N H_2_SO_4_ at 60 °C. Cell growth was monitored by measuring OD_600_ using Ultrospec 3000 spectrophotometer (GE Healthcare, Chalfont St. Giles, UK). Optical density was converted to a cell concentration defined as gram dry cell weight (gDCW) using the predetermined standard curve (1 OD_600_ = 0.451 gDCW L^−1^). When glucose and glycerol were co-utilized, the SA yield was calculated based on glucose equivalent (mol SA per mol glucose equivalent) for clear comparison. As the number of carbons in glucose and glycerol differ by a factor of 2, the amount of glycerol consumed (mol) was converted to mol glucose equivalent to calculate the total amount of carbon sources consumed during the course of fed-batch fermentation.

### Measurement of intracellular pH

The intracellular pH of the *M. succiniciproducens* PALK, PALKcgmdh, and PALKmsmdh^G11Q^ strains were determined using pHrodo^TM^ Green AM Intracellular pH Indicator kit (Cat. no. P35373, Invitrogen, Carlsbad, CA, USA). The cells were collected at late exponential phase using centrifugation at 5566×*g* and 4 °C. The cells were then washed with Live Cell Imaging Solution (Invitrogen) to remove the culture medium. Next, the cells were resuspended with the pHrodo^TM^ AM staining solution containing pH-sensitive fluorogenic probes, which exhibit increasing fluorescence with decrease in pH, and incubated at 37 °C for 30 min. The pH-sensitive fluorogenic probe was modified with acetoxymethyl ester enabling the probe to easily permeate the cell membrane and to be retained within the intracellular space^63^. Finally, the fluorescence intensity of the probe inside the cells was measured using the Spark^®^ multimode microplate reader (Tecan, Männedorf, Switzerland) at excitation/emission of 509/533 nm. A calibration curve, which shows the fluorescence intensity emitted by the fluorogenic probe inside the cells when the intracellular pH ranged from 4.5 to 7.5, was derived using the Intracellular pH Calibration Buffer kit (Cat. no. P35379, Invitrogen) and was used to quantify the intracellular pH of the *M. succiniciproducens* PALK, PALKcgmdh, and PALKmsmdh^G11Q^ strains.

### Measurement of intracellular OAA concentration

Before analyzing the intracellular OAA concentration, the intracellular metabolites were extracted from the *M. succiniciproducens* PALK strain using a modified version of the quenching/extraction protocol reported from previous study^64^. Briefly, cells were cultivated to the late exponential phase and 60 mL of culture broth was mixed with 180 mL of ice-cold quenching solution (60% v/v aqueous methanol). The mixture was immediately centrifuged at 5566×*g* and 0 °C for 10 min to remove supernatant. Next, the cell pellet was treated with liquid nitrogen and freeze-dried. Extraction of intracellular metabolites was performed by treating the dried cells with 0.8 mL of cold 1 M perchloric acid. Then, the treated cells were centrifuged at 15,871×*g* and 0 °C for 15 min to collect the supernatant. Cell debris was resuspended using deionized water and filtered using a preweighed 0.2-μm membrane filter (Whatman International Ltd., Kent, UK). The filtered membrane filter was fully dried in 70 °C oven and weighed. Then, gDCW of the sample analyzed was determined by subtracting the weight of membrane filter from the total weight. Finally, the supernatant containing intracellular metabolites was neutralized using 0.4 mL of cold 3 M KHCO_3_ and centrifuged at 15,871×*g* and 0 °C for 15 min to remove precipitate.

The amount of intracellular OAA in the supernatant was quantified using the OAA assay kit (Sigma-Aldrich, St. Louis, MO, USA). The probe in the reaction mixture that is originally colorless changes to an intensely colored and fluorescent product once exposed to pyruvate, which is converted from OAA (one of the metabolites in the supernatant) by an enzyme mix. The reaction mixture contained 2 μL of enzyme mix, 2 μL of developer, 2 μL of OAA probe, and 44 μL of supernatant, which was previously diluted (1:1) using assay buffer to adjust the fluorescence for correct calculation of OAA using curve fitting method. The assay buffer, enzyme mix, developer, and OAA probe were provided in the OAA assay kit. The fluorescence intensity of the probe was measured using the Spark^®^ multimode microplate reader at excitation/emission of 535/587 nm. As intracellular pyruvate already exists in the *M. succiniciproducens* PALK strain, the fluorescence intensity of the probe from a reaction mixture not containing enzyme mix (control) was also analyzed to determine background fluorescence. The background fluorescence was subtracted from the fluorescence intensity of the probe from a reaction mixture containing enzyme mix. A calibration curve showing the fluorescence intensity emitted by the probe when the amount of OAA ranged from 0 to 1.0 mol, was also generated and used to quantify the amount of intracellular OAA in the sample analyzed. Finally, the volume and dry mass of a single cell of *M. succiniciproducens* PALK strain were measured (described below) and were used to calculate the intracellular OAA concentration in a single cell.

### Measurement of single cell volume and dry mass

The single cell volume and dry mass of *M. succiniciproducens* PALK strain were analyzed using 3D quantitative phase imaging (QPI) and quantitative image analysis. The 3D QPI of live cells (cultured up to late exponential phase) were performed using a commercial holotomography HT-2H (Tomocube Inc., Daejeon, Republic of Korea), which is based on Mach–Zehnder interferometry equipped with a digital micromirror device (DMD). A coherent monochromatic laser (*λ* = 532 nm) was divided into two paths, a reference beam and a sample beam, using a 2×2 single-mode fiber coupler. A 3D refractive index (RI) tomogram was reconstructed from multiple 2D holographic images acquired from 49 illumination conditions, a normal incidence, and 48 azimuthally symmetric directions with a polar angle (64.5°). The DMD was used to control the angle of an illumination beam impinging onto the sample^65^. The diffracted beams from the sample were collected using a high numerical aperture (NA = 1.2) objective lens UPLSAP 60XW (Olympus, Tokyo, Japan). The off-axis hologram was recorded by a CMOS image sensor FL3-U3-13Y3MC (FLIR Systems, Wilsonville, Oregon, USA). The visualization of 3D RI maps was carried out using a TomoStudio^TM^ software (Tomocube Inc.) Detailed information on the principle of optical diffraction tomography and a reconstructed MATLAB code can be found elsewhere^66–69^. Each cell detected from 3D QPI was segmented using RI thresholds determined by Otsu’s method and marker-controlled watershed segmentation. The single cell volume of *M. succiniciproducens* PALK strain was calculated based on the physical size of individual voxels. The single cell dry mass of *M. succiniciproducens* PALK strain was calculated using 0.19 mL g^−1^ as refractive index increment^70,71^.

### Reporting summary

Further information on research design is available in the Nature Research Reporting Summary linked to this article.

## Supplementary information

Supplementary Information

Peer Review File

Reporting Summary

Description of Additional Supplementary Files

Supplementary Data 1

Supplementary Data 2

## Data Availability

Data supporting the findings of this work are available within the paper and its Supplementary Information file. A reporting summary for this Article is available as a Supplementary Information file. The datasets generated and analyzed during the current study are available from the corresponding author upon request. Information on the PDB accessions 6ITL and 6ITK can also be found at 10.2210/pdb6ITL/pdb and 10.2210/pdb6ITK/pdb, respectively. The source data underlying Fig. 3, Supplementary Figs. 10, 11, 14, 15, and 16, and Supplementary Table 10 are provided as a Source Data file.

## Source Data

Source Data
