# Supplementary material for: Enhanced succinic acid production by Mannheimia employing optimal malate dehydrogenase
Source: Nat Commun. 2020 Apr 23;11:1970. doi: 10.1038/s41467-020-15839-z (PMC7181634; doi:10.1038/s41467-020-15839-z)
Supplement: Supplementary file 1 — Supplementary Information [file 41467_2020_15839_MOESM1_ESM.pdf]

# Enhanced succinic acid production by *Mannheimia* employing optimal malate dehydrogenase

Ahn and Seo *et al.*

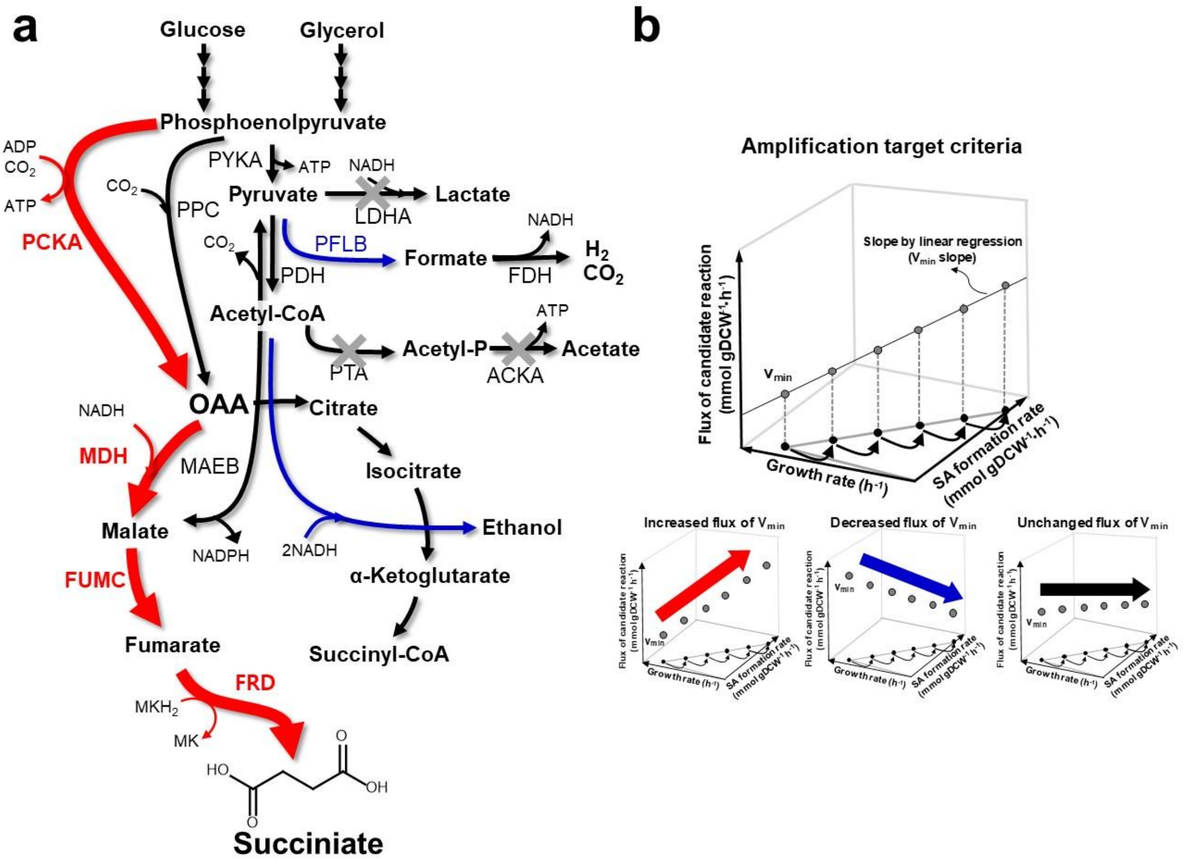


**Supplementary Figure 1. Selection of target enzymes based on *in silico* simulation.** (a) Correlations of metabolic reactions with SA in *M. succiniciproducens*. Red and blue arrows indicate reactions that positively and negatively relate with SA production rate, respectively. Black arrows indicate reaction fluxes that showed no specific correlations with SA production. Multiple arrows represent series of reactions. X mark represents deletion of gene. (b) Criteria of identifying target enzyme for enhanced SA production. To select the amplification candidates, the slope of minimal flux values ($V_{min}$ slope) was calculated using a linear regression between the enforced production rate of the target chemical and the $V_{min}$ values of the candidate reactions. The positively related reactions in $V_{min}$ slope (increased flux pattern of $V_{min}$) were selected as target enzymes. Abbreviations: Acetyl-P, acetyl-phosphate; ACKA, acetate kinase; FDH, formate dehydrogenase; FRD, fumarate reductase; FUMC, fumarate hydratase; LDHA, lactate dehydrogenase; MAEB, malate dehydrogenase (oxaloacetate-decarboxylating); MDH, malate dehydrogenase; MK, menaquinone; $MKH_2$, menaquinol; OAA, oxaloacetate; PCKA, phosphoenolpyruvate carboxykinase; PDH, pyruvate dehydrogenase; PFLB, pyruvate formate lyase; PPC, phosphoenolpyruvate carboxylase; PTA, phosphate acetyltransferase; PYKA, pyruvate kinase.

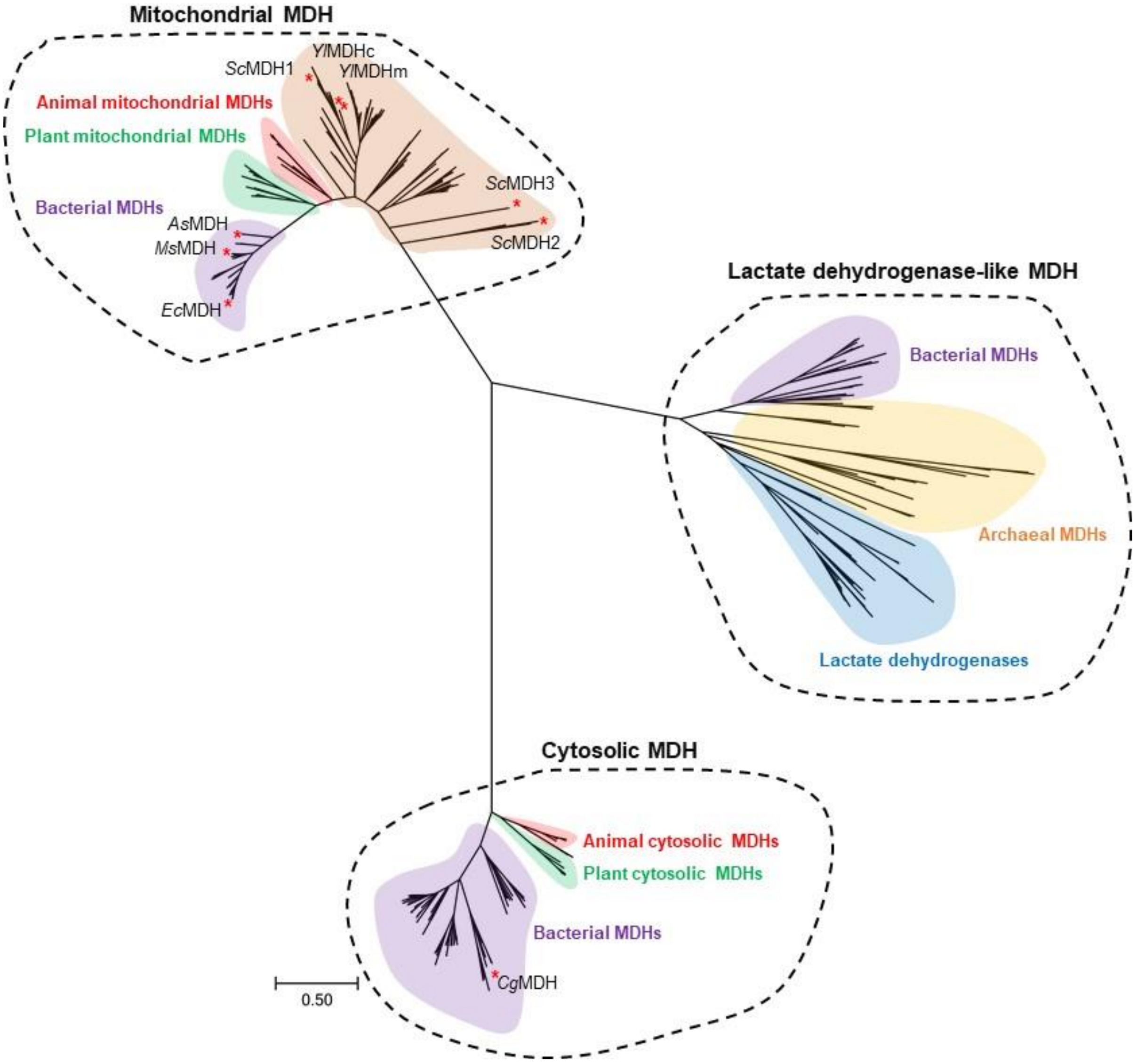


**Supplementary Figure 2. Phylogenetic tree of MDH.** MDHs from various organisms of all kingdoms are categorized accordingly. The MDHs used in this work are indicated by red asterisks.

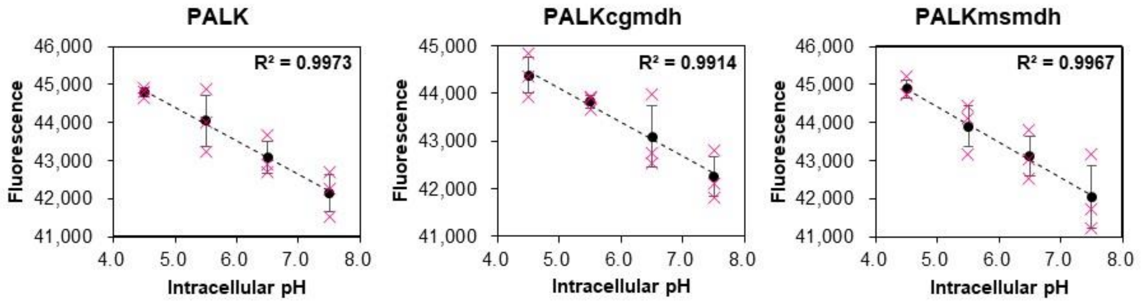


**Supplementary Figure 3. Standard curves of *M. succiniciproducens* PALK, PALKcgmdh, and PALKmsmdh$^{G11Q}$ strains.** Data are presented as mean values ± standard deviation. Standard deviations were generated from n=3 independent experiments.

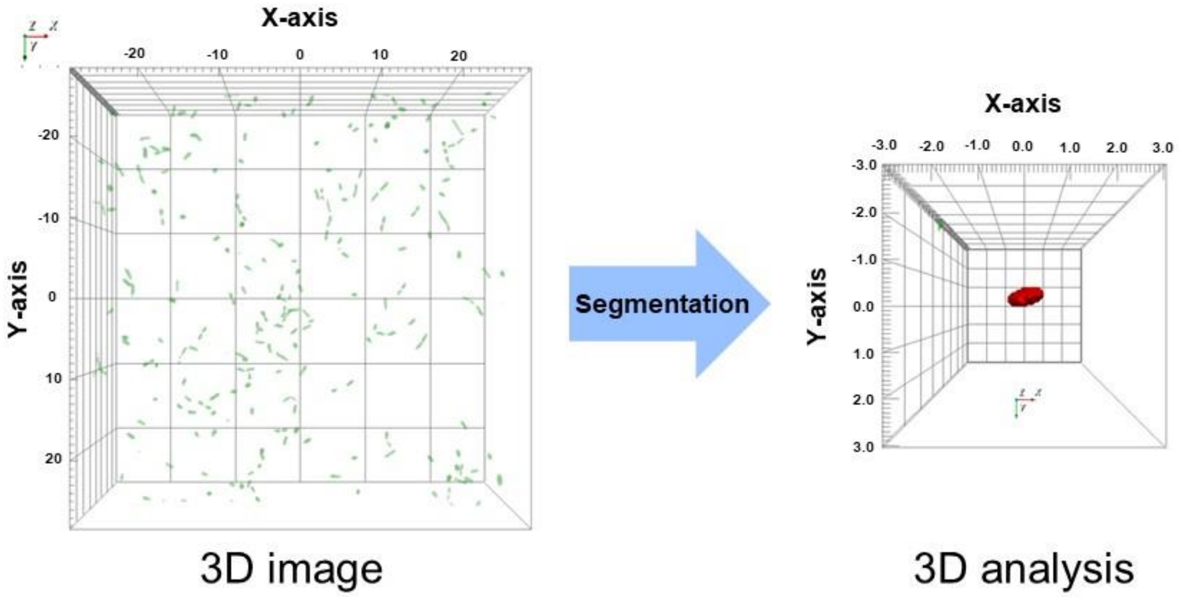


**Supplementary Figure 4. 3D QPI of live cells cultured up to late exponential phase and 3D image of segmented single cell.**

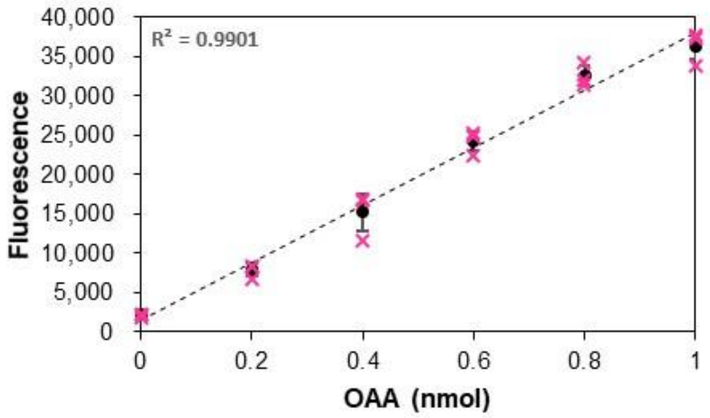


**Supplementary Figure 5. The OAA calibration curve utilized to quantify the intracellular OAA concentration in *M. succiniciproducens*.** Data are presented as mean values ± standard deviation. Standard deviations were generated from n=3 independent experiments.

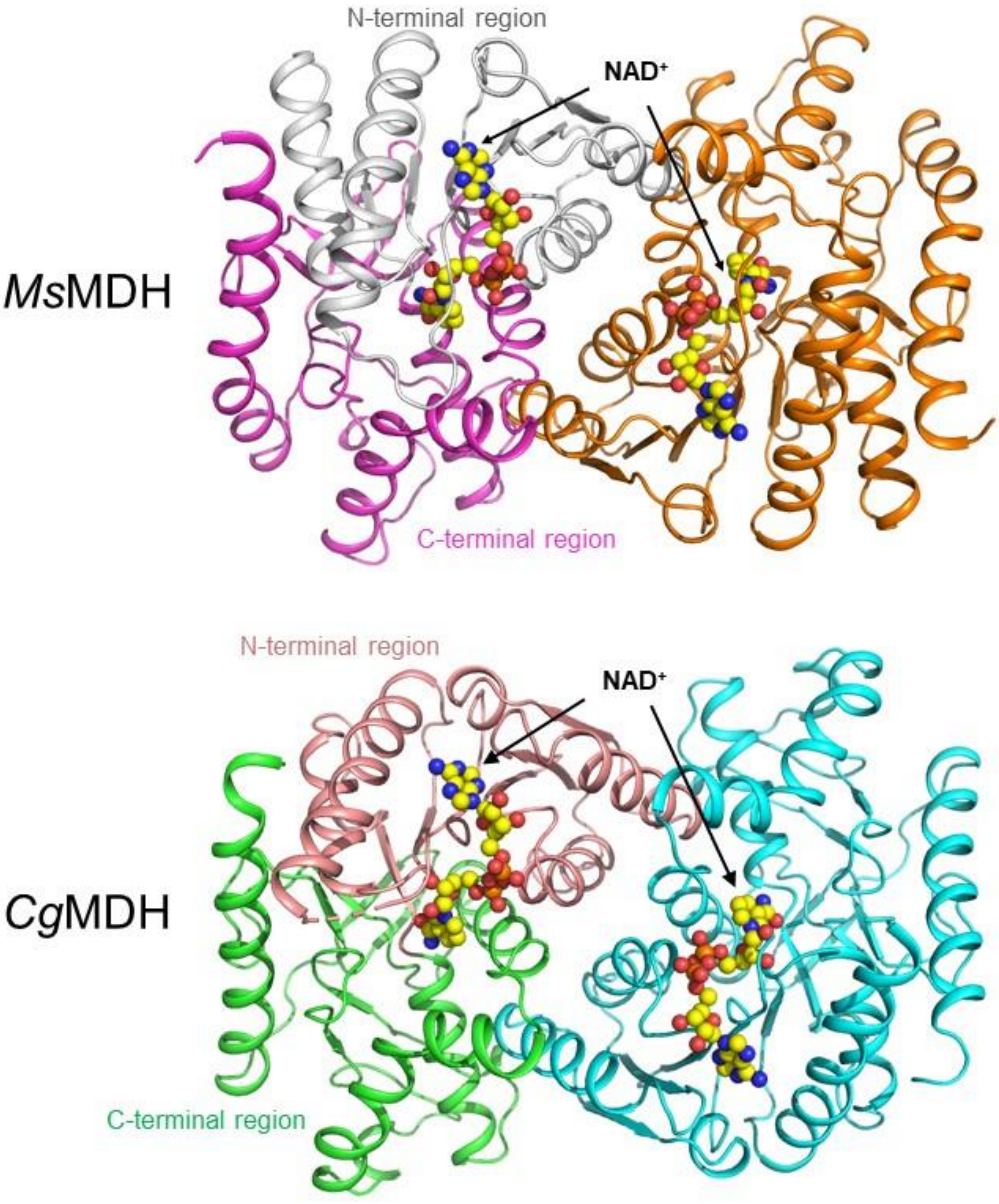


**Supplementary Figure 6. Overall structures of *Ms*MDH and *Cg*MDH.** (Top) Homodimer molecules of *Ms*MDH are distinguished by magenta and orange color. The distinct N-terminal region is distinguished from the C-terminal region by grey color. The bound $NAD^+$ molecule is shown as sphere model. The yellow, blue, and red spheres represent carbon, nitrogen, and hydrogen atoms, respectively, of the $NAD^+$ molecule. (Bottom) Homodimer molecules of *Cg*MDH are distinguished by green and cyan color. The distinct N-terminal region is distinguished from the C-terminal region by salmon color.

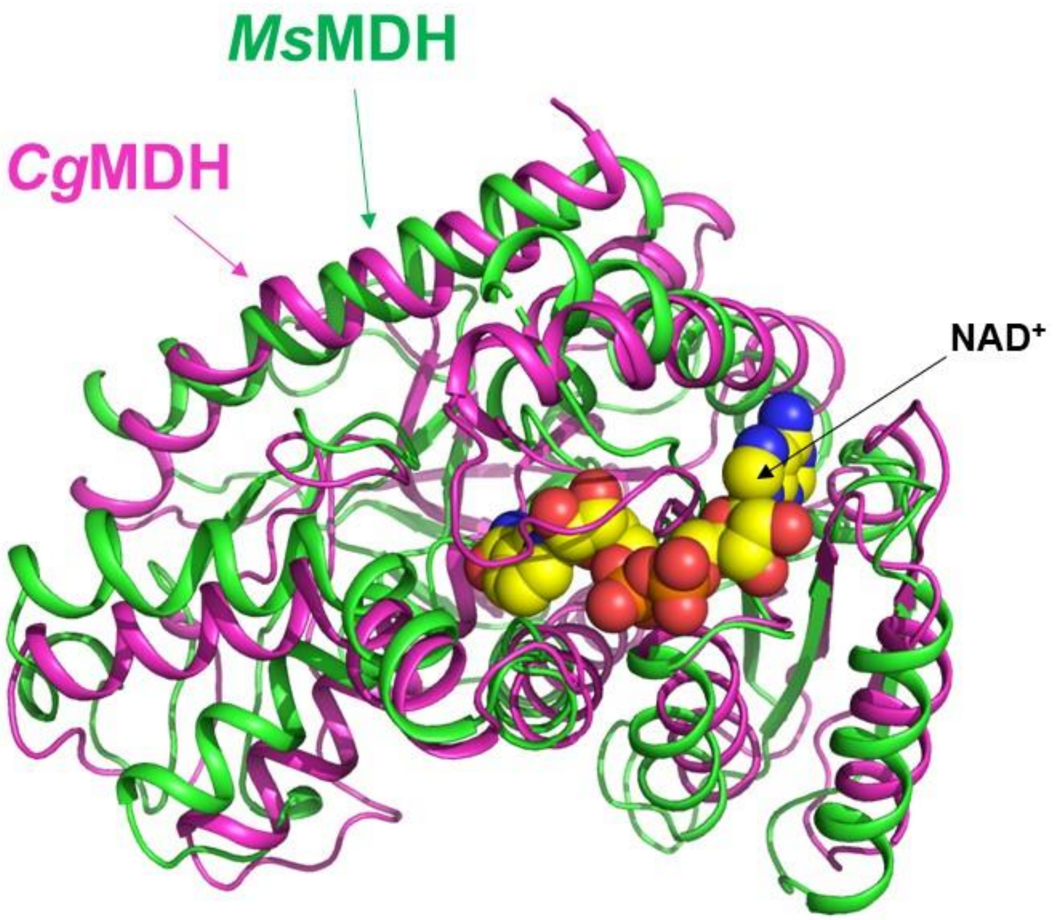


**Supplementary Figure 7. Superimposition of the active sites of *Ms*MDH and *Cg*MDH.** Crystal structure of *Ms*MDH and *Cg*MDH are shown as green and magenta colored model, respectively. The bound $NAD^+$ molecule is shown as sphere model. The yellow, blue, and red spheres represent carbon, nitrogen, and hydrogen atoms, respectively, of the $NAD^+$ molecule.

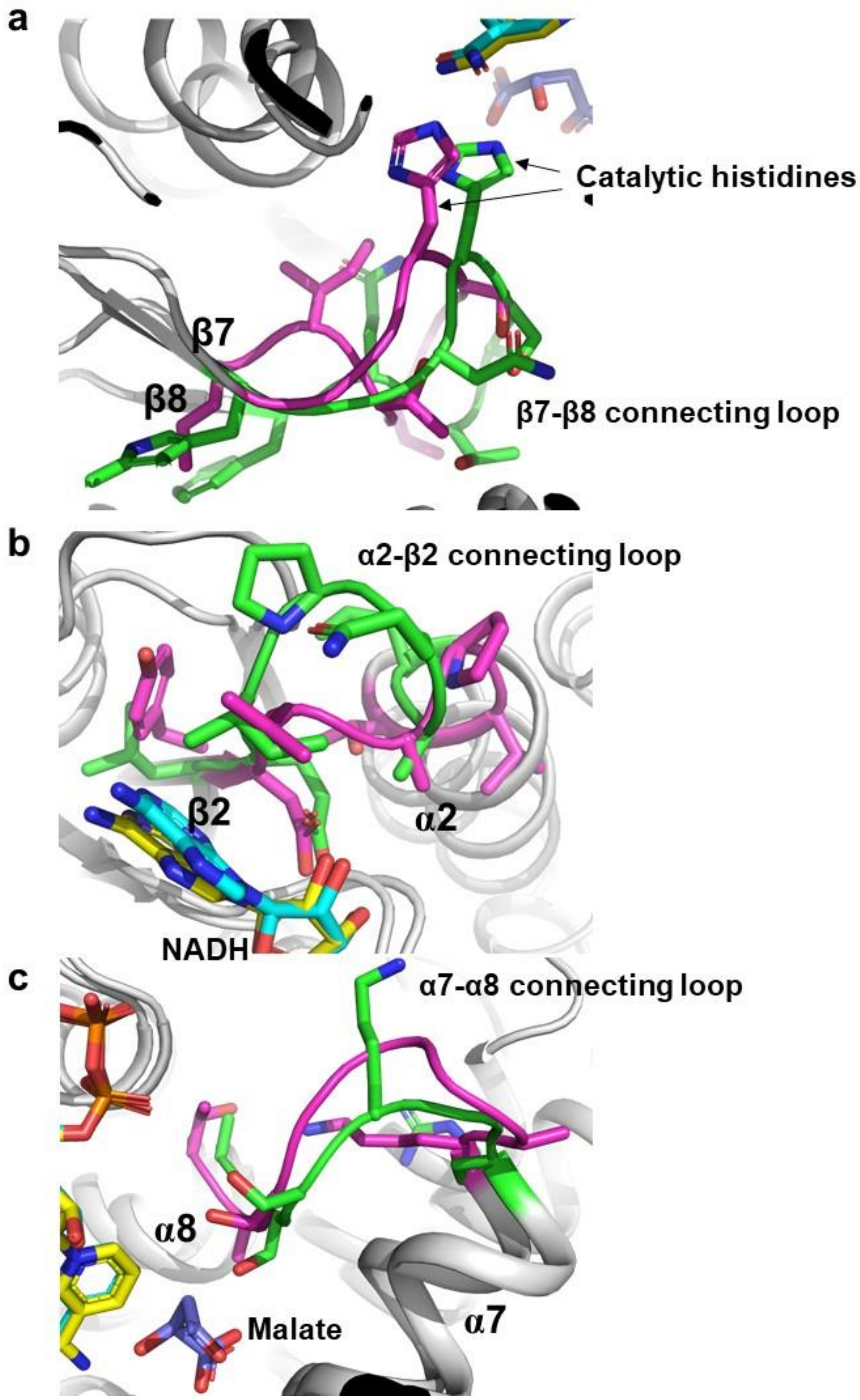


**Supplementary Figure 8. Structural comparison between *Ms*MDH and *Cg*MDH.** *Ms*MDH (magenta) and *Cg*MDH (green) structures are shown as cartoon and stick models (a) β7-β8 connecting loop (b) α2-β2 connecting loop (c) α7-α8 connecting loop.

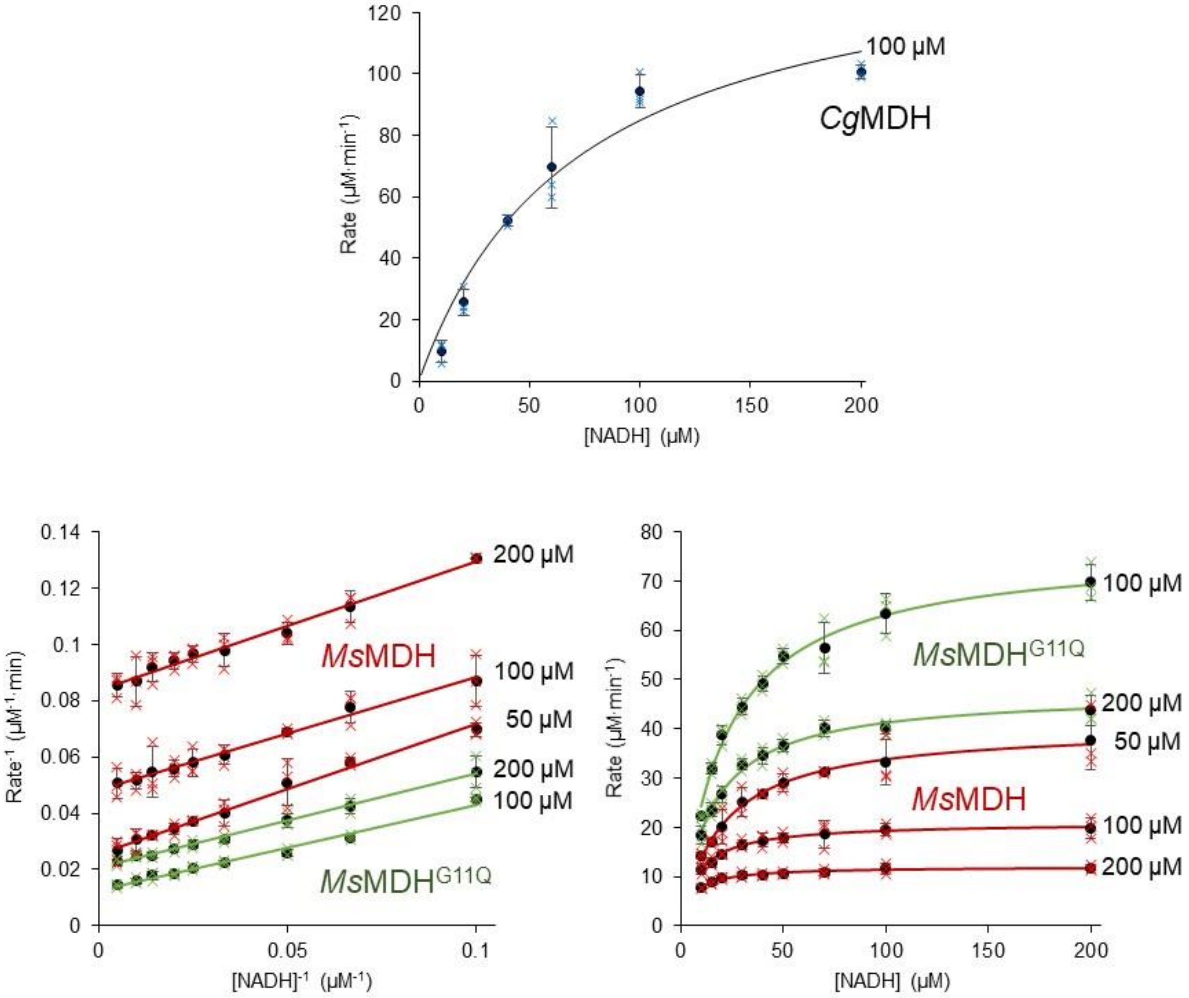


**Supplementary Figure 9. Michaelis-Menten and Lineweaver plots of *Ms*MDH, *Ms*MDH$^{G11Q}$, and *Cg*MDH for NADH**. The rates of *Cg*MDH for NADH were determined at 100 µM OAA. The rates of *Ms*MDH were determined at 50, 100, and 200 µM OAA while the rates of *Ms*MDH$^{G11Q}$ were determined at 100 and 200 µM OAA. Data are presented as mean values ± standard deviation. Standard deviations were generated from n=3 independent experiments.

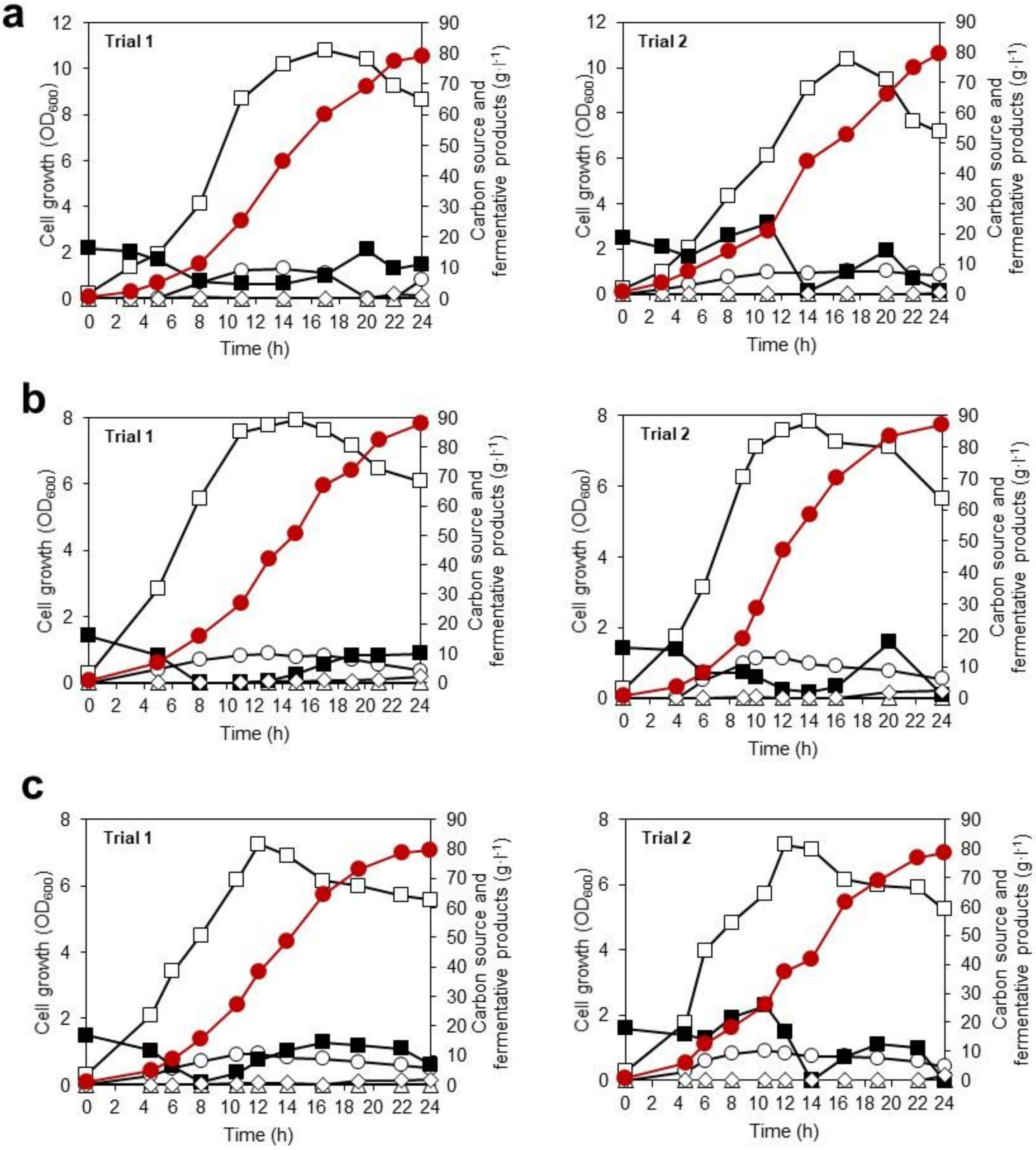
a
b
c
Trial 1
Trial 2
Cell growth (OD$_{600}$)
Carbon source and fermentative products (g·l$^{-1}$)
Time (h)

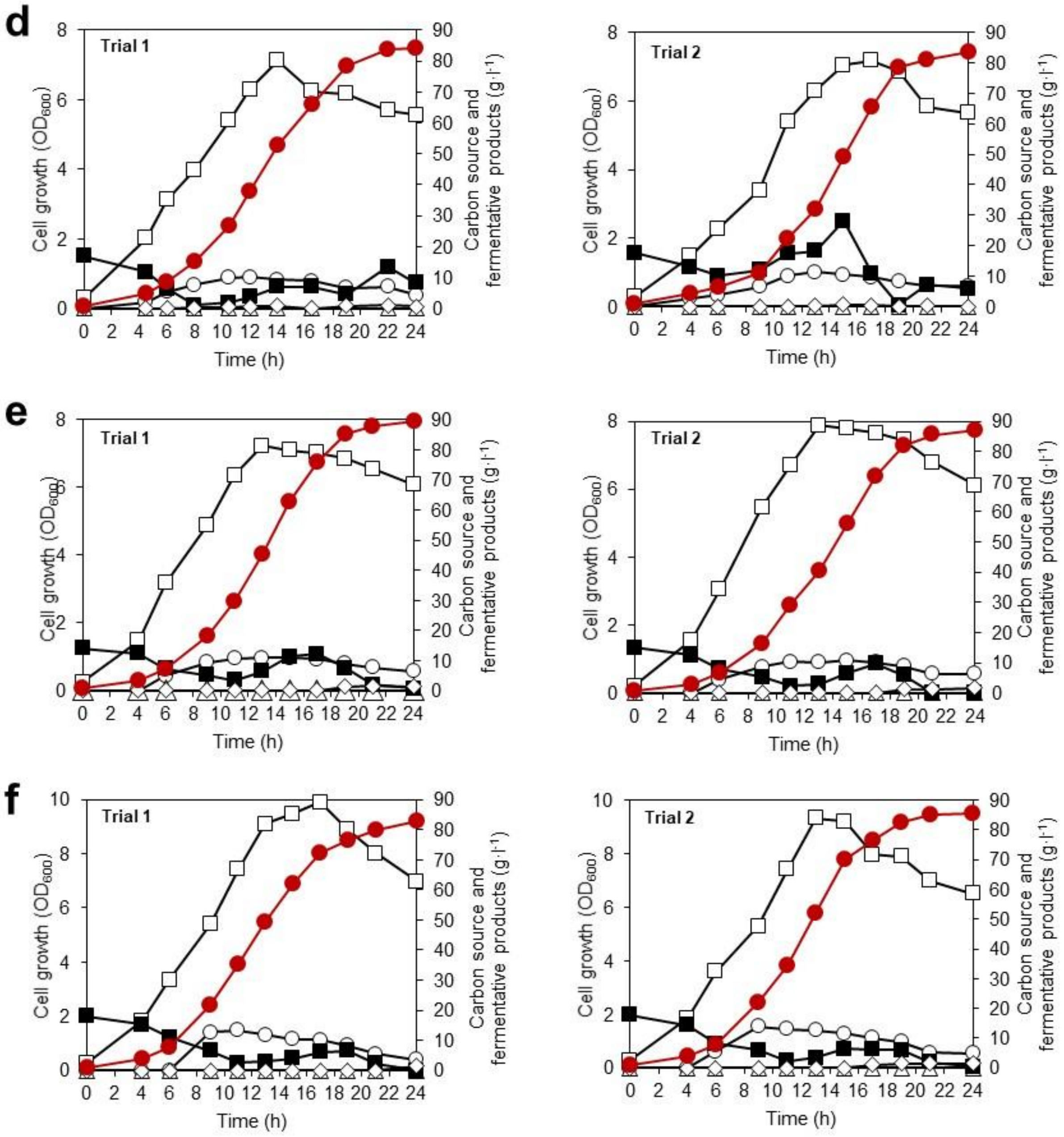


**Supplementary Figure 10. Fed-batch fermentation profiles (in duplicate) of the *M. succiniciproducens* (a) PALK (pMS3-msmdh), (b) PALK (pMS3-cgmdh), (c) PALK (pMS3-cgmdh$^{Q20G}$), (d) PALK (pMS3-msmdh$^{G11Q}$), (e) PALKcgmdh, and (f) PALKmsmdh$^{G11Q}$ strains in CDM using glucose as a carbon source (continued)**. Symbols: White square, cell growth; red circle, SA; black square, glucose; white circle, pyruvate; white diamond, acetate; white triangle, formate. Fermentations were all performed in duplicate (n=2 independent experiments) to confirm reproducibility. Source data are provided as a Source Data file.

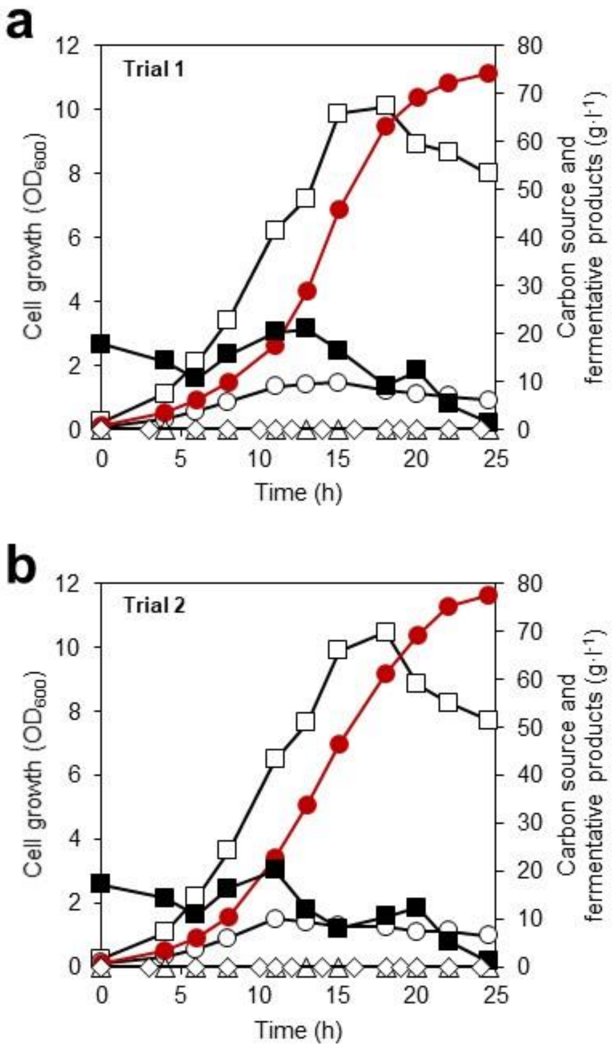


**Supplementary Figure 11. Fed-batch fermentation profiles of the *M. succiniciproducens* PALKPfrdmsmdh strain in CDM using glucose as carbon source.** Fermentations were performed in duplicate to demonstrate reproducibility. Symbols: White square, cell growth; red circle, SA; black square, glucose; white circle, pyruvate; white diamond, acetate; white triangle, formate. Fermentations were all performed in duplicate (n=2 independent experiments) to confirm reproducibility. Source data are provided as a Source Data file.

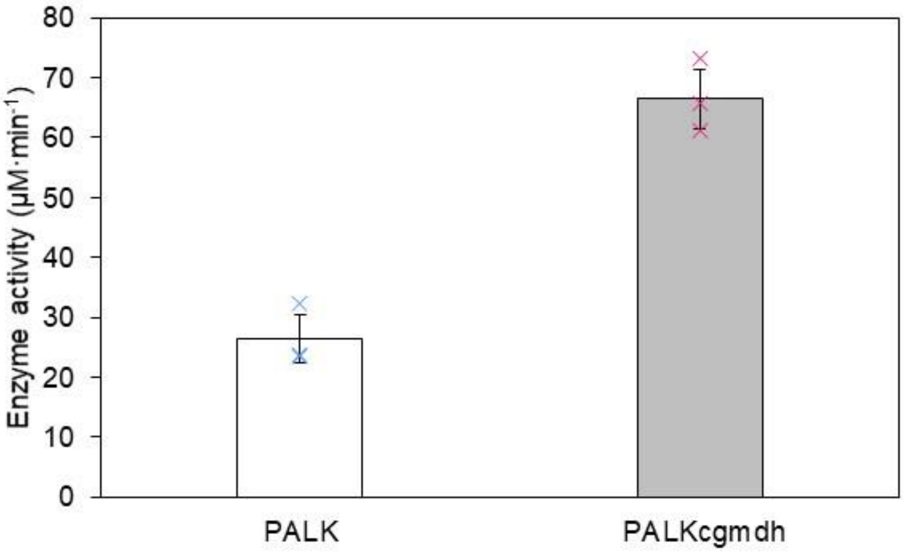


**Supplementary Figure 12. Enzyme activities in the cell extracts of *M. succiniciproducens* PALK and PALKcgmdh strains.** Enzyme activity of cell extract was determined using reaction mixture containing 200 μM NADH and 100 μM OAA. The concentrations of the total proteins in the reaction mixtures containing the PALK and PALKcgmdh cell lysates were 8.8 and 7.6 μg·mL$^{-1}$, respectively. Data are presented as mean values ± standard deviation. Standard deviations were generated from n=3 independent experiments.

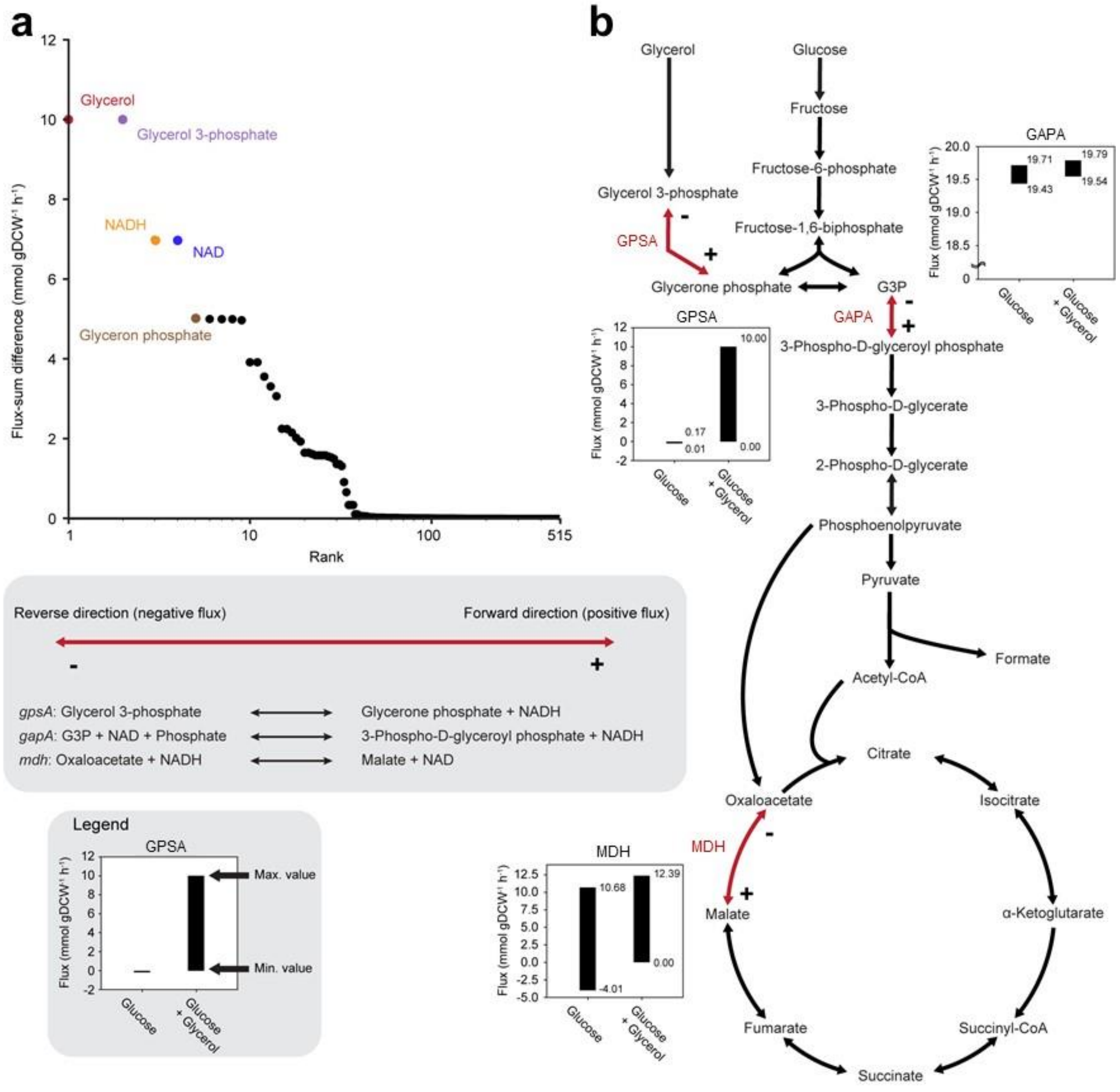


**Supplementary Figure 13. Difference in metabolic fluxes in the PALK strain generated from single (glucose) or dual (glucose and glycerol) carbon source utilization.** (a) *In silico* flux-sum differences from single and dual carbon source utilization. Colored metabolites indicate top five metabolites showing the highest flux-sum difference. The turnover rate of NADH was increased when dual carbon sources were utilized. (b) *In silico* flux variability of reactions in the SA production pathway when utilizing single or dual carbon source. Red arrows indicate reactions using $NAD^+$/NADH as cofactors. Negative flux indicates a reaction flowing in reverse direction. The metabolic fluxes of three $NAD^+$/NADH -utilizing reactions were increased toward positive direction when dual carbon sources were used. Abbreviations: G3P, glyceraldehyde 3-phosphate; GPSA, glycerol 3-phosphate dehydrogenase; GAPA, glyceraldehyde 3-phosphate dehydrogenase; MDH, malate dehydrogenase.

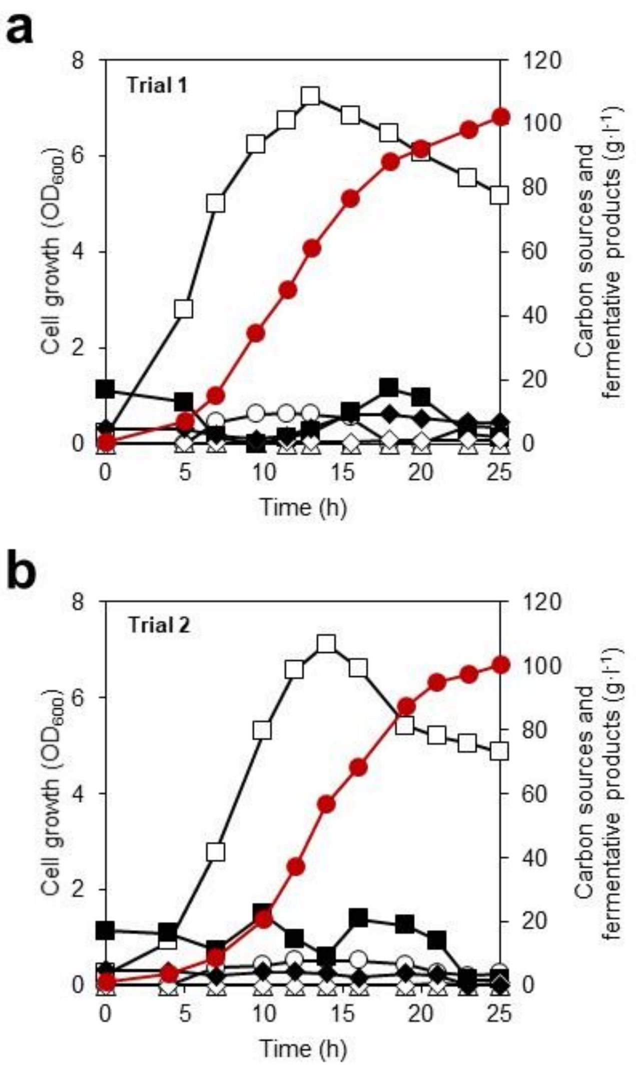


**Supplementary Figure 14. Fed-batch fermentation profiles of the *M. succiniciproducens* PALK (pMS3-cgmdh) strain in CDM using glucose and glycerol as dual carbon sources.** Fermentations were performed in duplicate to demonstrate reproducibility. Symbols: White square, cell growth; red circle, SA; black square, glucose; black diamond, glycerol; white circle, pyruvate; white diamond, acetate; white triangle, formate. Fermentations were all performed in duplicate (n=2 independent experiments) to confirm reproducibility. Source data are provided as a Source Data file.

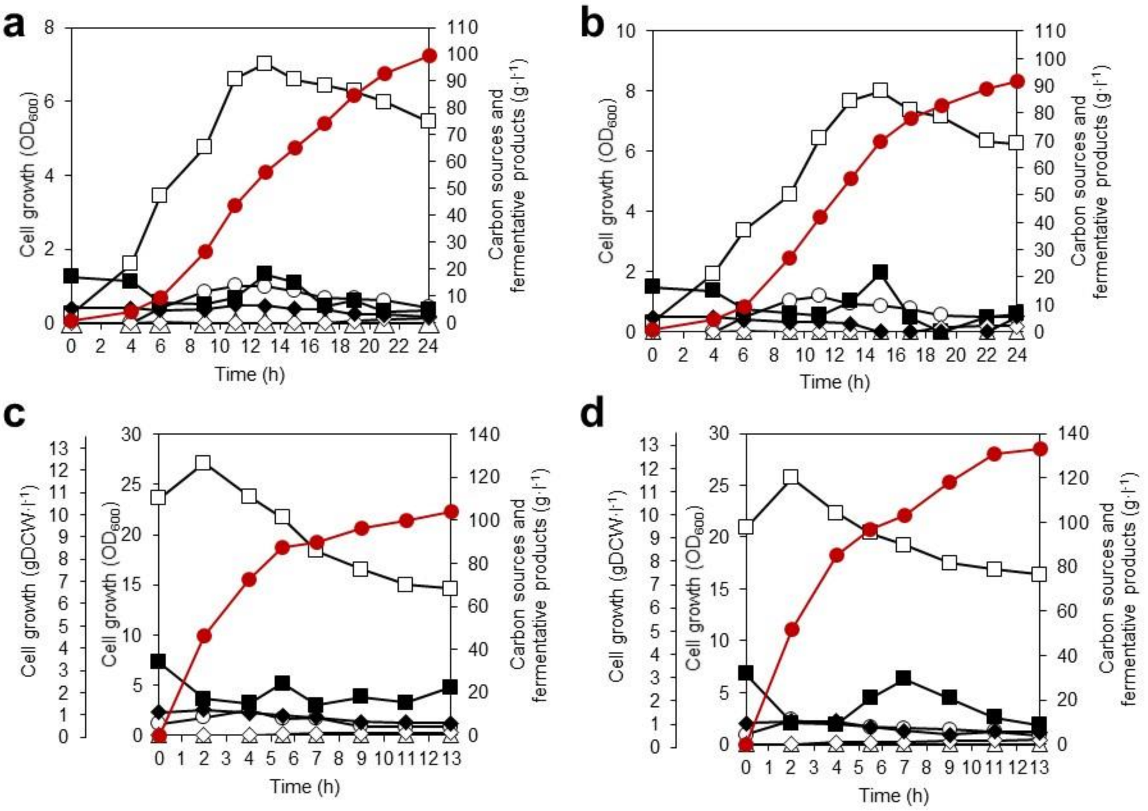


**Supplementary Figure 15. Fed-batch fermentation profiles of the *M. succiniciproducens* PALK, PALKcgmdh, and PALKmsmdh$^{G11Q}$ strains to demonstrate reproducibility.** Fed-batch fermentations of the (a) PALKcgmdh and (b) PALKmsmdh$^{G11Q}$ strains were carried out in CDM using glucose and glycerol as dual carbon sources. Fed-batch fermentations of the (c) PALK and (d) PALKcgmdh strains were carried out using glucose and glycerol with increased initial cell mass ($OD_{600}$ = 23.6 and 20.9, respectively). Symbols: White square, cell growth; red circle, SA; black square, glucose; black diamond, glycerol; white circle, pyruvate; white diamond, acetate; white triangle, formate. Fermentations were all performed in duplicate (n=2 independent experiments) to confirm reproducibility. Source data are provided as a Source Data file.

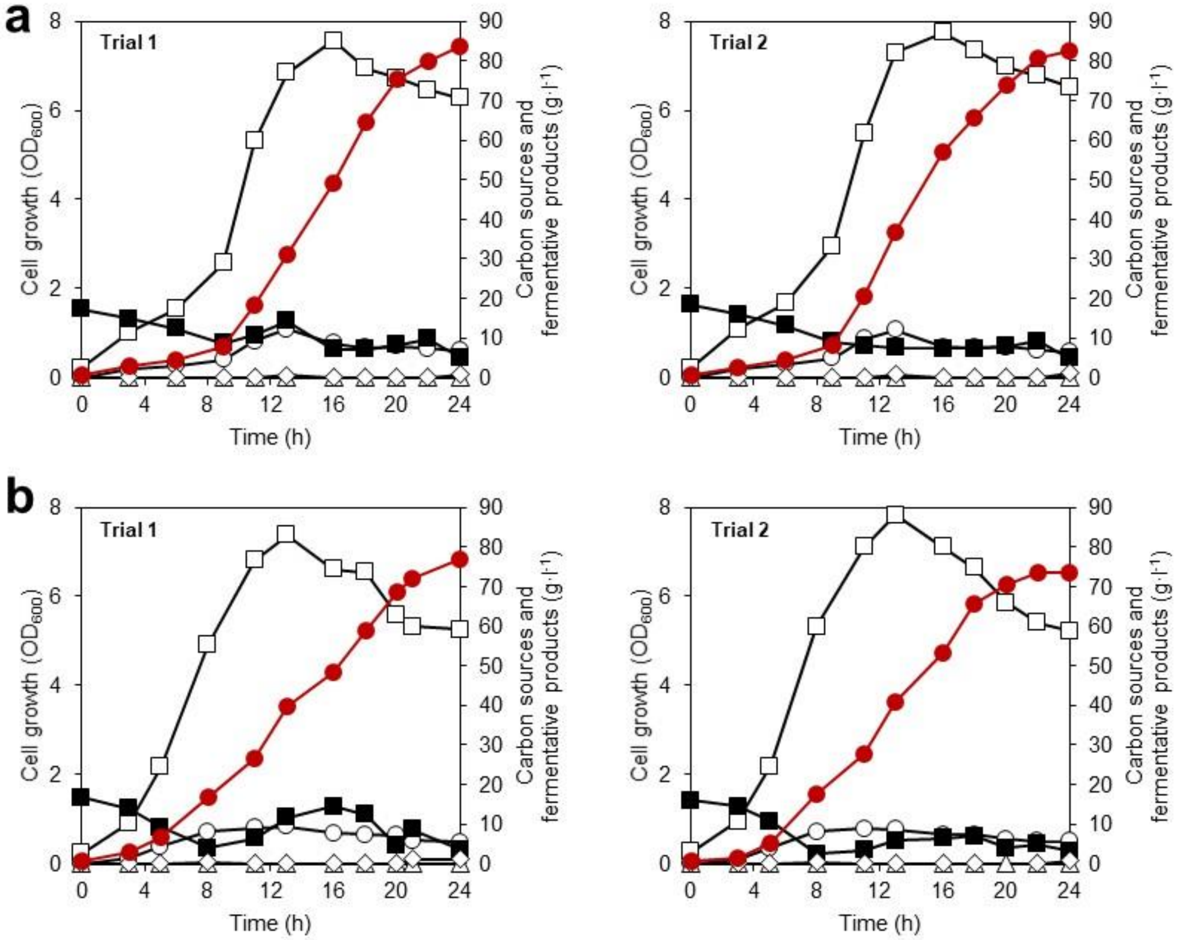


**Supplementary Figure 16. Fed-batch fermentation profiles (in duplicate) of the *M. succiniciproducens* (a) PALK (pMS3-atmdhc1) and (b) PALK (pMS3-atmdhm1) strains in CDM using glucose as carbon source.** Fermentations were performed in duplicate to demonstrate reproducibility. Symbols: White square, cell growth; red circle, SA; black square, glucose; white circle, pyruvate; white diamond, acetate; white triangle, formate. Fermentations were all performed in duplicate (n=2 independent experiments) to confirm reproducibility. Source data are provided as a Source Data file.

**Supplementary Table 1. Amplification target reactions identified by the FVSEOF algorithm.**

| Rank | Enzyme | Reaction[a] | $V_{min}$ slope |
|---|---|---|---|
| **1** | Fumarate reductase | $MKH_2$ + FUM <-> SUCC + MK + $H_{xt}$ | 0.986 |
| **2** | Phosphoenolpyruvate carboxykinase | $CO_2$ + PEP + ADP -> ATP + OAA | 0.629 |
| **3** | Malate dehydrogenase | NADH + OAA <-> MAL + NAD | 0.444 |
| **4** | Fumarate hydratase | MAL <-> FUM | 0.187 |

[a] Abbreviations: FUM, fumarate; MAL, malate; MK, menaquinone; $MKH_2$, menaquinol; OAA, oxaloacetate; PEP, phosphoenolpyruvate; SUCC, SA.

**Supplementary Table 2. Intracellular pH of various *M. succiniciproducens* strains.**

| Strain | Fluorescence | Intracellular pH[a] |
|---|---|---|
| PALK | 42,698±177.99 | 6.86±0.21 |
| PALKcgmdh | 42,930.67±119.15 | 6.63±0.17 |
| PALKmsmdh$^{G11Q}$ | 42,796.67±205.27 | 6.74±0.22 |

[a] Intracellular pH of *M. succiniciproducens* PALK, PALKcgmdh, and PALKmsmdh$^{G11Q}$ strains were determined by fitting the fluorescence to the corresponding standard curves shown in Supplementary Fig. 3. Data are presented as mean values ± standard deviation. Standard deviations were generated from n=3 independent experiments.

**Supplementary Table 3. Summary of the quantitative image analyses of ten segmented single cells detected from 3D QPI.**

| Single cell | 1 | 2 | 3 | 4 | 5 | 6 | 7 | 8 | 9 | 10 | Avg. | Std. dev.[a] |
|---|---|---|---|---|---|---|---|---|---|---|---|---|
| **Dry mass (pg)** | 0.08 | 0.09 | 0.07 | 0.06 | 0.07 | 0.1 | 0.09 | 0.1 | 0.06 | 0.06 | 0.08 | 0.02 |
| **Volume ($\mu m^3$)** | 0.78 | 0.84 | 0.77 | 0.58 | 0.71 | 1 | 0.88 | 0.97 | 0.65 | 0.54 | 0.77 | 0.15 |
| **Surface area ($\mu m^2$)** | 4.45 | 4.81 | 4.44 | 3.28 | 4.06 | 5.42 | 4.74 | 5.23 | 3.80 | 3.24 | 4.35 | 0.71 |
| **Projected area ($\mu m^2$)** | 0.82 | 0.80 | 0.81 | 0.63 | 0.70 | 0.97 | 0.84 | 0.93 | 0.65 | 0.56 | 0.77 | 0.13 |
| **Mean RI** | 1.35 | 1.35 | 1.35 | 1.35 | 1.35 | 1.35 | 1.35 | 1.35 | 1.35 | 1.35 | 1.35 | 0.00 |
| **Sphericity** | 0.92 | 0.89 | 0.91 | 1.03 | 0.95 | 0.89 | 0.94 | 0.91 | 0.95 | 0.99 | 0.94 | 0.04 |
| **Threshold RI** | 1.34 | 1.34 | 1.34 | 1.34 | 1.34 | 1.34 | 1.34 | 1.34 | 1.34 | 1.34 | 1.34 | 0.00 |
| **Dry mass/Volume ($gDCW \cdot L^{-1}$)** | 107.9 | 101.9 | 97.1 | 97.7 | 100.7 | 99.3 | 101.2 | 107.1 | 99.3 | 103.2 | 101.54 | 3.46 |

[a] Standard deviations were generated from n=10 independent experiments.

**Supplementary Table 4. Dried cell weight in each sample of the PALK and PALKcgmdh strains analyzed.**

| PALK strain | 1 | 2 | 3 | 4 | Avg. | Std. dev.[d] |
|---|---|---|---|---|---|---|
| Membrane filter (g)[a] | 0.13 | 0.13 | 0.13 | 0.13 | 0.13 | 0.0004 |
| Dried filter + dried cell (g)[b] | 0.21 | 0.21 | 0.21 | 0.21 | 0.21 | 0.0039 |
| Dried cell weight (g)[c] | 0.08 | 0.08 | 0.07 | 0.07 | 0.075 | 0.004 |
| **PALKcgmdh strain** | **1** | **2** | **3** | **4** | **Avg.** | **Std. dev.** |
| Membrane filter (g)[a] | 0.13 | 0.13 | 0.13 | 0.13 | 0.13 | 0.00 |
| Dried filter + dried cell (g)[b] | 0.21 | 0.21 | 0.21 | 0.21 | 0.21 | 0.004 |
| Dried cell weight (g)[c] | 0.08 | 0.08 | 0.07 | 0.07 | 0.078 | 0.005 |

[a] Weight of the membrane filter.

[b] Weight of the membrane filter and dried cell from sample.

[c] Weight of the dried cell from sample.

[d] Standard deviations were generated from n=4 independent experiments.

**Supplementary Table 5. Quantification of intracellular OAA concentration in a single cell of PALK and PALKcgmdh strains (4 samples).**

| | Fluorescence | | | | | |
|---|---|---|---|---|---|---|
| **PALK strain** | **1** | **2** | **3** | **4** | **Avg.** | **Std. dev.**[g] |
| **Sample**[*, a] | 26646.5 | 28514 | 29696 | 29766 | 28655.6 | 1262.1 |
| **Sample (no enzyme)**[*, b] | 11013 | 10950.5 | 10750.5 | 17120.5 | 12458.6 | 2693.2 |
| **Difference**[*, c] | 15633.5 | 17563.5 | 18945.5 | 12645.5 | 16197 | 2363.9 |
| **OAA (nmol·sample$^{-1}$)**[d] | 18.58 | 21.13 | 22.96 | 14.63 | 19.33 | 3.13 |
| **OAA (nmol·gDCW$^{-1}$)**[e] | 234.01 | 278.41 | 316.22 | 199.89 | 257.13 | 44.03 |
| **OAA concentration (μM·cell$^{-1}$)**[f] | 23.76 | 28.27 | 32.11 | 20.3 | 26.11 | 4.47 |
| **PALKcgmdh strain** | **1** | **2** | **3** | **4** | **Avg.** | **Std. dev.** |
| **Sample**[*, a] | 26414.5 | 25188 | 25307.5 | 25167.5 | 25519.38 | 519.56 |
| **Sample (no enzyme)**[*, b] | 14558 | 18548 | 19431.5 | 15243.5 | 16945.25 | 2082.38 |
| **Difference**[*, c] | 11856.5 | 6640 | 5876 | 9924 | 8574.13 | 2429.86 |
| **OAA (nmol·sample$^{-1}$)**[d] | 13.6 | 6.7 | 5.69 | 11.04 | 9.26 | 3.21 |
| **OAA (nmol·gDCW$^{-1}$)**[e] | 169.9 | 97.75 | 75.81 | 137.77 | 120.3 | 36.23 |
| **OAA concentration (μM·cell$^{-1}$)**[f] | 17.25 | 9.93 | 7.7 | 13.99 | 12.22 | 3.68 |

[*] Before measurement of fluorescence, the sample (dissolved in 800 μl perchloric acid and 400 μl $KHCO_3$) was diluted (1:1) using assay buffer to adjust the fluorescence for correct calculation of OAA amount using curve fitting method.
[a] Fluorescence intensity from a reaction mixture (50 μl) with enzyme mix. The fluorescence originated from intracellular pyruvate, which is originally present in the sample (background fluorescence), and the pyruvate produced by enzyme mix using intracellular OAA.
[b] Fluorescence intensity from a reaction mixture (50 μl) without enzyme mix (background fluorescence).
[c] Fluorescence intensity from pyruvate converted from intracellular OAA by the enzyme mix (a-b).
[d] Amount of OAA in the sample calculated using intracellular OAA calibration curve and by applying dilution factor.
[e] Amount of OAA in the sample (d) divided by gDCW of the sample (Supplementary Table 4).
[f] Intracellular concentration of OAA in a single cell of *M. succiniciproducens* calculated by multiplying dry mass/volume of single cell (101.54 gDCW·L$^{-1}$) determined in

Supplementary Table 3.
[g] Standard deviations were generated from 4 replicates.

**Supplementary Table 6. Data collection and refinement statistics of *Ms*MDH and *Cg*MDH.**

| | *Ms*MDH | *Cg*MDH |
|---|---|---|
| PDB code | 6ITL | 6ITK |
| Data collection | | |
| Wavelength (Å) | 0.97934 | 0.97934 |
| Unit cell (*a*, *b*, *c*; *α*, *β*, *γ*) (Å; °) | 80.09, 80.09, 193.15; 90.0, 90.0, 120.0 | 102.93, 116.94, 66.00; 90.0, 95.3, 90.0 |
| Space group | P6422 | C2 |
| Solvent content (%) | 52.04 | 56.77 |
| Protein chains in AU | 1 | 2 |
| Resolution range (Å) | 69.36-1.98 | 77.08-2.00 |
| Highest resolution shell (Å) | 2.01-1.98 | 2.03-2.00 |
| Unique reflections | 26683 | 50050 |
| Redundancy | 11.7 (9.4) | 2.7 (2.2) |
| Completeness (%) | 99.7 (99.8) | 96.3 (93.0) |
| $R_{merge}$ (%) | 7.2 (28.3) | 12.3 (39.2) |
| Average I/s (I) | 54.6 (11.0) | 18.0 (3.5) |
| Refinement | | |
| R (%) | 16.9 | 19.0 |
| $R_{free}$ (%) | 21.4 | 22.9 |
| Mean B value (Å$^2$)* | 27.5 | 24.4 |
| RMS deviation bond lengths (Å) | 0.021 | 0.010 |
| RMS deviation bond angles (°) | 2.166 | 1.624 |
| Number of amino acid residues | 312 | 636 |
| Number of water molecules | 138 | 228 |
| Ramachandran plot | | |
| Most favored regions (%) | 96.5 | 98.7 |
| Additional allowed Regions (%) | 3.2 | 1.3 |

*Mean B value is for both protein atoms and the solvent molecules.

**Supplementary Table 7. Kinetic parameters of *Cg*MDH, *Ms*MDH, and *Ms*MDH$^{G11Q}$ for NADH.**

| MDH | OAA (μM) | *kcat* ($s^{-1}$)* | *km* (μM)* |
|---|---|---|---|
| *Cg*MDH | 100 μM | 810.3 ±108.6 | 72.0 ±21.7 |
| *Ms*MDH | 50 μM | 225.4 ±3.5 | 19.8 ±1.0 |
| | 100 μM | 116.4 ±0.6 | 8.6 ±0.3 |
| | 200 μM | 66.5 ±0.7 | 5.4 ±0.4 |
| *Ms*MDH$^{G11Q}$ | 100 μM | 426.4 ±8.4 | 21.7 ±1.3 |
| | 200 μM | 263.0 ±3.8 | 14.9 ±0.8 |

* Data are presented as mean values ± standard deviation. Standard deviations were generated from n=3 independent experiments.

## Supplementary Table 8. Strains and plasmids used in this study.

| Strain or plasmid | Description$^a$ | Source |
|---|---|---|
| ***M. succiniciproducens* PALK** | *M. succiniciproducens* (*ldhA*::Km$^r$ *pta-ackA*::Sp$^r$) | [1] |
| ***M. succiniciproducens* PALK (pMS3)** | PALK harboring pMS3 | [2] |
| ***M. succiniciproducens* PALK (pMS3-msmdh)** | PALK harboring pMS3-msmdh | This study |
| ***M. succiniciproducens* PALK (pMS3-cgmdh)** | PALK harboring pMS3-cgmdh | This study |
| **PALK (pMS3-msmdh$^{G11Q}$)** | PALK harboring pMS3-msmdh$^{G11Q}$ | This study |
| **PALK (pMS3-cgmdh$^{Q20G}$)** | PALK harboring pMS3-cgmdh$^{Q20G}$ | This study |
| **PALKmsmdh$^{G11Q}$** | PALK *mdh*$^-$::P$_{frd}$-*msmdh*$^{G11Q}$ | This study |
| **PALKcgmdh** | PALK *mdh*$^-$::P$_{frd}$-*cgmdh* | This study |
| **PALKPfrdmsmdh** | PALK P$_{mdh}^-$::P$_{frd}$ | This study |
| ***E. coli* TOP10** | Str$^r$, cloning host | Lab stock |
| ***E. coli* BL21(DE3)-T1$^R$** | F$^-$ *omp*T, *hsdS$_B$* ($r_B^-m_B^-$) *gal dcm* λ (DE3) *tonA*, Expression host | Lab stock |
| ***E. coli* W3110** | K12 F$^-$ (*rmD-rmE*) | Lab stock |
| ***E. coli* (p10099A-cgmdh)** | *E. coli* W3110 harboring p10099A-cgmdh | This study |
| ***C. glutamicum* ATCC13032** | Wild type *C. glutamicum* | Lab stock |
| ***C. glutamicum* (pEKEx1-cgmdh)** | *C. glutamicum* ATCC13032 harboring pEKEx1-cgmdh | This study |
| **pET30a** | Km$^r$, bacterial expression vector with T7 promoter (5.4 kb), restriction enzyme cloning | Novagen/Merck |
| **pET30a-msmdh** | pET30a derivative containing *msmdh* gene from *M. succiniciproducens* (6.3 kb) | This study |
| **pET30a-asmdh** | pET30a derivative containing *asmdh* genefrom *A. succinogenes* (6.3 kb) | This study |
| **pET30a-cgmdh** | pET30a derivative containing *cgmdh* gene from *C. glutamicum* (6.3 kb) | This study |
| **pET30a-ecmdh** | pET30a derivative containing *ecmdh* gene from *E. coli* (6.3 kb) | This study |
| **pET30a-ylmdh** | pET30a derivative containing *ylmdh* gene from *Y. lipolytica* (6.4 kb) | This study |
| **pET30a-scmdh1** | pET30a derivative containing *scmdh1* gene from *S. cerevisiae* (6.3 kb) | This study |
| **pET30a-scmdh2** | pET30a derivative containing *scmdh2* gene from *S. cerevisiae* (6.3 kb) | This study |
| **pET30a-scmdh3** | pET30a derivative containing *scmdh3* gene from *S. cerevisiae* (6.3 kb) | This study |
| **pET30a-msmdh$^{G11Q}$** | pET30a derivative containing mutant *msmdh* gene (*msmdh*$^{G11Q}$) (6.3 kb) | This study |
| **pET30a-msmdh$^{L101Q}$** | pET30a derivative containing mutant *msmdh* gene (*msmdh*$^{L101Q}$) (6.3 kb) | This study |
| **pET30a-msmdh$^{A224S}$** | pET30a derivative containing mutant *msmdh* gene (*msmdh*$^{A224S}$) (6.3 kb) | This study |
| **pET30a-cgmdh$^{Q20G}$** | pET30a derivative containing mutant *cgmdh* gene (*cgmdh*$^{Q20G}$) (6.3 kb) | This study |
| **pET30a-cgmdh$^{Q117L}$** | pET30a derivative containing mutant *cgmdh* gene (*cgmdh*$^{Q117L}$) (6.3 kb) | This study |
| **pET30a-cgmdh$^{S242A}$** | pET30a derivative containing mutant *cgmdh* gene (*cgmdh*$^{S242A}$) (6.3 kb) | This study |
| **pMS3** | Ap$^r$, *E. coli*-rumen bacteria shuttle vector containing *M. succiniciproducens frd* promoter (P$_{frd}$) (4.3 kb) | [3] |
| **pMS3-msmdh** | pMS3 derivative containing *msmdh* gene from *M. succiniciproducens* (5.2 kb) | This study |
| **pMS3-cgmdh** | pMS3 derivative containing *cgmdh* gene from *C. glutamicum* (5.2 kb) | This study |
| **pMS3-msmdh$^{G11Q}$** | pMS3 derivative containing mutant *msmdh* gene (*msmdh*$^{G11Q}$) (5.2 kb) | This study |
| **pMS3-cgmdh$^{Q20G}$** | pMS3 derivative containing mutant *cgmdh* gene (*cgmdh*$^{Q20G}$) (5.2 kb) | This study |
| **pMS3-atmdhc1** | pMS3 derivative containing *atmdhc1* gene from A. thaliana (5.3 kb) | This study |
| **pMS3-atmdhm1** | pMS3 derivative containing *atmdhc1* gene from A. thaliana (5.4 kb) | This study |
| **pMSmulox** | Km$^r$, Cm$^r$, Knockout template vector, *lox66-cat-lox77* (3.5 kb) | [3] |
| **pSacHR06** | Km$^r$, plasmid containing *sacB* gene (4.7 kb) | [3] |
| **pCRX5** | Ap$^r$, pMS3-ts derivative containing *cre* gene (5.3 kb) | [3] |
| **pINmsmdh$^{G11Q}$** | Cm$^r$, *sacB* gene, *msmdh*$^{G11Q}$ gene integration vector, pMSmulox derivative (8.0 kb) | This study |
| **pINcgmdh** | Cm$^r$, *sacB* gene, *cgmdh* gene integration vector, pMSmulox derivative (8.0 kb) | This study |
| **pINPfrdmsmdh** | Cm$^r$, *sacB* gene, P$_{frd}$ integration vector, pMSmulox derivative (7.6 kb) | This study |
| **p10099A** | Ap$^r$, pBBR322 origin, synthetic BBa23100 constitutive promoter (2.6 kb) | Lab stock |
| **p10099A-cgmdh** | p10099A derivative containing *cgmdh* gene (3.6 kb) | This study |
| **pEKEx1** | Km$^r$, *E. coli-C. glutamicum* shuttle vector containing *tac* promoter and *lacI*$^Q$ (8.0 kb) | Lab stock |
| **pEKEx1-cgmdh** | pEKEx1 derivative containing *cgmdh* gene (9.0 kb) | This study |

[a]Ap, ampicillin; Km, kanamycin; Cm, chloramphenicol; Sp, spectinomycin; Str, streptomycin; r, resistance.

## Supplementary Table 9. Primers used in this study.

| Primer: Description | Sequence (5' to 3') |
|---|---|
| P1: *msmdh* or *msmdh*$^{G11Q}$ gene fragments for pMS3-msmdh or pMS3-msmdh$^{G11Q}$ (F) | TATCAACTCTACTGGGGAGGAATTCATGAAAGTTGCAGTTCTAG |
| P2: *msmdh* or *msmdh*$^{G11Q}$ gene fragments for pMS3-msmdh or pMS3-msmdh$^{G11Q}$ (R) | TCTAGAGGATCCCCGGGTACCTTAACCGTTAATAAAATCTTCAC |
| P3: *cgmdh or cgmdh*$^{Q20G}$ gene fragments for pMS3-cgmdh or pMS3-cgmdh$^{Q20G}$ (F) | TATCAACTCTACTGGGGAGGATGAATTCCCCGCAGAAC |
| P4: *cgmdh or cgmdh*$^{Q20G}$ gene fragments for pMS3-cgmdh or pMS3-cgmdh$^{Q20G}$ (R) | GGATCCCCGGGTACCGAGCTTTAGAGCAAGTCGCGCAC |
| P5: *atmdhc1* gene fragment for pMS3-atmdhc1 (F) | TATCAACTCTACTGGGGAGGATGGCGAAGGAACCAGTTC |
| P6: *atmdhc1* gene fragment for pMS3-atmdhc1 (R) | TCTAGAGGATCCCCGGGTACTTAAGAGAGGCATGAGTAAGC |
| P7: *atmdhm1* gene fragment for pMS3-atmdhm1 (F) | TATCAACTCTACTGGGGAGGATGTTCAGATCTATGCTCG |
| P8: *atmdhm1* gene fragment for pMS3-atmdhm1 (R) | TCTAGAGGATCCCCGGGTACTCACTGGTTGGCAAACTTG |
| P9: Upstream of *msmdh* gene for pINmsmdh$^{G11Q}$ or pINcgmdh (F) | TTCAACGGGAAACGTCTTGCTCGAGCCTTATGTGGACCGAGAAGA |
| P10: Upstream of *msmdh* gene for pINmsmdh$^{G11Q}$ or pINcgmdh (R) | GGCATTAGCCAACAGAATAGCTGACCGAAAAAGTGGCGGA |
| P11: *msmdh*$^{G11Q}$ or *cgmdh* gene fragments for pINmsmdh$^{G11Q}$ or pINcgmdh (F) | CTATTCTGTTGGCTAATGCC |
| P12: *msmdh*$^{G11Q}$ gene fragment for pINmsmdh$^{G11Q}$ (R) | TGTAGCCGCGTTCTAACGACTACGAATAATACCCGCAT |
| P13: *cgmdh* gene fragment for pINcgmdh (R) | TGTAGCCGCGTTCTAACGTCGACTCTAGAGGATCCCCG |
| P14: *lox66-cat-lox77* cassette for pINmsmdh$^{G11Q}$ or pINcgmdh (F) | CGTTAGAACGCGGCTACA |
| P15: *lox66-cat-lox77* cassette for pINmsmdh$^{G11Q}$ or pINcgmdh (R) | ATAGGGAGACCGGCAGATC |
| P16: Downstream of *msmdh* gene for pINmsmdh$^{G11Q}$ or pINcgmdh (F) | GATCTGCCGGTCTCCCTATTTAAGACTCCTTAATGTGGA |
| P17: Downstream of *msmdh* gene for pINmsmdh$^{G11Q}$ or pINcgmdh (R) | GCCGCCACCGCGGTGGAGCTCGCGTTAGTTGTTGAGTTAAT |
| P18: *cgmdh* gene fragment for p10099A-cgmdh (F) | TCCTAGGTACAGTGCTAGCGATGAATTCCCCGCAGAAC |
| P19: *cgmdh* gene fragment for p10099A-cgmdh (R) | GCCAAGCTTGCATGCCTGCATTAGAGCAAGTCGCGCAC |
| P20: *cgmdh* gene fragment for pEKEx1-cgmdh (F) | CAATTTCACACAGGAAACAGATGAATTCCCCGCAGAAC |
| P21: *cgmdh* gene fragment for pEKEx1-cgmdh (R) | AACAGCCAAGCTTGGCTGCATTAGAGCAAGTCGCGCAC |
| P22: Upstream of $P_{mdh}$ sequence for pINPfrdmsmdh (F) | CGGGAAACGTCTTGCTCGAGCAGGTGTATTCGGAGCAATA |
| P23: Upstream of $P_{mdh}$ sequence for pINPfrdmsmdh (R) | TCCGCTGAAAAATCTCGTGCAGTAAATAATTCAGGCAGTT |
| P24: Downstream of $P_{mdh}$ sequence for pINPfrdmsmdh (F) | GGATCTGCCGGTCTCCCTATTTTATCAACTCTACTGGGGA |
| P25: Downstream of $P_{mdh}$ sequence for pINPfrdmsmdh (R) | CCGCCACCGCGGTGGAGCTCATTTTTCACCTAAGTGACGT |
| P26: *lox66-cat-lox77* cassette for pINPfrdmsmdh (F) | ACGTTAGAACGCGGCTACAA |
| P27: *lox66-cat-lox77* cassette for pINPfrdmsmdh (R) | ATAGGGAGACCGGCAGATC |
| P28: $P_{frd}$ sequence for pINPfrdmsmdh (F) | GCACGAGATTTTTCAGCGGA |
| P29: $P_{frd}$ sequence for pINPfrdmsmdh (R) | TTGTAGCCGCGTTCTAACGTTAAGACTCCTTAATGTGGAA |
| P30: *msmdh* gene fragment for pET30a-msmdh (F) | GCGCCATATGAATTCCCCGCAGAACGTCTCCACC |
| P31: *msmdh* gene fragment for pET30a-msmdh (R) | GCGCCTCGAGGAGCAAGTCGCGCACTGCCTCGCGC |
| P32: *asmdh* gene fragment for pET30a-asmdh (F) | GCGCCATATGAAAGTAACCTTATTAGGCGCCAGC |
| P33: *asmdh* gene fragment for pET30a-asmdh (R) | GCGCCTCGAGTTCGCCGGTAACAAATTTCACACC |
| P34: *cgmdh* gene fragment for pET30a-cgmdh (F) | GCGCCATATGAAAGTTGCAGTTCTAGGTGCCGCA |
| P35: *cgmdh* gene fragment for pET30a-cgmdh (R) | GCGCCTCGAGACCGTTAATAAAATCTTCACCTGAC |
| P36: *ecmdh* gene fragment for pET30a-ecmdh (F) | GCGCCATATGAAAGTCGCAGTCCTCGGCGCTGCT |
| P37: *ecmdh* gene fragment for pET30a-ecmdh (R) | GCGCCTCGAGCTTATTAACGAACTCTTCGCCCAG |
| P38: *ylmdh* gene fragment for pET30a-ylmdh (F) | GCGCCATATGGTTAAAGCTGTCGTTGCCGGAGCC |
| P39: *ylmdh* gene fragment for pET30a-ylmdh (R) | GCGCCTCGAGGTTGGCAGGAGGAGGGTTAACAAT |
| P40: *scmdh1* gene fragment for pET30a-scmdh1 (F) | GCGCCATATGTTGTCAAGAGTAGCTAAACGTGCG |
| P41: *scmdh1* gene fragment for pET30a-scmdh1 (R) | GCGCCTCGAGTTTACTAGCAACAAAGTTGACACC |
| P42: *scmdh2* gene fragment for pET30a-scmdh2 (F) | GCGCCATATGCCTCACTCAGTTACACCATCCATA |
| P43: *scmdh2* gene fragment for pET30a-scmdh2 (R) | GCGCCTCGAGAGATGATGCAGATCTCGATGCAAC |
| P44: *scmdh3* gene fragment for pET30a-scmdh3 (F) | GCGCCATATGGTCAAAGTCGCAATTCTTGGCGCT |
| P45: *scmdh3* gene fragment for pET30a-scmdh3 (R) | GCGCCTCGAGTAGCTTGGAAGAGTCTAGGATGAA |

[a]Ap, ampicillin; Km, kanamycin; Cm, chloramphenicol; Sp, spectinomycin; Str, streptomycin; r, resistance.

**Supplementary Table 10. Effect of expressing or overexpressing *Cg*MDH in various SA producers.**

| Strain[a] | Acetic acid (g·L$^{-1}$) | Formic acid (g·L$^{-1}$) | Lactic acid (g·L$^{-1}$) | Pyruvic acid (g·L$^{-1}$) | Succinic acid (g·L$^{-1}$) | Ethanol (g·L$^{-1}$) |
|---|---|---|---|---|---|---|
| ***E. coli* W3110** | 0.521±0.003 | 0.263±0.010 | 0.300±0.004 | 0.153±0.006 | 0.14±0.006 | 0.290±0.008 |
| ***E. coli* (p10099A-cgmdh)** | 0.47±0.026 | 0.200±0.047 | 0.547±0.147 | 0.174±0.014 | 0.431±0.010 | 0.351±0.043 |
| ***C. glutamicum* ATCC13032** | 0.125±0.089 | 0.865±0.145 | 1.036±0.070 | 0 | 0.084±0.118 | 0 |
| ***C. glutamicum* (pEKEx1-cgmdh)** | 0.147±0.001 | 0.117±0.015 | 1.708±0.003 | 0.135±0.003 | 0.69±0.009 | 0 |

[a] Flask cultures were carried out using the *E. coli* W3110, *E. coli* W3110 (p10099A-cgmdh), *C. glutamicum* ATCC13032, and *C. glutamicum* (pEKEx1-cgmdh) strains. Data are presented as mean values ± standard deviation. Standard deviations were generated from n=3 independent experiments. Source data are provided as a Source Data file.

## Supplementary Note 1. Physiological functions of MDH variants.

Rumen bacteria including *A. succinogenes* and *M. succiniciproducens*, are natural SA producers that primarily use the reductive branch of the TCA cycle not only for SA production but also for cell growth[4]. Among enzymes associated with the TCA cycle, MDH that preferentially reduces OAA to malate is one of the key enzymes for anaerobic SA production in rumen bacteria. Although the importance of MDH for SA production has been emphasized in other studies[5-8], physiological study on MDHs of rumen bacteria has never been reported. Nonetheless, *As*MDH was selected for evaluation of its activity along with seven other MDHs from representative SA producers due to the following reasons: (1) wild type *A. succinogenes* can produce SA at a high level under anaerobic condition[9], (2) *A. succinogenes* and *M. succiniciproducens* share similar metabolic characteristics[9], (3) *As*MDH is one of the key enzymes (along with PCKA, FUM, and FRD) for SA production in *A. succinogenes*[9], and (4) *As*MDH and *Ms*MDH are clustered together with the mitochondrial MDH (Supplementary Fig. 2).

Unlike rumen bacteria, wild type *E. coli* produces SA as a minor product under anaerobic condition. Thus, *E. coli* has been metabolically engineered to produce SA using either reductive or oxidative TCA cycle in numerous studies[10]. Under aerobic condition, *E. coli* operates the oxidative branch of the TCA cycle and *Ec*MDH mainly oxidizes malate to OAA. On the other hand, *E. coli* operates the reductive branch of the TCA cycle and *Ec*MDH primarily reduces OAA to malate under anaerobic condition[10]. It should be noted that *Ec*MDH is clustered together with the mitochondrial MDH (Supplementary Fig. 2). According to a study, the aerobic respiration control protein (ArcA) in *E. coli* represses the *mdh* gene expression stronger under anaerobic condition resulting in higher *mdh* gene

expression under aerobic condition[11]. Thus, an *E. coli* strain deleted with the gene encoding ArcA was developed and the resulting strain was able to express the *mdh* gene 1.4-fold higher under anaerobic condition compared to that under aerobic condition[11]. In another study, the overexpression of *Ec*MDH by the *E. coli* NZN111 strain (△*pfl* and △*ldhA*), which could not grow anaerobically on glucose due to limited $NAD^+$ regeneration by inactivation of NADH-dependent lactate dehydrogenase, restored cell growth by regenerating $NAD^+$ and enabled anaerobic SA production (31.9 $g \cdot L^{-1}$ of SA from fed-batch fermentation)[6]. Based on these evidence, we were convinced that the *Ec*MDH can be used to effectively reduce OAA into malate under anaerobic condition and enhance SA production in *M. succiniciproducens*.

*Y. lipolytica* is a strictly aerobic yeast strain that is nonconventional for SA production[10]. *Y. lipolytica* can naturally produce variety of organic acids such as α-ketoglutaric, citric, and isocitric acids and has high tolerance to low pH condition. Such beneficial characteristics enabled researchers to successfully engineer *Y. lipolytica* to produce SA with high performance. Due to strictly aerobic nature of this yeast strain, studies only on the engineering of oxidative TCA cycle for SA production in *Y. lipolytica* have been reported[10]. Thus, detailed physiological study on the *Yl*MDH under anaerobic condition has never been reported. In a study on the analysis of catalytic activities of enzymes involved with TCA cycle in the α-ketoglutaric and citric acids producing *Y. lipolytica* strains, the enzymes associated with oxidative TCA cycle were strongly activated under aerobic condition[12]. However, high enzyme activities of *Yl*MDH, fumarase, and succinate dehydrogenase in *Y. lipolytica* were simultaneously present suggesting that the catalysis by these enzymes proceed significantly toward the direction of reductive branch of the TCA cycle. Based on these evidence, *Yl*MDH, which is clustered together with the mitochondrial MDH (Supplementary Fig. 2), was selected along with seven other MDHs from

representative SA producers for the evaluation of MDH activities.

In contrast to the strictly aerobic *Y. lipolytica*, *S. cerevisiae* is a well characterized yeast strain that can grow under both aerobic and anaerobic conditions[10]. The wild type *S. cerevisiae* does not naturally produce SA as an end product, especially under anaerobic condition, due to the possession of a fumarase, which catalyzes the reversible hydration of fumarate to malate, and fumarate reductase, which is expressed only under anaerobic condition. However, bio-based SA production using either reductive branch or oxidative TCA cycle has been achieved by metabolic engineering of *S. cerevisiae*[10]. Three isozymes of *Sc*MDH are present in *S. cerevisiae*: *Sc*MDH1, a mitochondrial enzyme; *Sc*MDH2, a cytosolic enzyme known to possess higher OAA reduction activity than malate oxidation activity; *Sc*MDH3, a glyoxysomal enzyme involved with the gluconeogenesis pathway and reoxidation of NADH produced from fatty acid β-oxidation[13]. Among the three isozymes described above, the overexpression of *Sc*MDH2 in *S. cerevisiae* was reported to enhance oxaloacetate reduction and eventually increase malate production[14]. As each of the isozymes of *Sc*MDH are expressed in different locations and have distinctive physiological functions in SA producing *S. cerevisiae*, we thought it would be interesting to evaluate these isozymes along with other MDHs from different microorganisms for their OAA reduction activities.

## Supplementary Note 2. Discussion on the possible effect of *Cg*MDH expression on the inhibition of FRD by OAA.

Several previously studied FRDs, including *E. coli* FRD, were found to possess *ki* values in the nM range for OAA. Despite FRD having such nM *ki* value, a metabolically engineered *E. coli* strain was reported to produce a large amount of SA under anaerobic condition[15]. The intracellular OAA concentration in *E. coli* was reported to be 30 μM[16]. This means that the FRD responsible in SA production in *E. coli* was not too much inhibited by the presence of 30 μM OAA. A couple of other studies have also reported that the intracellular OAA concentrations are in μM range. For example, the intracellular OAA concentrations of *C. glutamicum*[17] and *Saccharopolyspora erythraea*[18] were reported to be 7~88 μM and 75 $nmol \cdot gDCW^{-1}$, respectively; for comparison, the intracellular OAA concentration in PALKcgmdh strain we measured is equivalent to $120.3 \pm 36.23\ nmol \cdot gDCW^{-1}$. Furthermore, the *ki* values reported previously for other FRDs were all measured *in vitro* using the purified FRDs. Taken together, the actual *in vivo* inhibitory OAA concentration of FRD might be different from the inhibitory OAA concentration determined *in vitro*. It should also be noted that FRD is a membrane-bound protein[19], and thus OAA might be very weakly accessible to FRD.

Introduction of *Cg*MDH in the *M. succiniciproducens* PALK strain reduced the intracellular OAA concentrations from 26.11±4.47 (PALK) to 12.22±3.68 μM (PALKcgmdh) (Supplementary Fig. 5). Hence, if the actual *in vivo* inhibitory OAA concentration of *M. succiniciproducens* FRD is in the μM range (as observed in *E. coli*, which still efficiently produced SA even though *in vitro* inhibitory OAA concentration was in nM range), enhanced

SA production by the PALKcgmdh strain would have also been contributed by less inhibition of FRD by lower concentration of OAA. Further studies are needed to confirm this.

## Supplementary References


1. Choi, S. et al. Highly selective production of succinic acid by metabolically engineered *Mannheimia succiniciproducens* and its efficient purification. *Biotechnol. Bioeng*. **113,** 2168-2177 (2016).
2. Ahn, J.H., Lee, J.A., Bang, J. & Lee, S.Y. Membrane engineering via trans-unsaturated fatty acids production improves succinic acid production in *Mannheimia succiniciproducens*. *J. Ind. Microbiol. Biotechnol*. **45,** 555-556 (2018).
3. Kim, J.M., Lee, K.H., & Lee, S.Y. Development of a markerless gene knock-out system for *Mannheimia succiniciproducens* using a temperature-sensitive plasmid. *FEMS Microbiol. Lett*. **278,** 78 (2008).
4. Hong, S.H. et al. The genome sequence of the capnophilic rumen bacterium *Mannheimia succiniciproducens*. *Nat. Biotechnol.* **22,** 1275-1281 (2004).
5. Liang, L.-y. et al. Increased production of succinic acid in *Escherichia coli* by overexpression of malate dehydrogenase. *Biotechnol. Lett*. **33,** 2439-2444 (2011).
6. Wang, W., Li, Z., Xie, J. & Ye, Q. Production of succinate by a *pflB ldhA* double mutant of *Escherichia coli* overexpressing malate dehydrogenase. *Bioprocess Biosyst. Eng*. **32,** 737 (2009).
7. Yan, D. et al. Construction of reductive pathway in *Saccharomyces cerevisiae* for effective succinic acid fermentation at low pH value. *Bioresour. Technol*. **156,** 232-239 (2014).
8. McAlister-Henn, L., Steffan, J.S., Minard, K.I. & Anderson, S.L. Expression and function of a mislocalized form of peroxisomal malate dehydrogenase (MDH3) in yeast. *J. Biol. Chem*. **270,** 21220-21225 (1995).
9. Dessie, W. et al. Opportunities, challenges, and future perspectives of succinic acid production by *Actinobacillus succinogenes*. *Appl. Microbiol. Biotechnol*. 102, 9893-9910 (2018).
10. Lee, J. A., Ahn, J. H. & Lee, S. Y. 3.15 Organic Acids: Succinic and Malic Acids. In: Moo-Young, M., (eds) Comprehensive Biotechnology. Vol. 3, (Elsevier, Amsterdam, 2019).
11. Park S. J., Cotter P. A. & Gunsalus R. P. Regulation of malate dehydrogenase (mdh) gene expression in *Escherichia coli* in response to oxygen, carbon, and heme availability. *J. Bacteriol.* **177,** 6652-6656 (1995).
12. Il'chenko, A. P. et al., Metabolism of *Yarrowia lipolytica* grown on ethanol under conditions promoting the production of α-ketoglutarate and citric acids: a comparative study of the central metabolism enzymes, *Microbiology*, **71,** 269-274 (2002).
13. Moriyama, S., Nishio, K. & Mizushima, T. Structure of glyoxysomal malate de-hydrogenase (MDH3) from *Saccharomyces cerevisiae. Acta. Crystallogr. F* **74,** 617-

624 (2018).

14. Pines, O., Shemesh, S., Battat, E. & Goldberg, I. Overexpression of cytosolic malate dehydrogenase (MDH2) causes overproduction of specific organic acids in *Saccharomyces cerevisiae*. *Appl. Microbiol. Biotechnol.* **48,** 248-255 (1997).
15. Vemuri, G., Eiteman, M. & Altman, E. Succinate production in dual-phase *Escherichia coli* fermentations depends on the time of transition from aerobic to anaerobic conditions. *J. Ind. Microbiol. Biotechnol.* **28,** 325-332 (2002).
16. Peng, L., Arauzo-Bravo, M.J. & Shimizu, K. Metabolic flux analysis for a ppc mutant *Escherichia coli* based on $^{13}$C-labelling experiments together with enzyme activity assays and intracellular metabolite measurements. *FEMS Microbiol. Lett*. **235**, 17-23 (2004).
17. Petersen, S. et al. Metabolic consequences of altered phosphoenolpyruvate carboxykinase activity in *Corynebacterium glutamicum* reveal anaplerotic regulation mechanisms *in vivo. Metab. Eng*. **3,** 344-361 (2001).
18. Hong, M., Mou, H., Liu, X., Huang, M. & Chu, J. $^{13}$C-assisted metabolomics analysis reveals the positive correlation between specific erythromycin production rate and intracellular propionyl-CoA pool size in *Saccharopolyspora erythraea*. *Bioproc. Biosyst. Eng.* **40,** 1337-1348 (2017).
19. Iverson, T.M., Luna-Chavex, C., Cecchini, G. & Rees, D.C. Structure of the *Escherichia coli* fumarate reductase respiratory complex. *Science*. **284**, 1961-1966 (1999).
