# Supplementary material for: Enhanced succinic acid production by Mannheimia employing optimal malate dehydrogenase
Source: Nat Commun. 2020 Apr 23;11:1970. doi: 10.1038/s41467-020-15839-z (PMC7181634; doi:10.1038/s41467-020-15839-z)
Supplement: Supplementary file 4 — Description of Additional Supplementary Files [file 41467_2020_15839_MOESM4_ESM.docx]

**Description of Additional Supplementary Files**

File name: Supplementary Data 1
Description: Amino acid sequences used in the phylogenetic analysis. The position of G11Q is indicated by a reversed triangle in the alignment images.

File name: Supplementary Data 2
Description: Fed-batch fermentation results of the engineered *M. succiniciproducens* strains.
