# Supplementary material for: Enhanced succinic acid production by Mannheimia employing optimal malate dehydrogenase
Source: Nat Commun. 2020 Apr 23;11:1970. doi: 10.1038/s41467-020-15839-z (PMC7181634; doi:10.1038/s41467-020-15839-z)
Supplement: Supplementary file 5 — Supplementary Data 1 [file 41467_2020_15839_MOESM5_ESM.docx]

**Supplementary Data 1.** Amino acid sequences used in the phylogenetic analysis. The position of G11Q is indicated by a reversed triangle in the alignment images.


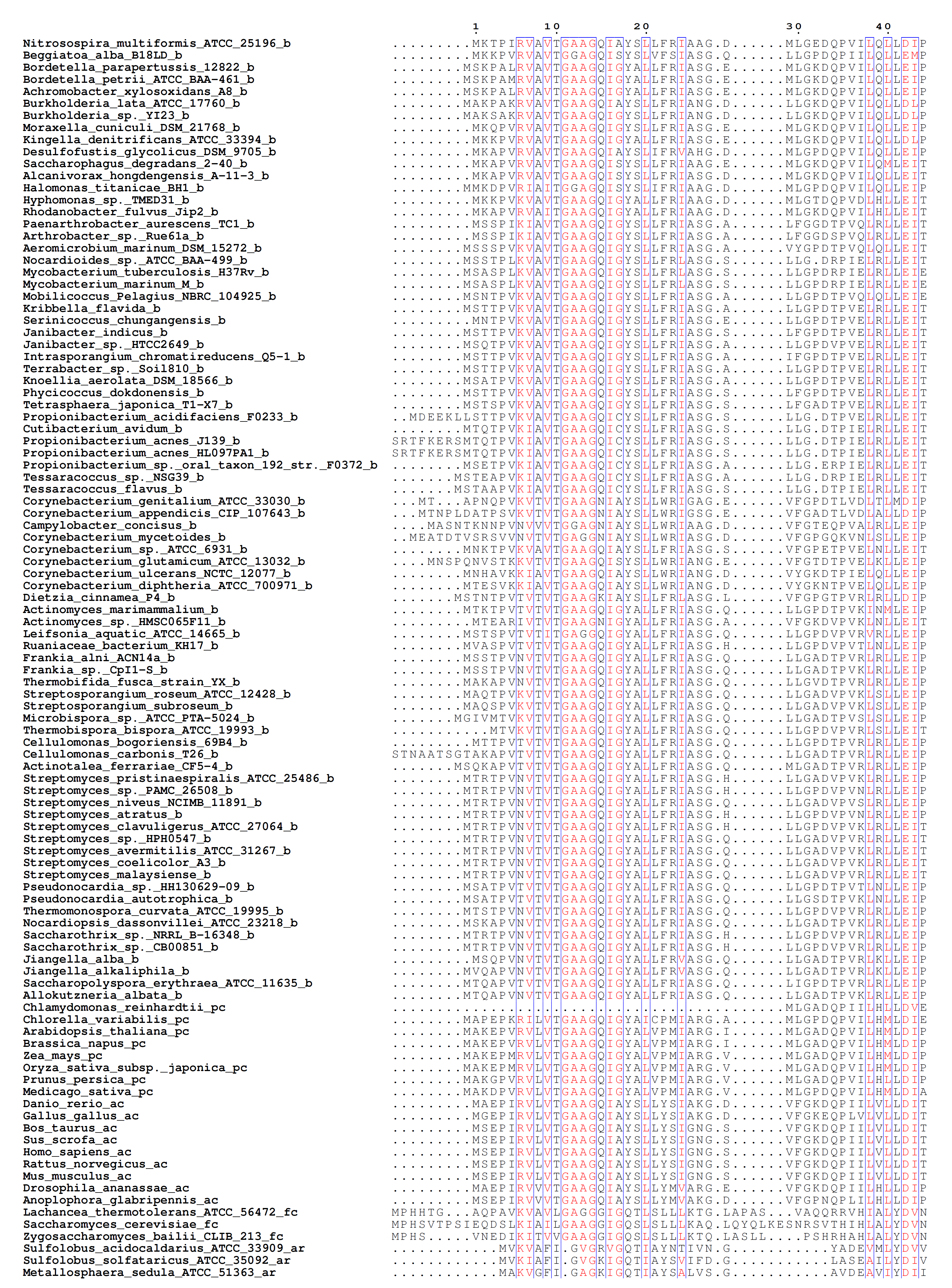


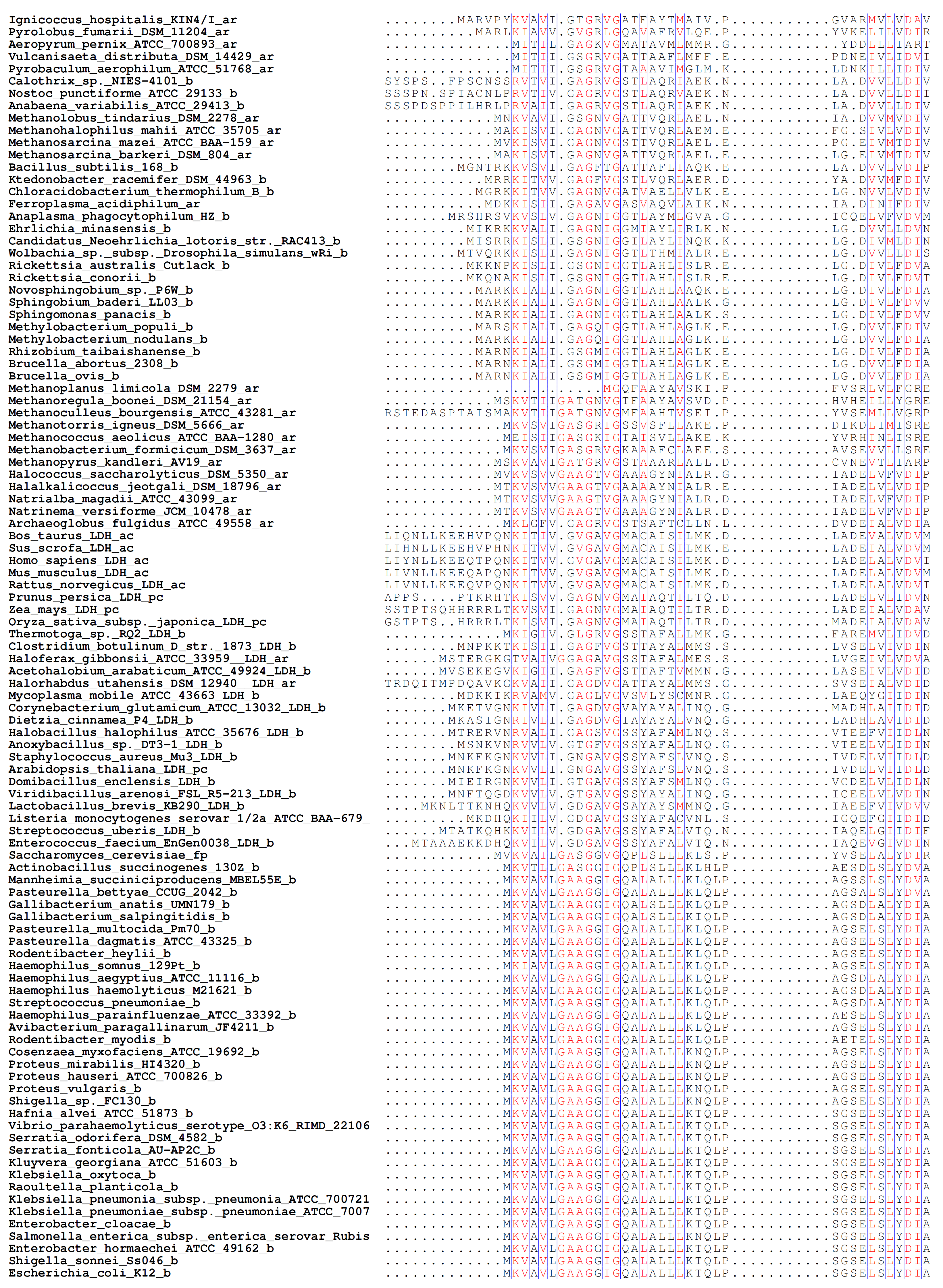


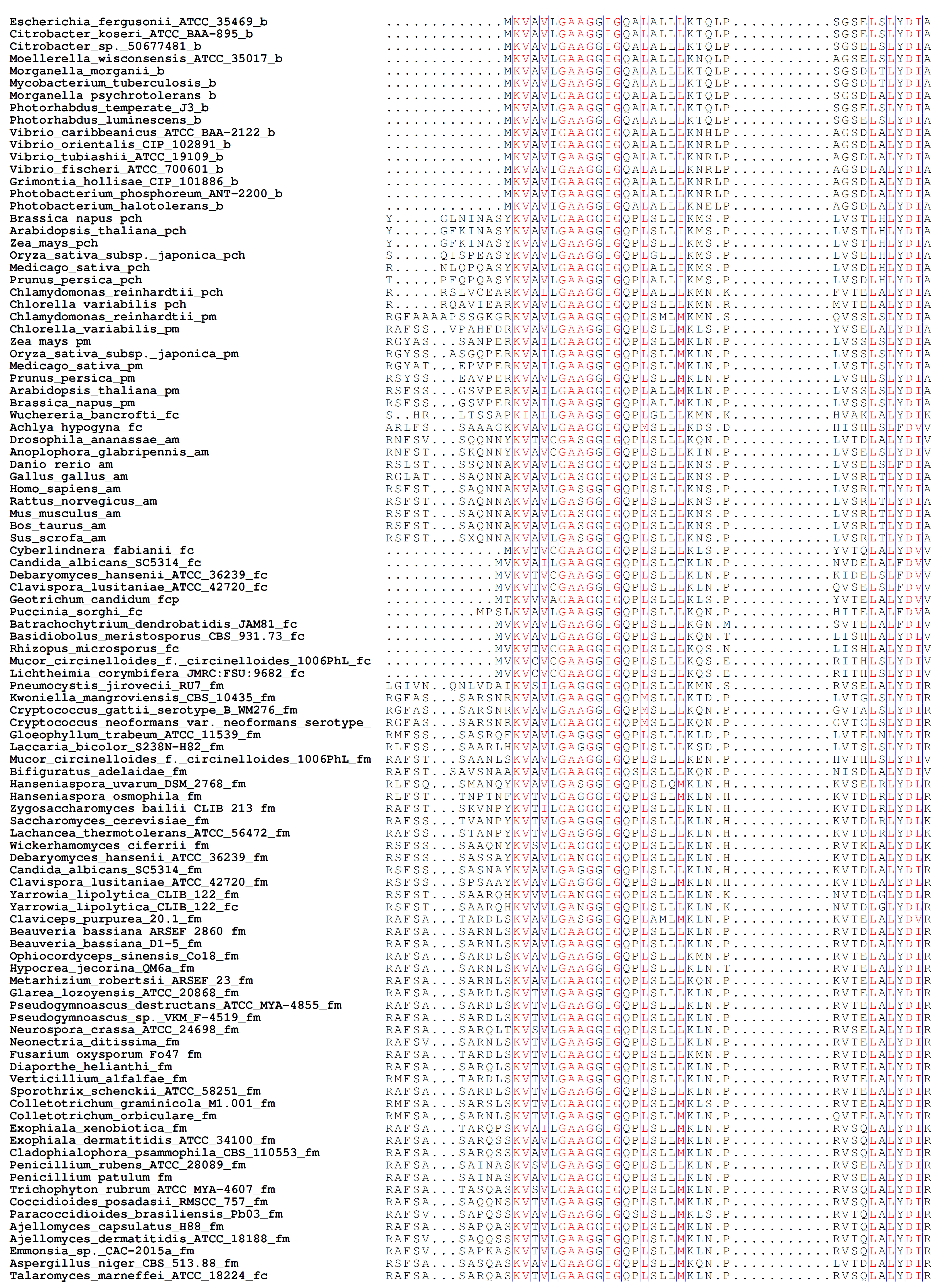

>Corynebacterium_glutamicum_ATCC_13032_b

MNSPQNVSTKKVTVTGAAGQISYSLLWRIANGEVFGTDTPVELKLLEIPQALGGAEGVAMELLDSAFPLLRNITITADANEAFDGANAAFLVGAKPRGKGEERADLLANNGKIFGPQGKAINDNAADDIRVLVVGNPANTNALIASAAAPDVPASRFNAMMRLDHNRAISQLATKLGRGSAEFNNIVVWGNHSATQFPDITYATVGGEKVTDLVDHDWYVEEFIPRVANRGAEIIEVRGKSSAASAASSAIDHMRDWVQGTEAWSSAAIPSTGAYGIPEGIFVGLPTVSRNGEWEIVEGLEISDFQRARIDANAQELQAEREAVRDLL

>Corynebacterium_ulcerans_NCTC_12077_b

MNHAVKKIAVTGAAGQIAYSLLWRIANGDVYGKDTPIELQLLEIPQAIGGAEGVAMELLDSAFPLLKNITVTDSADVAFDGTNAAFLVGAKPRGKGEERAALLTANGKIFGPQGDALNRNAADDIRVLVVGNPANTNALIAQSAAKDIPADRFNAMMRLDHNRGISQLADKINRDKNGIENFVVWGNHSAGQFPDIAYATVDGEKLADLVDDVWYRDEFIPRVAKRGAEIIEVRGKSSAASAASSAIDHMHDWINGTEGQWRTAAIPSDGSYGVPEGLIFGFPTIAEGGEWKIVDGLELSDFQKESIARNVKELEEERAAVADLLK

>Corynebacterium_diphtheria_ATCC_700971_b

MTESVKKIAVTGAAGQIAYSLLWRIANGDVYGKNTPVELQLLEIPQAIGGAEGVAMELLDSAFPLLKNIVVTDKAEVAFDGTNAAFLVGAKPRGKGEERADLLTANGKIFGPQGKALNDNAADDIRVLVVGNPANTNALIAQHAAKDIPADRFNAMMRLDHNRGIAQLSEKLGRDKNDIEKFVVWGNHSAGQFPDITYATIGGEAISGLVDHDWYTGEFIPRVAKRGAEIIEVRGKSSAASAASSAIDHMHDWINGTDGQWRTAAIPSDGSYGVPEGLIFGFPTISEDGQWKIVQDLELSDFQKDGIARNVTELEEEREAVKDLLG

>Corynebacterium_genitalium_ATCC_33030_b

MTAPNQPVKVTVTGAAGNIAYSLLWRIGAGEVFGPDTLVDLTLMDIPDNVQKVDGVAMELSDSALNKICNLHVTDDAQKAFDGTNAAFLVGAKPRGKGESRADMLEANGKIFIDQGRSINDHAADDVRVLVIGNPANTNTYIANKAAPDVGPEHFNALMRLDHNRTISMLSQKLDEHSSRFESIAVWGNHGDTMFPDIEYATVGGQPVKDRVERDWYVEEFIPRVAKRGSEIIEVRGRSSAASAASAAVDHMRDWVAGTEGKWVTSAVVSNGSYGVDEGLVFGLPVIGINGRWSVVEGLDLNEFQRERIDANIEALRAEAEIADKLF

>Dietzia_cinnamea_P4_b

MSTNTPVTVTVTGAAGKIAYSLLFRLASGLVFGPGTPVRLRLLDIPSAVVAAEGVAMEIEDCALPLVDSIEITDDVTTAFDGANAAFLVGARPRGKGMERADLLTANGAIFREQGRAINDHAADDIRVVVVGNPANTNAYIAHRHAPDVPAGRFTALMRLDHNRALSQLATRLGARVSELDGVVVWGNHSATQFPDVTYLTREGEPVTAGLDPEWLADTFIPRVAGRGAEIIAVRGSSSAASAAGAAVDQMRDWVCGTSGESWTTVALPSAGEYGTVPGVVTGLPCRSRGGEWEVVEGLEIGEFQRARIDASVAELVSERDAVRALGLAD

>Leifsonia_aquatic_ATCC_14665_b

MSTSPVTVTITGAGGQIGYALLFRIASGQLLGPDVPVRVRLLEIPAGRSAAEGTALELQDGAFPLLHSVDVTEDANAAFDGANVALLVGARPRGAGMERADLLEANGAIFGPQGAAINAGAADDIRVLVVGNPANTNALIASAHAPDVPAERFTAMTRLDHNRAVAQLAAKLDVPVTSIERVIVWGNHSASQYPDLSHATVDGRPATELVDERWLADEYIPRVAKRGAEIIQVRGSSSAASAASAAIDHVHDWVNGTGDRWTSAAIVSDGSYGVPAGLISSFPVRAVDGEWRIVDGLTIDDFSRQRIDASVAELVEERDAVRALGLL

>Cellulomonas_carbonis_T26_b

MSQSTNAATSGTAKAPVTVTVTGAAGQIGYALLFRIASGQLLGADTPVRLRLLEIPQGVKAAEGTAMELDDCAFPLLRSIDIADDPRVGFEGANVALLVGARPRTAGMERGDLLEANGGIFKPQGEAINAVAADDVRVLVVGNPANTNALIAASHAPDVPAERFTAMTRLDHNRALSQLAQKTGAGVADIQGLTIWGNHSATQYPDVFHATVAGRPATELVDETWLAETFIPTVAKRGAAIIEARGASSAASAANAAVDHVRDWVLGTPEGTWTSAAIPSDGSYGVPEGLISSFPVTSTGGRWEIVQGLDVNEFSRQRIDASITELVEEREAVRGLGLV

>Thermobifida_fusca_strain_YX_b

MAKAPVNVTVTGAAGQIGYALLFRIASGQLLGVDTPVRLRLLEIPQAIKAAEGTAMELDDCAFPLLAGVDIYDDPRKAFDGVNVALLVGARPRTKGMERRDLLEANGGIFGPQGAAINDGAADDVRVLVVGNPANTNALIAQAHAPDIPADRFTAMTRLDHNRALSQLAAKLNVSVSDIKKLTIWGNHSATQYPDIFHAEVNGRSAVEAVNDEEWLRDTFIPTVAKRGAAIIEARGASSAASAANAAIDHVYDWVNGTPEGDWTSVALPSDGSYGVPEGLVSSFPVVSRNGSWEIVQGLEINEFSRERIDASVRELEEEREAVRALGLIK

>Streptosporangium_roseum_ATCC_12428_b

MAQTPVKVTVTGAAGQIGYALLFRIASGQLLGADVPVKLSLLEIPQAVKAAEGTAMELDDCAFPLLSGIEVTDDANKAFDGASVALLVGARPRTAGMERGDLLQANGGIFGPQGKAINDNAADDIRVLVVGNPANTNSLIAQQHAPDVPAERFTAMTRLDHNRALSQLAAKLQVPVAEIKKMTIWGNHSATQYPDLFHAEVGGKIAAEQVDEAWLRDTFIPTVAKRGAAIIEARGASSAASAANAAIDHVFDWVNGTEWTSAAIPSDGSYGVPEGLISSFPVASRDGKWEIIQGLEIDAFSRERIDASVRELEEERAAVRELGLI

>Microbispora_sp._ATCC_PTA-5024_b

MGIVMTVKVTVTGAAGQIGYALLFRIASGQLLGADTPVSLSLLEIPQAVKAAEGTAMELDDCAFPLLRGIEISDDPARAFDGANVALLVGARPRTAGMERGDLLEANGGIFGPQGKAINDHAADDIRVLVVGNPANTNALIAQSAAPDVPAERFTAMTRLDHNRALSQLAAKLRVPVTEIRKLTIWGNHSATQYPDLFHAEVGGKIAAEQVEEEWLRDTFIPTVAKRGAAIIEARGASSAASAANAAIDHVHDWVNGTPEGDWTSAALPSDGSYGVPEGLISSFPVVSRGGRWEIVQGLEIDSFSRQRIDASVNELVEERDAVRALGLI

>Thermobispora_bispora_ATCC_19993_b

MTVKVTVTGAAGQIGYALLFRIASGQLLGPDVPVRLSLLEITPALKAAEGTAMELDDCAFPLLRGVDITDDPVKAFDGANVALLVGARPRTAGMERKDLLEANGGIFGPQGKAINDHAADDIRVLVVGNPANTNALIAKAHAPDVPADRFTAMTRLDHNRAISQLAQKLNVPVTDIKKMTIWGNHSTTQYPDLFHTEVGGKIAAELVEEEWLRDTFIPTVAKRGAAIIEARGASSAASAANAALNHVYDWVNGTPEGDWVSVALPSDGSYGVPEGLVCSFPAVSRNGRWEIVQGLEINEFSRERIDASVRELIEERDAVKALGLI

>Nocardiopsis_dassonvillei_ATCC_23218_b

MSKAPVNVTVTGAAGQIGYALLFRIASGQLLGADTPVKLRLLEIPQAVKAAEGTAMELDDCAFPLLQGIDIFDDATQAFQGANVALLVGARPRGKGMERGDLLEANGGIFKPQGEAINAGAADDIRVLVVGNPANTNALIAQSHAPDVPAERFTAMTRLDHNRALTQLAKKLDVSINDIKKLTIWGNHSATQYPDVFHAEVNGANAAKAVGDQAWLENDFIPTVAKRGAAIIEARGASSAASAANAAVDHVYDWVNGTPEGDWTSAAIPSDGSYGVPEGIISSFPVVSRGGRWEIVQGLEIDDFSRARIDASVKELVEERDTVKQLGLV

>Streptomyces_pristinaespiralis_ATCC_25486_b

MTRTPVNVTVTGAAGQIGYALLFRIASGHLLGADVPVKLRLLEIPQGLKAAEGTAMELDDCAFPLLKGIEITDDPNVGFDGANVALLVGARPRTKGMERGDLLSANGGIFKPQGKAINDNAADDVRVLVVGNPANTNALIAQAAAPDVPAERFTAMTRLDHNRAISQLAAKTGASVSDIKRLTIWGNHSATQYPDIFHAEIAGKSAAEIVDDEQWLADTFIPTVAKRGAAIIEARGASSAASAANAAIDHIHTWVNGTAEGDWTSMGIPSDGSYGVPEGLISSFPVVCKDGKYEIVQGLDINEFSRTRIDASVRELEEERDAVRELGLL

>Streptomyces_niveus_NCIMB_11891_b

MTRTPVNVTVTGAAGQIGYALLFRIASGQLLGADVPVSLRLLEIPQGLKAAEGTAMELDDCAFPLLRGIEITDDANAAFSGANVALLVGARPRTKGMERGDLLSANGGIFKPQGKAINDHAADDIKVLVVGNPANTNALIAQAAAPDVPAERFTAMTRLDHNRAISQLAAKTGAAVTDIKRLTIWGNHSATQYPDIFHAEIAGKNAAETVNDEQWLADTFIPTVAKRGAAIIEARGASSAASAANAAIDHVHTWVNGTPAGDWTSMGIPSDGSYGVPEGIISSFPVTTKDGRYEIVQGLDINDFSRARIDASVKELTEERDAVRELGLL

>Streptomyces_coelicolor_A3_b

MTRTPVNVTVTGAAGQIGYALLFRIASGQLLGADVPVKLRLLEITPALKAAEGTAMELDDCAFPLLQGIEITDDPNVAFDGANVALLVGARPRTKGMERGDLLEANGGIFKPQGKAINDHAADDIKVLVVGNPANTNALIAQAAAPDVPAERFTAMTRLDHNRALTQLAKKTGSTVADIKRLTIWGNHSATQYPDIFHATVAGKNAAETVNDEKWLADEFIPTVAKRGAAIIEARGASSAASAANAAIDHVYTWVNGTAEGDWTSMGIPSDGSYGVPEGIISSFPVTTKDGSYEIVQGLDINEFSRARIDASVKELSEEREAVRGLGLI

>Streptomyces_avermitilis_ATCC_31267_b

MTRTPVNVTVTGAAGQIGYALLFRIASGQLLGADVPVKLRLLEITPALKAAEGTAMELDDCAFPLLQGIDITDDPNVAFDGTNVGLLVGARPRTKGMERGDLLSANGGIFKPQGKAINDNAADDVKILVVGNPANTNALIAQAAAPDVPAERFTAMTRLDHNRALTQLAKKTGSTVADIKRLTIWGNHSATQYPDIFHASVAGKNAAEVVNDEKWLAEDFIPTVAKRGAAIIEARGASSAASAANAAIDHVYTWVNGTADGDWTSMGIPSDGSYGVPEGLISSFPVTTKDGRYEIVQGLEINEFSRARIDASVKELEEEREAVRALGLI

>Actinomyces_marimammalium_b

MTKTPVTVTVTGAAGQIGYALLFRIASGQLLGPDTPVKINMLEIPPALKAAEGTAMELNDCAFPLLDSINITDDANKGFDGANVALLVGARPRTKGMERADLLKANGGIFGPQGKAINDHAADDIRVLVVGNPANTNALIASAAAPDVPASRFTAMMRLDHNRAISQLAEKLNVPVADIKKMLVWGNHSADQYPDVSYATVAGKPAADMVDSAWLEEYFVPTVAKRGAAIIEARGASSAASAANAAIDHVYNWVNGTPENDWVTAGVASDGSYGVPEGLIYGFPCTSKGAEWEIVQGLELSEATKAGIQKNIEALESERDAVRELGLL

>Pseudonocardia_sp._HH130629-09_b

MSATPVTVTVTGAAGQIGYALLFRIASGQLLGPDTPVTLNLLEIPQAVKAAEGTAMELDDCAFPLLRGIDIFDDANAAFSGTNVALLVGARPRSKGMERGDLLEANGGIFGPQGKAINAGAADDVRVLVVGNPANTNALIAQAAAPDVPAERFTAMTRLDHNRALAQLSKKLGVGLDEISKLTIWGNHSATQYPDLFHAEVGGKIAAEQVEREWLENDFIPTVAKRGAAIIEARGASSAASAANAAIDHVYDWVNGTPAGDWTSAAIPSDGSYGVPEGIISSFPVTSENGAWKIVQGLEIDEFSRGKIDASVAELVEERDTVAKLGLI

>Saccharopolyspora_erythraea_ATCC_11635_b

MTQAPVTVTVTGAAGQIGYALLFRIASGQLIGPDTPVRLRLLEIPQAVKAAEGTAMELDDCAFPLLQGIEVTDDARTAFDGTNVALLVGARPRTKGMERGDLLEANGGIFKPQGEAINAGAADDVRVLVVGNPANTNALIAQAHAPDVPAERFTAMTRLDHNRALTQLAQKLGVSVNDIKKLTIWGNHSATQYPDLFHAEVNGKVAAEQVDQAWLADTFIPTVAKRGAAIIEARGASSAASAANAAIDHIHTWVNGTPEGDWTSAAVVSDGSYGVPEGLISSFPVVARDGRYEIVQGLEIDEFSRQRIDASVNELSEERDAVRKLGLL

>Intrasporangium_chromatireducens_Q5-1_b

MSTTPVKVAVTGAAGQIGYSLLFRIASGAIFGPDTPVELRLLEITPALKALEGVVMELDDCAFPTLAGVQIGDDATKMFDGVNFALLVGARPRGPGMERGDLLEANGGIFAPQGKALNEVAADDIRVVVTGNPANTNALIAMSNAPDIPNERFSALTRLDHNRAISQLAAKLGVPVTEVKKMTIWGNHSATQYPDIFHAEVGGKNAAEAVGDQDWIENTFIPTVAKRGAAIIEARGASSAASAASATIDHARTWASGTAEGDWVSMSVCSDGSYGVQEGLVSSFPVTVKDGQWSIVQGLEINDFSRGKIDASVAELADERDAVRQLGLI

>Jiangella_alba_b

MSQPVNVTVTGAAGQIGYALLFRVASGQLLGADTPVRLKLLEIPPAVKAAEGTAMELDDCAFPLLSGIDIYDDPRQAFDGVNVALLVGARPRTKGMERGDLLEANGGIFKPQGEAINAGAADDVRVLVVGNPANTNALIAQSHAPDVPADRFTAMTRLDHNRALSQLSAKLGVGVADIAKLTIWGNHSATQYPDIFHAEVAGKPASELVDEAWLTDDFIPTVAKRGAAIIEARGASSAASAANAAIDHVYDWVNGTPEGGWTSAAIVSDGSYGVPAGLISSFPVVSRGGAWEIVQGLEVSDFSRERIDASVRELTEERDAVAALGLV

>Frankia_alni_acN14a_b

MSSTPVNVTVTGAAGQIGYALLFRIASGQLLGADTPVRLRLLEIPQAVRAAEGTALELEDSAFPLLAGVDVFDDAKRAFEGTNVALLVGARPRTKGMERGDLLSANGGIFKPQGEAINSGAAEDIRVLVVGNPANTNALIAQTHAPDVPAERFTAMTRLDHNRAIAQLAKKLGVPSAEIRKITIWGNHSATQYPDIFHAQVGGRSGAEAVGDQKWIAEEFIPRVAKRGAEIIEVRGASSAASAASAAIDHVYTWVNGTPEGDWTSAAIPSDGSYGVPEGLISSFPVTAAGGKFEIVQGLELDAFSREKIDASVRELAEEREAVRALGLI

>Campylobacter_concisus_b

MASNTKNNPVNVVVTGGAGNIAYSLLWRIAAGDVFGTEQPVALRLLEIPAAVGAAEGTAMELADSAMPLVASVDVTDDERKAFDGAQSAFLVGAKPRGKGESRADMLEANGKIFIGQGRAINDGAADDIRVLVVGNPANTNAYIAAKAAPDVPAERFNAMMRLDHNRAVSVLSGKVAEPSTSIERLCVWGNHGDTQFPDLTYAQVDGEPVEVDRNWYVDEFIPRIAGRGAEIIEVRGKSSAASAASAAVDHMRDWVQGTNGRWTTAAVPSTGEYGVDEGLMFGFPATAEDGQWRVVEGLELSDFQRERIDANIEALRAEAAVADKLF

>Corynebacterium_sp._ATCC_6931_b

MNKTPVKVAVTGAAGQIAYSLLFRIASGSVFGPETPVELNLLEITPALHATEGVAMELFDSAFPLLTGINITDDPAKAFDGANAAFLVGAKPRGKGEERSALLGANGKIFGPQGAALNDHAADDIKVLVVGNPANTNTMIAASHAKDIPAERFTAMMRLDHNRSLSQLSQKIGVPTTEIKNMIVWGNHSADQFPDITYATIAGEKVSDKVDAAWVEQDFIPRVAKRGAEIIEVRGSSSAASAAAAAVDHMRDWVTGTPEGEWVSVALPSDGSYGIDEGLVAGVPCYAKDGEWVRVEGLDLSEVQRAGIERNVKALREERDAVKDLLV

>Corynebacterium_appendicis_CIP_107643_b

MTNPLDATPSVKVTVTGAAGNIAYSLLWRIGSGEVFGADTLVDLALLDIPSAVGTVDGVAMELNDSALGNVRSITVTDDPAKAFDGANAAFLVGAKPRGEGESRADMLAANGKIFVEQGRAINDHAADDVRVLVVGNPANTNAYIANKAAPDVGAEHFNALMRLDHNRAISMLSEKLGEHSSAFENIVVWGNHSDTQFPDTTYATVGGESVADRVEREWYENEFIPRVAKRGGEIIKVRGRSSAASAASAAVDHMRDWVTGTEGKWTTAAVVSKGSYGVDEGLVFGFPVIGINGRWGVVEGLELDDDQRARVDANIEALRAEAEIADKLF

>Corynebacterium_mycetoides_b

MEATDTVSRSVVNVTVTGAGGNIAYSLLWRIASGDVFGPGQKVNLSLLEIPSAVRVAEGVAMELADSAFPLLNSVTVTDDPRRGFDGANAAFLVGAKPRGKGESRKDLLTANGRIFTEQGSALNDAAADDIRVLVVGNPANTNAYIAAKAARDIPAERFNAMMRLDHNRAASQLAAKLGAAVGDIDGLVVWGNHSDTQFPDIEYLRVAGEQATGRVDRAWYVDEFIPRVAQRGAEIISVRGSSSAASAASAAVDHMRDWVQGTGGRWTTVARASTGEYGVDEGLVFGFPTVGINGRWAVVEGLDLTEFQRSRINANVEALREEAAVADSLF

>Dietzia_cinnamea_b

MNTNTPVTVTVTGAAGKIAYSLLFWLASGLVFGPGTPVRLRLLDIPSAVVAAEGVAMEIEDCALPLVDSIEITDDVTEAFDGANAAFLVGARPRGKGMERADLLTANGAIFREQGRAINDHAADDIRVVVVGNPANTNAYIAHRHAPDVPAGRFTALMRLDHNRALSQLATRLGARVSELDGVVVWGNHSATQFPDVTYLTRGGEPVTAGLDPEWLADTFIPRVAGRGAEIIAVRGSSSAASAAGAAVDQMRDWVRGTSGESWTTVALPSGGEYGTVPGVVTGLPCRSRGGEWEVVEGLEISDFQRARIDASVAELVSERDAVRALGLAD

>Ruaniaceae_bacterium_KH17_b

MVASPVTVTVTGAAGQIGYALLFRIASGHLLGPDVPVTLNLLEIPQAVKAAEGTAMELDDCAFPLLKGINITDDANVAFDGANVALLVGARPRGAGMERADLLAANGGIFGPQGKAINDNAADDIKVLVVGNPANTNALIAAHSAPDVPIERFNAMMRLDHNRAISQLATKTGAAVTEISKMTVWGNHSASQYPDIFNAVVGGTPASELVDQEWLESYFIPTVAKRGAAIIDARGASSAASAANAAIDHVYDWVNGTPEGDWVTAGIPSDGSYGVPEGIIAGFPVTSNGGSWEIVQGLDLSDFSRARVDASVAELVEEREAVQELGLLG

>Actinotalea_ferrariae_CF5-4_b

MSQKAPVTVTVTGAAGQIGYALLFRIASGQMLGADTPVRLRLLEIPQGVKAAEGTAMELDDCAFPLLQSIDITDDARVGFEGANVALLVGARPRSAGMERGDLLEANGGIFKPQGEAINAVAADDVRVLVVGNPANTNALIAQQHAPDVPAERFTAMTRLDHNRALSQLAQKTGTGVSEIQGLTIWGNHSATQYPDVFHATVAGKPVTDLVDEAWLTSTFIPTVAKRGAAIIEARGASSAASAANAAVDHVRDWVLGTPEGSWTSAAIVSDGSYGVPEGLISSFPVTSAGGAWEIVQGLEVGDFSRERIDASVAELSEERDAVRELGLI

>Cellulomonas_bogoriensis_69B4_b

MTTPVTVTVTGAAGQIGYALLFRIASGQLLGPDTPVRLRLLEIPQGVKAAEGTAMELDDCAFPLLRSIDISDDARVGFEGTNVALLVGARPRSKGMERGDLLEANGGIFKPQGEAINAVAADDVRVLVVGNPANTNALIASAHAPDVPDERFTAMTRLDHNRALSQLAARTGAGVTDIQGLTIWGNHSATQYPDVSHTTVAGRPATDLVDEAWLTDEFIPTVAKRGAAIIEARGASSAASAANAAIDHVRDWVLGTPEGSWTSAAIPSDGSYGVPEGVISSFPVVSRGGAWEVVQGLEVNDFSRARIDASVAELVEERDAVKGLGLV

>Streptosporangium_subroseum_b

MAQSPVKVTVTGAAGQIGYALLFRIASGQLLGADVPVKLSLLEIPQAVKAAEGTAMELDDCAFPLLSGIEVTADPNVAFDGTNVALLVGARPRTAGMERGDLLQANGGIFGPQGKAINDHAADDIRVLVVGNPANTNSLIAQQHAPDVPAERFTAMTRLDHNRALSQLAAKLQVPVTEIKKMTIWGNHSATQYPDLFHAEVGGKIAAELVDEAWLRDTFIPTVAKRGAAIIEARGASSAASAANAAIDHVFDWVNGTEWTSAAIPSDGSYGVPEGIISSFPVASRNGKWEIIQGLEINAFSRERIDASARELEEERAAVRELGLI

>Streptomyces_atratus_b

MTRTPVNVTVTGAAGQIGYALLFRIASGHLLGPDVPVNLRLLEIPQGLKAAEGTAMELDDCAFPLLRGIEITDDANKAFDGANVALLVGARPRTKGMERGDLLSANGGIFKPQGKAINDNAADDIKVLVVGNPANTNALIAQAAAPDVPAERFTAMTRLDHNRAISQLAAKTGAAVSDIKKLTIWGNHSATQYPDIFHAEVAGKNAAELVNDEAWLADTFIPTVAKRGAAIIEARGASSAASAANAAIDHVHTWVNGTAAGDWTSMGIPSDGSYGVPEGIISSFPVTTKDGKYEIVQGLEINDFSRARIDASVKELTEERDAVRELGLI

>Streptomyces_sp._PAMC_26508_b

MTRTPVNVTVTGAAGQIGYALLFRIASGHLLGPDVPVNLRLLEIPQGLKAAEGTAMELDDCAFPLLRNIEITDNPDVGFAGANVALLVGARPRTKGMERGDLLSANGGIFKPQGKAINDNAADDIKVLVVGNPANTNALIAQAAAPDVPAERFTAMTRLDHNRAISQLAARTGAAVSDIKRLTIWGNHSATQYPDIFHAEIAGKNAAEVVNDEVWLADTFIPTVAKRGAAIIEARGASSAASAANAAIDHVHTWVNGTAEGDWTSMGIPSDGSYGVPEGIISSFPVTTKDGKYEIVQGLDINEFSRARIDASVQELTEERDAVRELGLI

>Streptomyces_malaysiense_b

MTRTPVNVTVTGAAGQIGYALLFRIASGQLLGADVPVRLRLLEITPALKAAEGTAMELDDCAFPLLQGIDISDDPNVAFDGANVALLVGARPRTKGMERGDLLEANGGIFKPQGKAINDHAADDIKVLVVGNPANTNALIAQAAAPDVPAERFTAMTRLDHNRALTQLAKKTGSTVADIKRLTIWGNHSATQYPDIFHATVGGKNAAEVVSDEKWLAEDFIPTVAKRGAAIIEARGASSAASAANAAIDHVHTWVNGTADGDWTSMGIPSDGSYGVPEGLISSFPVTTKDGKYEIVQGLEINEFSRARIDASVKELSEEREAVRALGLI

>Actinomyces_sp._HMSC065F11_b

MTEARIVTVTGAAGNIGYALLFRIASGAVFGKDVPVKLNLLEITPALKAAEGTAMELDDAAFPLLEGVDIFDDANKAFAGTNAAFLVGARPRTKGMERADLLEANGGIFGPQGKAINDNAADDVRVLVVGNPANTNALIASAAAPDVPTSRFTAMMRLDHNRAMSQLSHKLQVPSRLIKDMVVWGNHSADQFPDVTYARVDGKSAKDLVDEAWLDEYFVPTVAKRGAAIIEARGASSAASAASAAIDHMHDWVNGTPEGDWVTSGSVSDGSYGVPEGLIYGFPVTSTGGEWEIVQGLELDDRQKAGIARNVKALEEERDAVRALGFVK

>Pseudonocardia_autotrophica_b

MSATPVTVTVTGAAGQIGYALLFRIASGQLLGSDTPVKLNLLEIPQAVKAAEGTAMELDDSAFGLLRGIDIYDDAKAAFDGANVALLVGARPRTKGMERGDLLEANGGIFGPQGKAINDGAASDIRVLAVGNPANTNALIAQAAAPDVPADRFTAMTRLDHNRALAQLAKKLGVGLDEITKLTIWGNHSATQYPDLFHAEVGGKIAAEQVEREWLENDFIPTVAKRGAAIIEARGASSAASAANAAIDHVYDWVNGTPAGDWTSAAIPSDGSYGVPEGLISSFPVTSENGEWKIVQGLEIDEFSRSKIDASVAELGEERDAVQKLGLL

>Jiangella_alkaliphila_b

MVQAPVNVTVTGAAGQIGYALLFRVASGQLLGADTPVRLKLLEIPPAVKAAEGTAMELDDCAFPLLSGIDIYDDPRQAFDGVNVALLVGARPRTKGMERGDLLEANGGIFKPQGEAINAGAASDVRVLVVGNPANTNALIARSHAPDVPADRFTAMTRLDHNRALSQLSAKLGVGVADISKLTIWGNHSATQYPDIFHAEIAGKPASELVDESWLSDDFIPTVAKRGAAIIEARGASSAASAANAAIDHVYDWVNGTPEGDWTSAAIVSDGSYGVAEGLISSFPVVSRDGAWEIVQGLEVSDFSRARIDASVQELTEERDAVAALGLI

>Allokutzneria_albata_b

MTQAPVNVTVTGAAGQIGYALLFRIASGQLLGADTPVKLRLLEIPQAVKAAEGTAMELDDCAFPLLKGIDITDDAKKAFDGTNVALLVGARPRTKGMERGDLLAANGGIFKPQGEAINAGAADDVRVLVVGNPANTNALIAQAHAPDVPAERFTAMTRLDHNRALSQLSAKLGVAVTDIKKLTIWGNHSATQYPDLFNAEVGGKVAAEQVEQEWLKDTFIPTVAKRGAAIIEARGASSAASAANAAIDHVYDWVNGTPEGDWTSAAVVSDGSYGVPEGLISSFPVVSRNGRYEIVQGLEIGEFSRERIDASVQELIEERDAVKQLGLI

>Frankia_sp._CpI1-S_b

MSSTPVNVTVTGAAGQIGYALLFRIASGQLLGADTPVKLRLLEIPQAVRAAEGTALELEDSAFPLLAGVDVFDDAKRAFEGTNVALLVGARPRTKGMERGDLLSANGGIFKPQGEAINSGAAEDIRVLVVGNPANTNALIAQTHAPDVPAERFTAMTRLDHNRAIAQLAKKLGVPSAEIRKITIWGNHSATQYPDIFHAQVGGRSGAEAVGDQKWIAEEFIPRVAKRGAEIIEVRGASSAASAASAAIDHVYTWVNGTPEGDWTSAAIPSDGSYGVPEGLISSFPVTAAGGKFEIVQGLELDAFSREKIDASVRELAEEREAVRALGLI

>Mannheimia_succiniciproducens_MBEL55E_b

MKVAVLGAAGGIGQALALLLKLQLPAGSSLSLYDVAPVTPGVAKDLSHIPTDVVVEGFAGTDPSEALKGADIVLISAGVARKPGMTRADLFGVNAGIIRSLTEKVAEQCPKACVGIITNPVNAMVAIAAEVLKKAGVYDKRKLFGITTLDILRAETFIAELKGLDPTRVTIPVIGGHSGVTILPLLSQVQNVEWSSEEEIIALTHRIQNAGTEVVEAKAGGGSATLSMAQAAARFALALVKASQGAKVVECAYVEGDGKYARFFAQPVRLGTEGVEEYLTLGKLSAFEEKALNAMLETLQGDIKSGEDFING

>Pasteurella_multocida_pm70_b

MKVAVLGAAGGIGQALALLLKLQLPAGSELSLYDIAPVTPGVAADVSHIPTAVKVQGFAGEDPTPALQGADVVLISAGVARKPGMDRSDLFNINAGIVRNLIEKVAQVCPKACVGIITNPVNTTVAIAAEVLKKAGVYDKRKLFGVTTLDIIRSETFVSELKNLEPARTTVPVIGGHSGVTILPLLSQVQYAEWNDAEIAPLTKRIQNAGTEVVEAKAGGGSATLSMAQAAARFALSLVKGLNGENVVECTYVEGDGKYARFFAQPVRLGKEGVEEILPLGSLSAFEQHALDAMLETLRADIELGEKFVNQ

>Pasteurella_dagmatis_ATCC_43325_b

MKVAVLGAAGGIGQALALLLKLQLPAGSELSLYDIAPVTPGVAADVSHIPTAVKVQGFAGEDPTLALQGADVVLISAGVARKPGMDRSDLFNINAGIVRNLIEKVAVVCPKACVGIITNPVNTTVAIAAEVLKKAGVYDKRKLFGVTTLDIIRSETFVSELKNLDPIRTIVPVIGGHSGVTILPLLSQVQYVEWKEEEIAPLTKRIQNAGTEVVEAKAGGGSATLSMAQAAARFALSLVKGLQGENVVECTYVEGCGKYARFFAQPVRLGREGVEEILPIGPLSAFEQQALDTMLETLRADIELGEKFVNQ

>Gallibacterium_anatis_UMN179_b

MKVAVLGAAGGIGQALSLLLKLQLPAGSDLALYDIAPVTPGVAVDVSHIPTAVTVKGFAGEDPTPALEGADVVLISAGVARKPGMDRSDLFNINAGIVKNLIEKVAATCPKACVGIITNPVNTTVAIAAEVLKKAGVYDKRKLFGVTTLDTLRSETFVAELKNVDVSKVQVPVIGGHSGVTILPLLSQVHYVEWKEEEIEPLTKRIQNAGTEVVNAKAGGGSATLSMAQAAARFALSLVRALNGEKVVECTYVEGNGEYARFFAQPVRLGKEGVEALLPIGPLSAFEKAAVEAMLPTLKADIELGENFVK

>Haemophilus_parainfluenzae_ATCC_33392_b

MKVAVLGAAGGIGQALALLLKLQLPAESELSLYDIAPVTPGVAKDVSHIPTAVKVEGFAGEDPTPALKGADVVLISAGVARKPGMDRSDLFNINAGIVRNLIEHIAKTCPKACVGIITNPVNTTVAIAAEVLKKAGVYDKRKLFGVTTLDVLRSETFVSELKGLNVSRTSVPVIGGHSGVTILPLLSQVQYAEWKEEEIAPLTKRIQNAGTEVVEAKAGGGSATLSMAQAAARFARSLVKGLSGETVVECTYVEGDGKYARFFAQPVRLGKEGVEEILPIGTLSKFEQDALEAMLPTLRADIELGEKFING

>Haemophilus_haemolyticus_M21621_b

MKVAVLGAAGGIGQALALLLKLQLPAGSDLALYDIAPVTPGVAVDVSHIPTAVNVKGFSGEDPSPALEGADVVLISAGVARKPGMDRSDLFNINAGIVRGLIEKVAVTCPKACVGIITNPVNTTVAIAAEVLKKAGVYDKRKLFGVTTLDVLRSETFVAELKGLNVSRTSVPVIGGHSGVTILPLLSQVQYAEWNEDEIEPLTKRIQNAGTEVVNAKAGGGSATLSMAQAAARFARSLVKGLSGETVVECTYVEGDGKYARFFSQPVRLGKEGVEEILPIGLLSNFEQQALENMLPTLRADIELGEKFING

>Avibacterium_paragallinarum_JF4211_b

MKVAVLGAAGGIGQALALLLKLQLPAGSELSLYDIAPVTPGVAADVSHIPTAVKVQGFAGEDPTPALEGANVVLISAGVARKPGMDRSDLFNINAGIVRNLVEKVAQVCPKACIGIITNPVNTTVAIAAEVLKKAGVYDKRKLFGVTTLDVLRSETFVAELKNLDVSRTAVPVIGGHSGVTILPLLSQVQYAEWKEEEIAPLTKRIQNAGTEVVEAKAGGGSATLSMAQAAARFARSLVKGLSGETVVECSYVEGDGKYARFFAQPVRLGKEGVEEILPIGELSAFEQNALEAMLPTLRADIELGEKFINS

>Rodentibacter_myodis_b

MKVAVLGAAGGIGQALALLLKLQLPAETELSLYDIAPVTPGVAADVSHIPTAVKVKGFSGEDPTPALEGADVVLISAGVARKPGMDRSDLFNINAGIVRGLIEKVAATCPKACIGIITNPVNTTVAIAAEVLKKAGVYDKRKLFGVTTLDVLRSETFVAELKNLNVSRTTVPVIGGHSGVTILPLLSQVPYAEFNAEEIAPLTKRIQNAGTEVVEAKAGGGSATLSMAQAAARFARSLVKGLSGETVVECTYVEGDGKYARFFAQPVRLGKEGVEEILPIGPLSEFEQQALEAMLPTLRGDIELGEKFING

>Cosenzaea_myxofaciens_ATCC_19692_b

MKVAVLGAAGGIGQALALLLKNQLPAGSELSLYDIAPVTPGVAADLSHIPTDVTVKGFAGEDPSPALKGADVVLISAGVARKPGMDRSDLFNVNAGIVRNLIEKVAINCPKALIGIITNPVNTTVAIAAEVLKKAGVYDKKRLFGITTLDIIRANTFVAELKDKDPQKTNVPVIGGHSGVTILPLLSQVEGVSWTDEEVTALTKRIQNAGTEVVEAKAGGGSATLSMGQAAARFGLSLIRALNGEKEVIECTYVEGDGEYARFFAQPVRLGKNGIEEYLPIGKLSDYEKQSLNNMLDVLRKDIILGEEFINK

>Proteus_mirabilis_HI4320_b

MKVAVLGAAGGIGQALALLLKNQLPAGSELSLYDIAPVTPGVAADLSHIPTQVRVKGFAGEDPSPALKDADVVLISAGVARKPGMDRSDLFNVNAGIVRNLIEKVAQNCPKALIGIITNPVNTTVAIAAEVLKKAGVYDKKRLFGVTTLDIIRANTFVAELKGKDPQTTNVPVIGGHSGVTILPLLSQVAGVSFTDEEVAALTKRIQNAGTEVVEAKAGGGSATLSMGQAAARFGLSLIRALNGEKDVIECTYTEGDGEHARFFAQPVRLGKNGVEEYLPIGQLSDFEKQSLNGMLDVLKKDIILGEEFINK

>Morganella_morganii_b

MKVAVLGAAGGIGQALALLLKTQLPSGSDLTLYDIAPVTPGVAKDLSHIPTDVRITGFAGEDPTPALEGADIVLISAGVARKPGMDRSDLFNVNAGIVRNLVEKIAKTCPKALIGIITNPVNTTVAIAAEVLKKAGVYDKNRLFGVTTLDIIRSNTFVAELKGKKPQELEVPVIGGHSGVTILPLLSQIPGVSFSDAEIDSLTKRIQNAGTEVVEAKAGGGSATLSMGQAAARFGLSLVRAMQGESNVVECTYTEGDGKYARFFAQPVVLGKNGVEKRLDIGSLSAYEEKALNGMLDVLKADIALGEKFINS

>Photorhabdus_temperate_J3_b

MKVAVLGAAGGIGQALALLLKTQLPSGSELSLYDIAPVTPGVAADLSHIPTDVRIKGFAGEDATPALEGADVVLISAGVARKPGMDRSDLFNVNAGIVRNLVEQVAKTCPKALIGIITNPVNTTVAIAAEVLKKAGVYDKNRLFGVTTLDIIRSNTFVAELKGKKPQEIEVPVIGGHSGVTILPLLSQIPGVSFTDEELAALTKRIQNAGTEVVEAKAGGGSATLSMGQAAARLGLSLVRGLQGESNVVECAYVEGDGKYARFFAQPVRLGKNGVEERLEIGKLSDFEQKALEDMLDVLRKDIELGEKFINN

>Moellerella_wisconsensis_ATCC_35017_b

MKVAVLGAAGGIGQALALLLKNQLPAGSELSLYDIAPVTPGVAADLSHIPTDVKVIGFSGEDATPALHGADVVLISAGVARKPGMDRADLFNVNAGIVRNLVQQIATTCPKALIGIITNPVNTTVAIAAEVLKKAGVYDKNRLFGVTTLDIIRSNTFIAELKGKNPQTTEVPVIGGHSGVTILPLLSQVPGVSFTDDEIIALTKRIQNAGTEVVEAKAGGGSATLSMGQAAARFGLSLIRGLQGESNVIECTYTEGDGQHARFFAQPVRLGKNGVEERLPIGELSAFEQNALNSMLDVLKADIELGEKFING

>Shigella_sonnei_Ss046_b

MKVAVLGAAGGIGQALALLLKTQLPSGSELSLYDIAPVTPGVAVDLSHIPTAVKIKGFSGEDATPALEGADVVLISAGVARKPGMDRSDLFNVNAGIVKNLVQQVAKTCPKACIGIITNPVNTTVAIAAEVLKKAGVYDKNKLFGVTTLDIIRSNTFVAELKGKQPGEVEVPVIGGHSGVTILPLLSQVPGVSFTEQEVADLTKRIQNAGTEVVEAKAGGGSATLSMGQAAARFGLSLVRALQGEQGVVECAYVEGDGQYARFFSQPLLLGKNGVEERKSIGTLSAFEQNALEGMLDTLKKDIALGEEFVNK

>Klebsiella_pneumonia_subsp._pneumonia_ATCC_700721_b

MKVAVLGAAGGIGQALALLLKTQLPSGSELSLYDIAPVTPGVAVDLSHIPTDVKIKGFSGEDATPALEGADVVLISAGVARKPGMDRSDLFNVNAGIVKNLVQQIAKTCPQACIGIITNPVNTTVAIAAEVLKKAGVYDKNKLFGVTTLDIIRSNTFVAELKGKSATEVEVPVIGGHSGVTILPLLSQIPGVSFSDQEIADLTKRIQNAGTEVVEAKAGGGSATLSMGQAAARFGLSLVRAMQGEKGVVECAYVEGDGHYARFFSQPLLLGKNGVEERQSIGKLSAFEQQALEGMLDTLKKDIALGEDFVNK

>Enterobacter_hormaechei_ATCC_49162_b

MKVAVLGAAGGIGQALALLLKTQLPSGSELSLYDIAPVTPGVAVDLSHIPTAVKIKGFSGEDARPALQGADVVLISAGVARKPGMDRSDLFNVNAGIVKNLVQQIAETCPKACIGIITNPVNTTVAIAAEVLKKAGVYDKNKLFGVTTLDIIRSNTFVAELKGKQPTEVEVPVIGGHSGVTILPLLSQIPGVSFTEQEVADLTKRIQNAGTEVVEAKAGGGSATLSMGQAAARFGLSLVRALQGEKDVVECAYVEGDGEHARFFSQPLLLGKNGVEERKSIGTLSAFEQNAMEGMLDTLKKDITLGEEFVNK

>Escherichia_fergusonii_ATCC_35469_b

MKVAVLGAAGGIGQALALLLKTQLPSGSELSLYDIAPVTPGVAVDLSHIPTAVKIKGFAGEDATPALEGADVVLISAGVARKPGMDRSDLFNVNAGIVKNLVQQIATTCPKACIGIITNPVNTTVAIAAEVLKKAGVYDKNKLFGVTTLDIIRSNTFVAELKGKQPDEIEVPVIGGHSGVTILPLLSQIPGVSFTEQEVADLTKRIQNAGTEVVEAKAGGGSATLSMGQAAARFGLSLVRALQGEKGVVECAYVEGDGQYARFFSQPLLLGKNGVEERKSIGKLSAFEQNALEGMLDTLKKDIQLGEAFVNK

>Citrobacter_koseri_ATCC_BAA-895_b

MKVAVLGAAGGIGQALALLLKTQLPSGSELSLYDIAPVTPGVAVDLSHIPTAVKIKGFSGEDATPALEGADVVLISAGVARKPGMDRSDLFNVNAGIVKNLVQQIATTCPKACVGIITNPVNTTVAIAAEVLKKAGVYDKNKLFGVTTLDIIRSNTFVAELKGKLPTDVEVPVIGGHSGVTILPLLSQIPGVSFTEQEVADLTKRIQNAGTEVVEAKAGGGSATLSMGQAAARFGLSLVRALQGEKDVVECAYVEGDGQYARFFSQPLLLGKNGVEERKSIGKLSAFEQNALEGMLDTLKKDIALGEEFVNK

>Raoultella_planticola_b

MKVAVLGAAGGIGQALALLLKTQLPSGSELSLYDIAPVTPGVAVDLSHIPTDVKIKGFSGEDATPALVGADVVLISAGVARKPGMDRSDLFNVNAGIVKNLVQQIAKTCPQACIGIITNPVNTTVAIAAEVLKKAGVYDKNKLFGVTTLDIIRSNTFVAELKGKSASDVEVPVIGGHSGVTILPLLSQIAGVSFSEQEVADLTKRIQNAGTEVVEAKAGGGSATLSMGQAAARFGLSLVRAMQGEKGVVECAYVEGDGQYARFFSQPLLLGKNGIEERQSFGKLSAFEQQALEGMLDTLKKDIALGEDFVNK

>Kluyvera_georgiana_ATCC_51603_b

MKVAVLGAAGGIGQALALLLKTQLPSGSELSLYDIAPVTPGVAVDLSHIPTDVKIKGFSGEDATPALEGADVVLISAGVARKPGMDRSDLFNVNAGIVKNLVQQIAKTAPKACIGIITNPVNTTVAIAAEVLKKAGVYDKNKLFGVTTLDIIRSNTFVAELKGKSAGDVEVPVIGGHSGVTILPLLSQISGVSFSDQEVNDLTKRIQNAGTEVVEAKAGGGSATLSMGQAAARFGLSLVRALQGEKGVVECAYVEGDGQYARFFSQPLLLGKNGIEERQSIGKLSAYEQQALEGMLDTLRKDIQLGEDFINK

>Serratia_odorifera_DSM_4582_b

MKVAVLGAAGGIGQALALLLKTQLPSGSELSLYDIAPVTPGVAVDLSHIPTAVNIKGFAGEDAKPALQGADVVLISAGVARKPGMDRSDLFNVNAGIVRNLIQQVAETCPKACIGIITNPVNTTVAIAAEVLKKAGVYDKNKLFGVTSLDIIRSNTFVAELKGKKPEELNVPVIGGHSGVTILPLLSQIPGVTFSEQEVADLTKRIQNAGTEVVEAKAGGGSATLSMGQAAARFGLSLVRALQGEQGVVECAYVEGDGKYARFFAQPLLLGKNGVEERKDIGTLSAFEQKALEEMLDTLRKDIELGETFVNK

>Serratia_fonticola_AU-AP2C_b

MKVAVLGAAGGIGQALALLLKTQLPSGSELSLYDIAPVTPGVAVDLSHIPTAVKIKGFSGEDATPALHGADVVLISAGVARKPGMDRSDLFNVNAGIVRNLIEQVAKTCPKACIGIITNPVNTTVAIAAEVLKKAGVYDKNKLFGITSLDIIRSNTFVAELKGKQPEELNVPVIGGHSGVTILPLLSQIPGVSFSDQEVADLTKRIQNAGTEVVEAKAGGGSATLSMGQAAARFGLSLVRALSGEKGVVECAYVEGDGKYARFFAQPLLLGKNGIEERKDIGTLSAFEQKALSEMLDVLHKDIELGEKFINN

>Hafnia_alvei_ATCC_51873_b

MKVAVLGAAGGIGQALALLLKTQLPSGSELSLYDIAPVTPGVAVDLSHIPTAVKIKGFSGEDARPALEGADIVLISAGVARKPGMDRSDLFNVNAGIVRNLIEQVASTCPKACIGIITNPVNTTVAIAAEVLKKAGVYNKDKLFGISTLDVIRSNTFVAELKGKNPAEVEVPVIGGHSGVTILPLLSQIPGVTFSEHEVADLTKRIQNAGTEVVEAKAGGGSATLSMGQAAARFGLSLVRAMQGESNVVECAYVEGDGKYARFFAQPLLLGKEGIVERKSIGSLSAFEQKALEGMLEVLHKDIELGENFVNK

>Photobacterium_phosphoreum_ANT-2200_b

MKVAVIGAAGGIGQALALLLKNRLPAGSDLALYDIAPVTPGVAADLSHIPTPVAIKGYCGDDPTPALEGADVVLISAGVARKPGMDRSDLFNVNAGIVKSLAEKIAVVCPQACVGIITNPVNTTVAIAADVLKKAGVYDKRKLFGITTLDVLRSETFVAELKGVSPCDIRVPVIGGHSGVTILPLLSQVEGVSFSEDEVKALTPRIQNAGTEVVEAKAGGGSATLSMGQAACRFGLAMVRALQGEQGVVECAYVEGDGKHTRFFAQPVLLGKDGIEEVMDYGTLSAFEQNALESMLDTLRADITLGEEFAAK

>Grimontia_hollisae_CIP_101886_b

MKVAVIGAAGGIGQALALLLKNRLPAGSDLALYDIAPVTPGVAVDLSHIPTPVSIKGYCGEDPTPALEGADVVLISAGVARKPGMDRSDLFNVNAGIVRSLSEKIADVCPKALIGIITNPVNTTVAIAAEVLKAKGVYDKKRLFGVTTLDIIRSETFVAELKGKNPGDVRVPVIGGHSGVTILPLLSQVEGVEFTEEEVAALTPRIQNAGTEVVEAKAGGGSATLSMGQAACRFGLALVRAAQGEQGIVECAYVEGDGKYARFFAQPVRLGKNGVEEILDHGPLSAFEQNALDSMLDTLRKDIALGEEFVK

>Vibrio_orientalis_CIP_102891_b

MKVAVIGAAGGIGQALALLLKNRLPAGSDLALYDIAPVTPGVAADLSHIPTPVSIKGYAGEDPSPALEGADVVLISAGVARKPGMDRADLFNVNAGIVKSLAQKIADVCPKALVGIITNPVNTTVPIAAEVLKKAGVYDKRRLFGVTTLDVIRSETFVADLKDKDPGDVRVPVIGGHSGVTILPLLSQVEGVEFTAEEVEALTKRIQNAGTEVVEAKAGGGSATLSMGQAACRFGLALVKAAQGEEVVEYAYVEGDGEHAPFFAQPVKLGKDGVEEVLSYGKLSDFEKSALDGMLETLNGDIQTGVDFVNN

>Vibrio_tubiashii_ATCC_19109_b

MKVAVIGAAGGIGQALALLLKNRLPAGSDLALYDIAPVTPGVAADLSHIPTPVSIKGYAGEDPTPALEGADVVLISAGVARKPGMDRADLFNVNAGIVKSLAQRIADVCPKALVGIITNPVNTTVPIAAEVLKQAGVYDKRRLFGVTTLDVIRSETFVADLKDKDPGDVRVPVIGGHSGVTILPLLSQVEGVEFTDEEVAALTKRIQNAGTEVVEAKAGGGSATLSMGQAACRFGLALVKALQGEDVIEYAYVEGDGEHAPFFAQPVKLGKEGVEEVLSYGELSDFEKSALDGMLETLNGDIQTGVDFVK

>Vibrio_parahaemolyticus_serotype_O3:K6_RIMD_2210633_b

MKVAVLGAAGGIGQALALLLKTQLPSGSELSLYDIAPVTPGVAVDLSHIPTAVKIKGFSGEDARPALEGADIVLISAGVARKPGMDRSDLFNVNAGIVRNLIEQVASTCPKACIGIITNPVNTTVAIAAEVLKKAGVYNKDKLFGISTLDVIRSNTFVAELKGKNPAEVEVPVIGGHSGVTILPLLSQIPGVTFSEHEVADLTKRIQNAGTEVVEAKAGGGSATLSMGQAAARFGLSLVRAMQGESNVVECAYVEGDGKYARFFAQPLLLGKEGIVERKSIGSLSAFEQKALEGMLEVLHKDIELGENFVNK

>Vibrio_caribbeanicus_ATCC_BAA-2122_b

MKVAVIGAAGGIGQALALLLKNHLPAGSDLALYDIAPVTPGVAADLSHIPTPVSIKGYSGEDPTPALEGADVVLISAGVARKPGMDRADLFNVNAGIVKSLAEKIAVVCPAACVGIITNPVNTTVPIAAEVLKRAGVYDKRKLFGVTTLDVIRSETFVAELKGKDPLQVKVPVIGGHSGVTILPLLSQVEGVEFSSEEIESLTKRIQNAGTEVVEAKAGGGSATLSMGQAACRFGLALVRALQGEQGVVECAYVEGDSEHAPYFAQPVKLGKDGVEEVLSYGKLSEYEQAALNGMLDTLNNDINIGVDFAK

>Vibrio_fischeri_ATCC_700601_b

MKVAVIGAAGGIGQALALLLKNRLPAGSDLALYDIAPVTPGVAADLSHIPTPVSIKGYCGEDPTPALEGADVVLISAGVARKPGMDRSDLFNINAGIVKSLTEKIAVTCPKACIGIITNPVNTTVAIAAEVLKKAGVYDKNKLFGVTTLDVIRSETFVAELKDKDPGEIRVPVIGGHSGVTILPLLSQVQGVEFTAEEVAALTPRIQNAGTEVVEAKAGGGSATLSMGQAACRFGLSLVKALSGEEGVVECAYVEGNGEHARFFAQPILLGKNGVEEIQSYGELSAFEQEALESMLDTLRGDIKIGEEFVQ

>Pasteurella_bettyae_CCUG_2042_b

MKVAVLGAAGGIGQALALLLKLQLPAGSALSLYDVAPVTPGVAKDISHIPTEVAVEGFAGTDPSDALKGADIVLISAGVARKPGMTRADLFDVNANIIRSLIEKVAEVCPKACVAIITNPVNATVAIAAEVLKKAGVYDKRKLFGVTTLDTLRSETFIGELKGLDPTRVTIPVIGGHSGVTILPLLSQVQNVEWSSAEEIETLTKRIQNAGTEVVEAKAGGGSATLSMAQAAARFCLSLVKGLNGAKVVECSYVEGDGKYARFFAQPVRLGKEGVEEFLPLGKLSAFEEEALKGMLETLHADIASGEKFIKG

>Gallibacterium_salpingitidis_b

MKVAVLGAAGGIGQALSLLLKIQLPAGSDLALYDIAPVTPGVAVDVSHIPTAVNVKGFAGEDPTPALEGADVVLISAGVARKPGMDRSDLFNINAGIVKNLIEKVAATCPKACVGIITNPVNTTVAIAAEVLKKAGVYDKRKLFGVTTLDTLRSETFVAELKNVDVSKVQVPVIGGHSGVTILPLLSQVHYVSWKDEEIEPLTKRIQNAGTEVVNAKAGGGSATLSMAQAAARFALSLVRALNGEKVVECTYVEGDGKYARFFAQPVRLGKEGVEELLPIGPLSAFEQAAVEAMLPTLKADIELGENFVK

>Haemophilus_parainfluenzae_b

MKVAVLGAAGGIGQALALLLKLQLPAESELSLYDIAPVTPGVAKDVSHIPTAVKVEGFAGEDPTPALKGADVVLISAGVARKPGMDRSDLFNINAGIVRNLIEHIAKTCPKACVGIITNPVNTTVAIAAEVLKKAGVYDKRKLFGVTTLDVLRSETFVSELKGLNVSRTSVPVIGGHSGVTILPLLSQVQYAEWKEEEIAPLTKRIQNAGTEVVEAKAGGGSATLSMAQAAARFARSLVKGLSGETVVECTYVEGDGKYARFFAQPVRLGKEGVEEILPIGTLSKFEQDALEAMLPTLRADIELGEKFING

>Haemophilus_aegyptius_ATCC_11116_b

MKVAVLGAAGGIGQALALLLKLQLPAGSDLALYDIAPVTPGVAVDVSHIPTAVNVKGFSGEDPTPALEGADVVLISAGVARKPGMDRSDLFNINAGIVRGLIEKVATTCPKACVGIITNPVNTTVAIAAEVLKKAGVYDKRKLFGVTTLDVLRSETFVAELKGLNVSRTSVPVIGGHSGVTILPLLSQVQYAEWNEDEIEPLTKRIQNAGTEVVNAKAGGGSATLSMAQAAVRFARSLVKGLSGETVVECTYVEGDGKYARFFSQPVRLGKEGVEEILPIGPLSNFEQQALENMLPTLRADIELGEKFING

>Haemophilus_somnus_129Pt_b

MKIAVLGAAGGIGQALALLLKLQLPAGSELSLYDIAPVTPGVAADVSHIPTAVKIQGFAGEDPTPALENADVVLISAGVARKPGMDRSDLFNINAGIVKNLIEKVAKTCPKACVGIITNPVNTTVAIAAEVLKKAGVYDKRKLFGVTTLDVLRSETFVAELKGLNVSRIAVPVIGGHSGVTILPLLSQVQYAEWEEDEIAPLTKRIQNAGTEVVEAKAGGGSATLSMAQAAARFALSLVQGLSGETVVECTYVEGDGKYARFFAQPVRLGKEGVEEILPVGTLSAFEQKALEDMLPTLRADIELGEKFVNN

>Rodentibacter_heylii_b

MKVAVLGAAGGIGQALALLLKLQLPAGSELSLYDIAPVTPGVAVDVSHIPTAVDVKGFSGEDPSAALQGADVVLISAGVARKPGMDRSDLFNINAGIVRNLVEKVAQVCPKACIGIITNPVNTTVAIAAEVLKKAGVYDKRKLFGVTTLDVLRSETFVAELKNLNVSRTAVPVIGGHSGVTILPLLSQVQYVQWNDDEIEPLTKRIQNAGTEVVEAKAGGGSATLSMAQAAARFARSLVKALSGETVVECTYVEGDGKYARFFAQPVRLGREGVEEILPIGILSQFEQKALEDMLPTLRADISLGEKFING

>Proteus_hauseri_ATCC_700826_b

MKVAVLGAAGGIGQALALLLKNQLPAGSELSLYDIAPVTPGVAADLSHIPTQVTVKGFAGEDPSPALKGADVVLISAGVARKPGMDRSDLFNVNAGIVRNLIEKVAQNCPKALIGIITNPVNTTVAIAAEVLKKAGVYDKRRLFGITTLDIIRANTFVAELKGKDPQKTNVPVIGGHSGVTILPLLSQVADVSLTNDEIAALTHRIQNAGTEVVEAKAGGGSATLSMGQAAARFGLSLIRALNGEKNVIECTYTEGDGEYARFFAQPILLGKNGVEEYLSIGKLSDFEKQALNGMLDVLKKDIILGEEFINK

>Proteus_vulgaris_b

MKVAVLGAAGGIGQALALLLKNQLPAGSELSLYDIAPVTPGVAADLSHIPTQVTVKGFAGEDPSPALKGADVVLISAGVARKPGMDRSDLFNVNAGIVRNLIEKVAQNCPKALIGIITNPVNTTVAIAAEVLKKAGVYDKKRLFGITTLDIIRANTFVAELKGQDPQKTNVPVIGGHSGVTILPLLSQVDGVSFTDDEVSALTKRIQNAGTEVVEAKAGGGSATLSMGQAAARFGLSLIRALNGEKNVIECTYTEGDGEYARFFAQPILLGKNGVEEYLSIGKLSDFEKQSLNGMLDVLKKDIILGEEFINK

>Morganella_psychrotolerans_b

MKVAVLGAAGGIGQALALLLKTQLPSGSDLALYDIAPVTPGVAKDLSHIPTDVRITGFAGEDPTPALEGADIVLISAGVARKPGMDRSDLFNINAGIVRNLVEKVAKTCPKALIGIITNPVNTTVAIAAEVLKKAGVYDKNRLFGVTTLDIIRSNTFVAELKGKKPQELEVPVIGGHSGVTILPLLSQIPGVSFSDAEIDSLTRRIQNAGTEVVEAKAGGGSATLSMGQAATRFGLSLVRAMQGESNVVECTYTEGDGKYARFFAQPVVLGKNGVEKRLDIGSLSAYEEKALNGMLDVLKADIALGEKFINS

>Photorhabdus_luminescens_b

MKVAVLGAAGGIGQALALLLKTQLPSGSELSLYDIAPVTPGVAVDLSHIPTEVKIKGFAGEDATPALEGADVVLISAGVARKPGMDRSDLFNVNAGIVRNLVEQVAKTCPKALIGIITNPVNTTVAIAAEVLKKAGVYDKNRLFGVTTLDVIRSNTFIAELKGKKPQEIEVPVIGGHSGVTILPLLSQIPDVSFTDEELVSLTKRIQNAGTEVVEAKAGGGSATLSMGQAAARLGLSLVRGLQGESDVVECAYVEGDGEYARFFAQPIRLGKNGVEERLDIGQLSDFEQKALEGMLDVLRKDIELGEKFINN

>Shigella_sp._fc130_b

MKVAVLGAAGGIGQALALLLKNQLPAGSELSLYDIAPVTPGVAADLSHIPTQVTVKGFAGEDPSPALKGADVVLISAGVARKPGMDRSDLFNVNAGIVRNLIEKVAQNCPKALIGIITNPVNTTVAIAAEVLKKAGVYDKKRLFGITTLDIIRANTFVAELKGKDPQKTNVPVIGGHSGVTILPLLSQVDGVSFTDDEVAALTKRIQNAGTEVVEAKAGGGSATLSMGQAAARFGLSLIRALNGEKNVIECTYTEGDGEYARFFAQPILLGKNGVEEYLSIGKLSDFEMQSLNGMLDVLKKDIILGEEFINK

>Enterobacter_cloacae_b

MKVAVLGAAGGIGQALALLLKTQLPSGSELSLYDIAPVTPGVAVDLSHIPTDVKIKGFSGEDATPALEGADVVLISAGVARKPGMDRSDLFNVNAGIVKNLVQQIAKTCPQACIGIITNPVNTTVAIAAEVLKKAGVYDKNKLFGVTTLDIIRSNTFVAELKGKSATEVEVPVIGGHSGVTILPLLSQIPGVSFSEQEVADLTKRIQNAGTEVVEAKAGGGSATLSMGQAAARFGLSLVRAMQGEKGVVECAYVEGDGQYARFFSQPLLLGKNGVEERQSIGKLSAFEQQALEGMLDTLKKDIALGEDFVNK

>Klebsiella_oxytoca_b

MKVAVLGAAGGIGQALALLLKTQLPSGSELSLYDIAPVTPGVAVDLSHIPTDVKIKGFSGEDATPALEGADVVLISAGVARKPGMDRSDLFNVNAGIVKNLVQQIAKTCPQACVGVITNPVNTTVAIAAEVLKKAGVYDKNKLFGVTTLDIIRSNTFVAELKGKSSSEVEVPVIGGHSGVTILPLLSQIPGVSFSEQEVADLTKRIQNAGTEVVEAKAGGGSATLSMGQAAARFGLSLVRAMQGEKGVVECAYVEGDGQYARFFSQPLLLGKNGVEQRQSIGTLSAFEQQALEGMLDTLKKDIALGEDFVNK

>Klebsiella_pneumoniae_subsp._pneumoniae_ATCC_700721_b

MKVAVLGAAGGIGQALALLLKTQLPSGSELSLYDIAPVTPGVAVDLSHIPTDVKIKGFSGEDATPALEGADVVLISAGVARKPGMDRSDLFNVNAGIVKNLVQQIAKTCPQACIGIITNPVNTTVAIAAEVLKKAGVYDKNKLFGVTTLDIIRSNTFVAELKGKSATEVEVPVIGGHSGVTILPLLSQIPGVSFSDQEIADLTKRIQNAGTEVVEAKAGGGSATLSMGQAAARFGLSLVRAMQGEKGVVECAYVEGDGHYARFFSQPLLLGKNGVEERQSIGKLSAFEQQALEGMLDTLKKDIALGEDFVNK

>Citrobacter_sp._50677481_b

MKVAVLGAAGGIGQALALLLKTQLPSGSELSLYDIAPVTPGVAVDLSHIPTAVKIKGFSGEDATPALEGADVVLISAGVARKPGMDRSDLFNVNAGIVKNLVQQIATTCPKACIGIITNPVNTTVAIAAEVLKKAGVYDKNKLFGVTTLDIIRSNTFVAELKGKLPTDVEVPVIGGHSGVTILPLLSQIPGVSFTEQEVADLTKRIQNAGTEVVEAKAGGGSATLSMGQAAARFGLSLVRALQGEKGVVECAYVEGDGQYARFFSQPLLLGKNGVEERQSIGKLSAFEQNALEGMLDTLKKDIQLGEEFVNK

>Salmonella_enterica_subsp._enterica_serovar_Rubislaw_A4-653_b

MKVAVLGAAGGIGQALALLLKNQLPSGSELSLYDIAPVTPGVAVDLSHIPTAVKIKGFSGEDATPALEGADVVLISAGVARKPGMDRSDLFNVNAGIVKNLVQQIAKTCPKACVGIITNPVNTTVAIAAEVLKKAGVYDKNKLFGVTTLDIIRSNTFVAELKGKLPTEVEVPVIGGHSGVTILPLLSQIPGVSFTEQEAAELTKRIQNAGTEVVEAKAGGGSATLSMGQAAARFGLSLVRALQGEKGVVECAYVEGDGQYARFFSQPLLLGKNGVEERKSIGTLSAFEQHSLDAMLDTLKKDIQLGEDFINK

>Photobacterium_halotolerans_b

MKVAVIGAAGGIGQALALLLKNELPAGSDLALYDIAPVTPGVAADLSHIPTPVSIKGYAGEDPTPALEGADVVLISAGVARKPGMDRADLFNVNAGIVKSLAEKIAVVCPNACIGIITNPVNTTVAIAADVLKKAGVYDKRKLFGVTTLDIIRSETFVGELKGLNPREVNVPVIGGHSGVTILPLLSQVEGVEFTEEEVKALTPRIQNAGTEVVEAKAGGGSATLSMGQAACRFGLALVRALQGEQNVVECAYVEGNGKHARFFAQPVRLGKDGVEEVLDYGSLSAFEQNALDSMLDTLKGDIALGEEFAAK

>Vibrio_orientalis_CIP102891_b

MKVAVIGAAGGIGQALALLLKNRLPAGSDLALYDIAPVTPGVAADLSHIPTPVSIKGYAGEDPSPALEGADVVLISAGVARKPGMDRADLFNVNAGIVKSLAQKIADVCPKALVGIITNPVNTTVPIAAEVLKKAGVYDKRRLFGVTTLDVIRSETFVADLKDKDPGDVRVPVIGGHSGVTILPLLSQVEGVEFTAEEVEALTKRIQNAGTEVVEAKAGGGSATLSMGQAACRFGLALVKAAQGEEVVEYAYVEGDGEHAPFFAQPVKLGKDGVEEVLSYGKLSDFEKSALDGMLETLNGDIQTGVDFVNN

>Mycobacterium_tuberculosis_b

MKVAVLGAAGGIGQALALLLKTQLPSGSDLTLYDIAPVTPGVAKDLSHIPTDVRITGFAGEDPTPALEGADIVLISAGVARKPGMDRSDLFNVNAGIVRNLVEKIAKTCPKALIGIITNPVNTTVAIAAEVLKKAGVYDKNRLFGVTTLDIIRSNTFVAELKGKKPQELEVPVIGGHSGVTILPLLSQIPGVSFSDAEIDSLTKRIQNAGTEVVEAKAGGGSATLSMGQAAARFGLSLVRAMQGESNVVECTYTEGDGKYARFFAQPVVLGKNGVEKRLDIGSLSAYEEKALNGMLDVLKADIALGEKFINS

>Streptococcus_pneumoniae_b

MKVAVLGAAGGIGQALALLLKLQLPAGSDLALYDIAPVTPGVAVDVSHIPTAVNVKGFSGEDPTPALEGADVVLISAGVARKPGMDRSDLFNINAGIVRGLIEKVAVTCPKACVGIITNPVNTTVAIAAEVLKKAGVYDKRKLFGVTTLDVLRSETFVAELKGLNVSRTSVPVIGGHSGVTILPLLSQVQYAKWNEDEIEPLTKRIQNAGTEVVNAKAGGGSATLSMAQAAARFARSLVKGLSGETVVECTYVEGDGKYARFFSQPVRLGKEGVEEILPIGPLSNFEQQALENMLPTLRADIELGEKFING

>Saccharomyces_cerevisiae_fc

MPHSVTPSIEQDSLKIAILGAAGGIGQSLSLLLKAQLQYQLKESNRSVTHIHLALYDVNQEAINGVTADLSHIDTPISVSSHSPAGGIENCLHNASIVVIPAGVPRKPGMTRDDLFNVNAGIISQLGDSIAECCDLSKVFVLVISNPVNSLVPVMVSNILKNHPQSRNSGIERRIMGVTKLDIVRASTFLREINIESGLTPRVNSMPDVPVIGGHSGETIIPLFSQSNFLSRLNEDQLKYLIHRVQYGGDEVVKAKNGKGSATLSMAHAGYKCVVQFVSLLLGNIEQIHGTYYVPLKDANNFPIAPGADQLLPLVDGADYFAIPLTITTKGVSYVDYDIVNRMNDMERNQMLPICVSQLKKNIDKGLEFVASRSASS

>Zygosaccharomyces_bailii_CLIB_213_fc

MPHSVNEDIKITVVGAAGGIGQSLSLLLKTQLASLLPSHRHAHLALYDVNADAVRGVTADLSHIDTGVSVSGYEGDGIGGALEHADVVLIPAGVPRKPGMTREDLLAINAKIIKSLGASIAKYCDLSKVFVLLISNPINSLVPVLVKELEAHAPGLPVERRVFGITRLDAVRASTFLHEATVRAGSTPKSNTLPYVPVIGGHSGETIVPLFSLVPGASDLSKDTLKELVHRVQYGGDEVVKAKNGAGSATLSMAHAAYKVVESFIPLLTGSVPSIEGTFYVALQDSKGQPINASAQKLLSHINGLPYFAVPLRVTSAGVDEVDAGIIERMSLFERERLLAPCLGKLQGNISTGLSL

>Lachancea_thermotolerans_ATCC_56472_fc

MPHHTGAQPAVKVAVLGAGGGIGQTLSLLLKTGLAPASVAQQRRVHIALYDVNRDAVAGAATDLSHIDTPVSVSWHAPEPAGADALADPLQACLAGAQLVVIPAGVPRKPGMTRDDLFNINAQIVRTLAGGIARHCDLAHVFVLLISNPVNSLVPVLVETLTQHCGADAALAAQIPRRAFGLTQLDAVRASSFLHQALDCEPSETAVVPVVGGHSGNTILPLFSQARIEPARGSGAAAESRTSTKAGAKIGAKSGATAMPALDPDVRQRLVHRVQFGGDEVVRAKNGAGSATLSMAYAGAQVACKFAEMLLGARGEVRDTLYAKVDGHVPLDAITEHVGYFSVPLTVTAAQGAAHADTHVLEQMDAYEREQLWPACLAELRESVDRGLEWAHAHR

>Ashbya_gossypii_ATCC_10895_fc

MQASTYYVISNMPHTTADSSTQRVRVAVLGAAGGIGQPLSLLLKTQLAQVLGDANASLELALYDVAADALAGVAADLSHVNTPVEVSHHVPSSREDEEALREALTGASVVVIPAGVPRKPGMTRDDLININAGIIKTLAKGIAGACDLEKVFVLVISNPVNSLVPVMVRQLIRHAEAKQAPHAGVERRVFGVTQLDMVRASAFVRSLGELGNEVPSVPVIGGHSGETILPLFGPVQQRLQFSLEQRKKLTHRVQYGGDEIVAAKKGAGSATLSMAYAAYVVAERFTNLVLGNVSEIQETLYVSLYDRENKPIAEGAAELLSNIGETPYFAVPVKISADAGVVRVEHEVWQQLDDYDREHLLPACLEGLRKNIETGETLGSN

>Candida_albicans_SC5314_fc

MVKVAILGAAGGIGQPLSLLTKLNPNVDELALFDVVNVPGVGADLSHINSDSKTQSYLPKDKEDKTALAAALKGSDLVIIPAGVPRKPGMTRDDLFNINASIVQGLAEGIAANSPKAFVLVISNPVNSTVPIVAETLQAKGVYDPARLFGVTTLDIVRANTFISQLFLDQTKPSDFNINVVGGHSGETIVPLYSLGNSKQYYDILSEEQKKELIKRVQFGGDEVVQAKNGAGSATLSMAYAGYRLAESILAAVNGKTDIVECTFLNLDSSIKGASEARKLVKDLDFFSLPVQLGKNGITEVKYDILNQISDDEKKLLEVAIEQLQKNIEKGVSFAKK

>Debaryomyces_hansenii_ATCC_36239_fc

MVKVTVCGAAGGIGQPLSLLLKLNPKIDELSLFDVVNVPGVGADLSHICSNSSTSSHLPSSREDKSALAESLKGSDLVIIPAGVPRKPGMTRDDLFNINASIVRDLAQGIAENAPKAFVLIISNPVNSTVPIVAETLKKNGVYNPQRLFGVTTLDIVRANTFISQKYAKETKATDFNINVIGGHSGETIVPLYSIGNSKSYYDKLSEEDKKALINRVQFGGDEVVKAKDGAGSATLSMAYAGYKLAESILKALTSKGEEVVECTFLNLDDSIKGAAEAKKLVKNLDFFSLPVRLGANGIEEVKYDILNNISADEKKLLEVAIEQLSGNIDKGVAFIKK

>Clavispora_lusitaniae_ATCC_42720_fc

MVKVTVCGAAGGIGQPLSLLLKLNPQVSELSLFDVVNVPGVGADLSHINSGAVTKSFLPSSKEDTTALAGALKGSDLVVIPAGVPRKPGMTRDDLFNINASIVQSLAKGIAENAPHAFVLVISNPVNSTVPIVAETLKKYNVFNPSKLFGVTTLDIVRANTFISQLFPSDTKPTDFEVPVVGGHSGETIVPLYSLGAKSYYDKLSDEQKKELVHRVQFGGDEVVQAKNGAGSATLSMAYAGYRLAESLLKALSGSSVTECTFLYLDSSIKGADEAKKLVKGLDFFSLPSTLGKGGISSVDYTVLEKANSEEKKLLEVAIEQLKGNIAKGVSFGK

>Penicillium_rubens_ATCC_28089_fc

MVKAVVLGAAGGIGQPLSLLLKACPLVDDLALYDVVNTPGVAADLSHISSVAKITGYLPKDDGLKLALTGADVVVIPAGIPRKPGMTRDDLFKVNAGIVKGLVESIAEFAPKAFILVISNPVNSTVPIAAEVLKAAGVFDPKRLFGVTTLDVVRAETFTQEFSGVKNAADATVPVIGGHSGETIVPLFSKVSPSFQIPADRYDALVNRVQFGGDEVVKAKDGAGSATLSMAFAGFRFAEAVIKASKGEKGIVEPTFVYLPGVAGGDEIAKATGLDFFSTPVELGVNGAEKAINILEGVTEQEKKLLEACIKGLQGNIEKGVEFAKNSSPK

>Aspergillus_flavus_ATCC_200026_fc

MVKAAVLGASGGIGQPLSLLLKTCPLVEELALYDVVNTPGVAADLSHISSIAKISGFLPKDDGLKQALTGANIVVIPAGIPRKPGMTRDDLFKINAGIVRDLVKGIAEFCPKAFVLVISNPVNSTVPIAAEVLKAAGVFDPKRLFGVTTLDVVRAETFTQEFSGQKDPSAVQIPVVGGHSGETIVPLFSKTTPAIQIPEEKYDALIHRVQFGGDEVVQAKDGAGSATLSMAYAGYRFAESVIKASKGQTGIVEPTFVYLPGIPGGDEIVKATGVEFFSTLVTLGTNGAEKASNVLEGVTEKEKKLLEVCTKGLKGNIEKGIDFVKNPPPKL

>Aspergillus_oryzae_RIB_40_fc

MVKAAVLGASGGIGQPLSLLLKTCPLVEELALYDVVNTPGVAADLSHISSIAKISGFLPKDDGLKQALTGANIVVIPAGIPRKPGMTRDDLFKINAGIVRDLVKGIAEFCPKAFVLVISNPVNSTVPIAAEVLKAAGVFDPKRLFGVTTLDVVRAETFTQEFSGQKDPSAVQIPVVGGHSGETIVPLFSKTTPAIQIPEEKYDALIHRVQFGGDEVVQAKDGAGSATLSMAYAGYRFAESVIKASKGQTGIVEPTFVYLPGIPGGDEIVKATGVEFFSTLVTLGTNGAEKASNVLEGVTEKEKKLLEACTKGLKGNIEKGIDFVKNPPPK

>Aspergillus_niger_CBS_513.88_fc

MVKAAVLGASGGIGQPLSLLLKTSPLVDDLALYDVVNTPGVAADLSHISSVAKISGFLPKDDGLKHALTGADIVVIPAGIPRKPGMTRDDLFKINAGIVRDLVKGIAEYCPKAFVLIISNPVNSTVPIAAEVLKAAGVFDPKRLFGVTTLDVVRAETFTQEFSGHKDPSAVRIPVVGGHSGETIVPLFSKAAPAFQIPADKYDALVNRVQFGGDEVVKAKDGAGSATLSMAYAGFRFAQSVIKAAQGQSGIVEPTFVYLPGIAGGEDISKATGLEFFSTLVELGTNGAEKAINVLDGVTEKEKTLIEACTKGLKGNIEKGIEFVKNPPPK

>Neosartorya_fumigate_CEA10_fc

MVKAAVLGASGGIGQPLSLLLKACPLVDELALYDVVNTPGVAADLSHISSVAKVSGYLPKDDGLKNALTGTDIVVIPAGIPRKPGMTRDDLFKVNAGIVRDLVTGIAQYCPKAFVLIISNPVNSTVPIAAEVLKKQGVFDPKRLFGVTTLDIVRAETFTQEYSGQKDPSKVQIPVVGGHSGETIVPLFSKASPALDIPADKYDALVNRVQFGGDEVVKAKDGAGSATLSMAYAGFRFAEKVIRASQGQSGIVEPTYIYLRGVTGGEEIANETGVEFFSTLVELGRNGAEKAINILQGVTEQEKKLLEACTKGLKGNIEKGIEFVKNTPPK

>Talaromyces_marneffei_ATCC_18224_fc

MFAARQSFGAFQKRAFSASARQASKVTVLGAAGGIGQPLSLLMKLNPRVSQLALYDIRGGPGVAADLSHINTNSTVTGYDPTPSGLSEALKDAEIVLIPAGVPRKPGMTRDDLFNTNASIVRDLAKATADAAPNAKILVISNPVNSTVPIVAEVFKSKGVYNPKRLFGVTTLDVVRASRFISQVKTTDPANEEVPVVGGHSGVTIVPLLSQSNHADIEGETRDALVNRIQFGGDEVVKAKDGAGSATLSMAFAGARFAESLLKAAQGVQGVIEPTFVDSPLYKDQGIDFFASRVELGPDGVKEILPVGQVNAYEEKLLEACLADLKKNIKKGVDFVAQNP

>Ajellomyces_dermatitidis_ATCC_18188_fc

MVKAVVLGASGGIGQPLSLLLKASPLVDQLALYDVVNTPGVAADLSHISSIATVKGYLPKDDGLKDALTGADVVVIPAGIPRKPGMTRDDLFKVNAGIVQTLVKGIAEHSPKAFILIISNPVNSTVPIAAEVLKAAGVFDPKRLFGVTTLDVVRAETFTQEFTGQKDPSKTTIPVIGGHSGETIVPLFSQAKPAVNIPADRYDALVNRVQFGGDEVVKAKDGAGSATLSMAYAGFRFAESIIKASTGEKGIVEPTYVYLPGVEGGDAIKSKVGLDFFSIPVELGTSGAEKAQNILGDITEQEKKLLEVCIKGLKGNIEKGVEFAQSPPPKL

>Ajellomyces_capsulatus_H88_fc

MVKAVVLGASGGIGQPLSLLLKASLLVDELALYDVVNTPGVASDLSHISTIATVKGYLPKDDGLKDALTGADVVVIPAGIPRKPGMTRDDLFKVNAGIVQTLAKGIAEHSPKAFVLIISNPVNSTVPIAAEVLKAAGVFDPKRLFGVTTLDVVRAETFTQEFTGQKDPSKTTIPVIGGHSGETIVPLFSQAKPAVQIPTDRYDALVNRVQFGGDEVVKAKDGTGSATLSMAYAGFRFSESVIRASKGESGIVEPTYVYLPGIQGGDVIKNLVGLDYFSIPVELGKSGAERALDILGSITEQEKKLLEVCTKGLKGNIEKGIDFVKNPPTKL

>Paracoccidioides_brasiliensis_Pb03_fc

MVKAVVLGASGGIGQPLSLLLKASPLVDELALYDVVNTPGVAADLSHISTVATIKGYLPDNDGLKNALTGADIIVIPAGIPRKPGMTRDDLFKVNAGIVQTLVKGIAEFSPKAFILVISNPVNSTVPIAAEVLKAAGVFDPKRLFGVTTLDVVRAETFTQEFTGQKDPSKTSIPVIGGHSGETIVPLFSQAKPPVTIPADRYDGLVNRVQFGGDEVVKAKDGAGSATLSMAYAGFRFAESVIKASKGEKGIVEPTYIYLSGVEGGEAIKREVGLDFFSIPVELGASGAEKAHNILGGITEQEKKLLEACTKGLKGNIEKGIEFAKNPPPK

>Coccidioides_posadasii_RMSCC_757_fc

MKAAVLGASGGIGQPLSLLLKICPLVDELALFDVVNTPGVTADLSHISSVAKTSGFLKDDDGLKKALTGTDLVVIPAGIPRKPGMTRDDLFKINAGIVKELVQGVADYCPKAFVLIISNPVNSTVPIAAEVLKAAGVFDPKKLFGVTTLDVVRAETFSQEFNGQKNPADTVIPVIGGHSGETIVPMFSQAKPAFTVPADRYDALVNRIQFGGDEVVKAKSGAGSATLSMAWAGFRFAESVIKAVKGQKGIVESTFVYLPGVQGGDSIIKKTGLEFFSTPVELGASGAEKAIDILDGATEKEKQLLDVCYKGLKGNIEKGIDFVKNPPQKL

>Trichophyton_rubrum_ATCC_MYA-4607_fc

MVKAAVLGASGGIGQPLSLLLKICPLVDELVLYDVVNSPGVTADLSHISTAAKTSGYLPKDEGLKNALTGCELVLIPAGIPRKPGMTRDDLFTVNAGIVRDLVHGVAEFCPKAFVLIISNPVNSTVPIAAEVLKKAGVFDAKRLFGVTTLDILRAETFAQKYTGEKNPSDATIHVIGGHSGETIVPVYSLAKPVADIPESEYAEIIKRVQFGGDEVVKAKDSAGSATLSMAYAGYRFALSVMKAAKGEKGIVEPTFVHLSGINGGDVVAKETGLEYFSMPVELGPSGAENIVNILPNVNEREKALLEVCKSGLQGNIAKGISFVQNPPQKL

>Claviceps_purpurea_20.1_fc

MVKAVVAGASGGIGQPLSLLLKTSPLVDELALYDVVNTPGVAADLSHISSIAKVTGYLPANDGAKAAFKDADVVVIPAGIPRKPGMTRDDLFNINAGIVKGLIETIAEVAPKAFILVISNPVNSTVPISAEVLKAKGVFNAQRLFGVTTLDIVRAETFVAEIVGEKEPQKLTIPVVGGHSGETIVPLLSQASPAVNIPADKYDALIKRIQFGGDEVVEAKNGAGSATLSMAYAGYRFTEKLLRAVKGEKGLIEPSYVYLPGVPGGDSIAKETGCDFFSVPVELGPNGAEKATNPLGQLTDKEKGLLSKAVEGLNGNITKGINFAHNPPQK

>Gibberella_fujikuroi_CBS_195.34_fc

MVKAVVAGASGGIGQPLSLLLKTSPHIDELALYDVVNTPGVATDLSHISSRAKTTGYLPANDGAKAAFKDADIIVIPAGIPRKPGMTRDDLFNINAGIVKGLIEVVAEVAPKAFILVISNPVNSTVPIAAEVLKAKGVFNPQRLFGVTTLDIVRAETFVAEITGRANPQELTIPVIGGHSGETIVPLFSKATPAVDIPKDKYDALVNRVQFGGDEVVKAKDGAGSATLSMAYAGFRFAEKVLRAVKGEKGLVEPSYVYLPKVSEGGIPGGEAIAKVTGTDFFSVPIQLGPDGAEQANNPLEGITEQEKALLAKATEGLKGNISKGVSFVHNPPQK

>Colletotrichum_graminicola_M1.001_fc

MVKAVVAGASGGIGQPLSLLLKLSPLVDELALYDVVNTPGVAADLSHISSVAKTTGYLPKDDGAKAAFKDADIIVIPAGIPRKPGMTRDDLFNINAGIVKGLIEVAAEVAPKAFILVISNPVNSTVPISAEVLKAKGVFNPQRLFGVTTLDIVRAETFVAEIAGKSKPHELNVPVIGGHSGETIVPLFSQVQPSVSIPDDKYDALVNRVQFGGDEVVKAKDGAGSATLSMAYAGFRFAEKVLKAVKGEKGLVEPSYVYLPGVPGGKEIAEKTGVDFFSVPIELGPNGAEKAVDILGNITEKEKKLLEAAVAGLKGNIKKGVDFAHNPPQK

>Gloeophyllum_trabeum_ATCC_11539_fc

MVKAVVLGAAGGIGQPLSLLLKTNPLISELSLYDIVNTPGVAADLSHIATPAKVQGFLPADDGLKKALTGADIVVIPAGVPRKPGMTRDDLFKINAGIVRDLATGIATTAPKAFVLVISNPVNSTVPIVAEVFKKNGVFDPKRIFGVTTLDVVRASTFVSEILGDLSLAPSIKVPVVGGHSGVTIVPLLSQSSHPLPTSLKGDALDKLVNRIQFGGDEVVKAKDGAGSATLSMAYAGAEFAEKLLRAAKGEKGIVAPTYVHLSADKAGGDAIKKEIGKDLDYFSAPVELGPEGVVKINSLGKINEYEQKLIQAALPDLATNIEKGASFIDAPKL

>Puccinia_sorghi_fc

MPSLKVAVLGAAGGIGQPLSLLLKQNPHITELALFDVAPVVKGVAVDISHINTPSTVTGHVPDGDGLAAALTGANLVVIPAGVPRKPGMTRDDLFKINAGIVRDLATSMAKNCPKACILVISNPVNSTVPIVAEVFKKAGVFDAKKLFGVTTLDVVRASTFVSEVAGQSEKAHEYKIPVIGGHSGVTILPLLSQSKPPLPQSLLSDKSKIAELVKRIQFGGDEVVAAKDGAGSATLSMAFAGFRFAESLIKARLGQSGVIEMGYIYVADDKHISAHTEGLEYFSVPIELGAEGVGKLLPIGDINDHEKEMLKACVSELKESITKPIVSSLKQKEKKLLQ

>Batrachochytrium_dendrobatidis_JAM81_fc

MVKVAVLGAAGGIGQPLSLLLKGNMSVTELALFDIVNTPGVAADLSHINTPAKVTGYVGDEQLADALKGAHIVVIPAGVPRKPGMTRDDLFNINAGIVKNLAIGCAKNCPKAFIAVISNPVNSTVPIVAEVFKQHGVFDFRRIFGVTTLDVVRAASFVSEIAGTAAASTNVAVIGGHSGATILPILSALPHQFTDAQRDALVQRIQFGGDEVVKAKNGAGSATLSMAYAGARFVNSLLEASVHKKTGIKECTYIKTDVAAADGLEYFSTVVELGVDGVAVAHPLPNLSAHEKVLYTAAAAELKANIQKGVDFVAKASL

>Wuchereria_bancrofti_fc

MTAIGRISLVLEALTRNLQMCSHRLTSSAPKIALLGAAGGIGQPLGLLLKMNKHVAKLALYDIKDTPGVAADLSHIDTRAHVTGHTSPNELDEALQGADIVVIPAGLPRKPGMTRDDLFNTNASIVRDLSEAAAKNCPKAFIAIITNPVNSTVPIACEIFKKRGVFDPRRIFGVTTLDAVRSAAFVAGAKNLDAEETDIPVIGGHSGITIIPLLSQAKPLCKFSDDEVKKLTERIQNAGTEVVKAKAGAGSATLSMALAASKFVESLLRGLRGEKSIQCAYVASDACSGVDYFATPLEFGKNGVEKVLGMGKLSTYEQGLVDAAVPELKKNISKGLKFVSG

>Cyberlindnera_fabianii_fc

MKVTVCGAAGGIGQPLSLLLKLSPYVTQLALYDVVNAPGVGADLSHIDTDATISSHLPADNGLQHALEDADIVVIPAGVPRKPGMTRDDLFAINAGIVRDLAQAVASFAPKAHVLVISNPVNSTVPIFAEVLKKNGVFDARRLFGVTTLDLVRANTFISQLSEGKLDASKMDIPVIGGHSGETIVPLFSVGAPSDFYNSLSEEQRDALVHRVQYGGDEVVQAKKGAGSATLSMAYSGFKIAEKLMKAIAGETVDSKLGSSFVYLDDQIPGVSDAKALISNDNVHFISLPVTLGANGIESVDASILEKMSDHEKQLFAKALNTLEGSIAKGVDFIKGA

>Geotrichum_candidum_fc

pMTKVVVAGAAGGIGQPLSLLLKLSPYVTELALYDVVNTPGVAADLSHISTAAAVTGHLPKKSAADEPTSEDGLKEALTGAGLVIVPAGVPRKPGMTRDDLFNVNAGIVRTIADGVARHAPTAFLLIISNPVNSTVPVAVEVLRQYGVFDARRVFGVTTLDVVRAATFTSTVLSRAGEAARPDDVAVPVVGGHSGETIVPLLSQATVRGARVSGAGAKALAPGTPELAALVKRIQFGGDEVVQAKNGGGSATLSMAYAGYKFAEAVLRAAAVPQTASADAPITAETYVYIGPGSQVAGAAEVRAALSSGEAPLDYFSVPVALGPAGASHVLDPALLTRVSDYERGLLQVAAKGLAANIRKGLEFAAKNPVNFSKL

>Sphaerulina_musiva_SO2202_fc

MVKAVVAGASGGIGQPLSLLLKACPLVDHLSLYDVVNTPGVTADLSHISSIATIDGYLPDGGEGIKKAFKGADIVVIPAGIPRKPGMTRDDLFKINAGIVQGLVQGIAENCPDAFILIISNPVNSTVPIAAEVLKKAGVFNPKKLFGVTTLDVVRSETFVQSLTGEKDPSKTVIPVIGGHSGETIVPLFSQAKPAVKIPEDKLDALTHRVQFGGDEVVKAKDGAGSATLSMAYAGFRFAEKVIRAAKGESGIVEPTFVYLPGVAGGDEIVKETGLEFFSVPVELGKDGAAKAVNIVKAANEYEKKLLTKCYEGLKGNIEKGIDFVANPPAKS

>Penicillium_subrubescens_fc

MVKAVVLGASGGIGQPLSLLLKASPLVDELALYDVVNTPGVAADLSHISSPAKITGYLPKDDGLKNALTGADIVVIPAGIPRKPGMTRDDLFKINAGIVRDLAKGIAEFCPNAFTLVISNPVNSTVPIAAEVLKAAGVFNPQRLFGVTTLDVVRAETFTQEFSGLSDPSKATVPVIGGHSGETIVPLFSKVSPDFKIPADRYDALVNRVQFGGDEVVKAKDGAGSATLSMAFAGFRFAEAVIKAAKGEKGIVEPTFVYLPGVPGGDEIAKATGVDFFSTPVELGPNGAEKATNILGGVTDQEKTLLEACIKGLKGNIEKGVEFVKNSSK

>Coccidioides_posadasii_C735_fc

MKAAVLGASGGIGQPLSLLLKICPLVDELALFDVVNTPGVTADLSHISSVAKTSGFLKDDDGLKKALTGTDLVVIPAGIPRKPGMTRDDLFKINAGIVKELVQGVADYCPKAFVLIISNPVNSTVPIAAEVLKAAGVFDPKKLFGVTTLDVVRAETFSQEFNGQKNPADTVIPVIGGHSGETIVPMFSQAKPAFTVPADRYDALVNRIQFGGDEVVKAKSGAGSATLSMAWAGFRFAESVIKAVKGQKGIVESTFVYLPGVQGGDSIIKKTGLEFFSTPVELGASGAEKAIDILDGATEKEKQLLDVCYKGLKGNIEKGIDFVKNPPQK

>Cladophialophora_psammophila_CBS_110553_fc

MVKAVVLGASGGIGQPLSLLCKISPVIDELALFDVVNTPGVAADLSHISSVASIAGYVAAPGDFKGEKPETEEAKKAALTGADIVIIPAGVPRKPGMTRDDLFKINAGIVKGLIEACAKYCPKAYICVISNPVNSTVPIAAEVLKAAGVFDPRRLFGVTTLDVVRAQTFVGEIIGEKDPRKLTIPVIGGHSGNTIVPLFSQAKPPVNIPSDKLDALVNRVQFGGDEVVKAKDGAGSATLSMAYAGFRFAEALIKAAKGEKGVIEPTFVYLPGVPGGDAIAKATGCDYFSVPVELGPDGAEKAIDILGSANDYEKKHLEEAIKGLKTNIETGISFVKNPPK

>Rhynchosporium_agropyri_fc

MGIKAVVAGAAGGIGQPLSLLLKASPLITELALYDVVNTPGVAADLSHISSPAKVTGYLPKDDGAKAAMKDADIIVIPAGIPRKPGMTRDDLFKINAGIVKGLIEIIAEVAPKAYILIISNPVNSTVPIAAEVLKAKGVFDPKRLFGVTTLDVVRAETFVAEIVGEKTPSSLTIPVVGGHSGETIVPLFSQAGVKIPADKLEALVQRVQFGGDEVVKAKDGAGSATLSMAYAGFRFAEAVLKGLAGEKDIIEPSFVYLPGVPGGEAIAKETGCDFFSVPVHLGTSGVEKAINPLANINDAEKTLLKACVEGLKGNIAKGVEFAHNPPQK

>Hypocrea_atroviridis_ATCC_20476_fc

MVKAAVLGASGGIGQPLSLLLKICPLVDELVLYDVVNSPGVTADLSHISTAAKTSGYLPKDEGLKNALTGCELVLIPAGIPRKPGMTRDDLFTVNAGIVRDLVHGVAEFCPKAFVLIISNPVNSTVPIAAEVLKKAGVFDAKRLFGVTTLDILRAETFAQKYTGEKNPSDATIHVIGGHSGETIVPVYSLAKPVADIPESEYAEIIKRVQFGGDEVVKAKDSAGSATLSMAYAGYRFALSVMKAAKGEKGIVEPTFVHLSGINGGDVVAKETGLEYFSMPVELGPSGAENIVNILPNVNEREKALLEVCKSGLQGNIAKGISFVQNPPQKL

>Metarhizium_robertsii_fc

MVKAVVAGASGGIGQPLSLLLKNSPHIDELALYDVVNTPGVAADLSHISSTAKVTGYLPANDGAKAAFKDADIIVIPAGIPRKPGMTRDDLFNINAGIVKGLIETIAEVAPKAFVLVISNPVNSTVPISAEVLKAKKVFNAQRLFGVTTLDIVRAETFVAEIVGQKEPQKLTIPVVGGHSGETIVPLFSKANPAVNIPADKYDALVNRVQFGGDEVVKAKDGAGSATLSMAYAGFRFAEKLLRAVKGEKGLVEPSYVYLPGVPGGDAIAKETGCDFFSVPVELGPNGAEKATNPLEGLTEKEKGLLGKAVEGLKGNIKKGIDFAHNPPQK

>Magnaporthe_oryzae_Y34_fc

MVKAVVAGAAGGIGQPLSLLLKLCPLVDELALYDVVNTPGVAADLSHISSNAKIAGYLPKDDGGKKALKDADLIVIPAGVPRKPGMTRDDLFNINAGIVKGLIEIAAEVAPKAFILVISNPVNSTVPISAEVLKAKGVFNPQRLFGVTTLDIVRAETFVAEIAGKSNPQELTVPVIGGHSGETIVPLFSQVKPAVTIPDDKYDALVNRVQFGGDEVVKAKDGAGSATLSMAYAGYRFAEKLLKAIKGAKGLVEPSYVYLPGVPGGEAIAKKTGCDFFSVPIELGPNGAEKAHDVLGELTSKEQTLLEAAVNGLKGNIQKGVQFVNSPPQK

>Pycnoporus_coccineus_BRFM310_fc

MVKAVVLGAAGGIGQPLSLLLKTNPSITELSLYDIVNTPGVAADLSHIDTPAKVAGYLPPDDGLKKALTGADIVIIPAGVPRKPGMTRDDLFKINAGIVRDLAKGIAEAAPKAFVLVISNPVNSTVPIVAEVFKKAGVFDPKKIFGVTTLDVVRASTFVSEVLGDLSLAPKVTVPVVGGHSGVTIVPLLSQSSHPLPSGFSQESLDKLINRIQFGGDEVVKAKDGAGSATLSMAYAGAEFAYKIIRAAKGEKGIVAPTFVNLAADKEGGDALKKEIGRDLDYFSAPVELGTEGVAKINSLGKITEHEASLIKAAVPELATNIEKGVSFIESSKL

>Rhizoctonia_solani_fc

MSLKAVVLGAAGGIGQPLSLLLKANPAITELSLYDIVNTPGVAADLSHIDTPAIVEGYLPPNEGLAKALNGANIVVIPAGVPRKPGMTRDDLFKINAGIIRDLAIAIATNAPKAFILVISNPVNSTVPIVAEILKKHGVFDPKRLFGVTTLDVVRASTFIASVAGSPSAAPTYTVPVVGGHSGVTIVPLLSQATPSLPDSTAQSEIEALTKRIQFGGDEVVKAKDGAGSATLSMAYAAAEFTTAVLKGLKGEDVTVPSYVHLTADPEGGKALQSEIGAELEYFSTRIKLGPNGVEKIHPLGKLTEYETKLIKEAIPELKVNINKGTEFIAPSKL

>Basidiobolus_meristosporus_CBS_931.73_fc

MVKVAVLGAAGGIGQPLSLLLKQNTLISHLALYDLVNVPGVAADLSHINTPSKVTAHLGADNLQEALADAHVVVIPAGVPRKPGMTRDDLFKINAGIVRDLATAASKYCPKAFMLIISNPVNSTVPIVAEVFKKAGVYDPKRLFGVSTLDVVRASRFVSEVNPELNPVDTRVTVIGGHSGTTIIPLLSQLNPTQKFTQEQIEALTHRIQFGGDEVVKAKDGAGSATLSMAFAGARFAINILEASVGGKTGIIEPSYVDLSAYVEGGNTVKAETQDCAYFATNVELGKDGVEKIFSIGKLSEFEQKLLAEAIPQLKANVESGVNFINA

>Rhizopus_microsporus_fc

MVKVTVCGAAGGIGQPLSLLLKQSNLISHLALYDIVNAHGVAADLSHIDTSSKVTGHVGPLELEEAIKDADIVVIPAGVPRKPGMSRDDLFKINAGIVRDLAVAAANYAPKALMCIISNPVNSTVAIVAEVFKKYDVYNPRKIFGVTTLDLVRSSTFLAELLEANPRQVNVPVVGGHSGVTIIPLLSQVPGTEKLSEKQLEDLTKRIQFAGDEVVKAKNGAGSATLSMAYAGARFTLNLVESVFNGVTRIECAYVNLASDPEGAQSVHRIAGEEMEYFSVPLEFGREGIQRILPIGKMSRFEYQLLTSAVPELKSNIVKGVSFITDDSKL

>Mucor_circinelloides_f._circinelloides_1006PhL_fc

MVKVCVCGAAGGIGQPLSLLLKQSERITHLSLYDIVNTPGVAADLSHINTKSKVTGHVGPEQLKDAIQDSAVVVIPAGVPRKPGMTRDDLFKINAGIVRDLATAAAKYAPKAFMCIISNPVNSTVPIVAEVFKQHNVYDPRRIFGVTTLDIVRASTFVSELIGDEPSKLQVPVVGGHSGVTILPLLSQVTNKLTQDQIEKVTHRIQFGGDEVVKAKNGAGSATLSMAFAGARFALAVIDAAFAGKDIVECTYINLEADQNGAKAVKDLVGGDVPYFSVPVKLGKNGVERVLPIGSISPYEKQLFTKAASELKGNIAKGTNFVAGSKL

>Lichtheimia_corymbifera_JMRC:FSU:9682_fc

MVKVCVCGAAGGIGQPLSLLLKQSERITHLSLYDIVNTPGVAADLSHINTKSKVTGHVGPEQLKDAIQDSAVVVIPAGVPRKPGMTRDDLFKINAGIVRDLATAAAKYAPKAFMCIISNPVNSTVPIVAEVFKQHNVYDPRRIFGVTTLDIVRASTFVSELIGDEPSKLQVPVVGGHSGVTILPLLSQVTNKLTQDQIEKVTHRIQFGGDEVVKAKNGAGSATLSMAFAGARFALAVIDAAFAGKDIVECTYINLEADQNGAKAVKDLVGGDVPYFSVPVKLGKNGVERVLPIGSISPYEKQLFTKAASELKGNIAKGTNFVAGSKL

>Loa_loa_ac

MTAIGRMSTVLEPLMKNLQMCSQRLSSSAPKIALLGAAGGIGQPLGLLLKMNKHVANLALYDIKDTPGVAADLSHIDTRAHVTGYTGANELDKALKGADIVVIPAGLPRKPGMSRDDLFNTNASIVRDLSEAAAKYCPKAFVAIITNPVNSTVPIACEIFKKHGVFDPRRIFGVTTLDVVRSAAFVAEAKNLDAEQTNIPVIGGHSGITIIPLLSQAKPFCKFSDDEVKKLTERIQNAGTEVVKAKAGAGSATLSMALAASKFVENLLKGLRGEKSVQCAYVASDMCNGVDYFATPLEFGKNGVEKILGIGELSAYEQGLVDAAIPELKKNISKGKKFVSG

>Achlya_hypogyna_fc

MLARSLTARLFSSAAAGKKVAVLGAAGGIGQPMSLLLKDSDHISHLSLFDVVNTPGVAADLSHCNTRSTVTGHAGMENIEAALTGMDVVVIPAGVPRKPGMTRDDLFNTNASIVQSLAAACAKYCPEAMMLIIANPVNSTVPIVAETFKKAGVYNPKRLFGVTTLDVVRANTFVANSQGWNPRTTNVTVIGGHAGTTILPLLSHLKLDRQWSEDELHALIKRIQFGGDEVVQAKNGAGSATLSMAYAGARFTTRLLDAMTGKKDVIECSYTQNDVTKLPFFSTPVTLGPNGVEKVHEFGALNAVEQANFDAMLPDLEKQIAKGVAFVNKN

>Saccharomyces_cerevisiae_fm

MLSRVAKRAFSSTVANPYKVTVLGAGGGIGQPLSLLLKLNHKVTDLRLYDLKGAKGVATDLSHIPTNSVVKGFTPEEPDGLNNALKDTDMVLIPAGVPRKPGMTRDDLFAINASIVRDLAAATAESAPNAAILVISNPVNSTVPIVAQVLKNKGVYNPKKLFGVTTLDSIRAARFISEVENTDPTQERVNVIGGHSGITIIPLISQTNHKLMSDDKRHELIHRIQFGGDEVVKAKNGAGSATLSMAHAGAKFANAVLSGFKGERDVIEPSFVDSPLFKSEGIEFFASPVTLGPDGIEKIHPIGELSSEEEEMLQKCKETLKKNIEKGVNFVASK

>Lachancea_thermotolerans_ATCC_56472_fm

MFAKVAKRAFSSSTANPYKVTVLGAGGGIGQPLSLLLKLNHKVTDLRLYDLKGAAGVAADLSHIPTNSVVKGFSADAQDGIKSALKDTDVVLIPAGVPRKPGMTRDDLFSINASIVRDLAAACAENAPNAAILVISNPVNSTVPIVAEVLKSKGVYNPKKLFGVTTLDVIRASRFISEVSGTNPTTEKVNVIGGHSGITIIPLISQTKHKLMDKEKRDALIHRIQFGGDEVVKAKNGAGSATLSMAQAGARFANSVLAGLEGEADVIEPSFVDSPLFKSEGIEFFASPVKLGPQGVEKIFSIGEISSEEQELLDKCKETLKKNIEKGTAFVKS

>Zygosaccharomyces_bailii_CLIB_213_fm

MFTRIARRAFSTSKVNPYKVTILGAGGGIGQPLSLLLKLNHKVTDLRLYDLKGAPGVAADLSHIPTNSKVSGFTPDNEGLSKALKDADVVLIPAGVPRKPGMTRDDLFSINASIVRDLAKAAGDAAPNANILVISNPVNSTVPIVAETLKKKGVYNPHKLFGVTTLDSIRAARFISEVEGTDPTTEHVNVVGGHSGITIVPLISQTKYKTMDKTTREALIHRIQFGGDEVVKAKDGAGSATLSMAQAGATFANAVLSGLGGERDVIASSYVDSPLYKSEGVEFFASPVTLGPDGISKVHPVGDISSEEEQLLEKAKETLKKNITKGFAFIDKE

>Hanseniaspora_uvarum_DSM_2768_fm

MLQTTFSRRLFSQSMANQYKVAVLGASGGIGQPLSLQMKLNHKVSELRLYDLRLAKGVAADLSHIPTDSKVIGYSNDEPEGLAKALDNADVVLIPAGVPRKPGMTRDDLFSINAGIVRDLAEAVAKNCPNASICVISNPVNSTVPIVAKVLQKHGVYNPKKLYGVTTLDIIRASRFISEVEGTNPTQEKVNVVGGHSGITIIPLISQTNHKLMPKEVKDKLIHRIQFGGDEVVKAKDGAGSATLSMSYAGAVFADKVLDGLLGEKDVVVNSFVESPLYKEEGIDFFSSPITLGPEGVQKVHPIGEISSEEEALLAECKETLKKNIAKGYKFVEESKL

>Candida_albicans_SC5314_fm

MFSKVATRSFSSSASNAYKVAVLGAGGGIGQPLSLLLKLNHKVTDLALYDIRGAPGVAADVSHVPTNSTVKGYNPDQIEEALTGSDVIVIPAGVPRKPGMTRDDLFNTNASIVRDLAKAAADYAPNAAVCIISNPVNSTVPIVAEVFKSKGNYNPNKLFGVTTLDVLRAARFVSEVAGTNPVNENVPVVGGHSGVTIVPLLSQTKHKDLSGETRDALVHRIQFGGDEVVQAKDGAGSATLSMAQAGARFAGAVLDGLAGEKDVIECTFVDSPLFKDEGVDFFSTKVTLGVDGVKTVHPIGEISDYEEAQVKEAKDTLIKNIKKGVDFVAQNP

>Debaryomyces_hansenii_ATCC_36239_fm

MFTKVAARSFSSSASSAYKVAVLGANGGIGQPLSLLLKLNHKVTDLALYDLKGAPGVAADVSHIPTNSTVSGYDPEGLEQALTGSDIIVIPAGVPRKPGMTRDDLFNTNASIVRDLAKAAADYAPDAAVCVISNPVNSTVPIVAEVLKSKGTYNPKKLFGVTTLDVLRASRFVSEVAGSNPVHEKVTVVGGHSGITIVPLLSQTEHKSLDQETRDALIHRIQFGGDEVVQAKNGAGSATLSMAQAGARFTGAVLDGLAGERDIVEPSFVDSPLFKSEGVEFFSSKVTLGQDGVSTVHPLGGLSDHEESLVKEAKDTLIKNIQKGVDFVKQNP

>Clavispora_lusitaniae_ATCC_42720_fm

MFKAATRSFSSSPSAAYKVAVLGAGGGIGQPLSLLMKLNHKVTDLALYDLRGAPGVAADVSHVPTNSTVKGYEPEHLEEALKGADVVVIPAGVPRKPGMTRDDLFNTNASIVRDLAKAVADTAPNAAVCIISNPVNSTVPIVAEVFKSKGVYNPKKLFGVTTLDVLRASRFVSEVAGTNPVHEKVTVVGGHSGITIVPLLSQTTHKDLPAETRDALVHRIQFGGDEVVQAKNGAGSATLSMAQAGARFAGSVLNGLAGEKDIVEPTFVDSPLFKDEGVEFFSSKVTLGVDGVKTVHPLGELSDYEEELVKKAKETLITNIKKGVDFVKQNP

>Yarrowia_lipolytica_CLIB_122_fm

MFRTRVTGSTLRSFSTSAARQHKVVVLGANGGIGQPLSLLLKLNKNVTDLGLYDLRGAPGVAADVSHIPTNSTVAGYSPDNNGIAEALKGAKLVLIPAGVPRKPGMTRDDLFNTNASIVRDLAKAVGEHAPDAFVGVIANPVNSTVPIVAEVLKSKGKYDPKKLFGVTTLDVIRAERFVSQLEHTNPTKEYFPVVGGHSGVTIVPLVSQSDHPDIAGEARDKLVHRIQFGGDEVVKAKDGAGSATLSMAQAAARFADSLLRGVNGEKDVVEPTFVDSPLFKGEGIDFFSTKVTLGPNGVEEIHPIGKVNEYEEKLIEAAKADLKKNIEKGVNFVKQNP

>Aspergillus_flavus_ATCC_200026_fm

MIPSQPCVGKESETDRFSRQQTKFVSEFLLTGNQRPPIPLPFLSFSPFSSSFLFSFHLTFPFLPDRILSILSYTSFTMFAARQSFNLLQKRAFSASASQASKVAVLGAAGGIGQPLSLLLKLNPRVSELALYDIRGGPGVAADLSHINTNSTVSGYEATPSGLRDALKGSEIVLIPAGVPRKPGMTRDDLFNTNASIVRDLAKAAAEASPEANILVISNPVNSTVPIVSEVFKSKGVYNPKRLFGVTTLDVVRASRFISQVQKTDPSNEAVTVVGGHSGVTIVPLLSQSSHPSIEGKTRDELVNRIQFGGDEVVKAKDGAGSATLSMAMAGARMAESLLKAAQGEKGVVEPTFVDSPLYKDQGVDFFASKVELGPNGVEKILPVGQVNAYEEKLLEACLGDLKKNIQKGIDFVKANP

>Aspergillus_oryzae_ATCC_42149_fm

MFAARQSFNLLQKRAFSASASQASKVAVLGAAGGIGQPLSLLLKLNPRVSELALYDIRGGPGVAADLSHINTNSTVSGYEATPSGLRDALKGSEIVLIPAGVPRKPGMTRDDLFNTNASIVRDLAKAAAEASPEANILVISNPVNSTVPIVSEVFKSKGVYNPKRLFGVTTLDVVRASRFISQVQKTDPSNEAVTVVGGHSGVTIVPLLSQSSHPSIEGKTRDELVNRIQFGGDEVVKAKDGAGSATLSMAMAGARMAESLLKAAQGEKGVVEPTFVDSPLYKDQGVDFFASKVELGPNGVEKILPVGQVNAYEEKLLEACLGDLKKNIQKGIDFVKANP

>Aspergillus_niger_CBS_513.88_fm

MFAARQSLNLLQKRSFSASASQASKVAVLGAAGGIGQPLSLLMKQNPLVTDLALYDIRGGPGVAADISHINTNSTVKGYEPTPSGLRDALKGSEIILIPAGVPRKPGMTRDDLFNTNASIVRDLAKAAAEAAPEANILVISNPVNSTVPIVSEVYKSKGVYNPKRLFGVTTLDVVRASRFISQVKGTNPANEAVTVIGGHSGVTIVPLLSQSNHPDISGTVRDELVNRIQFGGDEVVKAKDGAGSATLSMAMAGARFADSLLRAANGEKGIVEPTFVESPLFKDQGVNFFASKVELGPNGVEKIHEVGPVNEYEQGLIQTALGDLKKNIQKGVDFVKQNP

>Neosartorya_fumigate_CEA10_fm

MFAARQSFNLLQKRAFSASASQASKVAVLGAAGGIGQPLSLLLKLNPRVSELALYDIRGGPGVAADLSHINTNSTVTGYDPTPSGLRDALKGSEIVLIPAGVPRKPGMTRDDLFNTNASIVRDLAKAAAEASPEANILVISNPVNSTVPIVAEVFKSKGVYNPKRLFGVTTLDVVRASRFISQIKKTDPAKEAVPVVGGHSGVTIVPLLSQSNHPDIEGETRDTLVNRIQFGGDEVVKAKDGAGSATLSMAMAGARFAESLLKAAQGEKGVIEPTFVESPLYKDQGVDFFASRVELGPNGVEKILEVGKVNAYEEKLIQAALTDLKKNIQKGRDFVAQNP

>Penicillium_rubens_ATCC_28089_fm

MFAARRTVNLFQKRAFSASAINASKVSVLGAAGGIGQPLSLLLKLNPRVSELALYDIRGGPGVAADLSHINTNSTVTGYNPDASGLRDCLEGSEIILIPAGVPRKPGMTRDDLFNTNASIVRDLAKAAAEAAPKAHVLVIANPVNSTVPIVAEVYKARNVYDPKRLFGVTTLDVVRASRFISQVQNTNPAGEAVPVVGGHSGVTIVPLLSQSNHSSIAGQARDALVNRIQFGGDEVVKAKDGAGSATLSMAMAGARFAESLLRAAQGEKGVIEPTFVDSPLYKDQGIDFFASRVELGPNGVEKINSVGEVNEYEQGLLDACLTDLKKNIQKGVDFVKANP

>Trichophyton_rubrum_ATCC_MYA-4607_fm

MFAARRTATLFQRRAFSATASQASKVSVLGAAGGIGQPLSLLMKLNPRVSQLALYDIRGGPGVAADLSHINTNSVVSGHEPTPSGLKEALEGSEIVLIPAGVPRKPGMTRDDLFATNASIVRDLAKAAADHCPNANILVISNPVNSTVPIVAEVFKAKNVYNPKRIFGVTTLDVLRASRFVSEIKNTDPADEKIPVVGGHSGVTIIPLISQSNHPDIAGEALDKLTNRIQFGGDEVVKAKAGAGSATLSMAQAGARFADSLLKATQGEKNVIEPTFVDSPLYKDQGIEFVASNVRLGPNGVEEILPIGKVSEYEQKLLENCLVELKKNIQKGVDFVKANP

>Ajellomyces_dermatitidis_ATCC_18188_fm

MFAARRAFGFAQRRAFSVSAQQSSKVTVLGAAGGIGQPLSLLMKLNPRVTQLALYDIRGGPGVAADLSHINTNSTVTGYDPTPSGLREALKDAEIVLIPAGVPRKPGMTRDDLFNTNASIVRDLAKAAAEASPNANILVISNPVNSTVPIVAEVFKARNVYNPKRLFGVTTLDVVRASRFISQVKGTDPANENVTVVGGHSGVTIVPLISQSNHPDISGEKLDALVNRIQFGGDEVVKAKDGAGSATLSMAMAGARFAESLLKASQGVKDVVEPTFVESPLYKDQGVNFFASSVRLGPNGVEEILPIGKVSAHEQKLVGACLVDLKKNIAKGVEFVKNNP

>Ajellomyces_capsulatus_H88_fm

MLAARRAFGFAQRRAFSASAPQASKVTVLGAAGGIGQPLSLLMKLNPRVTQLALYDIRGGPGVAADLSHINTNSTVTGYDPTPSGLREALKDSEIVLIPAGVPRKPGMTRDDLFNTNASIVRDLAKAAAEASPKANILVISNPVNSTVPIVAEVFKSKNVYNPKRLFGVTTLDVVRASRFISQVKGTDPANEKVTVVGGHSGVTIVPLVSQSNHGDISGEKLDALVNRIQFGGDEVVKAKDGAGSATLSMAMAGARFAESLLKASQGVKDVIEPTFVDSPLYKDQGIDFFASNVRLGPNGVEEIIPVGAVSPYEQKLVDACLVDLKKNIAKGVEFVKNNP

>Paracoccidioides_brasiliensis_Pb03_fm

MFAARRAFGFAQRRAFSVSAPQSSKVAVLGAAGGIGQSLSLLMKLSPRVTQLALYDIRGGPGVAADLSHINTNSTVTGYDPTPSGLRDALKDSEIVLIPAGVPRKPGMTRDDLFNTNASIVRDLAKAAADASPNANILVIANPVNSTVPIVAEVFKSKNVYNPKRLFGVTTLDVIRASRFISQAKGTDPKDEKVTVVGGHSGVTIVPLISQSNHPDISGEKLETLVNRIQFGGDEVVKAKDGAGSATLSMAMAGARFAESLLKASQGEKDVIEPTFVDSPLYKDQGINFFASNVKLGPNGVEEILPVGKVSEYEQKLIDTCLVDLKKNITKGVQFVKTNP

>Coccidioides_posadasii_RMSCC_757_fm

MFAARRTVGLFQRRAFSASAQQNSKVTVLGAAGGIGQPLSLLMKLNPRVSQLALYDIRGGPGVAADLSHINTNSTVTGHDPTPSGLREALTDAEIVLIPAGVPRKPGMTRDDLFNTNASIVRDLAKAAADAAPKANILVISNPVNSTVPIVAEVFKSKNVYNPKRLFGVTTLDVVRASRFISEIKKTDPANEEVPVIGGHSGVTIVPLVSQSNHPDITGEALEALVNRIQFGGDEVVKAKAGAGSATLSMAMAGARFAESLLKASQGVKDVIEPTFVESPLYKSQGIDFFASRVRLGPNGVEEILPVGKVSEYEQKLLDACLVDLKKNITKGIDFVKNNP

>Claviceps_purpurea_20.1_fm

MFAARIQRRAFSATARDLSKVAVLGASGGIGQPLAMLMKLNPKVTELALYDVRGAPGVAADLSHINTKSTVTGYEFSPAGLAACVKGADIVLIPAGVPRKPGMSRDDLFNTNASIVRDLSKAVAVSAPKAKVLVIANPVNSTVPICAEVFKAQGVYNPKTLFGVTTLDVVRASRFVSEIKKTDPKDENVTVVGGHSGVTIVPLFSQSNHPDLSSNKDLVKRVQFGGDEVVQAKDGAGSATLSMAMAGARMADSLLRAAAGEKGIVEPTFVDSPLFKDQGVDFFSSQVELGPDGVEKILPIGEIDANEQKLVEACLGDLKKNIQKGIKFVAENPSA

>Colletotrichum_graminicola_M1.001_fm

MSFVQRRMFSASARSLSKVTVLGAAGGIGQPLSLLMKLSPRVTELALYDIRGGPGVAADISHVNTKSSVKGYDPTATGLASALKGAEVVLIPAGVPRKPGMTRDDLFNTNASIVRDLAKACAESCPDANILVISNPVNSTVPIVSEVFKARGVYNPKRLFGVTTLDVVRASRFVSEIKDTDPKDENITVVGGHSGVTIVPLFSQSNHPELSSNAELVNRVQFGGDEVVKAKDGAGSATLSMAFAGARMAESLLRASQGEKGIVEPTFVDSPLYKDQGIEFFSSKVELGPNGVEKILPIGKVDAVEEKLLEACFADLKKNIAKGVAFVAQNPPK

>Exophiala_dermatitidis_ATCC_34100_fm

MSFVRQMFGQVQRRAFSASARQSSKVAVLGAAGGIGQPLSLLMKLNPRVSQLALYDIRMGPGVAADLSHINTKSTVKGYDPTPSGLRECLTGSEIILIPAGVPRKPGMTRDDLFNTNASIVRDLAKAAADAAPEAKLLVISNPVNSTVPICAEVFKSKGVYNPKRLFGVTTLDVVRASRFISEIKGTDPAEENVTVVGGHSGVTIVPLISQSRHPDISGEKLDALVNRIQFGGDEVVKAKDGAGSATLSMAFAGARFAESLLRASQGEKGVIEPTFVDSPLYKDQGVEFFASRVELGPEGAEKILPVGKINKYEEGLLEACLTDLKKNIQKGIDFVKANP

>Cladophialophora_psammophila_CBS_110553_fm

MSFARQMFGQVQRRAFSASARQSSKVAVLGAAGGIGQPLSLLMKLNPRVSQLALYDIRLGPGVAADISHINTKSTVKGYDPTPSGLRECLEGSDIILIPAGVPRKPGMTRDVDLFNTNASIVRDLAKAAADAAPNANLLIISNPVNSTVPICAEVFKSKGVYNPKRLFGVTTLDLVRASKFVSELKGTDPADEKITVVGGHSGVTIVPLISQSGHHDITGEKLDALINRIQFGGDEVVKAKDGAGSATLSMAMAGARFAESLLKASQGEKGVVECTFVDSPLYKDQGIDFFSSKVELGPDGVQKIHEVGRINKYEENLLEAAMGDLKKNIQKGVDFVKANP

>Hypocrea_jecorina_QM6a_fm

MFAARIQRRAFSASARNLSKVAVLGAAGGIGQPLSLLLKLNTRVTELALYDIRGGPGVAADISHVNTKSLVKGYEATPSGLAAALKGSDIVLIPAGVPRKPGMTRDDLFNTNASIVRDLAKAVAESAPKAKLLIISNPVNSTVPICAEVFKARGVYDPKKLFGVTTLDVVRASRFVSEIKGTDPKDENITVVGGHSGVTIVPLFSQSNHPELSSNAELVNRVQFGGDEVVKAKDGAGSATLSMAFAGARMADSLLRAADGEKGVIEPTFVDSPLYKDQGIDFFSSNVELGPNGVEKIHPIGKIDANEEKLIQACLGDLKKNIAKGVAFVNENPGK

>Metarhizium_robertsii_arSEF_23_fm

MLASRIQRRAFSASARNLSKVAVLGAAGGIGQPLSLLLKQNPKVTELALYDIRGGPGVAADLSHINTKSTVKGYEPTAAGLAECVKGSDIVLIPAGVPRKPGMTRDDLFNTNASIVRDLAKAVAESAPNAKLLVIANPVNSTVPICAEVFKARGVYNPKTLFGVTTLDVVRASRFVSEIKGTDPKDENITVIGGHSGVTIVPLFSQSNHPDLSSNADLVKRVQFGGDEVVKAKDGAGSATLSMAMAGARMADSLLRAAAGEKGVIEPTFVDSPLYKDQGIDFFSSKVELGPDGVKQILPVGEIDAAEEKLVEACLGDLKKNIEKGVTFVAQNPGK

>Ophiocordyceps_sinensis_Co18_fm

MFAAGIQRRAFSASARDLSKVAVLGAAGGIGQPLSLLLKMNPRVTELALYDIRLAPGVAADVAHINTKSKVKGYDATPSGLADCLKGSEVVLIPAGVPRKPGMTRDDLFNTNASIVRDLATAVAASAPKAKVLIISNPVNSTVPICAEVFKSKGVYNPKTLFGVTTLDVVRASRFVSEIKDTDPKDENITVVGGHSGVTIVPLFSQSKHPELSSNAELVKRVQFGGDEVVKAKDGAGSATLSMAMAGARMAESLLRAAQGEKGVVEPTFVDSPLYKDQGIQFFSSKVELGPNGVEKILPLGKLDVIEEKLLQACLSDLKKNIDKGVAFVAQNPGK

>Beauveria_bassiana_arSEF_2860_fm

MFAASRIQTRAFSASARNLSKVAVLGAAGGIGQPLSLLLKLNPRVTDLALYDIRGGPGVAADISHVNTKSTVTGYDPTPEGLAACLKDAEIVLIPAGVPRKPGMTRDDLFNTNASIVRDLAKAVAQSAPKAKTLVIANPVNSTVPICAEVFKAKGVYNPKTLFGVTTLDVVRASRFVSALKGTDPKDENITVVGGHSGVTIVPLFSQSNHPDLSSNADLIKRVQFGGDEVVKAKDGAGSATLSMAMAGARMADSLLRAAAGEKVIEPTFVESPLYKDQGIEFFSSKVELGPDGVKEILPVGKVDATEQGLLDACIVDLKKNIEKGVSFVAQNPGN

>Sporothrix_schenckii_ATCC_58251_fm

MFAARIQRRAFSASARDLSKVTVLGAAGGIGQPLSLLLKLNPRVTELALYDIRGGPGVAADISHVNTKSTVKGYEPTASGLSAALKGSEVVLIPAGVPRKPGMTRDDLFNTNASIVRDLAKAVAESAPNANVLVISNPVNSTVPIVKEVFKARGVYNPKRLFGVTTLDVVRASRFVSEIKGSDPKDENITVVGGHSGVTIVPLFSQSSHPELSSNADLVKRVQFGGDEVVQAKDGAGSATLSMAMAGARMADSVLRASQGEKGVVEPTFVESPLYKDQGIEFFSSKVELGPEGVEKILPVGEVDSVEQGLLEACFTDLKKNIAKGVEFVASNPGK

>Neurospora_crassa_ATCC_24698_fm

MFAASRIQTRAFSASARQLTKVSVLGAAGGIGQPLSLLLKLNPRVSELALYDIRGAPGVGADLSHINTKSTVKGYEPTASGLADALKGSEIVLIPAGVPRKPGMTRDDLFNTNASIVRDLAKACAESCPEANILVISNPVNSTVPIVSEIFKKAGVYNPKRLFGVTTLDVVRASRFVSEIKGTDPKDENITVVGGHSGVTIVPLFSQSKHPELSKNEQLIHRVQFGGDEVVKAKDGAGSATLSMAMAGARMAESLLRAAQGEKGVIEPTFVDSPLYKDQGIDFFASKVELGPNGVEKIYPVGPVDEVEQKLLDACLVDLKKNIQKGKDFVAANPGK

>Glarea_lozoyensis_ATCC_20868_fm

MFAARRAFAGVAQSRAFSASARDLSKVTVLGAAGGIGQPLSLLLKLNPRVTELALYDIRGGPGVAADISHINTKSKVTGYDPTPSGLASALKGAEIVLIPAGVPRKPGMTRDDLFNTNASIVRDLAKAAAESAPEANILVISNPVNSTVPIVAEIFKAKGVYNPKRLFGVTTLDVVRASRFVSEIKNSDPVDENITVIGGHSGVTIVPLFSQSSHPDLVGNAELLQRVQFGGDEVVKAKDGAGSATLSMAMAGARFAESLLKAAQGQKGVVEPTFVDSPLYKDEGIDFFASKVELGPNGVEKIMDVGKLDAQEQKLLDACKEDLKKNIKKGVVFVAENPGK

>Pseudogymnoascus_destructans_ATCC_MYA-4855_fm

MFAARTFSAAQRRAFSASARDLSKVTVLGAAGGIGQPLSLLLKLNPRVTELALYDIRGGPGVAADISHINTGSNVTGYEPTPSGLAAALKDAEIVLIPAGVPRKPGMTRDDLFNTNASIVRDLAKAAAQSAPNANILVIANPVNSTVPIVAEVFKKAGVYNPKRLFGVTTLDVVRASRFVSEIKKTDPADEAITVVGGHSGVTIVPLFSQSKHADLVGNAALLNRVQFGGDEVVKAKDGAGSATLSMAMAGARFAESLLKAAQGEKGVTEPTFVDSPLYKDQGVDFFASKVELGPSGVEKIHEVGKVTAEEQKLLDAALADLKKNIEKGVQFVATNPGN

>Zymoseptoria_tritici_CBS_115943_fm

MFAARATKSFMGAAVQRRAFSASASNLSKVVVLGAGGGIGQPLSLLLKLNPRVTELALYDIKGAPGVAADVGHINTKSTVTGYSPDGEGLGACLKGAEIVVIPAGVPRKPGMTRDDLFNTNASIVRDLAKAAAKHCPEASLLVISNPVNSTVPITAEVFKAAGVYNPKKLFGVTTLDVVRASRFISQIKSSDPANEKVTVVGGHSGETIVPLLSQAGHKLEGDELANYIKRVQFGGDEVVKAKDGAGSATLSMAMAGARFTESLLKAAQGEKNVKEETYVDSPLYKDQGVNYFSSTVTLGPNGVEEIHPVGKITEHEQGLLDVCLKDLKKNIEKGEQWVKENP

>Pneumocystis_jirovecii_RU7_fm

MVDSKNNDLYKDKHDTETNSFTMTSMKRGPASIVFSDTVNTYGCILPVISLSIDIQKILHYLLPLPSVIRKFYNCKSFKLADSTVIPVFYIRERISQFAPEHWLNTRTCTHYLRISPYSRRFYPIHDIIISSQCVFMPQKLFLRSSARSIFFIIKYLPHPPSFSVILHWLYTNNTHKLYRNILRAINKHKTLEWIKGFSLNIRSLGIVNQNLVDAIKVSILGAGGGIGQPLSLLLKMNSRVSELALYDIRGAPGVAADCSHINTPCKVSGYGPEENGLFKALRDADVVLVPAGVPRKPGMTRDDLFVTNASIVRDLAKFTADVAPNAHLLIISNPVNSTVPICAEVYKRKGVYNPKKLFGITTLDAVRASWFVSEMKNTDPRDENVDIVGGHSGVTIVPLLSKVSHNFTDEETETLTTRIQFAGDEVVKAKNGTGSATLSMAYAAARFTNSLLRGIQGERDVIESAYIETTLYLNKGCRFFSMNVGLGLEGIEKVYPFENTSAYEKELLEVCYVELQKNIEKGIKFVEETY

>Gloeophyllum_trabeum_ATCC_11539_fm

MLARSAIRTLSSQSRMFSSSASRQFKVAVLGAGGGIGQPLSLLLKLDPLVTELNLYDIRGAPGVAADVSHVDTNSEVKGFAADKLDEALDGTRVIVIPAGVPRKPGMTRDDLFNTNASIVRDLAAAIGRVSPQACICVISNPVNSTVPIVAATLEKQGVFDPKRLFGVTTLDVVRAARFLGGIQGVDPKQTPVTVVGGHSGATIVPLLSQNSHGKGLKGETYEKLVHRIQFGGDEVVKAKDGAGSATLSMAYAGARFTNSVLRGLNGEKGLVTPTFVKSPLFADQGVDFFSSNVELGPNGVEKIHPIGPISPEEEKLLQACLPDLKKNIEKGKAFVAQS

>Laccaria_bicolor_S238N-H82_fm

MFARTAARSLAAPSSRLFSSSAARLHKVAVLGAGGGIGQPLSLLLKSDPLVTSLSLYDIRGAPGVAADVSHVDTGSEVTGYAADQLDQALEGVKVVVIPAGVPRKPGMTRDDLFNTNASIVRDLAAAVARVAPEAHILVISNPVNSTVPIVAATLEKAGVFDPRRVFGVTSLDVVRARRFLSEAVGTSPKNTGVTVVGGHSGPTIVPLLSQTEWGKQVTGEAWKKLVHRIQFGGDEVVKAKDGAGSATLSMAYAGASFTNHLLRGLSGEKGVITPTFVKNPLYADQGIDFFSSPVELGVNGVEKIHPIGTISADEQALLDACLPELKKNIEKGKAFVA

>Cryptococcus_gattii_serotype_b_WM276_fm

MFARQVAKNSSSLARGFASSARSNRKVAVLGAAGGIGQPMSLLLKQNPGVTALSLYDIRGAPGVAADISHVNTHSTVKGFEKDDIKEALTGAEIVIIPAGVPRKPGMTRDDLFNTNASIVRDLAEACAEYCPKAFIGVIANPVNSTVPIFAEVYKKKGIFDEKRIFGITTLDVVRASRFLGEVKGKDPKDVKVTVVGGHSGVTIVPLLSQTPEGKDVSGEAYKALVHRIQFGGDEVVKAKAGTGSATLSMGYAGARFTDSLIRALNGETGVVEPTFVKSPLYESEGVEYFASNVELGPEGVKKINPVGQLSAEEQELLKACLPDLAKNIKKGVDFVKA

>Cryptococcus_neoformans_var._neoformans_serotype_D_b-3501A_fm

MFARQVAKNSSSLARGFASSARSNRKVAVLGAAGGIGQPMSLLLKQNPGVTGLSLYDIRGAPGVAADISHVNTHSTVKGFEKDDIKEALTGAEIVIIPAGVPRKPGMTRDDLFNTNASIVRDLAEACAEYCPKAFIGIISNPVNSTVPIFAEVLKKKGVFDEKRVFGITTLDVVRASRFLGEIKGKDPKDIKVTVVGGHSGVTIVPLLSQTPEGKDVSGEAYKALVNRIQFGGDEVVKAKAGTGSATLSMGYAGARFTDSLIRALNGETGIVEPTFVKSPLYESEGVEYFASNVELGPEGVKKINPVGQLSAEEQELLKACLPDLVKNIKKGVDFVKA

>Mucor_circinelloides_f._circinelloides_1006PhL_fm

MFATSRLFTSIAAKRAFSTSAANLSKVAVLGAAGGIGQPLSLLLKENPHVTHLSLYDIVNTPGVAADIGHINTNSKVTGHTPENDGLKAALEDAHVVVIPAGVPRKPGMTRDDLFNTNASIVRDLASAAAKYCPNAHFLIISNPVNSTVPIFAETLKKAGVFNPKRLYGVTTLDVVRASRFVAEVKNLDPKDVKVTVVGGHSGVTIVPLLSQTGIEFTKEELDALTHRIQFGGDEVVQAKNGTGSATLSMAYAGARMANSVLEATVGGKKGVVEPSFVKSDVFAKEGVEYFSTNIELGPEGVEKINGLGEISDYEKELIAKAIPELKKNIVKGNDFVQ

>Zygosaccharomyces_bailii_fm

MFTRIARRAFSTSKVNPYKVTILGAGGGIGQPLSLLLKLNHKVTDLRLYDLKGAPGVAADLSHIPTNSKVSGFTPDNEGLSKALKDADVVLIPAGVPRKPGMTRDDLFSINASIVRDLAKAAGDAAPNANILVISNPVNSTVPIVAETLKKKGVYNPHKLFGVTTLDSIRAARFISEVEGTDPTTEHVNVVGGHSGITIVPLISQTKYKTMDKTTREALIHRIQFGGDEVVKAKDGAGSATLSMAQAGATFANAVLSGLGGERDVIASSYVDSPLYKSEGVEFFASPVTLGPDGISKVHPVGDISSEEEQLLEKAKETLKKNITKGFAFIDKE

>Hanseniaspora_osmophila_fm

MFSTKALNKSTQRLFSTTNPTNFKVTVLGAGGGIGQPLSLLMKLNHKVTDLRLYDLRGAKGVATDLSHIPTNSTVQGFTPEESDGLKNALDNTDVVLIPAGVPRKPGMTRDDLFSINAGIVRDLAKAAAQYCPNAAICVISNPVNSTVPIVAEVFKKSGVYNPKKLFGVTTLDSIRASRFISEVEGTNPTQEKVTVVGGHSGVTILPLISQTKHSMMPKETREKLIHRIQFGGDEVVQAKNGAGSATLSMAQAGALFAGKILQGLDGEADVITPSFVDSPLFKNEGIEFFSSPITLGPEGAEKIHSLGNLSSEEEEMLATCKETLVKNIAKGKKFIDDSKL

>Wickerhamomyces_ciferrii_fm

MFSRIAKRSFSSSAAQNYKVSVLGAGGGIGQPLSLLLKLNHRVTKLALYDLKGAPGVAADISHIPTDSKVEGFTPENDGLKNALTGTDVVLIPAGVPRKPGMTRDDLFNINASIVRDLASAVAEHAPNANVLVISNPVNSTVPIVREVFKQKNVYNKNRLFGVTTLDVLRASRFLSEVVGTNPANERVSVVGGHSGITIVPLLSQTTHKDLKEDVRDALIHRIQFGGDEVVKAKDGAGSATLSMAQAGARFAGAVLKGLDGEKDIIEPTFVENPIFQNEGIDFFATEVTLGPEGVSKIHGLGEISSHEEELISKAKEALVKNIAKGQTFVTEK

>Talaromyces_marneffei_fm

MFAARQSFGAFQKRAFSASARQASKVTVLGAAGGIGQPLSLLMKLNPRVSQLALYDIRGGPGVAADLSHINTNSTVTGYDPTPSGLSEALKDAEIVLIPAGVPRKPGMTRDDLFNTNASIVRDLAKATADAAPNAKILVISNPVNSTVPIVAEVFKSKGVYNPKRLFGVTTLDVVRASRFISQVKTTDPANEEVPVVGGHSGVTIVPLLSQSNHADIEGETRDALVNRIQFGGDEVVKAKDGAGSATLSMAFAGARFAESLLKAAQGVQGVIEPTFVDSPLYKDQGIDFFASRVELGPDGVKEILPVGQVNAYEEKLLEACLADLKKNIKKGVDFVAQNP

>Aspergillus_flavus_fm

MFAARQSFTLLQKRAFSASASQASKVAVLGAAGGIGQPLSLLLKLNPRVSELALYDIRGGPGVAADLSHINTNSTVSGYEATPSGLRDALKGSEIVLIPAGVPRKPGMTRDDLFNTNASIVRDLAKAAAEASPEANILVISNPVNSTVPIVSEVFKSKGVYNPKRLFGVTTLDVVRASRFISQVQKTDPSNEAVTVVGGHSGVTIVPLLSQSSHPSIEGKTRDELVNRIQFGGDEVVKAKDGAGSATLSMAMAGARMAESLLKAAQGEKGVVEPTFVDSPLYKDQGVDFFASKVELGPNGVEKILPVGQVNAYEEKLLEACLGDLKKNIQKGIDFVKANP

>Aspergillus_lentulus_fm

MFAARQSFNLLQKRAFSASASQASKVAVLGAAGGIGQPLSLLLKLNPRVSQLALYDIRGGPGVAADLSHINTNSTVTGYDSTPSGLRDALKGSEIVLIPAGVPRKPGMTRDDLFNTNASIVRDLAKAAAEASPEANILVISNPVNSTVPIVAEVFKSKGVYNPKRLFGVTTLDVVRASRFISQIKKTDPAKEAVPVVGGHSGVTIVPLLSQSNHPDIEGETRDALVNRIQFGGDEVVKAKDGAGSATLSMAMAGARFAESLLKAAQGEKGVIEPTFVESPLYKDQGVDFFASRVELGPNGVEKILEVGKVNAYEEKLIQAALTDLKKNIQKGRDFVAQNP

>Penicillium_patulum_fm

MFAARRTVNLFQKRAFSASAINASKVAVLGAAGGIGQPLSLLLKLNPRVSELALYDIRGGPGVAADLSHINTNSTVTGYNPDASGLRDCLSGAEIILIPAGVPRKPGMTRDDLFNTNASIVRDLAKAAAEAAPKAHVLVIANPVNSTVPIVAEVYKARNVYDPKRLFGVTTLDVVRASRFISQVQSTNPADEAVPVVGGHSGVTIVPLLSQSNHSSIAGQARDALVNRIQFGGDEVVKAKDGAGSATLSMAMAGARFAESLLRAAQGEKGVIEPTFVDSPLYKDQGIDFFASRVELGPNGVEKINSVGEVNEYEQGLLDACLADLKKNIQKGVDFVKANP

>Emmonsia_sp._CAC-2015a_fm

MFAARRAFGLAQRRAFSVSAPKASKVTVLGAAGGIGQPLSLLMKLNPRVSQLALYDIRGGPGVAADLSHINTNSTVTGYDPTPSGLREALKDAEIVLIPAGVPRKPGMTRDDLFNTNASIVRDLAKAAAEASPNANILVISNPVNSTVPIVAEIFKSKNVYNPKRLFGVTTLDVVRASRFISQVKGTDPANEKVTVVGGHSGVTIVPLVSQSNHPDIAGEKLDTLVNRIQFGGDEVVKAKDGAGSATLSMAMAGARFAESLLKASQGVKDVIEPTFVESPLFKDQGINFFASNVRLGPNGVEEILPIGKISAHEQKLVDACLVDLKKNIAKGVEFVKSNP

>Exophiala_xenobiotica_fm

MSFFARQMFGQVQRRAFSATARQPSKVAILGAAGGIGQPLSLLMKLNPRVSQLALYDIKLGPGVAADISHINTNSTVKGYDPTPSGLKECLKGSEIILIPAGVPRKPGMTRDDLFNTNATIVRDLAKAAAQAAPEAKLLIISNPVNSTVPICAEVYKNAGVYNPKTLFGVTTLDVVRASRFVSEVKGTDPANENITVVGGHSGITIVPLISQSGHSDITGEKLDALVNRIQFGGDEVVKAKDGAGSATLSMAMAGARFAESLLKASQGEKGVVECTFVDSPLYKDQGVEFFASKVELGPDGVQKIHEVGKLSKQEEQMLEACLGDLKKNIQKGIDFVKANP

>Fusarium_oxysporum_Fo47_fm

MFAASRIQRRAFSATARDLSKVTVLGAAGGIGQPLSLLLKMNPRVTDLALYDIRGGPGVAADISHVNTKSTVKGYEPNAAGLKDALSGAEVVLIPAGVPRKPGMTRDDLFNTNASIVRDLAKAAAQAAPKAKLLIISNPVNSTVPIVKEVFKAEGVYNPKTLFGVTTLDVVRASRFVSEIKGTDPKDENITVVGGHSGVTIVPLFSQSNHPDLSSNAELVKRVQFGGDEVVKAKDGAGSATLSMAMAGARMADSILRAVQGEKGVIEPSFVESPLYKDQGIEFFSSKVELGPEGVEKIHPLGKLDANEEKLVEAALVDLKKNIEKGVAFVASNPPK

>Neonectria_ditissima_fm

MFAASKLQRRAFSVSARNLSKVTVLGAAGGIGQPLSLLLKLNPRVTELALYDIRGGPGVAADISHVNTKSSVKGYEPSPSGLASALKGAEVVLIPAGVPRKPGMSRDDLFNTNASIVRDLAKAAAESSPDAKVLVIANPVNSTVPIVAEVYKSKGVYNPKKLFGVTTLDVVRASRFVSEIKDTDPKDENVTVIGGHSGVTIVPLFSQSNHPDLSSNAELLKRVQFGGDEVVQAKDGAGSATLSMAMAGARMADSLLRAGQGEKGVVEPSFVESPLYKDQGVTFFSSKVELGPNGVEKIHDIGKVDATEEKLIEACLGDLKKNIEKGVKFVAENPGN

>Beauveria_bassiana_D1-5_fm

MFAASRIQTRAFSASARNLSKVAVLGAAGGIGQPLSLLLKLNPRVTDLALYDIRGGPGVAADISHVNTKSTVTGYDPTPEGLAACLKDAEIVLIPAGVPRKPGMTRDDLFNTNASIVRDLAKAVAQSAPKAKTLVIANPVNSTVPICAEVFKAKGVYNPKTLFGVTTLDVVRASRFVSALKGTDPKDENITVVGGHSGVTIVPLFSQSNHPDLSSNADLIKRVQFGGDEVVKAKDGAGSATLSMAMAGARMADSLLRAAAGEKVIEPTFVESPLYKDQGIEFFSSKVELGPDGVKEILPVGKVDATEQGLLDACIVDLKKNIEKGVSFVAQNPGN

>Colletotrichum_orbiculare_fm

MSFIQRRMFSASARNLSKVTVLGAAGGIGQPLSLLMKLNPQVTELALYDIRGGPGVAADISHVNTKSTVKGFDPTPTGLANALKGSEIVLIPAGVPRKPGMTRDDLFNTNASIVRDLAKACAESCPDANILVISNPVNSTVPIVSEVFKAHGVYNPKRLFGVTTLDVVRASRFVSEIKNTDPKDENITVVGGHSGVTIVPLFSQSNHPDLSSNAELVNRVQFGGDEVVKAKDGAGSATLSMAFAGARMAESLLRASKGEKGIVEPTFVDSPLYKDQGIDFFSSKVELGPNGVEKIHPLGKVDAAEEKLLEACFADLKKNIAKGVSFVAQNPPK

>Verticillium_alfalfae_fm

MFAPRIQRRMFSATARDLSKVTVLGAAGGIGQPLSLLLKLNPRVTELALYDIRGGPGVAADISHVNTKSKVTGYEATPAGLAAALKGADVVLIPAGVPRKPGMTRDDLFNTNASIVRDLAKAAADSAPEANLLIIANPVNSTVPICAEVYKKAGVYNPKRLFGVTTLDVVRASRFVSEIKGTDPKDEDITVVGGHSGVTIVPLFSQSNHPDLSSNEALINRVQFGGDEVVKAKDGAGSATLSMAMAGARMAESLLRASQGEKGIVEPTFVDSPLYKDQGIDFFSSKVELGPNGVEKIHPVGSVDAVEERLLEACFGDLKKNIAKGVAFVASNPPK

>Diaporthe_helianthi_fm

MFAAPRMASMCQRRAFSASARQLSKVTVLGAAGGIGQPLSLLLKLNPRVTELALYDIRMAPGVAADISHINTKSTVTGYDPTSSGLSDALKGAEIVLIPAGVPRKPGMTRDDLFNTNASIVRDLAKAAAEASPDANILVISNPVNSTVPIVAEVFKARGVYNPKRLFGVTTLDVVRASRFVSQLKGTDPADENITVVGGHSGVTIVPLFSQSAHPDLSSHAEIVNRVQFGGDEVVKAKDGAGSATLSMAMAGARMAESLLRAAQGEKGVVEPTFVDSPLYKDQGIDFFSSKVELGPNGVEKILPLGKIDANEEKMVEACLADLKKNIEKGVAFVAKNPGN

>Pseudogymnoascus_sp._VKM_F-4519_fm

MFAARTFSAAQRRAFSASARDLSKVTVLGAAGGIGQPLSLLLKLNPRVTELALYDIRGGPGVAADISHINTGSNVTGYEPTPSGLAAALKDAEIVLIPAGVPRKPGMTRDDLFNTNASIVRDLAKAAAQSAPNANILVIANPVNSTVPIVAEVFKKAGVYNPKRLFGVTTLDVVRASRFVSEIKKTDPADEAITVVGGHSGVTIVPLFSQSKHADLVGDAALLNRVQFGGDEVVKAKDGAGSATLSMAMAGARFAESLLKAAQGEKGVTEPTFVDSPLYKDQGVDFFASKVELGPSGVEKIHEVGKISAEEQKLLDAALADLKKNIEKGVQFVATNPGN

>Verruconis_gallopava_fm

MSFARQVFAQVQRRTFSASARQNSKVAVLGAAGGIGQPLSLLLKLNPRVSELALYDIRMAPGVAADVSHINTKSKVVGYDPTPSGLRECLTGAEIVLIPAGVPRKPGMTRDDLFNTNASIVRDLAKAAAEHSPEANMLIISNPVNSTVPITAEVFKAKGVYNPKRLFGVTTLDVVRASRFISEIKGTDPATENITVVGGHSGQTIVPLLSQSGNKLEGEALDKYIHRVQFGGDEVVQAKGGAGSATLSMAMAGARFAESLLKAAQGEKGVLEPTFVDSPLYKDRGCDFFASLVELGPNGVEKIHPVGKITDYEQKLLDVCLGDLAKNITKGVEWARANPGN

>Diplodia_corticola_fm

MFAARQVFGAAQRRAFSISARQNSKVTVLGAAGGIGQPLSLLLKLNPRVSELALYDIRGGPGVAADVSHINTKSNVKGYDPTPSGLRECLEGSEIVLIPAGVPRKPGMTRDDLFNTNASIVRDLAKATADAAPNANVLIISNPVNSTVPITAEVFKSKGVYNPKRLFGVTTLDVVRASRFISQLKGSDPANENITVIGGHSGATIVPLLSQAGHKLEGAELDEYVRRVQFGGDEVVQAKGGAGSATLSMAMAGARFAESLLRAAQGEKGVIEPTFVDSPLYKDQGCDFFASNVELGPNGVEKILPVGDVTAYEQKLLEVCVQDLAKNIKKGVDFAKQNP

>Zymoseptoria_tritici_fm

MFAARATKSFMGAAVQRRAFSASASNLSKVVVLGAGGGIGQPLSLLLKLNPRVTELALYDIKGAPGVAADVGHINTKSTVTGYSPDGEGLGACLKGAEIVVIPAGVPRKPGMTRDDLFNTNASIVRDLAKAAAKHCPEASLLVISNPVNSTVPITAEVFKAAGVYNPKKLFGVTTLDVVRASRFISQIKSSDPANEKVTVVGGHSGETIVPLLSQAGHKLEGDELANYIKRVQFGGDEVVKAKDGAGSATLSMAMAGARFTESLLKAAQGEKNVKEETYVDSPLYKDQGVNYFSSTVTLGPNGVEEIHPVGKITEHEQGLLDVCLKDLKKNIEKGEQWVKENP

>Aureobasidium_pullulans_fm

MFAARQVIGTVQRRAFSATARDASKVAVLGAGGGIGQPLSLLLKLNPRVTELALYDVRGAPGVAADCSHVNTKSVVKGYEQADVAEALKNSEIVLIPAGVPRKPGMTRDDLFNTNASIVRDLAKAAAEHCPKANILIISNPVNSTVPIAAEVFKAAGVYDPKRMFGVTTLDVVRASRFISQLKSTDPASENIVVVGGHSGATIVPLLSQSGYNLEGEQLDAYVKRVQFGGDEVVQAKGGAGSATLSMAMAGARFAESLLKAAQGEKGVIEPTFVDSPLYKDQGVDFFASQVELGPNGVEKIHPVGQVTDYEQKLLDACLKDLKGNIEKGVKFAKENP

>Cryptococcus_gattii_serotype_b_fm

MFARQVAKNSSSLARGFASSARSNRKVAVLGAAGGIGQPMSLLLKQNPGVTALSLYDIRGAPGVAADISHVNTHSTVKGFEKDDIKEALTGAEIVIIPAGVPRKPGMTRDDLFNTNASIVRDLAEACAEYCPKAFIGVIANPVNSTVPIFAEVYKKKGIFDEKRIFGITTLDVVRASRFLGEVKGKDPKDVKVTVVGGHSGVTIVPLLSQTPEGKDVSGEAYKALVHRIQFGGDEVVKAKAGTGSATLSMGYAGARFTDSLIRALNGETGVVEPTFVKSPLYESEGVEYFASNVELGPEGVKKINPVGQLSAEEQELLKACLPDLAKNIKKGVDFVKA

>Cryptococcus_neoformans_var._grubii_fm

MFARQVAKNSSSLARGFASSARSNKKVAVLGAAGGIGQPMSLLLKQNPGVTGLSLYDIRGAPGVAADISHVNTHSIVKGFEKDDIKEALTGAEIVIIPAGVPRKPGMTRDDLFNTNASIVRDLAEACAEYCPKAFIGIISNPVNSTVPIFAEVLKKKGVFDEKRVFGITTLDVVRASRFLGEIKGKDPKDIKVTVVGGHSGVTIVPLLSQTPEGKDVSGEAYKALVNRIQFGGDEVVKAKAGTGSATLSMGYAGARFTDSLIRALNGETGIVEPTFVKSPLYESEGVEYFASNVELGPEGVKKINPVGQLSAEEQELLKACLPDLAKNIKKGVDFVKA

>Kwoniella_mangroviensis_CBS_10435_fm

MFSRQVARSSSALARGFASSARSNRKVAVLGAAGGIGQPMSLLLKTDPLVTGLSLYDIRGAPGVAADISHVNTHSEVKGYEKDDIKAALTGAEVVIIPAGVPRKPGMTRDDLFNTNASIVRDLAEACAEYCPKAYIGIISNPVNSTVPIFAEVYKKKGVFDPKRLFGVTTLDVVRSSRFLGEIKGADPKDIKVTVVGGHSGATIVPILSHTAQGKDVSGETYKNLVHRIQFGGDEVVKAKAGTGSATLSMGFAGARFTNSLIRALNGESGVVEPTFVKSPLYESEGVEYFASNVELGPEGVKKINPVGELTAEEQELLKACLPDLAKNIKKGVEFVNKA

>Bifiguratus_adelaidae_fm

MFVTTRSIASTAKRAFSTSAVSNAAKVAVLGAAGGIGQSLSLLLKQNPNISDLALYDIVNTPGVAADISHINTNSKVTGYVPENNGLAKALEGSNVVVIPAGVPRKPGMTRDDLFNTNASIVQGLADACAQYCPTAHILIISNPVNSTVPIVAETFKARGVYDPKRLFGVTSLDVVRASRFVSEVKGGDPKDIHITVVGGHSGVTIVPLLSQSGKSFTQEELDALTHRIQFGGDEVVKAKDGKGSATLSMAFAGARFANSVLEATVGGKRGIVEPTFVESPVFADQGVEFFATNVELGANGVEKIHPLGKLSAYEEKLVEAALPELKKNIAKGKSFVASKQ

>Homo_sapiens_ac

MSEPIRVLVTGAAGQIAYSLLYSIGNGSVFGKDQPIILVLLDITPMMGVLDGVLMELQDCALPLLKDVIATDKEDVAFKDLDVAILVGSMPRREGMERKDLLKANVKIFKSQGAALDKYAKKSVKVIVVGNPANTNCLTASKSAPSIPKENFSCLTRLDHNRAKAQIALKLGVTANDVKNVIIWGNHSSTQYPDVNHAKVKLQGKEVGVYEALKDDSWLKGEFVTTVQQRGAAVIKARKLSSAMSAAKAICDHVRDIWFGTPEGEFVSMGVISDGNSYGVPDDLLYSFPVVIKNKTWKFVEGLPINDFSREKMDLTAKELTEEKESAFEFLSSA

>Rattus_norvegicus_ac

MSEPIRVLVTGAAGQIAYSLLYSIGNGSVFGKDQPIILVLLDITPMMGVLDGVLMELQDCALPLLQDVIATDKEEVAFKDLDVAVLVGSMPRREGMERKDLLKANVKIFKSQGAALEKYAKKSVKVIVVGNPANTNCLTASKSAPSIPKENFSCLTRLDHNRAKSQIALKLGVTADDVKNVIIWGNHSSTQYPDVNHAKVKLQGKEVGVYEALKDDSWLKGEFITTVQQRGAAVIKARKLSSAMSAAKAISDHIRDIWFGTPEGEFVSMGVISDGNSYGVPDDLLYSFPVVIKNKTWKFVEGLPINDFSREKMDLTAKELTEEKETAFEFLSSA

>Mus_musculus_ac

MSEPIRVLVTGAAGQIAYSLLYSIGNGSVFGKDQPIILVLLDITPMMGVLDGVLMELQDCALPLLQDVIATDKEEIAFKDLDVAVLVGSMPRREGMERKDLLKANVKIFKSQGTALEKYAKKSVKVIVVGNPANTNCLTASKSAPSIPKENFSCLTRLDHNRAKSQIALKLGVTADDVKNVIIWGNHSSTQYPDVNHAKVKLQGKEVGVYEALKDDSWLKGEFITTVQQRGAAVIKARKLSSAMSAAKAIADHIRDIWFGTPEGEFVSMGVISDGNSYGVPDDLLYSFPVVIKNKTWKFVEGLPINDFSREKMDLTAKELTEEKETAFEFLSSA

>Bos_taurus_ac

MSEPIRVLVTGAAGQIAYSLLYSIGNGSVFGKDQPIILVLLDITPMMGVLDGVLMELQDCALPLLKDVIATDKEEIAFKDLDVAILVGSMPRRDGMERKDLLKANVKIFKCQGAALDKYAKKSVKVIVVGNPANTNCLTASKSAPSIPKENFSCLTRLDHNRAKAQIALKLGVTSDDVKNVIIWGNHSSTQYPDVNHAKVKLQGKEVGVYEALKDDSWLKGEFITTVQQRGAAVIKARKLSSAMSAAKAICDHVRDIWFGTPEGEFVSMGIISDGNSYGIPDDLLYSFPVTIKDKTWKVVEGLPINDFSREKMDLTAKELAEEKETAFEFLASA

>Sus_scrofa_ac

MSEPIRVLVTGAAGQIAYSLLYSIGNGSVFGKDQPIILVLLDITPMMGVLDGVLMELQDCALPLLKDVIATDKEEIAFKDLDVAILVGSMPRRDGMERKDLLKANVKIFKCQGAALDKYAKKSVKVIVVGNPANTNCLTASKSAPSIPKENFSCLTRLDHNRAKAQIALKLGVTSDDVKNVIIWGNHSSTQYPDVNHAKVKLQAKEVGVYEAVKDDSWLKGEFITTVQQRGAAVIKARKLSSAMSAAKAICDHVRDIWFGTPEGEFVSMGIISDGNSYGVPDDLLYSFPVTIKDKTWKIVEGLPINDFSREKMDLTAKELAEEKETAFEFLSSA

>Gallus_gallus_ac

MGEPIRVLVTGAAGQIAYSLLYSIAKGDVFGKEQPLVLVLLDITPMMTVLEGVVMELQDCALPLLREVIPTDKEEVAFKDLDIAILVGSMPRREGMERKDLLKANVKIFKSQGAALDKYAKKTVKVVVVGNPANTNCLIASKSAPSIPKENFSCLTRLDHNRAKSQIALKLGVTSNDVKNVIIWGNHSSTQYPDVNHAKVNVKGKEVGVYEAIKDDSWLKGDFILTVQQRGAAVIKARKLSSAMSAAKAICDHVRDIWFGTPAGEFVSMGVISDGNSYGVPEDLLYSFPVVIKDKTWKFVEGLPINDFSREKMDLTAKELTEEKETAVEFLSSA

>Danio_rerio_ac

MAEPIRVLVTGAAGQIAYSLLYSIAKGDVFGKDQPIILVLLDITPMLPVLDGVVMELQDCALPLLREVIPTDKVEVGFKDLDAAILVGSMPRKEGMERKDLLKANVAIFKTQGEALEKYAKKTVKVLVVGNPANTNCLIASKSAPSIPKENFSCLTRLDHNRARSQVAMRVGVPSDSVKNVTIWGNHSSTQYPDVHHAIVTRNGKEIAAFDAVNDESWLKGDFISTVQQRGAAVIKARKLSSAMSAAKAICDHMRDIWFGTPDGEWVSMGIYSSGNSYGVPDDLMYSFPVKIKNKSWKVVDGLSINDFSRGKMDATAAELVEERDTALTFLSA

>Drosophila_ananassae_ac

MAEPIRVVVTGAAGQIAYSLLYMVARGEVFGKDQPIILHLLDIPPMVGVLEGVVMELADCALPLLVEVVPTTDPAVGFKDVSAAFLVGAMPRKEGMERKDLLSANVKIFRTQGQALDKFAKKDVKVLVVGNPANTNALVCSSYAPSIPRENFSAMTRLDQNRATSQIAAKVGVPISAVNNIIIWGNHSSTQYPDAGQGKVVVNGAVKSVVDAVNDASYLQGAFVETVQKRGAAVIAARKMSSAMSAAKAACDHMHDWWNGTAPGKFVSMGVFSDGSYNSPKDVIFSFPVEIKNKQWKIVEGLTLSDFAKSKLSITGKELQEEKDEALSVLDSNVSNL

>Anoplophora_glabripennis_ac

MSEPIRVVVTGAAGQIAYSLLYMVAKGDVFGPNQPLILHLLDIPPMMGVLEGVVMELADCALPLLQAVVPTADPAVAFKDVSSAFLVGSMPRREGMERKDLLSANVKIFKVQGEALDKYAKKDVKVLVVGNPANTNALVCSRYAPSIPKENFTAMTRLDQNRAQAQIAAKVGVPVAQVSNVIIWGNHSSTQFPDAAHAVVVVDGKEVKASEVVKDSDWIRNTFVQTVQKRGAAVIGARKMSSAMSAAKAACDHMKDWFSGTAPGKFVSMGVISDGSYGAPKDVVFSFPVSIQNGKWQIAQGLLLDDFAKEMLATTGKELEEERAEATAVVEA

>Homo_sapiens_am

MLSALARPASAALRRSFSTSAQNNAKVAVLGASGGIGQPLSLLLKNSPLVSRLTLYDIAHTPGVAADLSHIETKAAVKGYLGPEQLPDCLKGCDVVVIPAGVPRKPGMTRDDLFNTNATIVATLTAACAQHCPEAMICVIANPVNSTIPITAEVFKKHGVYNPNKIFGVTTLDIVRANTFVAELKGLDPARVNVPVIGGHAGKTIIPLISQCTPKVDFPQDQLTALTGRIQEAGTEVVKAKAGAGSATLSMAYAGARFVFSLVDAMNGKEGVVECSFVKSQETECTYFSTPLLLGKKGIEKNLGIGKVSSFEEKMISDAIPELKASIKKGEDFVKTLK

>Rattus_norvegicus_am

MLSALARPVGAALRRSFSTSAQNNAKVAVLGASGGIGQPLSLLLKNSPLVSRLTLYDIAHTPGVAADLSHIETRANVKGYLGPEQLPDCLKGCDVVVIPAGVPRKPGMTRDDLFNTNATIVATLTAACAQHCPEAMICIISNPVNSTIPITAEVFKKHGVYNPNKIFGVTTLDIVRANTFVAELKGLDPARVNVPVIGGHAGKTIIPLISQCTPKVDFPQDQLATLTGRIQEAGTEVVKAKAGAGSATLSMAYAGARFVFSLVDAMNGKEGVIECSFVQSKETECTYFSTPLLLGKKGLEKNLGIGKITPFEEKMIAEAIPELKASIKKGEDFVKNMK

>Mus_musculus_am

MLSALARPAGAALRRSFSTSAQNNAKVAVLGASGGIGQPLSLLLKNSPLVSRLTLYDIAHTPGVAADLSHIETRANVKGYLGPEQLPDCLKGCDVVVIPAGVPRKPGMTRDDLFNTNATIVATLTAACAQHCPEAMVCIIANPVNSTIPITAEVFKKHGVYNPNKIFGVTTLDIVRANTFVAELKGLDPARVNVPVIGGHAGKTIIPLISQCTPKVDFPQDQLATLTGRIQEAGTEVVKAKAGAGSATLSMAYAGARFVFSLVDAMNGKEGVVECSFVQSKETECTYFSTPLLLGKKGLEKNLGIGKITPFEEKMIAEAIPELKASIKKGEDFVKNMK

>Bos_taurus_am

MLSALARPAGAALRRSFSTSAQNNAKVAVLGASGGIGQPLSLLLKNSPLVSRLTLYDIAHTPGVAADLSHIETRATVKGYLGPEQLPDCLKGCDVVVIPAGVPRKPGMTRDDLFNTNATIVATLTAACAQHCPEAMICIISNPVNSTIPITAEVFKKHGVYNPNKIFGVTTLDIVRANAFVAELKDLDPARVNVPVIGGHAGKTIIPLISQCTPKVEFPQDQLTTLTGRIQEAGTEVVKAKAGAGSATLSMAYAGARFVFSLVDAMNGKEGVVECSFVKSQETDCPYFSTPLLLGKKGIEKNLGIGKVSPFEEKMIAEAIPELKASIKKGEEFVKNMK

>Sus_scrofa_am

MLSALARPAGAALRRSFSTSXQNNAKVAVLGASGGIGQPLSLLLKNSPLVSRLTLYDIAHTPGVAADLSHIETRATVKGYLGPEQLPDCLKGCDVVVIPAGVPRKPGMTRDDLFNTNATIVATLTAACAQHCPDAMICIISNPVNSTIPITAEVFKKHGVYNPNKIFGVTTLDIVRANAFVAELKGLDPARVSVPVIGGHAGKTIIPLISQCTPKVDFPQDQLSTLTGRIQEAGTEVVKAKAGAGSATLSMAYAGARFVFSLVDAMNGKEGVVECSFVKSQETDCPYFSTPLLLGKKGIEKNLGIGKISPFEEKMIAEAIPELKASIKKGEEFVKNMK

>Gallus_gallus_am

MRGAVEVVGVTAAAMLSRLARPAAVLCRGLATSAQNNAKVAVLGASGGIGQPLSLLLKNSPLVSRLTLYDIAHTPGVAADLSHIETRANVKGFLGPEQLPECLKGCDVVVIPAGVPRKPGMTRDDLFNTNASIVATLTTACAKHCPEAMICIISNPVNSTIPITSEVFKKHGVYNPNRIFGVTTLDIVRANTFVAELKGLDPARVSVPVIGGHAGKTIIPLISQCTPKVDFPQDQLEKLTGRIQEAGTEVVKAKAGAGSATLSMAYAGARFVFSLVDAMNGKEGVIECSFVRSEETESPYFSTPLLLGKNGIEKNLGIGKITPFEEKMVAEAMAELKASIKKGEDFAKNFK

>Danio_rerio_am

MFSRVARPTASLVRSLSTSSQNNAKVAVLGASGGIGQPLSLLLKNSPLVSELSLFDIAHTPGVAADLSHIETRAHVKGYIGADQLGDALKGCEVVVIPAGVPRKPGMTRDDLFNTNATIVATLVDGCARHCPQAMICIISNPVNSTIPITSEVMKKHGVYNPNKIFGVTTLDIVRANTFVAELKGLDPARVNVPVVGGHAGITIIPLISQCTPKVEFPADQLSALTGRIQEAGTEVVKAKAGAGSATLSMAYAGARFTFSLLDAMNGKEGVVECSFVRSEETECKYFSTPLLLGKNGIEKNLGLGKLSAFEEKLVADAMTELKGSIKKGEDFVANMK

>Drosophila_ananassae_am

MLKQVTKQLALQGVRNFSVSQQNNYKVTVCGASGGIGQPLSLLLKQNPLVTDLALYDIVHTPGVAADLSHIDTKSKTAGFMGADQLGDSLKGSDVVVIPAGVPRKPGMTRDDLFNVNAGIIKDISNSIAKNCPKALVAIITNPVNTCVPIAAEILKKAGVYDPKRLFGVSTLDVVRARAFIGHALGVDPQTVQIPVIGGHSGVTILPVLSQSQPQFKGNQDTIEKLTVRIQEAGTEVVKAKAGAGSATLSMAYAGARFAGSLLKGLNGEKNVIECSYVQSTITEATFFSTPLVLGKNGLQENLGLPKLNDYEKKLLEAAIPELKKNIQKGIDFANA

>Anoplophora_glabripennis_am

MFSRVVRPTLVSARNFSTSKQNNYKVAVCGAAGGIGQPLSLLLKINPLVSELSLYDIVHTPGVAADLSHIETAAKVKGFNGPENLLEALKNADVIIIPAGVPRKPGMTRDDLFNTNAGIVRDLAKAAAEAAPKALIGIITNPVNSAVPIACEVLKKAGKLDPKRIFGISTLDIVRANTFIAEAKGLNPKDVNVPVIGGHSGVTIIPLISRSTPSVSFPQEQLKALTTRIQEAGTEVVKAKAGAGSATLSMAYAGARFANSLLRGLKGEPNVIEPAYVISDVTEAPYFSTPLILGKNGIEKNLGLGTLSDFEKDLLKAAIPELKKNIEAGVKFANQ

>Arabidopsis_thaliana_pc

MAKEPVRVLVTGAAGQIGYALVPMIARGIMLGADQPVILHMLDIPPAAEALNGVKMELIDAAFPLLKGVVATTDAVEGCTGVNVAVMVGGFPRKEGMERKDVMSKNVSIYKSQAAALEKHAAPNCKVLVVANPANTNALILKEFAPSIPEKNISCLTRLDHNRALGQISERLSVPVSDVKNVIIWGNHSSSQYPDVNHAKVQTSSGEKPVRELVKDDAWLDGEFISTVQQRGAAIIKARKLSSALSAASSACDHIRDWVLGTPEGTFVSMGVYSDGSYSVPSGLIYSFPVTCRNGDWSIVQGLPIDEVSRKKMDLTAEELKEEKDLAYSCLS

>Brassica_napus_pc

MAKEPVRVLVTGAAGQIGYALVPMIARGIMLGADQPVILHMLDIPFAAEALNGVKMELIDAAFPLLKGVVATTDAVEGCTGVNVAVMVGGFPRKEGMERKDVMSKNVSIYKSQAAALEKHAAPNCKVLVVANPANTNALILKEFAPSIPEKNITCLTRLDHNRALGQISERLSVPVSDVKNVIIWGNHSSTQYPDVNHAKVQTSSGEKPVRELVKNDEWLNGEFITTVQQRGAAIIKARKLSSALSAASSACDHIRDWVLGTPEGTFVSMGVYSDGSYNVPSGLIYSFPVTCRNGEWDIVQGLPIDEVSRKKMDLTAEELKEEKDLAYSCLS

>Prunus_persica_pc

MAKGPVRVLVTGAAGQIGYALVPMIARGVMLGADQPVILHLLDIPPAAEALNGVKMELVDAAFPLLKGVVATTDVVEACTGVNIAVMVGGFPRKEGMERKDVMSKNVSIYKSQASALEKHAAANCKVLVVANPANTNALILKEFAPSIPEKNITCLTRLDHNRALGQVSERLNVQVSDVKNVIIWGNHSSSQYPDVNHATVKTPSGEKAVRELVADDAWLTGEFITTVQQRGAAIIKARKLSSALSAASSACDHIRDWVLGTPEGTWVSMGVYSDGSYNVPSGLIYSFPVTCQNGEWKIVQGLSIDEFSRKKLDATADELSEEKALAYSCLS

>Medicago_sativa_pc

MAKDPVRVLVTGAAGQIGYALVPMIARGVMLGPDQPVILHMLDIAPAAESLNGVKMELVDAAFPLLKGVVATTDVVEACTGVNIAVMVGGFPRKEGMERKDVMSKNVSIYKSQASALEKHAAANCKVLVVANPANTNALILKEFAPSIPERNISCLTRLDHNRALGQISERLNVQVSDVKNVIIWGNHSSTQYPDVNHATVNTPAGEKPVRQLVSDDAWLNGEFISTVQQRGAAIIKARKLSSALSAASAACDHIRDWVLGTPQGTFVSMGVYSDGSYNVPSGLIYSFPVTCANGEWKIVQGLSIDEFSRKKLDLTAEELTEEKNLAHSCLS

>Zea_mays_pc

MAKEPMRVLVTGAAGQIGYALVPMIARGVMLGADQPVILHMLDIPPAAEALNGVKMELVDAAFPLLKGVVATTDVVEACTGVNVAVMVGGFPRKEGMERKDVMSKNVSIYKSQASALEAHAAPNCKVLVVANPANTNALILKEFAPSIPEKNVTCLTRLDHNRALGQISERLNVQVSDVKNVIIWGNHSSSQYPDVNHATVKTSTGEKPVRELVSDDEWLNGEFITTVQQRGAAIIKARKFSSALSAASSACDHIRDWVLGTPEGTFVSMGVYSDGSYGVPSGLIYSFPVTCSGGEWKIVQGLPIDEFSRKKMDATAQELTEEKTLAYSCLE

>Oryza_sativa_subsp._japonica_pc

MAKEPMRVLVTGAAGQIGYALVPMIARGVMLGADQPVILHMLDIPPATESLNGLKMELVDAAFPLLKGIVATTDVVEACTGVNVAVMVGGFPRKEGMERKDVMSKNVSIYKSQASALEAHAAPNCKVLVVANPANTNALILKEFAPSIPEKNITCLTRLDHNRALGQISEKLNVQVTDVKNAIIWGNHSSTQYPDVNHATVKTPSGEKPVRELVADDEWLNTEFISTVQQRGAAIIKARKQSSALSAASSACDHIRDWVLGTPEGTFVSMGVYSDGSYGVPAGLIYSFPVTCSGGEWTIVQGLPIDEFSRKKMDATAQELSEEKTLAYSCLN

>Chlorella_variabilis_pc

MAPEPKRILVTGAAGQIGYAICPMIARGAMLGPDQPVILHMLDIEPAKQALEGVRMELVDAAYPLLMGVVASTDAEEACKGVDVAVMVGGFPRKAGMERKDVMSKNVAIYKAQASALEKNAAPGCKVLVVANPANTNSLILKEHAPSIPAENITCLTRLDHNRALGQLSERSGVHVGKVKNVIIWGNHSSTQYPDVNHATIDGKPAREVIGDDAYLDGDFISTVQQRGAAIIKARGLSSALSAASSACDHIRDWVLGTPEGTWVSMGVYSDGSYGAPKGVVYSFPVTCKAGTWSIVQGLSIDEPSAAKMKATGDELVEEKALAMECLGEAA

>Chlamydomonas_reinhardtii_pc

MLGADQPIILHLLDVEPAKNALEGLRMELVDGAYPLLEGVLTFTDVAAACKDVDVAVMVGGYPRKAGEERKDVMAKNVSIYQQQASALEANASKDVKVVVVANPANTNALILAENAPSIPRENITCLTRLDHNRALGQVAERTGSHVGIVKNVIIWGNHSSTQYPDVNHGTVGGKPIRSAVNDDTWLNGDFITTVQQRGAAIIKARGLSSALSAANAVCNHVRDWVRGTPGGSWTSMGVVSDGSYGVQRGLVYSYPVTCAGGKWKVVQGLPIDGPSRERLRVTEAELVEERDLALQCLAEKK

>Arabidopsis_thaliana_pm

MFRSMLVRSSASAKQAVIRRSFSSGSVPERKVAILGAAGGIGQPLALLMKLNPLVSSLSLYDIANTPGVAADVGHINTRSEVVGYMGDDNLAKALEGADLVIIPAGVPRKPGMTRDDLFNINAGIVKNLCTAIAKYCPHALINMISNPVNSTVPIAAEIFKKAGMYDEKKLFGVTTLDVVRARTFYAGKANVPVAEVNVPVIGGHAGVTILPLFSQATPQANLSSDILTALTKRTQDGGTEVVEAKAGKGSATLSMAYAGALFADACLKGLNGVPDVIECSYVQSTITELPFFASKVRLGKNGVEEVLDLGPLSDFEKEGLEALKPELKSSIEKGVKFANQ

>Brassica_napus_pm

MFRSALVRSSASAKQSLLRRSFSSGSVPERKVAILGAAGGIGQPLALLMKLNPLVSSLSLYDIANTPGVAADVGHINTRSQVVGYMGDDNLAKALEGADLVIIPAGVPRKPGMTRDDLFNINAGIVKNLWSAIAKYCPHALVNMISNPVNSTVPIAAEIFKKAGMYDEKKLFGVTTLDVVRVKTSYAGKANVPVAEVNVPAIVGHAGVTILPLFSQATPQAILSGDALTVTTKRTQDGGTEVEEAKAGKGSATLSMAYAGALFADACLKGLNGVPDVVECSYVQSTITELPFFASKVRLGKNGVEEVLDLGPLSDFEKEGLEALRPGIKSTIEKGVKFANQ

>Prunus_persica_pm

MRPSVSLVRSVQRVTRRSYSSEAVPERKVAVLGAAGGIGQPLSLLMKLNPLVSHLSLYDIAGTPGVAADVSHINTRSEVKGYAGEDQLAQALEGADVVIIPAGVPRKPGMTRDDLFNINAGIVKGLTAAIAKYCPNALINMISNPVNSTVPIAAEVLKKAGKYDDKRLFGVTTLDVVRAKTFYAGKANVNVAEVNVPVVGGHAGITILPLFSQATPTANLPHDVIKALTKRTQDGGTEVVEAKAGKGSATLSMAYAGALFADACLKGLNGVPDVVECSFVQSSVTELPFFASKVRLGRNGVEEVLGLGNLSDFEKEGLESLKPELKASIEKGIKFANQN

>Medicago_sativa_pm

MMRPSILRSVKSAVSRSITRRGYATEPVPERKVAILGAAGGIGQPLSLLMKLNPLVSTLSLYDIAGTPGVAADVSHINSRSQVTGYAGEDELGKALEGADVVIIPAGVPRKPGMTRDDLFNINAGIVKSLATAISKYCPHALVNMISNPVNSTVPIAAEVFKKAGTYDEKRLFGVTTLDVVRAKTFYAGKANVPVAEVNVPVIGGHAGVTILPLFSQASPQANLDDDVIKALTARTQDGGTEVVTAKAGKGSATLSMAYAGAIFADACLKGLNGVPDVVECSYVQSNLIAELPFFASKVRIGKNGVEEILGLGSLSDFEKQGLENLKSELKSSIEKGIKFASQ

>Zea_mays_pm

MRPSLLKSTAELLRRSRGYASSANPERKVAILGAAGGIGQPLSLLMKLNPLVSSLSLYDIAGTPGVAADVSHINSPALVKGFMGDDQLGEALEGSDIVIIPAGVPRKPGMTRDDLFNINAGIVKALCTAIAKHCPNALVNMISNPVNSTVPIAAEVFKKAGTYDEKKLFGVTTLDVVRAKTFYAGKAGVPVTEVNVPVVGGHAGITILPLFSQATPASNSLSQEDIEALTKRTQDGGTEVVEAKAGKGSATLSMAYAGAVFADACLKGLNGVPDIVECSFVQSTVTELPFFASKVRLGKNGVEEVLGLGELNDFEKKGLESLKVELKSSIDKGIKFAHGS

>Oryza_sativa_subsp._japonica_pm

MRPSLMRSASQVLRRRRGYSSASGQPERKVAILGAAGGIGQPLSLLMKLNPLVSSLSLYDIAGTPGVAADVSHINAPAQVKGFMGDDQLGEALEGSDIVIIPAGVPRKPGMTRDDLFNINAGIVKNLCTAIAKYCPNALVNMISNPVNSTVPIAAEVFKKAGTYDEKKLFGVTTLDVVRAKTFYAGKANVPVTDVNVPVVGGHAGITILPLFSQATPATNALSDEDIKALTKRTQDGGTEVVEAKAGKGSATLSMAYAGAVFANACLKGLNGVPDVVECSFVQSTVTELPFFASKVKLGKNGVEEVLGLGQLSDFEKEGLENLKGELKASIEKGIKFANA

>Chlorella_variabilis_pm

MQAIKEVVKHARAFSSVPAHFDRKVAVLGAAGGIGQPLSLLMKLSPYVSELALYDIAGTPGVAADVSHINSKATVKGYAGEDQLGEALKGADVVIIPAGVPRKPGMTRDDLFKINAGIVRALVEACGKHCPKALLNIISNPVNSTVPIAAETLKRLGVYDEKRVLGVTTLDVVRAKTFYAEKAGLDVSKVDVPVVGGHAGVTILPLFSQAVPNAANKLSEADIDALTKRTQDGGTEVVQAKAGKGSATLSMAYAGALFADACLRGLNGDPDVVECTYVASTITEVPFFSSKVKLGKNGVEQIYGLGSLNDYEAAGLKAMLPELRSSIEKGIAFAKGE

>Chlamydomonas_reinhardtii_pm

MSLQSSIRADSNCTLPNNPVCVLLPVDFIVAAMASSTSSAMAKWAAQAARGFAAAAPSSGKGRKVAVLGAAGGIGQPLSMLMKMNSQVSSLSLYDIAGTPGVAADVSHINTKAQVKGFDKDGLAEALRGCDLVIIPAGVPRKPGMTRDDLFKINAGIVRDLVTAVGQHCPGAVLNIISNPVNSTVPIAAEQLKKMGVYDKRKVMGVTTLDVVRAKTFYAEKNGLDVASVDVPVVGGHAGVTILPLFSQATPKATMSAEVLDALTKRTQDGGTEVVQAKAGKGSATLSMAYAAALFADSCLRGLNGAPVVECTYVESTVTDAPYFASKVKLSTEGVDKIHDLGPLSDYEKAGLKAMMPELLASIEKGVQFVKGA

>Sulfolobus_solfataricus_ATCC_35092_ar

MVKIAFIGVGKIGQTIAYSVIFDGLASEAILYDIVPELPEKFEHELRHAIATRGLSTEVIGTNSLDDVTNADIILIMAGKPRKPGMSRRDLFIDNAKIQIDLAKQLPPKNPGALYIMVANPVDMMASVFMRFSKQFTISTGDAVETMRLRSYIAKKLKVPANKVTGYVAGEHGEDAVVLWSTVKVNGKPFEEVAKGLTKEEVENYVKSIPGEIIRVMGGTTWGPATIIKDIVRSVVFNEGRVMSIATPRTYQGEIIHISVPIVVGAEIGPSLEGVLPESDRQRLNKSVEDFYRVYKENLDHLLQTVKQ

>Metallosphaera_sedula_ATCC_51363_ar

MAKVGFIGAGKIGQTIAYSALVSGAVDEAVIYDIIPELPDKFEHELRHAFATKGIKANVLGTNSLDDVSGMDIVVISAGKPRKPGMSRRDLFVDNAKIMIDLAQKLPSKNPGAIYLMVANPVDMMASVFMKYSKQFTISAGDQVETMRMRSFIAKKLKIPVTSVDGFVGGEHGEDAVVLWSTVKIKGKPVDEFNINKDEVSDYVKKIPGEIIRVIGGTTWGPGTIIADIIKSIAFSENRVMSIATPKEYEKEIIHVSAPTVVGSSIGPSLESLLDEKDRWHLNSAMKDFYEAYKENLKQLEQATKA

>Ignicoccus_hospitalis_KIN4/I_ar

MARVPYKVAVIGTGRVGATFAYTMAIVPGVARMVLVDAVPGLSKGVMEDIKHAAAVFRRSIQVEAYDDVSKVENADAIVITAGKPRKADMSRRDLAKVNAQIIRDIGDKLRDRNPGAFYMVITNPVDVMTMILSDVIGNKGTVIGTGTSLDTYRFRSAVSELLNEPIAAIDGYVVGEHGEEAFVAWSTVTVKGVPIDEYIKEKGLDLSRSRIEEYVKEVAATIIAKQGATIWGPAATFQEIVVSHLANEGRIIPVSVVQEVPGVGRVAVSVPTKISGRLVPLPQLLNEEERERLKRAAEAIKRVYEEAVSS

>Pyrolobus_fumarii_DSM_11204_ar

MARLKIAVVGVGRLGQAVAFRVLQEPYVKELILVDIREDFVQGIAEEFRHYVAIASMHDVEVEAVSDASEVAGADIVIVTAGVPRKPGMSRRDLAGQNAKIMKTIAEAMSPRNPNAVFLIATNPVDAMTMVFKRYARVEKVYGFGTMLDTARFRSILARELGVEPSRVTGFFVGEHGESGFVAWSTVYVDGIHVDKWVKRKGKLLDKQAVEQKVHRVAAEVIAATGATMWGPAGAFIKVIRGFAIPEDHLLAFGVEMKFPEVEEPVILSVPTMVTRGYVKPLLELLNEEERKKLVESAKSILNVYKQALTALEEQKES

>Methanoregula_boonei_DSM_21154_ar

MSKVTIIGATGNVGTFAAYAVSVDPHVHEILLYGREGREAFLKGLAQDFADSFAARGTNIRVTWTTSLKDVAGSDIVVITAGTPRGPGQNRLDLALGNARIIAPMARTIGTIAPDTKIIMVTNPVDVMTCVALKYSGLKPNQVFGLGTHLDSMRLKSLIASYFKVHVSEVHTRIIGEHGDSMVPLWSATTIGGIKISNLPAFAHLPVQDFIQSVKTSGEQIIKNKGSTVYGPGEAIATLVKTILGDENRILTVSAYVKSEVHGIGGVCIGVPARINKNGAFPVTIRIDESEVIAYRESVEKIRATIHQIIGELENDKDIGPAAPKRQKKTRV

>Bacillus_subtilis_168_b

MGNTRKKVSVIGAGFTGATTAFLIAQKELADVVLVDIPQLENPTKGKALDMLEASPVQGFDAKITGTSNYEDTAGSDIVVITAGIARKPGMSRDDLVSTNEKIMRSVTQEIVKYSPDSIIVVLTNPVDAMTYAVYKESGFPKERVIGQSGVLDTARFRTFVAEELNLSVKDVTGFVLGGHGDDMVPLVRYSYAGGIPLETLIPKERIDAIVERTRKGGGEIVNLLGNGSAYYAPAASLTEMVEAILKDQRRVLPTIAYLEGEYGYEGIYLGVPTIVGGNGLEQIIELELTDYERAQLNKSVESVKNVMKVLS

>Lactobacillus_brevis_KB290_LDH_b

MKNLTTKNHQKVVLVGDGAVGSAYAYSMMNQGIAEEFVIVDVVKERTEGDALDLEDAQVFTSPKNVYSGDYSDCKDADVVVITAGAPQKPGETRLDLVNKNLKILSSIVKPVVESGFDGIFVVAANPVDILTYATWKFSGFPKEKVIGSGTSLDTSRLRVALAKKFNVDPRNVEAYIMGEHGDSEFAAYDEATVGSKPLKVVAKEHGLTDDDLAKIEDDTRNKAYEIINRKGATYYGVATCLMRITKAILRDENAILPIGAALNGEYGLDDIFIGTPAIVNANGLGGVIEVPLSDKEKDLMAKSAETLKKVTQDGLAALEN

>Clostridium_botulinum_D_str._1873_LDH_b

MNPKKTKISIIGAGFVGSTTAYALMMSSLVSELVIVDINKDRATGEAMDLAHGASFVSPVNIYAGDYSDTKNSDIVIITAGAAQKPGETRLDLINKNFNIFKSIIPEITKYSPNSILLVVSNPVDILTYITYKLSGFPKERVIGSGTVLDTSRFRYLLSEHFDIDARNIHTYIMGEHGDSEIATWSITTVAGMDIQDYCNNFCHQCAGLEKYDIENSVKNAAYEVIEKKGATYYAIGLAVKRIVDAILRDENSILTVSSLLEGQYGLNDIYLGIPSIVGSTGVKKALEVSLNETESTKLINSANTLKQYIDKLSF

>Acetohalobium_arabaticum_ATCC_49924_LDH_b

MVSEKEGVKIGIIGAGFVGSTTAFTVMMNGLASEIVLVDIDQDKAEGEAMDLRHGASFVSPVNIETGGYQECQDADIVIITAGASQEPGETRLDLTKRNVEIFKDMIPKLTAEINSDTLLLVVTNPVDILSYVTWKLSDLPARQVIGSGTVLDSSRFRYILSQRCDIDARNIHGYIIGEHGDSEVPVWSATNIVGVPFEEFDEVCNQKKNNDDKSELISKIKNVAYEIIDRKGATYYAIALAINRIVKGIIRDENSILTLSTLLQGEYGFSDVYLSLPCIINRNGIREILELELSPAEQEALNTSAEVLQDLIGGKLNI

>Calothrix_sp._NIES-4101_b

MSYSPSFPSCNSSRVTVIGAGRVGSTLAQRIAEKNLADVVLLDIVPGMPQGLALDLIEARGIESHDRRIIGTNNYADTSASEIVVITAGFPRKPGMSRDDLLLTNAKIVVEAAKNAIKYSPQAIFIIVTNPLDVMTYLAWQATDLPRDRIMGMAGVLDSARFETFIALELGVAPKDVKAMVMGSHGDLMVPLPRYSTVNGVPITELLDAETIAKLVERTRNGGAEIVELMQTGGAFFAPASATCVMVESILHNQSRMLPAAALMKGEYGLNDVFIGVPCRLNCGGIEKIVQLKLTDIELEELHVSAANVRKNIDRAQEILMAGV

>Nostoc_punctiforme_ATCC_29133_b

MSSSPNSPIACNLPRVTIVGAGRVGSTLAQRVAEKNLADVVLLDIIAGMPQGLALDLMEARGIEIHNRQIIGTNNYADTSGSQIVVITAGLPRKPGMSRDDLLKTNAKIVVEAAKNAIAHSPNAIFIVVTNPLDVMTYLAWQATGLPRDRIMGMAGVLDSARFEAFIALELGVLPADVKAMVLGSHGDLMVPLSRYATVNGIPITELLDAATIERLIERTRNGGAEIVELMQTGGAFFAPASATSVMVESILLNQSRLLPVAAYLQGEYGLEDVVIGVPCRLGCGGIESVLELILSDEEREGLHTSAQSVRQNIERSQEILANKNNS

>Anabaena_variabilis_ATCC_29413_b

MSSSPDSPPILHRLPRVAIIGAGRVGSTLAQRIAEKNLADVVLLDIVEGMPQGLALDLLEARGIELHNRQIIGTNNYADTSGSQIVVITAGFPRKPGMSRDDLLRTNAKIVVEAAKQAIAYSPCAIFIVVTNPLDVMTYLAWEATGLPRNRIMGMAGVLDSARFETFIALELGVLPADVKAMVLGSHGDLMVPLSRHATVNGIPITELLDAATIERLVERTRNGGAEIVELMQTGGAFFAPASATSLMVESILLNQSRLLPVSVYLQGEYGLKDVVIGVPCRLGLNGIESVIELNLSDSEREALQTSAQSVQKNIERWHSTQHS

>Mycoplasma_mobile_ATCC_43663_LDH_b

MDKKIKRVAMVGAGLVGVSVLYSCMNRGLAEQYGIIDINDKLSVGHSLDFEDASAANNHNFSVGKIEYSDLKDYDVVVITAGRPQKPGETRLEMVADNAKIMSNIAKNIKKSGFKGVSIVVANPVDVMTFIYQHETGFDKNRVISSGTSLDSARLRFEISKKLKVHPKSVQAFVLGEHGDSSVSVYSAATVSGKSFNEIVKERGISKKELEDMHTTVYKKAYEIINRKGSTYFGIGSTVAELVEAILTDSHAIFGVGVYLTGQYGVKDLYIGVPTVLGSKGVVEVINFNLTKEEQEKFVSSATILKGNIQKALEAIKG

>Ktedonobacter_racemifer_DSM_44963_b

MRRKITVVGAGFVGSTLVQRLAERDYADVVMFDIVPNMPQGKALDIMQAGPVLGFDTQVTGTNDYADTVGSDIVVITSGFPRKPGMTRDDLVKKNQEIVSQVTEQVVKYSPDCIIICVTNPLDAMAQIALKVSGFPRERVLGMAGVLDTARFRTFIAQEVGASVRDVQAYVLGGHGDTMVPLARMCTVAGVPISQLISPERIEEIVQRTRDGGAEIVKLLGTGSAYYAPSASVLQMVDSIMLDKKMIMPCAVYLQGEYGIHDLFVGVPAQIGSKGLEKVIEIELNPSEREQLTKSADAVKELVHVMGI

>Thermotoga_sp._RQ2_LDH_b

MKIGIVGLGRVGSSTAFALLMKGFAREMVLIDVDKKRAEGDALDLIHGTPFTRRANIYAGDYADLKGSDVVIVAAGVPQKPGETRLQLLGRNARVMKEIARNVSKYAPDSIVIVVTNPVDVLTYFFLKESGMDPRKVFGSGTVLDTARLRTLIAQHCGFSPRSVHVYVIGEHGDSEVPVWSGAMIGGIPLQNMCQICQKCDSKILENFAEKTKRAAYEIIERKGATHYAIALAVADIVESIFFDEKRVLTLSVYLEDYLGVKDLCISVPVTLGKHGVERILKLNLNEEELEAFRKSASILKNAINEITAEENKHQNTGG

>Chloracidobacterium_thermophilum_b_b

MGRKKITVVGAGNVGATVAELLVLKELGNVVLVDIVEGLPQGKGLDLNEMAPVEGYDALVTGTNGYDETKDSDVVVITSGAPRKPGMSRDDLVEINQKIVASVTEQVAPRSPNAILVVVANPLDAMCTVAHRVSGFPRERVVGMAGVLDSARMRYFLADALQVSVENITAFVLGGHGDTMVPLPRYSTCAGIPIPELLPEDTIAAIVERTRHGGAEIVKLLGTSAWYAPASATVEMVEAIIKDKKKILPCAALLQGEYGIDGLFIGVPVKLGRGGVEQIIEIKLTEDEAAALKKSAAAVQELVNLLK

>Plasmodium_falciparum_7G8_am

TKIALIGSGQIGAIVGELCLLENLGDLILYDVVPGIPQGKALDLKHFSTILGVNRNILGTNQIEDIKDADIIVITAGVQRKEGMTREDLIGVNGKIMKSVAESVKLHCSKAFVICVSNPLDIMVNVFHKFSNLPHEKICGMAGILDTSRYCSLIADKLKVSAEDVNAVILGGHGDLMVPLQRYTSVNGVPLSEFVKKNMISQNEIQEIIQKTRNMGAEIIKLAKASAAFAPAAAITKMIKSYLYNENNLFTCAVYLNGHYNCSNLFVGSTAKINNKGAHPVEFPLTKEEQDLYTESIASVQSNTQKAFDLIK

>Methanosarcina_mazei_ATCC_bAA-159_ar

MVKISVIGAGNVGSTTVQRLAELEPGEIVMTDIVEGMPQGKALDLMQAGAINGYDTRITGTNDYADIANSDLVIITAGIARKPGMSREDLIKTNSKIIGDVAGNIAKYAPNSIVINVTNPLDIITYVAMKATGFDPEKVFGMSGVLDAGRFASFIAEELKCSKRDVEAMVIGGHGDLMVPLPQYTTVSGIPLPELLPEKTIDRLVERTVNGGAEIVELLKQGSAFYAPSAAIVRMAEAVIKDSRRVLPASAYLEGQYGQKGIYFGVPVKLGANGIEEILELKLEDSQCEILKKSSETIRKGISKLEI

>Methanosarcina_barkeri_DSM_804_ar

MAKISVIGAGNVGATTVQRLAELELGEIVMTDIVEGLPQGKALDLIQAGAIKGYDTSIIGTNDYAEIVDSDLVIITAGIARKPGMTREDLIKTNSKIIAEVSRNIAKYAPDSIVINVTNPLDIITYIAMKSTGFETKKVFGMSGVLDSGRFASFIAEELKCSKKDVQAMVIGGHGDLMVPLPQYTTVSGVPLTDLLPGDRIARLVERTVNGGAEIVELLKQGSAFYAPSAAIVSMAEAVIKNSKRILPASAYLEGHYGQEGIYFGVPVKLGASGVEEILELKLDESQYETLRKSSETIRNTISQLEI

>Methanolobus_tindarius_DSM_2278_ar

MNKVAVIGSGNVGATTVQRLAELNIADVVMVDIVEGLPQGKALDILQAAPLMGYDVDVTGTNDYADIAGSDIVVVTAGIARKPGMDRSDLLATNIKITQQVCENIQKYAPDSIIMTVTNPLDIITYAALRCTQFDRNRVFGMSGLLDSSRFASFIAKEMNCSVRDINAMVLGGHGDSMVPLPEYSTVSGIPLSRLMDGETINRIVERTVNAGAEIVGHLKNGSAFYAPSAAITIMVEAILNDTKKIVPASAFLNGEYGQHDICLGVPVKLGRNGVEEIIELELTDEETSALQKSAETVKEGIATIRI

>Methanohalophilus_mahii_ATCC_35705_ar

MAKISVIGAGNVGATTVQRLAEMEFGSIVLVDIVEGLPQGKALDLMQAGAIVGYDTKIKGSNDYADIVDSDIVIITAGAARKPGMSRADLININTKIMKDVCEKIKQYAPKSIVIAVTNPLDIMTYVALEITGFEPNRVFGMSGILDTGRFASFIADELAYSKRDVEAMVIGGHGDQMLPLPQHSSVFGTPLPELLGEDVIAGLVERTIHGGAEIVALLKQGSAFYAPSAAIVQMVESILRDEKRILPVSAYLQGEYGQKGIYFGVPAKIGCDGIEGIIELELTEEQEHALKESSASIRENIEDLDI

>Methanoculleus_bourgensis_ATCC_43281_ar

MARRHLSPETAVTRGAKSEEGHSGLLDPLYPDGETSSHEVGGEGSTSRRGRRPLAAGTGSGPEGSRLTVDVRRPWGCGVEEGCSGECLCARSTEDASPTAISMAKVTIIGATGNVGMFAAHTVSEIPYVSEMLLVGRPGREDFLGGCCHDLSDSFAARGTHVRLSYSTRLADAEDSDVIICTAGVPRKPGQDRNDLALENAKIVAETAETIGRCSPDAILFMVTNPVDVITAVALKYSGFQPRQVFGLGTHLDSMRLKSLIARYFRVHVSEVHTRIIGEHGESMVPLWSATTIGGIQIHNLPTFSGLPVQEMIDAVRTSGQAIIKDKGSTVYGPGEAIATLVRTILGDENRILTVSSYITSEVHGIGDVCIGVPARLNRNGVFPVPIRLQGDEVTGFQESVQKIRTITTEVMERLEEAR

>Methanoplanus_limicola_DSM_2279_ar

MGQFAAYAVSKIPFVSRLVLFGREGNEDFLEGIKLDFIDSFSALGRDVKLSASTNPGDIEGSDVVIITSGVPRSPGQDRLDLAKQNARVVAYYSKMIAGYAPDAIIMVVSNPVDVMTAVALECSGFIPSRVFGLGTHLDSMRLKSYIANHFSVHVSEVHTRIIGEHGETMVPLWSATTIGGIQVQNLPEFSSLPKDEFVECVKVAGSEIINKCGATIYGPGEAIATIVRTILGNENRILSVSAYIRSEVNDIGDVCIGVPVRINRKGVFPVPIRISEEEVSGFQRSVDRIKSITSEVLSDLKSENAEIDKKNNVVHENDLLF

>Ferroplasma_acidiphilum_ar

MDKKISIIGAGAVGASVAQVLAIKNIADINIFDIVEGVAEGKALDIQEGAPHFGFDCKLHGFCTQDPKEYANLKGSDVIVVTAGLARRPGMSRDDLLVKNIGIMKDVGEQIKKYSPDSIIVAVTNPADIMAYALQKASGISPERIIGLGGSLDSSRFRTFLAEALNVSVKDVNAFVIGGHGDDMVPFIHYSSVSGIPISDLLSEEKIQEIIKRTRFGGGEILNLYKTGTAFYAPSISISVMVESVINDQHRVIPCAAYLTGKHAENYKVSNAFIGVPIKIGKNGVEEIYDLKFSEADAKEWMKSVESVKKNNKLADDYFASH

>Methanotorris_igneus_DSM_5666_ar

MKVSVIGASGRIGSSVSFLLAKEPDIKDLIMISREKSLNKLKGLQMDIYDALAGLRSDANIEVHSDKDLSVVDGSDIVIITAGVPRKENMSRLDLAKINAEIVGRYAREIAKICDTKLFIITNPVDVMTYKAFIESNYDRNMVFGLGTHLDSLRFKVAIAKFFGVHIDDVRTRIIGEHGDTMVPLLSATGIGGIPIQMMPNFEKFPYKEVIDDVKNKGSEIIKLNGGSEFGPASAVINIVNCIIHDEKRLLTLSAYLDGEIDGIRDVCIGVPVKVGRNGIEEVVPIKLENDELEAFRHSVKVVKEYCELVKNI

>Methanococcus_aeolicus_ATCC_bAA-1280_ar

MEISIIGASGKIGTAISVLLAKEKYVRHINLISREKSLDKLKGLKLDIYDAIAAEELDVEISVHDEKDLSVVCNSKITIIPAGAPRTGDMTRLDLAKKNAGIVKRYAKDIGKTCDTKLFMITNPVDVMTHKALIESGYDKSQVFGLGTHLDSMRFKVAIAKYFNAHIGDVRTRIIGEHGDSMVPLISSTAIGGIPITRLPEYKDFPYNEVVDFVKNGGKRIIQLKGGSEYGPASAVLNVVRCIANDNKKYLTLSTYLDGELDGIKDVCIGVPVVIGKKGIERIVPIELDEKEFNDFKKSVEIVKRYWNEVKGI

>Methanobacterium_formicicum_DSM_3637_ar

MKVSVIGASGRVGKAAAFCLAEESAVSEVVLLSREKSLGQVQGEALDMNDAMAAKDIRVLITPTANFEDIADSKIVVITSGMPRTPEMTRMDVAIPNAKIIAEYSQLVAKHAPESIILVITNPVDVMTYVAYKASGFPRNRVIGLGNHLDSLRLKNLIAKHFNIHVSEIHTRIIGEHGDHMVLLLSSTSIGGILVKYFPQYQSFDVDAIVDKVKNAGSYVINKKGATEYGPAFAISNIVKTIINDEKRILTLTTYLDGEIDDVSDVCLGVPVKLGINGVERIISGKMSDNELEDFKTAARVVKSATDEVMASLEDKVNFSK

>Archaeoglobus_fulgidus_ATCC_49558_ar

MKLGFVGAGRVGSTSAFTCLLNLDVDEIALVDIAEDLAVGEAMDLAHAAAGIDKYPKIVGGADYSLLKGSEIIVVTAGLARKPGMTRLDLAHKNAGIIKDIAKKIVENAPESKILVVTNPMDVMTYIMWKESGKPRNEVFGMGNQLDSQRLKERLYNAGARNIRRAWIIGEHGDSMFVAKSLADFDGEVDWEAVENDVRFVAAEVIKRKGATIFGPAVAIYRMVKAVVEDTGEIIPTSMILQGEYGIENVAVGVPAKLGKNGAEVADIKLSDEEIEKLRNSAKILRERLEELGY

>Methanopyrus_kandleri_AV19_ar

MSKVAVIGATGRVGSTAAARLALLDCVNEVTLIARPKSVDKLRGLRRDILDSLAAAQKDAEITIGCERDDYVDADVIVMTAGIPRKPGQTRLDLTKDNAAIIKKYLEGVAEENPEAIVLVVTNPVDVLTYVALKVSGLPKNRVIGLGTHLDSMRFKVLIAKHFNVHMSEVHTRIIGEHGDTMVPVISSTSVGGIPVTRMPGWEDFDVEEAVREVKEAGQRIIETWGGSQFGPAQAITNLVRTILQDERRVLTVSAYLDGEIDGIRDVCIGVPARLGREGVLEIVPIELEEDEMRAFRRSVKVVKEATREAMEAISER

>Halorhabdus_utahensis_DSM_12940__LDH_ar

MAPNLYPRLSITRDQITMPDQAVKGKVAIIGAGDVGATTAYALMMSGSVSEIALVDIDHEKAEGEAMDLRHGAAFVKPVNIYAGDYEDAHDADVVIIAAGASQKPGETRLELLERNVDIFHDMVPRITDGLADDAVVLVVANPVDVLSYVTWKVSDLPWHRVIGTGTVLDTSRFRNVLSKNCNVDARNIHAYVIGEHGDSEVLVWSATHMAGVPFDDYCPVCDKDCDLNNRDLIADEVQGAAYEIIERKGATYYAIGLATTEIVESIIRDENSILTVSTLMDGQYGLEDVYLSLPAVVNRGGIRRVVDLDLDENEREQFIESGELLSNEIAKLDI

>Halalkalicoccus_jeotgali_DSM_18796_ar

MTKVSVVGAAGTVGAAAAYNIALREIADELVLVDIPEKEDDTVGQAADVNHGVAYDANTTVRQGGYEATEGSDVVVITAGIPRSPGQSRLDLAGDNAPIMADIGSSIEEHNDDYVTITTSNPVDLLNRHLYETGERAREKVIGFGGRLDSARFRYVLAERFDTQVQNVEATILGEHGDSQVPVFSKVRVDGRDPEFSDAEKEEILDELQESAMNVIERKGATEWGPATGVGHMVEAVIRDTGEVFPASVPLSGEFGHSGVGLGVPAKLGSEGVEEVVEWDLDEYEREQLGAAADKLAEQYDQID

>Halococcus_saccharolyticus_DSM_5350_ar

MVKVSVVGAAGTVGAAAGYNIALRGIADELVFVDIPDQEDTTIGQAADVNHGVAYDTNTTVRQGTYEDTAGSDVVVITAGIPRQPGQTRLDLGEDNAPIMEDIGSSLAEHNDEFVSVTTSNPVDLLNRHLYETGDRPREHVIGFGGRLDSARFRYVLGERFDTQVGNVEASIIGEHGDAQVPVFSKVRVDGRDPDFSDDERTDILESLQESAMNVIERKGATQWGPATGVGHVVESIIRDTGTVLPGSLVLNGEYGHDDVGLGVPLKLTSDGAEVVDWNLSEYEREQLGQAADKLADQYETIS

>Haloferax_gibbonsii_ATCC_33959__LDH_ar

MSTERGKGTVAIVGGAGAVGSSTAFALMESSLVGEIVLVDVAKERVEGEAMDLNHGSYFTSPVRVRTGDYEDCWDADVVIVTAGASQKPDETRLDLMERNADIFADMIPQITEGLNDDAVMLIVTNPVDVLSYVTWKVSDLPAERVIGSGTVLDTSRFRHSLSREFDLDPANVHAYVVGEHGDSEVLVWSSANLGGIPFESYATSHGVDDVDALKAGVETEVREAAYEIIDRKERTNYGVARSVAATTERILGGDNSILTVSTLVSGEHDIDGVYMSLPCTVNETGVRDVHEFDLSADETAALRESADVIRESIDRLDVA

>Natrialba_magadii_ATCC_43099_ar

MTKVSVVGAAGTVGAAAGYNIALRDIADELVFVDIPDKEDDTIGQAADANHGAAYDSNTTIRQGGYEATEGSDVVVITAGIPRQPGQTRIDLAGDNAPIMEDIGSSIAEYNDDFITVTTSNPVDLLNRHLYEAGNRARDKVIGFGGRLDSARFRYVISQRYDVPVQNVEATILGEHGDAQVPVFSKVRVNGQDLEFTDDEKDELLEELQTSAMNVIEKKGATEWGPATGVGHMVEAILRDTGEVLPASVTLEGEFGHEDTAFGVPVKLGSNGVEEVVEWDLTEYERNQLGEAAEKLSDQYDKIA

>Natrinema_versiforme_JCM_10478_ar

MTKVSVVGAAGTVGAAAGYNIALRDIADELVFVDIPDKEDDTIGQAADANHGAAYDSNTTIRQGGYEDTEGSDVVVITAGIPRQPGQTRIDLAGDNAPIMEDIGSSIAEHNDDFITVTTSNPVDLLNRHLYETGDRAREKVIGFGGRLDSARFRYVISKRFDAPVQNVDATILGEHGDAQVPVFSKVRVNGQDPEFDEDERDDLLSELQTSAMNVIEKKGATQWGPATGVGHMVEAILRDTGEVLPGSVKLEGEFGHEDTAFGVPVKLGSDGVEEIVEWDLTEFERNQLGEAAEKLSEQYDKIS

>Vulcanisaeta_distributa_DSM_14429_ar

MITIIGSGRVGATTAAFLMFFEPDNEIVLIDVIKNLPQGEALDLNHAAAILGKSVRYRGSNDYKDMEGSDLVIVTAGLARKPGMTREELAAKNAEIVASIAEQIRKYAPNSIVIITTNPLDAMVYVLYKKLGFPRNRVIGFSGVLDSQRMAYYASLLVGIAPESIIPVVLGQHGENMYPVPEASFVYGKPLTEFITKEQYDDIVKKTVQAGAEITNLRGFSSNWGPAAGLALMVDSIKKDRKRIFEASVYLDGEYGVRDVFAEVPVVLGKNGVEKIIELNLNEEQKKKFLASVEAIRKNLMQVPPQFLS

>Pyrobaculum_aerophilum_ATCC_51768_ar

MITIIGSGRVGTAAAVIMGLMKLDNKILLIDIVKGLPQGEALDMNHMSSILGLDVEYVGSNEYKDIEGSDLIIVTAGLPRKPGMTREQLLEANAKIVAEIGREIKKYAPDSIVILTTNPLDAMTYVMWKATGFPRERVIGFSGVLDAGRLAFYAAKKLGISPASILPIVLGQHGESMFPVPSKSYVHGVPLSKLLTEEQLKEVVEETVKAGARITELRGFSSNWGPGAGLAIMAEAVKRDAKRALIASVVLQGEYGVRDVPVEVPIILGRSGVVKVLEVELTEEERQKFMQSVEAVKKLVASVPPSYLQ

>Aeropyrum_pernix_ATCC_700893_ar

MITILGAGKVGMATAVMLMMRGYDDLLLIARTPGKPQGEALDLAHAAAELGVDIRISGSNSYEDMRGSDIVLVTAGIGRKPGMTREQLLEANANTMADLAEKIKAYAKDAIVVITTNPVDAMTYVMYKKTGFPRERVIGFSGILDSARMAYYISQKLGVSFKSVNAIVLGMHGQKMFPVPRLSSVGGVPLEHLMSKEEIEEVVSETVNAGAKITELRGYSSNYGPAAGLVLTVEAIKRDSKRIYPYSLYLQGEYGYNDIVAEVPAVIGKSGIERIIELPLTEDEKRKFDEAVQAVKKLVETLPPQLRE

>Sulfolobus_acidocaldarius_ATCC_33909_ar

MVKVAFIGVGRVGQTIAYNTIVNGYADEVMLYDVVPELPEKFEHEIRHALAALRVKTELLSTNNIDDISGADIVVITAGKPRKPGMSRRDLFIDNAKIMIDLAKKLPKKNKGAMYIMVANPVDMMASVFMKYSGENTISTGNQVETMRMRSYIAKKLNIPAYEVGGYVGGEHGEAAMVLWSTVTVKGKPFSESLGVNKAEVEDYVKKIAAEIIRVLGGTTWGPGADIEEVIRSVALNEGKVMSVAFPHKYEDEIIHISEPVVVGRTVGPALTSALDENDKARLSQAIKEVYNVYKSNLKELEQVIS

>Desulfofustis_glycolicus_DSM_9705_b

MKAPVRVAVTGAAGQIAYSLIFRVAHGDMLGPDQPVILQLLEIPPAMGALNGVVMELKDCAYPLVAGIVASDDPNVAFKDVDYAFLVGARPRGPGMERSDLLEANAKIFSVQGKALNDHASKDVKVLIVGNPANTNALITLKNAPNLNPKNITSMMRLDHNRAMSQIAEKAGSHTTKVEKVVVWGNHSATQYPDISYATVDGKAVKSVVDNDWYVDTFIPTVQQRGAAIIKARGASSAASAACSAVDHMRDWALGSNGKWVSMGVFAAGNPYGIDENIMYSFPITCENGQWKIVEGLEISEFSRDMMAKTEAELVGEREAIASLL

>Hyphomonas_sp._TMED31_b

MKKPVKVAVTGAAGQIGYALLFRIAAGDMLGTDQPVDLHLLEITPALGALNGVVMELNDCAFPLLNSIVATDDPNVAFKDCDYALLVGAMPRKQGMERKDLLSANGGIFGPQGKAINDHASRDVKVLVVGNPANTNALIAQQNAPDLDPKCFTAMVRLDHNRAMSQLAEKTGTHNTDIKNVIIWGNHSATQYPDIHHATVKGDAAKPQVDDAWYQDTFIPDVQQRGAAIIKARGASSAASAASAAIDHMRSWALGTPEGEWVSMGIPSDGSYGIEPGVIYGYPCTCKDGKYEIVQGLDINEFSRAKMDATDAELREERAAVEHLFA

>Rhodanobacter_fulvus_Jip2_b

MKAPVRVAITGAAGQIGYALLFRIAAGDMLGKDQPVILHLLEITPALPALQGVVMELNDCAFPTLAGIVATDDLNVAFKDVDYALLVGARPRGPGMERKDLLEANGAIFGPQGKALNDHAKRDVKVLVVGNPANTNALIAQQNAPDLDPKCFTAMVRLDHNRALSLLAEKTGKHNTDVKHMTIWGNHSSTQFPDLSHVTVDGKPALELVDQSWYESDFIPTVQQRGAAIIKARGASSAASAASAAIDHVRTWALGTAEGDWVSMGIPSDGSYGIEPGVIYGYPVTVKNGKYEIVQGLAVSDFARGRMDATAKELREERAGVEHLFAKK

>Halomonas_titanicae_bH1_b

MMKDPVRIAITGGAGQISYSLIFRIAAGDMLGPDQPVILQLLEIPQAMDALNGVVMEVNDCAFPLVQDIVATDDPNVAFKDADFALLVGARPRGPGMERKDLLEANAAIFSVQGKALNDHASRDVKVLVVGNPANTNALIASCNAPDLDAGQFTAMTRLDHNRALTQLAQKTGKHVTDVENMIIWGNHSATQYPDLAQCKVDGKPAFDLVERDWYENDFIPTVQQRGAAIIKARGASSAASAASSAIDHMHDWALGSKGIVSMAIPSDGSYGIDEGIIYSYPVRCQGGKYEIVQGFEIDEFSQEKMKATEKELREERAAVEHLLG

>Saccharophagus_degradans_2-40_b

MKAPVRVAVTGAAGQISYSLLFRIAAGEMLGKDQPVILQMLEITPALEALKGVAMELDDCAFPLLAGMVCSDDPNVAFKDADYALLVGARPRGPGMERKDLLEANAAIFSVQGKAINDHASRDIKVLVVGNPANTNALIAQRNAPDINPRQFTAMMRLDHNRGLSQLAAKLDVTLEDITKMTIWGNHSATQYPDLFHTLVKGDVAVEQVEKDWYENDFIPTVQQRGAAIIKARGASSAASAANAAIFHMRDWALGTPENDWVSMGVYSDGSYGIEEGLIYSFPCVCKNGDWEIVQGLTIDEFSRAKMTATENELKEERDAVKSLLPA

>Alcanivorax_hongdengensis_A-11-3_b

MKAPVRVAVTGAAGQISYSLLFRIASGDMLGKDQPVILQLLEITPALEALKGVIMELEDCAFPLVAGISGTDDANVAFKDADYALLVGARPRGPGMERKDLLEANAAIFSAQGKAINDNASKDIKVLVVGNPANTNALIAQRNAPDINPRQFTAMTRLDHNRGMAQLATKLGKTVNDIKKMTIWGNHSSTQYPDLHHCEVDGKIAVDQVEQDWYEGTYIPEVQQRGAAIIKARGASSAASAANAAVDHMRSWALGTAEGDWVSMGIYSDGSYGIQEGLIYSFPCTCKDGDWSIVQGLEVNDFSRAKMQATEQELAEERDAVSHLLP

>Beggiatoa_alba_b18LD_b

MKKPVRVAVTGGAGQISYSLVFSIASGQLLGPDQPIILQLLEMPVAMEALKGVAMELDDCAFPLLHDIVTTDDVKTAFTDIEYALLVGAKPRGPGMERNDLLRDNGKIFTTQGKALNDYANRNVKVLVVGNPANTNAYIAMRSAPDLNPKNFTAMTRLDHNRALSQLSAKTGTKVTDIKKMVIWGNHSSTQYPDITNTTVAGKAAKSLVDQNWIVNDFIPTVQQRGAAIIKARGKSSAASAANAAVDHIRDWALGSNGEWVSMAVPSDGSYGIPEGLIYSFPITTANGEYSIVQGLEIDEFSRDRMIKTQNELEEERSAIADLLG

>Moraxella_cuniculi_DSM_21768_b

MKQPVRVAVTGAAGQIGYSLLFRIASGEMLGKDQPVILQLLEIPVEKAQQALKGVIMELDDCAFPLLVDVIGTDDPNVAFKDADYALLVGARPRGPGMERADLLQENAKIFTVQGKALNDVASRDVKVLVVGNPANTNAYIAMKSAPDLPAKNFTAMLRLDHNRALTQVAKKTGKAVKDIKQLTVWGNHSPTMYADYRFATINGESVKDMINDQDWNANVFLPTVGKRGAAIIEARGLSSAASAANAAIDHMRDWALGTNGEWVTMGIPSDGSYGIPEGVMFGFPVTTENGEYKIVQGLEIDEFSQERINITLNELEEERAAIDHLFA

>Burkholderia_lata_ATCC_17760_b

MAKPAKRVAVTGAAGQIAYSLLFRIANGDLLGKDQPVILQLLDLPQAQGAVKGVVMELDDCAFPLLSGVVITDDPKVAFKDADVALLVGARPRSKGMERKDLLSANAEIFTVQGAALNEVASRDVKVLVVGNPANTNAYIAMKSAPDLPKKNFTAMLRLDHNRALSQLAAKSGKPVASIEKLAVWGNHSPTMYPDFRFATAEGESLLKLINDDVWNRDTFIPTVGKRGAAIIEARGLSSAASAANAAIDHVRDWVLGTNGKWVTMGIPSDGSYGIPEDIIYGVPVVCENGEYKRIEGLEIDAFSREKMDGTLAELLEERDGVAHLLKN

>Burkholderia_sp._YI23_b

MAKSAKRVAVTGAAGQIGYSLLFRIANGDLLGKDQPVILQLLDLPQAQGAVKGVVMELEDCAFPLLAGVVVTDDPKVAFKDADVALLVGARPRSKGMERKDLLSANAEIFTVQGKALNDVASRDVKVLVVGNPANTNAYIAMKSAPDLPKKNFTAMLRLDHNRALSQLAAKSGKPVASIEKLAVWGNHSPTMYPDFRFATAEGQDLTKLINDDEWNRNTFIPTVGKRGAAIIEARGLSSAASAANAAIDHVRDWVLGTNGKWVTMGIPSDGSYGIPEDIIYGVPVTCENGEYKRVEGLEIDAFSREKMDGTLNELLEERDGVSHLLGK

>Bordetella_petrii_ATCC_bAA-461_b

MSKPAMRVAVTGAAGQIGYALLFRIASGEMLGKDQPVILQLLEIPDEKAQKALKGVIMELDDCAFPLLQEVTAHSDPRTAFKDADVALLVGARPRGPGMERKDLLSVNAQIFTAQGKALNDVASRNVKVLVVGNPANTNAYIAMKSAPDLPAKNFTAMLRLDHNRALSQLAAKSGKAVADIEKLVVWGNHSPTMYPDYRFATVGGQSLAKLINDDAWNRDTFIPTVGKRGAAIIEARGLSSAASAANAAIDHVRDWVLGSNGKWVTMGIPSDGSYGIPEGIIYGVPVTTENGEYKRVEGLEIDAFSRERLDFTLKELLEERDGVKDLLK

>Bordetella_parapertussis_12822_b

MSKPALRVAVTGAAGQIGYALLFRIASGEMLGKDQPVILQLLEIPDEKAQKALKGVIMELEDCAFPLLHEVTAHSDPRTAFKDADVALLVGARPRGPGMERKDLLSVNAQIFTAQGRALNDVASRNVKVLVVGNPANTNAYIAMKSAPDLPAKNFTAMLRLDHNRALSQLSAKSGKRVADIEKLIVWGNHSPTMYPDFRFATVGGQGLTQLINDDAWNRDTFIPTVGKRGAAIIEARGLSSAASAANAAIDHVRDWVLGSNGKWVTMGIPSDGSYGIPEGIIYGFPVVTENGEYKMIKDLEIDAFSRERLDFTLKELLEERDGVKDLLK

>Achromobacter_xylosoxidans_A8_b

MSKPALRVAVTGAAGQIGYALLFRIASGEMLGKDQPVILQLLEIPDEKAQKALKGVIMELDDCAFPLLQEVTAHSDPRTAFKDADVALLVGARPRGPGMERKDLLSVNAQIFTAQGKALNDVASRNVKVLVVGNPANTNAYIAMKSAPDLPAKNFTAMLRLDHNRALSQLAAKSGKAVADIEKLVVWGNHSPTMYPDYRFATVGGESLSKLINDDAWNRDTFIPTVGKRGAAIIEARGLSSAASAANAAIDHVRDWVLGSNGKWVTMGIPSDGSYGIPEGIIYGVPVTTENGEYKRIEGLEIDAFSRERLDFTLKELLEERDGVKDLLK

>Kingella_denitrificans_ATCC_33394_b

MKKPVRVAVTGAAGQIGYALLFRIASGEMLGKDQPVILQLLDLPQAQKAVQGVMMELQDCAFPLLDSMIATDNPEVAFKDADIAILVGARPRGPGMERADLLQANAQIFTVQGAALNKVASRDVKVLVVGNPANTNAYIAMKSAPDLPAKNFTAMLRLDHNRAASQLAEKTGKAVRDIEKLCVWGNHSPTMYADYRFATIGGESVKDLINDQDWNANVFLPTVGKRGAAIIEARGLSSAASAANAAIDHIRDWVLGTNGKWVTMGVPSDGSYGIPAETIFGFPVTCENGEYKIVQGLEIDAFSQERIDKTLAELQAEKDGVKDLL

>Nitrosospira_multiformis_ATCC_25196_b

MKTPIRVAVTGAAGQIAYSLLFRIAAGDMLGEDQPVILQLLDIPQSLPSLKGVVMELDDCAFPLLRDITITDDPKTAFRDINIAMLVGARPRTKGMERKDLLEANGTIFRAQGKALDEVAGRDVKVLVVGNPANTNAYITMKNAPSLKPTSFSSMMRLDHNRAVFQLAVKVGQPVSSVRKMIVWGNHSSAQYPDLSHAEVDGHNAADLVNDMAWIETGFIPVIQKRGMEVIEARGSSSAASAANAAICHMRDWVSGTPEGDWVSMGIPSDGSYGIPEGVIYGYPVTCQGGEYKIVPDLEISEFSRMKMQASYRELMGERESIKHLLG

>Mycobacterium_tuberculosis_H37Rv_b

MSASPLKVAVTGAAGQIGYSLLFRLASGSLLGPDRPIELRLLEIEPALQALEGVVMELDDCAFPLLSGVEIGSDPQKIFDGVSLALLVGARPRGAGMERSDLLEANGAIFTAQGKALNAVAADDVRVGVTGNPANTNALIAMTNAPDIPRERFSALTRLDHNRAISQLAAKTGAAVTDIKKMTIWGNHSATQYPDLFHAEVAGKNAAEVVNDQAWIEDEFIPTVAKRGAAIIDARGASSAASAASATIDAARDWLLGTPADDWVSMAVVSDGSYGVPEGLISSFPVTTKGGNWTIVSGLEIDEFSRGRIDKSTAELADERSAVTELGLI

>Mycobacterium_marinum_M_b

MSASPLKVAVTGAAGQIGYSLLFRLASGSLLGPDRPIELRLLEIEPALKALEGVVMELDDCAFPLLSGVEIGSDANKIFDGANLALLVGARPRGPGMERSDLLEANGAIFTAQGKALNEVAADDIRVGVTGNPANTNALIAMTNAPDIPRERFSALTRLDHNRAISQLAAKTGVAVTDIKKMTIWGNHSATQYPDLFHAEVKGKNAAEVVNDQAWIEEYFIPTVAKRGAAIIDARGASSAASAASATVDAARSWLLGTPADDWVSMAVLSDGSYGVPEGLISSFPVTTKDGNWSIVKGLEIDEFSRGRIDKTTAELADERKAVTELGLI

>Nocardioides_sp._ATCC_bAA-499_b

MSSTPLKVAVTGAAGQIGYSLLFRLASGSLLGDRPIELRLLEITPALKALEGVVMELDDCAFPNLAGVQIGDDAEQIFDGVNLALLVGARPRGPGMERGDLLSANGAIFTAQGKALNKVAADDVRIGVTGNPANTNALIAMTNAPDIPQARFSALTRLDHNRAISQLAAKTGAAVTDIKKMTIWGNHSATQYPDVFHAEIGGRNAAEVVGDQDWIESTFIPTVAKRGAAIIEARGSSSAASAASATIDAARDWLFGSADADWVSMAVVSDGSYGVPEGLISSFPVTTKDGDWEIVQGLEIDDFSRAKIDASTAELADEREAVKELGLI

>Aeromicrobium_marinum_DSM_15272_b

MSSSPVKVAVTGAAGQIGYSLLFRIASGAVYGPDTPVQLQLLEITPALKALEGVVMELDDCAFPTLAGVEIGDDAEKIFDGVNLALLVGARPRGPGMERGDLLEANGAIFTAQGKALNKVAADDVRIGVTGNPANTNALIAQRNAPDIPADRFSALTRLDHNRAISQLAARTGSSVTEIAHMTIWGNHSATQYPDLFHATVGGRNAAEVVDDQAWIENDFVPTVAKRGAAIIEARGSSSAASAASATIDAARDWLHGSAADDWVSMAVQSDGSYGIPEGLIYSFPVTTSGGDWSIVQGLDIDEFSRGRMDATAAELIEERDAVQALGLI

>Tessaracoccus_sp._NSG39_b

MSTEAPVKIAVTGAAGQICYSLLFRIASGALLGDRPIELRLLEITPALKALEGVVMELDDCAFGNVVNIEIGDDPKKVFDGVNLAMLVGAMPRKAGMERGDLLSANGAIFTAQGKALNEVAADDIKVLVTGNPANTNALIASKNAPDIPAERFNALTRLDHNRALTQLAQKLGVSVNDISHMTIWGNHSATQYPDLFNALVGGKNAAELVDDQAWIEDTFIPTVAKRGAAIIEARGLSSAASAANATVEHMRDWVLGTPEGDWISMAVPSDGSYGVPEGLISSFPVTVKDGEYSIVQGLELNDFSRAKIDASVAELVDERNAVTELGLI

>Propionibacterium_acidifaciens_F0233_b

MDEEKLLSTTPVKVAVTGAAGQICYSLLFRIASGSLLGDTPVELRLLEITPALPRLEGVVMELDDCAFPNLAGVEIGDDPEKVFDGVDLAMLVGAMPRKEGMDRSDLLSANGKIFTAQGAALNKVAASDVKVLVTGNPANTNALIAMDNAPDIPNERFSALTRLDHNRAKTQLAKKLGVNVGEVTNMTIWGNHSNTQYPDLFNAKVGGTCAYDLVNDQAWYENEYIPTVAKRGGAVIKARGASSAASAANATVEAMHDWVLGTPEDDWVSMSVVSDGSYGVPEGLISSFPVTVRNGTYEIVQGLEINDFGKKKIAETVAELQTEQGAVRELGLI

>Propionibacterium_acnes_J139_b

MAGRHLALMGEVILDTNKSPGQLHIVTASRLQNSQVSSRTFKERSMTQTPVKIAVTGAAGQICYSLLFRIASGSLLGDTPIELRLLEITPALKALEGVVMELDDCAFGNLVNIEIGDDPKKVFDGVNAAFLVGAMPRKAGMERSDLLTKNGAIFTAQGKALNDVAADDVRVLVTGNPANTNALIAATNAVDIPNDHFAALTRLDHNRAKTQLARKTGKTVNDVRHMTIWGNHSSTQYPDVFHAEVAGQKATNLVDEAWIENEFIPTVAKRGAAIIDARGASSAASAANATVECMRDWMGSTPEGDWVSMAIPSDGSYGVPEGLISSFPVTITNGKVEIVQGLDIDDFSRAKIDASAKELADERDAVKELGLI

>Mobilicoccus_Pelagius_NBRC_104925_b

MSNTPVKVAVTGAAGQIGYSLLFRIASGALLGPDTPVQLQLLEIEPALKSLEGVVMELDDCAFPTLAGVEIGSDPNVIFDGANIALLVGARPRTKGMERGDLLSANGAIFTGQGKALNDHAADDIRIGVTGNPANTNALIAMSNAPDIPSERFSALTRLDHNRAISQLAAKLGVPVTEIKKMTIWGNHSATQYPDLFHAEVSGKNAAEAVGDQAWIENDFIPTVAKRGAAIIEARGASSAASAASATIDAARDWIKGSAEGDWISMAVASDGSYGVPEGIISSFPVTTKNGDWEIVQGLEIDDFSRGKIDASVKELEEERDAVKELGLI

>Terrabacter_sp._Soil810_b

MSTTPVKVAVTGAAGQIGYSLLFRIASGALLGPDTPVELRLLEITPALKALEGVVMELDDCAFPTLAGVQIGDDATKVFDGVNHALLVGARPRGPGMERGDLLEANGGIFAPQGKALNEVAADDIRITVTGNPANTNALIAMSNAPDIPTERFSALTRLDHNRAISQLAAKLGVPVTEITKMTIWGNHSATQYPDLFHAEVGGRNAAQAVGDQDWLAGTFIPTVAKRGAAIIEARGASSAASAASATIDHARTWVQGTPDGDWVSMAVRSDGSYGVQEGLISSFPVTVEDGRWSIVQGLDIDDFSRGKIDASVAELAEERDAVKGLGLI

>Janibacter_sp._HTCC2649_b

MSQTPVKVAVTGAAGQIGYSLLFRIASGALLGPDVPVELRLLEITPALKALEGVVMELDDCAFPTLAGVQIGDDAETVFDGVNHALLVGARPRGPGMERGDLLSANGGIFAPQGKALNKVAADDVHITVTGNPANTNALIAMSNAPDIPTSQFSALTRLDHNRAISQLAAKLGVPVTEIKKMTIWGNHSATQYPDLFHAEVSGKNAAQLVRNDTDGDSWLADTFIPTVAKRGAAIIEARGASSAASAASATIDHARTWVEGTAEGDWVSMAVRSDGSYGVQEGLISSFPVTVKDGQWSIVQGLTIDEFSRGRIDASVAELAEERDAVKGLGLI

>Paenarthrobacter_aurescens_TC1_b

MSSPIKIAVTGAAGQIGYSLLFRIASGALFGGDTPVQLRLLEITPALKALEGVVMELDDCAFPALDSVEIGDDADHIFDGVTLALLVGARPRSKGMERGDLLSANGAIFTAQGKALNRVAADDVRIGVTGNPANTNALIAMSNAPDIPASRFSALTRLDHNRAIGQLAAKTHARVGDIRKMTVWGNHSATQYPDIFHAEVAGRNAAEVVNDQDWIENEFIPTVAGRGAAIIEARGASSAASAASATIDAARDWLLGTPEGDWVSMAVASDGSYGVPEGLMYSYPVTTSGGNWEIVEGLEVNDFSRRKMDATAAELFDERAAVANLGLI

>Saccharothrix_sp._NRRL_b-16348_b

MTRTPVNVTVTGAAGQIGYALLFRIASGHLLGPDVPVRLRLLEIPQAVKAAEGTAMELDDCAFPLLSGIDITDDAKTAFDGVNVALLVGARPRTKGMERGDLLEANGGIFKPQGEAINAGAADDVRVLVVGNPANTNALIAQQHAPDVPAERFTAMTRLDHNRALSQLAKKLGVSVTDIKKLTIWGNHSATQYPDLFHAEVNGQVAAEAVNDQAWLENEFIPTVAKRGAAIIEARGASSAASAANAAIDHVHDWVNGTAEGDWVSMAIPSDGSYGVPEGLISSFPVTVTDGKYSIVQGLEIDDFSRARIDASVAELVEERDAVKALGLI

>Streptomyces_sp._HPH0547_b

MTRTPVNVTVTGAAGQIGYALLFRIASGQLLGADVPVRLRLLEITPALKAAEGTAMELDDCAFPLLQGIDITDDPNVAFDGANVALLVGARPRTKGMERGDLLEANGGIFKPQGKAINDHAADDIKVLVVGNPANTNALIAQAAAPDVPAERFTAMTRLDHNRALTQLAKKTGSSVADIKKLTIWGNHSATQYPDIFHAEIAGKNAAQVVNDEKWLADEFIPTVAKRGAAIIEARGASSAASAANAAIDHVHTWVNGTAEGDWTSMGVPSDGSYGVPEGIISSFPVTTKDGAYEIVQGLEINEFSRTRIDASVKELTDERDAVRGLGLI

>Streptomyces_clavuligerus_ATCC_27064_b

MTRTPVNVTVTGAAGQIGYALLFRIASGHLLGPDVPVKLRLLEITPALGAAQGTAMELDDCAFPLLRGIDITDDPNVAFDGANVALLVGARPRTKGMERGDLLEANGGIFKPQGKAINDHAADDIKVLVVGNPANTNALIAQAAAPDVPAERFTAMTRLDHNRALSQLAAKTGTTVADIKRLTIWGNHSATQYPDIFHAEIAGKNAAEVVNDQAWLADTFIPTVAKRGAAIIEARGASSAASAANAAIDHVHTWVNGTAEGDWTSMGIPSDGSYGVPEGLISSFPVTVKDGRYEIVQGLEINEFSRTRIDASVQELAEERDAVRALGLI

>Thermomonospora_curvata_ATCC_19995_b

MTSTPVNVTVTGAAGQIGYALLFRIASGQLLGPDVPVRLRLLEIPQAIKAAEGTAMELDDCAFPLLAGIDITDDLKTAFDGANIALLVGARPRTKGMERRDLLEANGGIFKPQGEAINAHAADDIKVLVVGNPANTNALIARSHAPDVPADRFTAMTRLDHNRAIAQLSKKAGVPVSSIKKMTIWGNHSATQYPDLFHAEIDGRNAAEVVGDQEWLENDFIPTVAKRGAAIIEARGASSAASAASAAIDHIHTWVNGTPEGDWTSMAVVSDGSYGVPEGLVSSFPVTCSGGKWEIVQGLEIDEFSRGKIDASVAELVEERDAVRNLGLI

>Kribbella_flavida_b

MSTTPVKVAVTGAAGQIGYSLLFRIASGALLGPDTPVELRLLEITPALKALEGVVMELDDCAFPTLAKVEIGDDPNTIFDGANVALLVGARPRTKGMERGDLLEANGAIFTGQGKALNDHAADDIRVTVTGNPANTNALIAKSNAPDIPAERFSALTRLDHNRALAQLAKKTGTSVNDLKKLTIWGNHSATQYPDIFHAEVAGKNAAEVVNDQTWLENDFIPTVQKRGAAIIEARGASSAASAAAATIDHTRDWLRGSADGDWLSMAVVSDGSYGVPEGLISSFPVITKDGNWEIVQGLEINDFSRGRIDASAAELAEERDAVQQLGLIG

>Tessaracoccus_flavus_b

MSTAAPVKIAVTGAAGQICYSLLFRIASGSLLGDTPIELRLLEITPALKALEGVVMELDDCAFGNLVNIEIGDDPNKVFDGVNLAMLVGAMPRKAGMERGDLLSANGAIFTAQGKALNEVAADDVKVLVTGNPANTNALIAQQNAPDIPAERFNALTRLDHNRALSQLAAKLGVSVTEISHMTIWGNHSATQYPDLFNAKVNGQSAAEAVNDQEWIENTFIPTVAKRGAAIIEARGLSSAASAANATVEHMRDWVSGTKEGDWVSMAVPSDGSYGVPEGLISSFPCTVKDGKYEIVQGLDLNEFSRSKIDASVAELVDERNAVTELGLI

>Propionibacterium_sp._oral_taxon_192_str._F0372_b

MSETPVKIAVTGAAGQICYSLLFRIASGALLGERPIELRLLEITPALKALEGVVMELDDCAFPNLTSIVIGDDPKKVFEGVNAAMLVGAMPRKAGMERGDLLSANGAIFTAQGKALNEVAADDVRVLVTGNPANTNALIAMSNAPDIPNERFNALTRLDHNRAKAMLATKVGASVGDVKHMTIWGNHSSTQYPDLFHAEVAGKPAGELVDQAWVEDEFIPKVAKRGAAIIEARGASSAASAANATVEHMRDWILGTPQGDWVSMAVPSDGSYGVAEGVISSFPCVVKNGKYEIVQGLELDAFSRAKLDASVAELLDEKKAVTELGLI

>Cutibacterium_avidum_b

MTQTPVKIAVTGAAGQICYSLLFRIASGSLLGDTPIELRLLEITPALKALEGVVMELDDCAFGNLVNIEIGDDPKKVFDGVNAAFLVGAMPRKAGMERSDLLTKNGAIFTAQGKALNEVAADDVRVLVTGNPANTNALIAATNAADIPNDHFAALTRLDHNRAKTQLARKTGKTVNDVRHMTIWGNHSSTQYPDVFHAEVAGQKATELVDEAWIENEFIPTVAKRGAAIIDARGASSAASAANATVECMRDWMGSTPEGDWVSMAIPSDGSYGVPEGLISSFPVTVKDGKVEIVQGLEIDDFSRAKIDASVKELSDERDAVKELGLI

>Propionibacterium_acnes_HL097PA1_b

MGEVILDTNKSPGQLRVVTASRLQNSQVSSRTFKERSMTQTPVKIAVTGAAGQICYSLLFRIASGSLLGDTPIELRLLEITPALKALEGVVMELDDCAFGNLVNIEIGDDPKKVFDGVNAAFLVGAMPRKAGMERSDLLTKNGAIFTAQGKALNDVAADDVRVLVTGNPANTNALIAATNAVDIPNNHFAALTRLDHNRAKTQLARKTGKTVNDVRHMTIWGNHSSTQYPDVFHAEVAGQKATNLVNEAWIENEFIPTVAKRGAAIIDARGASSAASAANATVECMRDWMGSTPEGDWVSMAIPSDGSYGVPEGLISSFPVTITNGKVEIVQGLDIDDFSRAKIDASAKELADERDAVKELGLI

>Janibacter_indicus_b

MSTTPVKVAVTGAAGQIGYSLLFRIASGSLFGPDTPVELRLLEITPALKALEGVVMELDDCAFPTLAGVEIGDDAEKVFDGVNHALLVGARPRGPGMERGDLLEANGGIFAPQGAALNKVAADDVRVTITGNPANTNALIAMNNAPDIPNERFSALTRLDHNRAISQLSAKLGVPVTEIKKMTIWGNHSATQYPDLFHAEVSGKNAAEAVGDQEWLESTFIPTVAKRGAAIIEARGASSAASAASATVDHARDWALGTPEGDWVSMSVCSDGSYGVPEGLISSFPVTVKDGKWEIVQGLEIDDFSRGKIDASVAELAEERDAVTKLGLIKG

>Phycicoccus_dokdonensis_b

MSTTPVKVAVTGAAGQIGYSLLFRIASGSLFGPDTPVELRLLEITPALKALEGVVMELDDCAFPTLAKVEIGDDAEKVFDGVNHALLVGARPRGPGMERGDLLEANGGIFAPQGKALNKVAADDIRVTVTGNPANTNALIAMSNAPDIPQERFSALTRLDHNRAISQLAAKTGAAVTEITHMTIWGNHSATQYPDLFHAEVGGRNAAELVNDEAWLADTYIPTVAKRGAAIIDARGASSAASAASATVDHARDWALGTPDGDWVSMSVRSDGSYGVPEGLISSFPVTTSGGDWQIVQGLEINDFSRARIDASVKELEEEREAVRGLGLLG

>Knoellia_aerolata_DSM_18566_b

MSATPVKVAVTGAAGQIGYSLLFRIASGALLGPDTPVELRLLEITPALKSLEGVVMELDDCAFPTLAKVEIGDDATKVFDGVNLALLVGARPRGPGMERGDLLSANGGIFAPQGKALNEVAADDVRITVTGNPANTNALIAMSNAPDIPTERFSALTRLDHNRAISQLAAKLGVPVTEIKKMTIWGNHSATQYPDLFHAEVGGRNAAEVVGDQEWLAGTFIPTVAKRGAAIIEARGASSAASAASATIDHARTWVQGTPDDDWVSMAVRSDGSYGVEEGLISSFPVTVKDGRWSIVQGLEIDEFSRGKIDASVAELAEERDAVRELGLI

>Tetrasphaera_japonica_T1-X7_b

MSTSPVKVAVTGAAGQIGYSLLFRIASGSLFGADTPVELRLLEITPALKALEGVVMELDDCAFPTLAGVEIGDDPTKIFDGVNFALLVGARPRGPGMERGDLLEANGGIFAPQGKALNEVAADDIRVTVTGNPANTNALIAMSNAPDIPSERFSALTRLDHNRAISQLAAKTGAAVTDITHMTIWGNHSATQYPDLFHALVKGQNAAELINDEDWLANTYIPTVAKRGAAIIDARGASSAASAASATIDHARTWAQGTPEGDWVSMSVRSDGSYGVPEGLISSFPVTVENGDWSIVQGLDINEFSRTRIDASVKELGDERDAVKGLGLI

>Serinicoccus_chungangensis_b

MNTPVKVAVTGAAGQIGYSLLFRIASGELLGPDTPVELRLLEITPALGALEGVVMELDDCAFPLLAGVETGDDADVVFDGVNVALLVGARPRTKGMERGDLLEANGAIFTAQGKALNSHAADDVRITVTGNPANTNALIAMSNAPDIPTERFSALTRLDHNRAIAQLAAKVGAPVTDVSHMTIWGNHSATQYPDLFHAQVGGRNAAEAVGDQQWIEDTFIPTVAKRGAAIIEARGSSSAASAASATIDHARDWLRGSPEGDWVSMAVRSDGSYGVEEGLISSFPVTTRDGSYEIVQGLDIDDFSRGRIDATVAELSEEKAAVTQLGLV

>Arthrobacter_sp._Rue61a_b

MSSPIKIAVTGAAGQIGYSLLFRIASGALFGGDSPVQLRLLEITPALKALEGVVMELDDCAFPALDSVEIGDDADHIFDGVNLALLVGARPRSKGMERGDLLSANGDIFTAQGKALNRVAADDVRIGVTGNPANTNALIAMSNAPDIPASRFSALTRLDHNRAIGQLAAKTHARVGDIRKMTVWGNHSATQYPDIFHAEVAGRNAAEVVNDQDWIENEFIPTVAGRGAAIIEARGASSAASAASATIDAARDWLLGTPEGDWVSMAVASDGSYGVPEGLMYSYPVTTSGGNWEIVEGLEVNDFSRRKMDATAAELFDERAAVAELGLI

>Saccharothrix_sp._CB00851_b

MTRTPVNVTVTGAAGQIGYALLFRIASGHLLGPDVPVRLRLLEIPQAVKAAEGTAMELDDCAFPLLSGIDITDDAKTAFDGVNVALLVGARPRTKGMERGDLLEANGGIFKPQGEAINAGAASDVRVLVVGNPANTNALIAQRHAPDVPAERFTAMTRLDHNRALSQLAKKLGVSVTDIKKLTIWGNHSATQYPDLFHAEVKGQNAAQAVNDQAWLENEFIPTVAKRGAAIIEARGASSAASAANAAIDHVHDWVNGTAEGDWVSMAIPSDGSYGVPEGLISSFPVTVTDGEYSIVQGLEIDEFSRARIDASVAELVEERDAVKALGLI

>Streptomyces_clavuligerus_b

MTRTPVNVTVTGAAGQIGYALLFRIASGHLLGPDVPVKLRLLEITPALGAAQGTAMELDDCAFPLLRGIDITDDPNVAFDGANVALLVGARPRTKGMERGDLLEANGGIFKPQGKAINDHAADDIKVLVVGNPANTNALIAQAAAPDVPAERFTAMTRLDHNRALSQLAAKTGTTVADIKRLTIWGNHSATQYPDIFHAEIAGKNAAEVVNDQAWLADTFIPTVAKRGAAIIEARGASSAASAANAAIDHVHTWVNGTAEGDWTSMGIPSDGSYGVPEGLISSFPVTVKDGRYEIVQGLEINEFSRTRIDASVQELAEERDAVRALGLI

>Arabidopsis_thaliana_pc

hMATATSASLFSTVSSSYSKASSIPHSRLQSVKFNSVPSFTGLKSTSLISGSDSSSLAKTLRGSVTKAQTSDKKPYGFKINASYKVAVLGAAGGIGQPLSLLIKMSPLVSTLHLYDIANVKGVAADLSHCNTPSQVRDFTGPSELADCLKDVNVVVIPAGVPRKPGMTRDDLFNINANIVKTLVEAVAENCPNAFIHIISNPVNSTVPIAAEVLKKKGVYDPKKLFGVTTLDVVRANTFVSQKKNLKLIDVDVPVIGGHAGITILPLLSKTKPSVNFTDEEIQELTVRIQNAGTEVVDAKAGAGSATLSMAYAAARFVESSLRALDGDGDVYECSFVESTLTDLPFFASRVKIGKNGLEAVIESDLQGLTEYEQKALEALKVELKASIDKGVAFANKPAAAAAN

>Brassica_napus_pc

hMAATSAASSISIGSTVPRASSSLPQSRAQAVNFNYSLPRFTALRSSTLLSGPDSSSFAKSLRGSVTKSQSTDTKPYGLNINASYKVAVLGAAGGIGQPLSLLIKMSPLVSTLHLYDIANVKGVAADLSHCNTPSQVRDFTGPAELADCLKDVNVVVIPAGVPRKPGMTRDDLFNINAGIVKTLVEAVADNCPNAFIHIISNPVNSTVPIAAEVLRKKGVYDPKKLFGVTTLDVVRANTFVSQKKNLKLIDVDVPVIGGHAGITILPLLSKTKPSVSFTDEEIEKLTVRIQNAGTEVVDAKAGAGSATLSMAYAAARFVESSLRALDGDGDVYECSFVDSTLTDLPFFASRIKIGRNGVEAVIESDLQGLTEYEHKALEALKPELKASIEKGVAFANKPAN

>Medicago_sativa_pc

hMAAAASATFTIGTTQTGRSLPQSSPFGLKFNSQVNFNTFSGLKAMPSLRCDSESSFSGKETCAALRATFAPKAQKENRNLTRNLQPQASYKVAVLGAAGGIGQPLALLIKMSPLVSDLHLYDIANVKGVAADISHCNTPSKVLDFTGASELANCLKGVDVVVIPAGVPRKPGMTRDDLFNINAGIVRDLVTAVADNCPNAFIHIISNPVNSTVPIAAEILKQKGVYDPKKLFGVSTLDVVRANTFVAQKKNLRLIDVDVPVVGGHAGITILPLLSKTKPSVSFTDEEIEELTVRIQNAGTEVVEAKAGAGSATLSMAYAAARFVESSLRALDGDADVYECSFVQSDLTDLPFFASRVKIGRKGVEALIPTDLQGLSEYEQKALEALKPELKASIEKGVAFAQKQTVTA

>Oryza_sativa_subsp._japonica_pc

hMASAVTISSVGAQAGMISKPRNHGFTSYSGLKAASSVSFESGSSFLGRNASLRASVAPRIVPKAKSGSQISPEASYKVAVLGAAGGIGQPLGLLIKMSPLVSELHLYDIANVKGVAADLSHCNTPSQVLDFTGPSELANCLKGVDVVVIPAGVPRKPGMTRDDLFNINASIVKSLVEAVADNCPEAFIHIISNPVNSTVPIAAEVLKQKGVYNPKKLFGVTTLDVVRANTFVAQKKNLKLIDVDVPVVGGHAGITILPLLSKTMPSVTFTDEETEQLTKRIQNAGTEVVEAKAGAGSATLSMAYAAARFVESSLRALAGDPDVYECTFVQSELTELPFFASRVKLGKNGVESIISADLEGVTEYEAKALEALKSELKASIEKGIEFVHKQQTAAASV

>Chlamydomonas_reinhardtii_pc

hMLLANRVNAGAARRVSGRAAAPIAVRAGRRSLVCEARKVALLGAAGGIGQPLALLLKMNKFVTELALYDIANVVGVAADLSHCNTPVKVTGYTGPEELGACLKGADLIVIPAGVPRKPGMTRDDLFNTNAGIVKALVEAVAKHAPNAVLEIITNPVNSTVPIAVETLKLAGVYDPKKVIGVTSLDIVRANTFVSEAKGLDMKDVDVPVIGGHAGSTILPLLSQTTPPVTFTEAEKKAMTDKIANAGTVVVEAKAGKGSATLSMAYAAARMAESTLLGLNGEPNIYECAFVQSDVVADCPFFASKVLLGPNGVAKVMGLGELDAFEQAAMAAMLPQLKSEIQKGLDFVKSPPAPAS

>Chlorella_variabilis_pc

hMEQRPIVASRQQPVRAGRRQAVIEARKVAVLGAAGGIGQPLSLLLKMNRMVTELALYDIANVAGVAADLSHCNTNTKVTGYTGAEELAGALKGAELVVIPAGVPRKPGMTRDDLFNINAGIVKTLCEGVAASCPDAIIAIISNPVNSTVPICAEVLKKAGVYNPRKVMGVTTLDVVRANTFVAEAKGLDTKDVDVPVIGGHAGETILPLLSQATPRVQFSPEEAAKMTERIQNAGTEVVEAKAGAGSATLSMAYAAARFAESVLLGLSGEQDIIECTYVESEVVPGFQYFASKVRLGPDGVEEFLPLGPLTAFEQEGLEKMKGLLSKNIEAGIAFANK

>Zea_mays_pc

hMATATSASLFSTVSSSYSKASSIPHSRLQSVKFNSVPSFTGLKSTSLISGSDSSSLTKTLRGSVTNAQTSDKKPYGFKINASYKVAVLGAAGGIGQPLSLLIKMSPLVSTLHLYDIANVKGVAADLSHCNTPSQVRDFTGPSELADCLKDVNVVVIPAGVPRKPGMTRDDLFNINANIVKTLVEAVAENCPNAFIHIISNPVNSTVPIAAEVLKKKGVYDPKKLFGVTTLDVVRANTFVSQKKNLKLIDVDVPVIGGHAGITILPLLSKTKPSVNFTDEEIQELTVRIQNAGTEVVDAKAGAGSATLSMAYAAARFVESSLRALDGDGDVYECSFVESTLTDLPFFASRVKIGKNGLEAVIESDLQGLTEYEQKALEALKVELKASIDKGVAFANKPAAAAAN

>Prunus_persica_pc

hMAATSAAASFSIGTTCSLGHKAVSFPQSKPCSLRFNSQNPLRSSFNGLKATTSLACESETSFLGKETTAALRASFALKAHKGAQVVQTPFQPQASYKVAVLGAAGGIGQPLALLIKMSPLVSDLHLYDIANVKGVAADLSHCNTPSQVLDFTGASELAKSLKGVDVVVIPAGVPRKPGMTRDDLFNINAGIVKNLVEAVADNCPEAFIHIISNPVNSTVPIAAEVLKKKGVYNPKKLFGVTTLDVVRANTFVAQKKNLKLIDVDVPVVGGHAGITILPLLSKTKPSVSLTDEEVEKLTVRIQNAGTEVVEAKAGAGSATLSMAYAAARFVESSLRALDGDGDVYECSYVESDLTELPFFASRVKLGRKGVEALIPSDLQGLTEYEQKALEALKPELKASIEKGIAFANKQAVTA

>Lachnospiraceae_bacterium_CAG:25_b

MDYNKLALEMHEQNKGKIAVRSKVTVKTRDDLSTAYTPGVAEPCRKIRDNKEEVYRYTAKGNLVAVVSDGTAVLGLGDIGPEAAMPVMEGKALLFKEFADIDAFPICLDTKDTEEIIKTVKNIAPCFGGINLEDISAPRCFEIEKRLKEELDIPVFHDDQHGTAIVVAAGLLNALKFVGKKMEDANIVINGAGSAGISICKLLLQFGAGNVVLVDQKGALCPGEDWMNPAQKDMAEITNKEKQTGTLTEIIKDKDVFIGVSAPNIVTAEMVSTMADDAIIFAMANPTPEIMPDEAKKGGARVIATGRSDFPNQINNVLVFPGIFRGALDARAGQITEEMKMAAARAIASIISDDELNEEYIIPGAFDERVCKAVAKAVAEESQK

>Coprococcus_sp._CAG:782_b

MDYNSLSLKMHEENKGKVEVISKVKIKDRDDLSTAYTPGVAEPCRKIRDNKADVYKYTCKGNMVAVVSDGTAVLGLGDIGPEAAIPVMEGKSILFKEFGNVDAFPICLDTKDVDEIVETVKRIAPVFGGINLEDISAPRCFEIERRLKEELDIPVFHDDQHGTAIVVSAGLINALKLVGKPFDQANVVINGAGSAGISICRLLLQLGIGNVVLVDKNGALCPGQDWMNPAQTEMAEITNKDRQTGAIAEIMKGKDVFIGVSAPNIVTADMVASMAKDPIVFAMANPTPEIMPEEAKKGGVRVMATGRSDYPNQINNVLVFPGIFRGALDAKATAITEEMKIAAAKAIASIVSDDELNEEYIIPGAFDERVAKVVAKAVCDEAHRLGITK

>Coprococcus_comes_ATCC_27758_b

MKMSAIEQALEMHEKWNGKIETTAKSHVNSREDLAIAYTPGVAEPCKVIAKDPDAAYKYTMKANTIAVVSDGSAVLGLGNIGAKAAMPVMEGKAVLFKEFGGVNAVPICLDTQDTEEIIKTVVNIAPAFGGINLEDISAPRCFEIESRLKELLDIPVFHDDQHGTAIVVLAGIINALKVTGKKKEDCKIVVNGAGSAGVAITKLLLTYGFPKITMCDINGIISADSPNLNWMQKEMTKVTNLDHETGTLADALKGADIFVGVSAPNIVSQEMVASMNKDAILFAMANPVPEIMPDLAKAAGAKVVGTGRSDFPNQVNNVVAFPGIFKGALEGRAPQITEEMKLATAKAIAGLVPDEELNEDNILPEAFDPRVSQVVSEAVKALI

>Dorea_sp._CAG:105_b

MNYNEMALKMHEEHKGKISVTSKVAVKTRDDLSTAYTPGVAEPCRKIRDDKSEVYRYTAKGNLVAVVSDGTAVLGLGDIGPEAAMPVMEGKSILFKEFAGIDAFPICLDTKDTDEIVETVKRLAPTFGGINLEDISAPRCFEIERRLKEELDIPVFHDDQHGTAIVVSAGLTNALKYVGKEFSEAKVVINGAGSAGISICKLLLELGVGDIVLVDRNGILAVGEEWMNPAQKEMAEKTNKEQIHGDLKTAMQGRDVFVGVSAPNIVTAEMVSTMAKDAVVFAMANPTPEIMPDEAKKGGAKVVATGRSDFPNQINNVLVFPGIFRGALDARATDITEEMKIAAVKAIADIIKPEELTEDYIIPGAFDERVADNVAREVAKTAIETGVSKLKKAE

>clostridioforme_90A7_b

MDYAEAALKMHRENHGKLEMASKIPLTTRDELSTAYTPGVAAPCLKIKEDKSEAYTYTAKGNLVAVVTDGTAVLGLGDIGPEAAMPVMEGKAVLFKKFGGVDAVPICLDTRDTEEIIETVKRIAPTFGGINLEDISAPRCFEIEQRLERELDIPVFHDDQHGTAIVATAALINALKLTGKQMDGIKVIVNGPGSAGTAITEMLLGAGVKDLIVCDEHGALCPGREKMDGHKEKLSLMTNAGKEKGRLEDVIAGADVFIGVSAAGVVSKDMVRTMKKDAIVFAMANPVPEIMYEEAKEAGARVMATGRSDCPNQINNVLVFPGLFRGALDCRARDITCGMKLAAAYGIAGLVSGEELGEEYIIPSAFDERVAKAVAEAVKRAAVKRDAAKMDAVKPAALKAAGEVKAAGEVKQGRE

>Lachnoclostridium_phytofermentans_b

MTLNEKALKLHEEWNGKLETTSKCQVKSREDLALAYTPGVAEPCKVIANDSEAAYRYTIKANTVAVVSDGSAVLGLGNIGAHAAMPVMEGKCVLFKEFGGVNAFPICLDTQDTEEIIKTIIHIAPAFGGINLEDISAPRCFEIEARLKEQLSIPVFHDDQHGTAIVVLAGIINALKVTNKKKEDCRVVVNGAGSAGIAITKLLLNYGFCHLTLCDKVGILSNQTENLNWMQKEMMEVTNLEGATGTLADALKGTDIFIGVSAPNIVAPEMVSSMNKDSILFAMANPVPEIMPDLAKEAGAKVVGTGRSDFPNQVNNVIAFPGIFKGALEGRATQITEEMKLGAAKAIADLVSNEELNEDYIIPEAFDERVCEVVSNAVKAYIK

>Anaerosporobacter_mobilis_DSM_15930_b

MTTNEKALILHEEWNGKIETVSKCTVKSREDLAIAYTPGVAEPCKVIAQDKEKAYKYTIKSNMVAVISDGSAVLGLGNIGPYAAMPVMEGKAVLFKEFGNVNAFPICLDTQDTEEIIKTIVNIAPAFGGINLEDISAPRCFEIEERLKELLDIPVFHDDQHGTAIVVLAGIINALKVTGKQKETCKVVVNGAGSAGIAISKLLITYGFKNIIMCDKVGILCKGADGLNWMQEQMMDVTNLEQKQGSLADALKGADIFVGVSAPNIVSKEMVASMNKDSILFAMANPVPEIMPDIAKEAGAKVVGTGRSDFPNQVNNVVAFPGIFKGALEGRATQITEEMKLAAAEAIASLVDEKDLNEDNIMPEAFDPRVAEVVSNAVKSHIVR

>Clostridium_gasigenes_b

MDYNKLSLEMHENNKGKISITSKVSVKTREDLSTAYTPGVAEPCRKIHANKEDVYKYTAKGNLVAVVTDGTAVLGLGDIGPEAAMPVMEGKAILFKEFANIDAFPICLDTTDVDEIVKTVKYLAPTFGGINLEDISAPRCFEIERRLKEELDIPVFHDDQHGTAIVVSAGLINALKLVGKKMQDVNIVINGAGSAGISICKLLLQFNVGNVVLVDRNGAVESSAEWLNEAQKDMAKITNKHNEKGNLIDVMKGKDIFIGVSGPNCVTSEMVATMNKDAIVFAMANPSPEIMPEEAKKGGARVIATGRSDFPNQINNVLVFPGIFRGALDVRATDITEEMKLAAAKAIASLIEESELNEDYIIPGAFDSRVAQVVAKEVARVAVESGISKLNK

>Clostridium_botulinum_C/D_str._DC5_b

MNYSEESLKIHEKNKGKISVTSKVSVTNKEELSTAYTPGVAEPCREIAKHGDNVYKYTSKGNLVAVVTDGTAVLGLGDIGPKAAMPVMEGKAILFKEFADIDAFPICLDTKDTEEIIRTVKYLAPTFGGINLEDISAPRCFEIERRLKEELDIPVFHDDQHGTAIVVLAGLLNALKVVGKELQNAKIVINGAGSAGISICKLLLLAGAKNVVMCDLKGALIKGAEWMNEAQKDIAEVTNKHLETGTIKDIIKGKDVFIGVSAANLLNADMVSTMNKDAIIFAMANPVPEIMPDEAKKGGARVIATGRSDFPNQINNVLVFPGIFKGALKARARDITEDMKLAAARAIASIVTDEELNEDYIIPDAFNKKVVSVVSNEVEKVARNTGITQE

>Clostridium_botulinum_b_str._Osaka05_b

MDIKEKSLMVHKKFKGKLSIEGKIQVKNKEDLSIAYTPGVAEPCVKISEDKSLVYEYTMKGNTVAVVTNGTAVLGLGDIGPYAGLPVMEGKALLFKEFANIDSFPICIDSKDPEEIIKTVKLIAPGFGGINLEDIKAPECFYIEKKLKEELDIPVFHDDQHGTAIVVLAGIYNALRFVGKKLEEARIVINGAGSAGISICKLLLQAGAKNIIMCDKEGSLVKGNNNLNEAQKLIAEVTNKENEKGILKDVIKGKDVFIGVSAPNIFTEEMVASMNNDSIVFAMANPTPEIMPDKAKKAGARVVATGRSDFPNQINNVLVFPGIFRGALDVRSKVINEEMKLAASKAIASLVQDNELNEEYIIPGAFDKRVAQVVAEEVKKVALKMGLSKL

>Clostridium_novyi_b_str._NCTC_9691_b

MNYSEKSLKIHEENKGKISVNSKIPVTNKEELSTAYTPGVAEPCREINKCPDNVYKYTAKGNLVAVVTDGTAVLGLGDIGPKAAMPVMEGKAILFKEFADIDAFPICLDTKDTEEIIKTIKYISPTFGGINLEDISAPRCFEIEKRLKEELDIPVFHDDQHGTAIVVLAGLLNALKVVGKELQSAKIVINGAGSAGISICKLLLLAGAKNIVMCDLKGALIKDAHWMNEAQKDIAEVTNKQLETGTIKDIIRGKDVFIGVSGPNVLNAHMVSTMNKDAIIFAMANPSPEIMPDEAKKGGARVIATGRSDFPNQINNVLVFPGIFKGALKARARDITEDMKLAAARAIASIVKDDELNENYIIPDAFNKKVVSVVSSEVEKVAENNRI

>Clostridium_magnum_DSM_2767_b

MNYFEESLKVHEKNAGKIEVISKVALETRDDLSLAYTPGVAEPCNKIHEDEENVYKYTTKGNLVAVVSDGTAVLGLGDIGAKAAIPVMEGKSILFKGFADVDAFPICLDTKDVDEIVKTVKLMAPVFGGINLEDIAAPRCFEIEDRLKEELDIPVFHDDQHGTAVVTLAGLINALKLTGKKFEDIKVVVNGAGAAGTSIIKLLLLSGVKDIVACDRKGIISRNQEDLNSAKKALAEITNPNNITGTLADAIVGADLFIGVSAPGALKPEMVKSMNKDSIIFAMANPVPEIMPEEAKAAGARVVGTGRSDFPNQVNNVLAFPGIFRGALDVRARQINEEMKLAAAHAIANYIKEEDINEDYVIPSALDKKVALKVAEAVAKAALETGVARIDVLKNY

>Caminicella_sporogenes_DSM_14501_b

MIDYNKLALELHKEKKGKISVVSKVKVENKEQLSTAYTPGVAEPCRVISKDKNAVYDYTSKSNLVAVVSDGTAVLGLGDIGATASIPVMEGKAVLFKSFADIDAFPICLDTRDVDEIVRTVKLLEPVFGGINLEDIASPRCFEIEEKLKKILDIPVFHDDQHGTAIVVAAGLINSLKLTNKKIEEIEIVVNGAGAAGIAITKLLLKLGVGNIVLCDKKGVINEDYEEANWAKREMAKITNKNKERGTLKDVIKGKDVFIGVSAPNIVTREMIESMADKAIVFAMANPIPEIMPDEAKLGGAFIVGTGRSDFPNQVNNVLAFPGIFRGALDVRASDINEEMKIAAAYAIANTISDDELTVDNILPKPFDKRVVKNVAEAVKNAAKKTGVSRI

>Ruminococcus_obeum_CAG:39_b

MDYAKESLRLHGEWKGKIEVVTRVPAENKDDLSLAYTPGVAQPCLEIQKDVNKSYDLTRRWNMCLVVTDGSAILGLGNIGPEAGMPVMEGKCALFKAFGDVDAFPLCIKSNDVDEIVNTIYLISGSFGGVNLEDISAPRCFEIEKKLKEKCDIPIFHDDQHGTAIVVSAGLTNALRLVGKKFSEAKVVINGAGSAGISICKLLLELGIGDVVMVDRKGILAPGEEWMNPAQKEIAEKTNKEQLHGDLKAAMKGRDIFVGVSAPNIVTAEMVSTMADDAIVFAMANPTPEIMPDEARKGGARVIATGRSDFPNQINNVLVFPGVFRGAFDVRASDINEEMKIAAAKAIAELISDEELSEDNIIPKAFDKRVGPAVAKAVAEAARKTGVARK

>Fusobacterium_varium_b

MPTVFERALEMHEKNKGKISIVSKVKVANKDDLSLAYSPGVAEPCRKIAANKEDVYKYTAKGNMVAVITDGTAVLGLGDIGPEAALPVMEGKCVLFKEFAGVDAIPICLDTKDPEEIIRTVKLLAPGLGGVNLEDISAPRCIEIETRLKKELDIPVFHDDQHGTAIVVAAGLINAFKVVGKKFSDAKVVVNGAGAAGSSITKLIRDLGAKEILVLDKPGILRRSEKESYDFSKKELAEITNPNDLAGDLAFAVQGADVFVGVSVGNILTTEMVKTMNKDAVIFAMANPTPEIMPEDALAGGAKIVGTGRSDYPNQINNVLVFPGLFKGALRAKSKKITEEMKLAAARGLASLITDEEMNENYIIPDAFDRRVADAVADAVEKVAREQGICRD

>Fusobacterium_necrophorum_subsp._funduliforme_b35_b

MSNVYEESLKLHEANRGKLSVTSKVSVKNREDLSLAYSPGVAEPCRKIQEKKEEVYRYTSRGNMVAVVTDGTAVLGLGDIGPEAALPVMEGKAVLFKEFGGVDAFPICLDTKDTEEIIATVKRIAPGFGGINLEDISAPRCVEIETRLKEELDIPVFHDDQHGTAIVVVAGLINALKLVHKKVEEIKVVINGIGAAGSSIAKLILQLGVPGKNMLLVGIDGILNRNTSEDYNQLHKELAFRTNDACQTGNLKDALQEADVFIGVSVGGIVSPEMIKTMNRDAIVFAMANPTPEIMPEEAKKAGARIVGSGRSDYPNQINNVLVFPGLFKGALRAKSKKITEEMKMAAAVGLAKLISEEELKEDYIIPGAFDPRVAETVAKEVEKVAKEQGICRE

>Paenibacillus_alvei_DSM_29_b

MPVALDIKEESLQLHKKLHGKIEVTSKIEVNSPEDLSLVYTPGVAESCRLIANDKNAANEYTLRGNMIAVVTDGSAVLGLGDIGPYAAMPVMEGKCMLFKQFGNVDAFPICLSTQNVDEIVNIVKNMEPTFSGINLEDISAPRCFEIERRLKEETNIPIFHDDQHGTAIVLLAALMNALKVVHKQMSNIRAVINGAGSAGIAIAKLLLKAGVQHVSLVDLKGVVCEGEEWMNPAQADMAMVTNREHIRGTLTEAVRGADVFIGVSAPKVLTQEHVRSMNEKPIIFAMANPTPEIFPDEALAAGAAVVCTGRSDFPNQVNNLLAFPGIFRGALDVRATDITDDMKLAAARGIAAIVTDAELSPTYIIPNPLDKRVVPSVASAIAQVAIETGAARVDKMPIYRV

>Bacillus_mycoides_b

MLESQINERSLLLHKELVGKIEITSKVEVNSADDLSLTYTPGVAESCKAIAADEETAYDYTARGNMVAVVSDGTAVLGLGDIGPKAAMPVMEGKSILFKKFANVDAFPLCLGTTDVDEIVTIVKNLEPTFAGINLEDIAAPRCFEIEKRLKEETNIPVFHDDQHGTAIVVLAAIINALKVVNKQMDAVKIVINGAGSAGIAIAKLLLQAGAQHITLVSLEGIVCEDESWMNPAQIEIAKKTNREYVRGTLKEAIHEADIFIGVSAPNVLTTELVQTMSEKPIVFAMANPIPEIYPEDALKAGAAVVGTGRSDYPNQVNNVLAFPGIFRGALDVRATDVTEEMKLAAAYGIANIITDEERNENYVIPNPLDKRVVPSVAAAVAKAAIESGVAQITKMPSYSK

>Kosmotoga_olearia_TBF_19.5.1_b

MDLKEKALKQHELWQGKLEVISKAKVETSEDLSIAYTPGVAEPCRKIAEDPEKVYKYTMKGNTVAVVTDGTAVLGLGDIGPEAALPVMEGKAVLFKEFAGIDAFPICLDTKDVDEIVKAVKYIAPGFGGINLEDISAPRCFEIEERLKAELNIPVFHDDQHGTAIVVLAGIINSLKIVQKKPNEMKVVINGAGAAGIAITKLLIKFGFKHIILCDKPGAIYRGADWTNPAQTKIAEITNPDNVKGTLKEVIKGADLFIGVSAPNIVTEEMIASMNKDAIVFAMANPVPEIMPDKAKAGGARIVGTGRSDFPNQVNNVLAFPGIFKGALKCRKQITDKMKIAAAYAIAGMIPESELNEENILPKPFQPGIADAVAEAVIKAAEEE

>Anaplasma_phagocytophilum_HZ_b

MRSHRSVKVSLVGAGNIGGTLAYMLGVAGICQELVFVDVMDGVPRGKLLDIGHALAISGVDITAVGGSDYAAIEGSDAIVVTAGLPRKEGMSREDLLMANAAVIKGVAENIRKYSPDAFVIVVTNPLDAMVWYMHQCSGLPVNKVVGMAGVLDSARFSFFLAKHMSVSVSSVSSVVLGGHGDLMLPLLKYSTVGGVSVSDLISCGRLSSEDVHAIIERTRKGGEEIVKLLKSGSAYYAPAASCMNMLESYLFDKRCVIPCSVGLDGKYGVNGGLFVGVPAVIGKNGVEEVIEYVLSQEEREIFEKSVGLISNSVKIISEQK

>Ehrlichia_minasensis_b

MIKRKKVALIGAGNIGGMIAYLIRLKNLGDVVLLDVNDGIAKGKALDMAESSPVGKYNGEILGTNNYADIEGADAIVVTAGIARKPGMSREDLVNTNVNIIREVAENICKYAPNAFVVVVTNPLDVMVFAMHKYSKLSSNMVVGMAGILDSARFSYFIAKELNVSVDNVSSLVLGGHGDLMLPLVKYSSVGGISIADLIKIGLITQDKVDAIIERTRKGGEEIVGLLKMGSAYYAPAESALLMIDSYLNDRRLILPCSVYLKGEYGVNNLFVGVPVIIGKNGVEKVIELQLTEQEKNVFDNSVKLIENLVSNI

>Candidatus_Neoehrlichia_lotoris_str._RAC413_b

MISRRKISLIGSGNIGGILAYLINQKKLGDIVMLDINEGLSRGKSLDISESSAVNGFCFDILGTSDYQEIRDSDAIIVTAGIARKPGMSRDDLLNINARIIEDIAKQIQKYSPNAFVIIITNPLDAMVWHLYKKSNLPSNMVVGMAGVLDSARFSYFIAQHLNISVENVSSLVLGSHGDLMLPLIRYSTVGGIPITDLIKLNIITQSDVDDIIQRTRKGGEEIVKLLQNGSAYYAPATSAIYMLESYLYDKKRILSCAAYLNGEYGVYDLFVGVPVIIGKNGIEKVIELDLLQSEKETFNNSIKLIKNLLQLLN

>Ehrlichia_chaffeensis_b

MIKRKKIALIGAGSIGGMIAYLVRSRNLGDVVLLDVNGGIAKGKALDIAESSPVAKHNGEILGTNNYADIEGADAIIVTAGISRKPGMSRDDLINTNVHVIKEVAENIAKYAPNAFVVVVTNPLDIMVLAMHKYSHLPSNMVVGMAGVLDAARFSYFIAKELNVSVDSVSSIVLGGHGDFMLPLVKYSSVGGISIADLVKMNLITQDRVNEIIEKTRKGGEEIVNLLKVGSAYYAPAESALLMVDSYLNDRRLMLSCSVYLKGEYGVHDLFVGVPVIIGKNGVEKVIELQLTEEEKNVFNDSVMSIRKLVSNI

>Wolbachia_sp._subsp._Drosophila_simulans_wRi_b

MTVQRKKISLIGAGNIGGTLTHMIALRELGDVVLLDISDGIPQGKALDIAESSPIDGFNVNITGTNRYEDIKNSDAIIITAGIARKPGMSRDDLLQTNAKVMKEVGENIKKYSPNAFVIVVTNPLDAMVSVVHKFSNLPTNMIVGMAGVLDSSRFRYFLASELNISVEDISAFVLGGHGDTMVPLINCASVAGVPLTQIIDMGLITQKKVDEIVERTRNGGKEIVDLLKSGSAYYAPASSAICMLESYLKDKRRILPCAAYLNGEYGVEELFIGVPVIIGKNGIEKILEVKMNDSEQEMFNKSVNSVRELVKSLGS

>Rickettsia_australis_Cutlack_b

MKKNPKISLIGSGNIGGTLAHLISLRELGDIVLFDVAEGVPQGKALDLMQAGTIAGSDIKIKGTNDYKDIEGSDAIIITAGLPRKPGMSRDDLISINTGIMKNVAENVKKYAPDAFVIVITNPLDVMVYVMLKASGLPHNKVIGMAGVLDSSRFNLFLAEEFKVSVSNVNSTVLGGHGDAMVPLARYSTISGIPIPDLIKMGLSSNENIEKIIDRTRNGGGDIVALLKTGSAYYAPAASAIEMLEAYLKDKRQILTCAAYLQGEYGVNDLYVGVPIIIGKEGVIKVVELQLTKEEKALFDKSVEGVKKLIETIK

>Rickettsia_conorii_b

MKQNAKISLIGSGNIGGTLAHLISLRELGDIVLFDVTEGVPQGKALDLMQAGTIAGSDIKIKGTNDYKDIEGSDAIIITAGLPRKPGMSREDLISINTGIMKTVAANVKKYAPDAFVIVITNPLDVMVYVMLKESGLPHNKVIGMAGVLDSSRFNLFLAEEFKVSVNNVNSMVLGGHGDAMVPLARYSTISGVPIPDLIKMGLSSNENIEKIIDRTRNGGGEIVALLKTGSAYYAPAASAIEMLESYLKDKRQILTCAAHLQGEYGVHDLYVGVPIMIGKEGVLRVIELQLTAEEKALFDKSVEGVKKLIETIK

>Methylobacterium_populi_b

MARSKIALIGAGQIGGTLAHLAGLKELGDVVLFDIVDGVPQGKALDIAESAPVDGFDAKYSGASDYSAIAGADVVIVTAGVPRKPGMSRDDLIGINLKVMEAVGAGIKEHAPNAFVICITNPLDAMVWALQKFSGLPTNKVVGMAGVLDSARFRHFLAEEFGVSVEDVTAFVLGGHGDDMVPLTRYSTVAGVPLTDLVKLGWTTQEKLDAMVERTRKGGGEIVNLLKTGSAFYAPASSAIAMAESYLRDKKRVLPCAAYLAGEYGVDGLYVGVPVVIGENGVERVLEVTFNEDEKAMFEKSVGAVKGLIAACQGINDKLA

>Methylobacterium_nodulans_b

MARKKIALIGAGQIGGTLAHLAGLKELGDVVLFDIADGVPQGKGLDIAESAPVDGFDAKYSGASDYSAIAGADVVIVTAGVPRKPGMSRDDLIGINLKVMEAVGTGIKTHAPNAFVICITNPLDAMVWALQKFSGLDPKKIVGMAGVLDSARFRHFLAEEFSVSVEDVTAFVLGGHGDDMVPLVRYSTVAGIPLPDLVKMGWTTQEKLDAMVERTRKGGGEIVNLLKTGSAFYAPAASAIAMAESYLKDKKRVLPCAAYLTGQYGVDGLFIGVPIVIGENGVERIVEVAFSAEEKAMFDKSVNSVKGLVEACKGINAALA

>Rhizobium_taibaishanense_b

MARNKIALIGSGMIGGTLAHLAGLKELGDIVLFDIADGVPQGKGLDIAQSSPVEGFNAKLTGSSDYAAIEGADVCIVTAGVPRKPGMSRDDLLGINLKVMEQVGSGIKKYAPNAFVICITNPLDAMVWALQKFSGLPANKVVGMAGVLDSSRFRLFLAEEFNVSVQDVTAFVLGGHGDTMVPLARYSTVGGIPLTDLVKMGWVTAERLEQIIQRTRDGGAEIVGLLKTGSAFYAPAASAIEMAESYLKDKKRVLPCAAHLTGQYGVKDMYVGVPTVIGAGGVERVIEIELNKDEEAAFQKSVGAVAGLCEACINIAPALK

>Brucella_abortus_2308_b

MARNKIALIGSGMIGGTLAHLAGLKELGDVVLFDIAEGTPQGKGLDIAESSPVDGFDAKFTGANDYAAIEGADVVIVTAGVPRKPGMSRDDLLGINLKVMEQVGAGIKKYAPEAFVICITNPLDAMVWALQKFSGLPAHKVVGMAGVLDSARFRYFLSEEFNVSVEDVTVFVLGGHGDSMVPLARYSTVAGIPLPDLVKMGWTSQDKLDKIIQRTRDGGAEIVGLLKTGSAFYAPAASAIQMAESYLKDKKRVLPVAAQLSGQYGVKDMYVGVPTVIGANGVERIIEIDLDKDEKAQFDKSVASVAGLCEACIGIAPSLK

>Brucella_ovis_b

MARNKIALIGSGMIGGTLAHLAGLKELGDVVLFDIAEGTPQGKGLDIAESSPVDGFDAKFTGANDYAAIEGADVVIVTAGVPRKPGMSRDDLLGINLKVMEQVGAGIKKYAPEAFVICITNPLDAMVWALQKFSGLPAHKVVGMAGVLDSARFRYFLSEEFNVSVEDVTAFVLGGHGDSMVPLARYSTVAGIPLPDLVKMGWTSQDKLDKIIQRTRDGGAEIVGLLKTGSAFYAPAASAIQMAESYLKDKKRVLPVAAQLSGQYGVKDMYVGVPTVIGANGVERIIEIDLDKDEKAQFDKSVASVAGLCEACIGIAPSLK

>Sphingobium_baderi_LL03_b

MARKKIALIGAGNIGGTLAHLAALKGLGDIVLFDVVEGVPQGKALDLSQCGPVEGFDAKITGTNDYADIKDADVIIVTAGVARKPGMSRDDLLGINLKVMKAVGEGIKNNAPNAFVICITNPLDAMVWALREFSGLPHNKVVGMAGVLDSARFSHFLAEEFQVSVKEVNSFVLGGHGDTMVPVVEYSTVAGIPIPDLIKMGRSTKERIDAIVQRTRSGGGEIVGLLKTGSAFYAPATSGIAMAEAYLFDQKRLLPCAANLTGQYGVDNLYVGVPVIIGKDGVEQVIEIDLDAEAKANLQVSVDAVKELLEACKGIDSSLA

>Novosphingobium_sp._P6W_b

MARKKIALIGAGNIGGTLAHLAAQKELGDIVLFDIAEGIPQGKALDLSQCGPVEGFDASITGTNDYADIAGADVVIVTAGVPRKAGMSRDDLLQINLKVMKAVGEGLKQYAPDAFVICITNPLDAMVWALREFSGLPANKVVGMAGVLDSARFSTFLAWEFGVSIRDVNTFVLGGHGDTMVPVVQYSTVNGIPVPDLIKMGLTTQEKIDAIVQRTRSGGGEIVGLLKTGSAFYAPAASAISMAEAYLNDQKRILPVAAYVDGQYGVDGLYVGVPAMIGADGIEKVIEIELDEEAKGNFQVSVDAVKELLVACKAIDGSLA

>Sphingomonas_panacis_b

MARKKIALIGAGNIGGTLAHLAALKSLGDIVLFDVVEGVPQGKALDLTQCAPVEGFDAKIIGTNDYADIAGADVIIVTAGVARKPGMSRDDLLGINLKVMKSVGEGIKANAPDAFVICITNPLDAMVWALREFSGLPAEKVVGMAGVLDSSRFSAFLAEEFQVSIKDVTSFVLGGHGDTMVPVIEYSTVSGIPVPDLIKMGRSTQEKIDAIIKRTRGGGGEIVALLKTGSAFYAPATSAISMAESYLYDQKRVLPAAVNLTGQYGVDNLYVGVPVVIGAGGVEQIVEIALSDEAKANLDVSVEAVKELLVACKAIDPSLN

>Escherichia_coli_K12_b

MKVAVLGAAGGIGQALALLLKTQLPSGSELSLYDIAPVTPGVAVDLSHIPTAVKIKGFSGEDATPALEGADVVLISAGVARKPGMDRSDLFNVNAGIVKNLVQQVAKTCPKACIGIITNPVNTTVAIAAEVLKKAGVYDKNKLFGVTTLDIIRSNTFVAELKGKQPGEVEVPVIGGHSGVTILPLLSQVPGVSFTEQEVADLTKRIQNAGTEVVEAKAGGGSATLSMGQAAARFGLSLVRALQGEQGVVECAYVEGDGQYARFFSQPLLLGKNGVEERKSIGTLSAFEQNALEGMLDTLKKDIALGEEFVNK

>Actinobacillus_succinogenes_130Z_b

MKVTLLGASGGIGQPLSLLLKLHLPAESDLSLYDVAPVTPGVAKDISHIPTSVEVEGFGGDDPSEALKGADIVLICAGVARKPGMTRADLFNVNAGIIQNLVEKVAQVCPQACVCIITNPVNSIIPIAAEVLKKAGVYDKRKLFGITTLDTIRSEKFIVQAKNIEINRNDISVIGGHSGVTILPLLSQIPHVEFTEQELKDLTHRIQNAGTEVVEAKAGAGSATLSMAYAAMRFVVSMARALNGEVITECAYIEGDGKFARFFAQPVRLGKNGVEEILPLGTLSAFEQQALEAMLPTLQTDIDNGVKFVTGE

>Saccharomyces_cerevisiae_fp

MVKVAILGASGGVGQPLSLLLKLSPYVSELALYDIRAAEGIGKDLSHINTNSSCVGYDKDSIENTLSNAQVVLIPAGVPRKPGLTRDDLFKMNAGIVKSLVTAVGKFAPNARILVISNPVNSLVPIAVETLKKMGKFKPGNVMGVTNLDLVRAETFLVDYLMLKNPKIGQEQDKTTMHRKVTVIGGHSGETIIPIITDKSLVFQLDKQYEHFIHRVQFGGDEIVKAKQGAGSATLSMAFAGAKFAEEVLRSFHNEKPETESLSAFVYLPGLKNGKKAQQLVGDNSIEYFSLPIVLRNGSVVSIDTSVLEKLSPREEQLVNTAVKELRKNIEKGKSFILDSSKL

>Yarrowia_lipolytica_fc

MFRTRVTGSTLRSFSTSAARQHKVVVLGANGGIGQPLSLLLKLNKNVTDLGLYDLRGAPGVAADVSHIPTNSTVAGYSPDNNGIAEALKGAKLVLIPAGVPRKPGMTRDDLFNTNASIVRDLAKAVGEHAPDAFVGVIANPVNSTVPIVAEVLKSKGKYDPKKLFGVTTLDVIRAERFVSQLEHTNPTKEYFPVVGGHSGVTIVPLVSQSDHPDIAGEARDKLVHRIQFGGDEVVKAKDGAGSATLSMAQAAARFADSLLRGVNGEKDVVEPTFVDSPLFKGEGIDFFSTKVTLGPNGVEEIHPIGKVNEYEEKLIEAAKADLKKNIEKGVNFVKQNP

>Yarrowia_lipolytica_fm

MVKAVVAGAAGGIGQPLSLLLKLSPYVTELALYDVVNSPGVAADLSHISTKAKVTGYLPKDDGLKNALTGANIVVIPAGIPRKPGMTRDDLFKINAGIVRDLVTGVAQYAPDAFVLIISNPVNSTVPIAAEVLKKHNVFNPKKLFGVTTLDVVRAQTFTAAVVGESDPTKLNIPVVGGHSGDTIVPLLSLTKPKVEIPADKLDDLVKRIQFGGDEVVQAKDGLGSATLSMAQAGFRFAEAVLKGAAGEKGIIEPAYIYLDGIDGTSDIKREVGVAFFSVPVEFGPEGAAKAYNILPEANDYEKKLLKVSIDGLYGNIAKGEEFIVNPPPAN
