# Supplementary material for: Enhanced succinic acid production by Mannheimia employing optimal malate dehydrogenase
Source: Nat Commun. 2020 Apr 23;11:1970. doi: 10.1038/s41467-020-15839-z (PMC7181634; doi:10.1038/s41467-020-15839-z)
Supplement: Supplementary file 6 — Supplementary Data 2 [file 41467_2020_15839_MOESM6_ESM.docx]

**Supplementary Data 2. Fed-batch fermentation results of the engineered *M. succiniciproducens* strains.**

| **Strain**^a^ | **Culture medium (carbon sources)^b^** | **Final SA (g·l^-1^)** | **Final lactic, acetic, formic and pyruvic acids (byproducts in total)**  **(g·l^-1^)** | **Byproducts per SA**  **(g·g^-1^)** | **Overall productivity**  **(g·l^-1^·h^-1^)** | **Yield^d^**  **(mol·mol^-1^)** | **Specific productivity**  **(g·gDCW^-1^·h^-1^)** | **Source** |
| --- | --- | --- | --- | --- | --- | --- | --- | --- |
| **Fermentation**  **trial #** |  |  |  |  |  |  |  |  |
| **PALK (pMS3)** | CDM (GLC) | 74.56 | 0, 1.37, 0, 6.66 (8.03) | 0.11 | 3.03 | 1.11 | 0.62 | [17] |
| **1** |  |  |  |  |  |  |  |  |
| **PALK (pMS3-msmdh)** | CDM (GLC) | 79.07 | 0, 0.84, 0, 6.34 (7.18) | 0.09 | 3.26 | 1.23 | 0.67 | Supplementary Fig. 10a |
| **1** |  |  |  |  |  |  |  |  |
| **2** | CDM (GLC) | 79.54 | 0, 0.59, 0, 6.35 (6.94) | 0.09 | 3.28 | 1.27 | 0.7 | Supplementary Fig. 10a |
| **PALK (pMS3-cgmdh)** | CDM (GLC) | 87.23 | 0, 2.36, 0, 6.12 (8.49) | 0.1 | 3.6 | 1.29 | 1.02 | Supplementary Fig. 10b |
| **1** |  |  |  |  |  |  |  |  |
| **2** | CDM (GLC) | 87.84 | 0, 2.17, 0, 4.03 (6.2) | 0.07 | 3.63 | 1.3 | 1.02 | Supplementary Fig. 10b |
| **PALK (pMS3-cgmdh^Q20G^)** | CDM (GLC) | 79.39 | 0, 1.72, 0, 5.78 (7.5) | 0.09 | 3.27 | 1.0 | 1.0 | Supplementary Fig. 10c |
| **1** |  |  |  |  |  |  |  |  |
| **2** | CDM (GLC) | 78.53 | 0, 1.84, 0, 4.75 (6.59) | 0.08 | 3.23 | 1.01 | 0.99 | Supplementary Fig. 10c |
| **PALK (pMS3-msmdh^G11Q^)** | CDM (GLC) | 84.19 | 0, 0.68, 0, 4.42 (5.1) | 0.06 | 3.48 | 1.08 | 1.08 | Supplementary Fig. 10d |
| **1** |  |  |  |  |  |  |  |  |
| **2** | CDM (GLC) | 83.52 | 0, 0.11, 0, 6.66 (6.77) | 0.08 | 3.44 | 1.11 | 1.06 | Supplementary Fig. 10d |
| **PALKcgmdh** | CDM (GLC) | 89.6 | 0, 1.3, 0, 6.41 (7.71) | 0.09 | 3.71 | 1.28 | 1.17 | Supplementary Fig. 10e |
| **1** |  |  |  |  |  |  |  |  |
| **2** | CDM (GLC) | 87.05 | 0, 1.64, 0, 6.28 (7.92) | 0.09 | 3.6 | 1.3 | 1.04 | Supplementary Fig. 10e |
| **PALKmsmdh^G11Q^** | CDM (GLC) | 82.76 | 0, 1.5, 0, 3.56 (5.06) | 0.06 | 3.42 | 1.15 | 0.76 | Supplementary Fig. 10f |
| **1** |  |  |  |  |  |  |  |  |
| **2** | CDM (GLC) | 85.67 | 0, 1.39, 0, 4.81 (6.2) | 0.07 | 3.54 | 1.16 | 0.85 | Supplementary Fig. 10f |
| **PALKpfrdmsmdh** | CDM (GLC) | 74.39 | 0, 0.65, 0, 6.08 (6.73) | 0.09 | 3.01 | 1.15 | 0.67 | Supplementary Fig. 11a |
| **1** |  |  |  |  |  |  |  |  |
| **2** | CDM (GLC) | 77.36 | 0, 0.35, 0, 6.43 (6.78) | 0.09 | 3.13 | 1.18 | 0.66 | Supplementary Fig. 11b |
| **PALK** | CDM  (GLC + GOL) | 90.68 | 0, 2.29, 0, 3.98 (6.27) | 0.07 | 3.49 | 1.15 | 1.0 | [15] |
| **1** |  |  |  |  |  |  |  |  |
| **PALK (pMS3-cgmdh)** | CDM  (GLC + GOL) | 101.96 | 0, 1.09, 0, 4.96 (6.05) | 0.06 | 4.06 | 1.34 | 1.25 | Supplementary Fig. 14a |
| **1** |  |  |  |  |  |  |  |  |
| **2** | CDM  (GLC + GOL) | 100.2 | 0, 0.81, 0, 3.67 (3.48) | 0.04 | 3.97 | 1.33 | 1.24 | Supplementary Fig. 14b |
| **PALKcgmdh** | CDM  (GLC + GOL) | 101.18 | 0, 1.46, 0, 4.71 (6.17) | 0.06 | 4.18 | 1.37 | 1.3 | Fig. 3a |
| **1** |  |  |  |  |  |  |  |  |
| **2** | CDM  (GLC + GOL) | 99.69 | 0, 1.88, 0, 5.75 (7.63) | 0.08 | 4.12 | 1.32 | 1.3 | Supplementary Fig. 15a |
| **PALKmsmdh^G11Q^** | CDM  (GLC + GOL) | 92.5 | 0, 1.98, 0, 4.92 (6.90) | 0.07 | 3.82 | 1.28 | 1.03 | Fig. 3b |
| **1** |  |  |  |  |  |  |  |  |
| **2** | CDM  (GLC + GOL) | 91.49 | 0, 1.98, 0, 5.3 (7.28) | 0.08 | 3.78 | 1.29 | 1.05 | Supplementary Fig. 15b |
| **PALK** | CDM  (GLC + GOL)  OD_600, 0_ = 21.1 (9.52 gDCW·l^-1^) | 98.28 | 0, 1.42, 0, 3.17 (4.59) | 0.05 | 8.93; 18.4^c^ | 0.93 | 0.94 | Fig. 3c |
| **1** |  |  |  |  |  |  |  |  |
| **2** | CDM  (GLC + GOL)  OD_600, 0_ = 23.6 (10.64 gDCW·l^-1^) | 103.98 | 0, 1.18, 0, 3.86 (5.04) | 0.05 | 9.08; 18.1^c^ | 1.0 | 0.85 | Supplementary Fig. 15c |
| **PALKcgmdh** | CDM  (GLC + GOL)  OD_600, 0_ = 19.3 (8.7 gDCW·l^-1^) | 134.25 | 0, 2.14, 0, 6.12 (8.26) | 0.06 | 10.33; 21.3^c^ | 1.25 | 1.19 | Fig. 3d |
| **1** |  |  |  |  |  |  |  |  |
| **2** | CDM  (GLC + GOL)  OD_600, 0_ = 20.9 (9.43 gDCW·l^-1^) | 133.28 | 0, 2.24, 0, 4.23 (6.47) | 0.05 | 10.25; 21.33^c^ | 1.28 | 1.09 | Supplementary Fig. 15d |
| **PALK (pMS3-atmdhc1)** | CDM (GLC) | 82.39 | 0, 1.14, 0, 6.51 (7.65) | 0.09 | 3.4 | 1.28 | 0.97 | Supplementary Fig. 16a |
| **1** |  |  |  |  |  |  |  |  |
| **2** | CDM (GLC) | 83.65 | 0, 0.82, 0, 6.8 (7.62) | 0.09 | 3.45 | 1.27 | 1.01 | Supplementary Fig. 16a |
| **PALK (pMS3-atmdhm1)** | CDM (GLC) | 76.83 | 0, 1.09, 0, 5.45 (6.54) | 0.09 | 3.17 | 1.27 | 0.89 | Supplementary Fig. 16b |
| **1** |  |  |  |  |  |  |  |  |
| **2** | CDM (GLC) | 73.64 | 0, 0.74, 0, 5.58 (6.32) | 0.09 | 3.04 | 1.16 | 0.86 | Supplementary Fig. 16b |

^a^ Grey-shaded fermentation results are presented in the main figures while those of other fermentations are provided in indicated Supplementary figures.

^b^ Abbreviations: GLC, glucose; GOL, glycerol; CDM, chemically defined medium; OD_600, 0_, initial OD_600._

^c^ Maximum productivity (g·l^-1^·h^-1^).

^d^ As two kinds of carbon source were used in this study, the yield was calculated based on glucose equivalent (mol SA per mol glucose equivalent) for comparison.
