# Supplementary material for: Enhanced succinic acid production by Mannheimia employing optimal malate dehydrogenase
Source: Nat Commun. 2020 Apr 23;11:1970. doi: 10.1038/s41467-020-15839-z (PMC7181634; doi:10.1038/s41467-020-15839-z)
Supplement: Supplementary file 2 — Peer Review File [file 41467_2020_15839_MOESM2_ESM.pdf]

Editorial Note: This manuscript has been previously reviewed at another journal that is not operating a transparent peer review scheme. This document only contains reviewer comments and rebuttal letters for versions considered at *Nature Communications*. Mentions of prior referee reports have been redacted.

Reviewers' comments:

Reviewer #1 (Remarks to the Author):

General comments:
The purpose of this work was to evaluate whether the step catalyzed by malate dehydrogenase could be rate limiting in the succinate production and productivity by Succinic producer Mannheimia. succinogenes. The authors reported unexpected data showing that the MDH of this bacteria (msMDH) is much less active that the one from Corynebacterium glutamicum, and more importantly, this msMDH is 10 to 20 times more sensitive to substrate inhibition (ie OAA used in the reduction sense as it is the way to produce succinate) than the that of Corynbacterium. It should be noticed that the Ki of msMDH is probably within the physiological concentration of OAA (although the authors were unable to measure it!), whereas cgMDH is likely not inhibited by physiological concentration of OAA. Thus, difference between these two MDHs is at two levels: higher activity at physiological pH (inset in figure 1) and strong inhibition of msMDH by OAA.
The authors crystallized both MDH and identified Gly11 in the catalytic sites of msMDH as the equivalent of Q20 in cgMDH for the binding of the pyrophosphate moieties of the NADH. They showed that replacing G by Q at position 11 resulted in increased catalytic activity, and more importantly, the inihibition by OAA is dramatically released, yet still the msMDHG11Q showed 3 to 4 times higher sensitivity to OAA at physiological pH (around 7.0) than that of the cgMDH. The authors mentioned in the discussion that the inhibition was uncompetitive!
The validation that higher activity MDH is critical in succinic acid production was shown by the finding that the replacement of msMDH by cgMDH in the engineered strain (PALK) increased this production by 10 -15%. As the engineered strain with the variant msMDHG11Q reached similar levels as with the cgMDH indicates. Is this later data could explained that the increased succinic production due to release of OAA inhibition or to higher activity of MDH per se. The authors should discuss on this point!
Overall, this work has been carried out and contains a lot of data, but way it is written does not sufficiently show the key information and importance of this work. It is written in a somewhat confusing manner. As for instance, there is not a single word stating in the abstract that the msMDH is highly sensitive to OAA. Also, there are important data in the discussion, such as that OAA inhibition is uncompetitive! Finally, this is a pretty good work that should find its place in dedicated journals like Metabolic Engineering rather than Nature Comm.

Specific comments
In the Abstract, it should be stated that OAA strongly inhibits MDH in uncompetitive manner with a Ki that is at least 10 times higher for msMdh than cgMDH
In the introduction, it should be stated that there are two types of MDH, cytosolic and mitochondrial and that msMDH belongs to mitochondrial family whereas cgMDH to cytosolic one. Then this should bring additional arguments to search for a role of MDH in succinate production. This would simplify part of the results in 7 and 8, since this distinction is known
Page 6, line 136 -139. It should be stressed that there is a great difference between the two enzymes with respect to OAA inhibition!
Page 7, line 151: Please provide a reference at the end of this sentence.
Page 7, line 158 to the end, page 8, line 166 -169. A great part should go into the Introduction, and the data should be put in Supp. data
Page 10 to 13. I do not see the reason to expend so much on all these fermentation data. There should be only reported key fermentation showing that both higher activity and release of OAZA inhibition have an impact in succinate production. Figures should be restricted to these key data, whereas a summary of all the conditions is clearly provided in Table 2
Page 17, line 391 to 395. This is clearly data that should go to 'results' section and in addition, data on NADH inhibition should be presented in Results section and these data would support the previous finding that OAA interacts at this region and the inhibition is probably due to prevention of NADH binding. In addition, this would support the finding that SA production is enhanced by co-cultivation with glucose and glycerol.

Figure 2 should be splitted in two figures, one with the model structure, the other with the kinetic data
Figure 3: This figure is not necessary as the main results are given in Fig.4 and data in figure 3 are nicely summarized in Table 2

Reviewer #2 (Remarks to the Author):

The manuscript of Ahn et al describes the engeneering of Mannheimia strain to produce high amounts of succinic acid. It consists basically of three parts. First, it describes a kinetic characterization of several MDHs from different sources. Second, it describes the structures of MDHs from M. succiniproducens and C. glutamicum and kinetic analysis of mutants produced based on the structural analysis. Finally, it analyses succinate production with homo- and heterologously (over)produced MDH genes. The part with structural analysis is actually redundant for the main message of the manuscript, i.e. engineering of succinate producer, but it is interesting and makes the manuscript more compelling. Although the revision has significantly improved the manuscript, I have identified several points that must be addressed before the publication of the study.

l. 78-84: how these enzymes were identified? Any experimental support for the identification, references?

L. 84-86: was thought to be: may be better “was predicted to be”?

Ref. 18 is not correct (page numbers)

Suppl Note: reductive TCA cycle: reductive branch of the TCA cycle.

Suppl Note: “fumarase, which irreversibly oxidizes fumarate into malate”: this is not correct. First, fumarase reaction is fully reversible. Second, there is no oxidation or reduction in fumarase reaction.

Suppl Note: S. cerevisiae does not possess the reductive TCA cycle.

Suppl. Note: “ScMDH1, which is [..] a member of TCA cycle”: I am not sure that it’s appropriate to call enzyme as a member of a cycle.

l. 216-218, 266-268: I do not believe that oxaloacetate intracellular concentration can reach the range of hundreds of microM. Mitochondrial concentration is actually in a low micromolar range. The papers cited in the manuscript for the intracellular oxaloacetate concentration (Refs. 35, 36) are not correct, as they do not report any value for oxaloacetate. Furthermore, high oxaloacetate concentration is highly unlikely, taking into account the kinetic properties (Km to OAA) of typical NAD-dependent malate dehydrogenase and high activity in most of bacteria. Please note that the specific activity of the enzyme in M. succiniproducrens is actually not reported. as the values given in Suppl Fig. 9 are inappropriate (μM min-1). There is no indication here how much protein from the extracts was used for the assay. Altogether, I also doubt that the issue with substrate inhibition by OAA has any physiological sense. To discuss it, the authors must determine intracellular oxaloacetate concentration.

Table 1: please add the information for which substrate(s) catalytic constants were measured (NADH and oxaloacetate for forward and malate/NAD for backward reaction?). This is actually not clear throughout the manuscript.

Please summarize the kinetic properties of enzymes in an understandable table (i.e., Km and Vmax values of different MDH to different (named!) substrates)

Figs. 1b, 2f: relative activity: which specific activity corresponds to 100%?

Figs. 1c, d, 2g, h: Rate: mkM min-1: please provide the activity in proper units (e.g., in mkmoles/min/mg protein). The values do not have much sense without the information on the enzyme amount.

Malate dehydrogrenase assays (l. 683-701): NADH extinction coefficient?

Discussion at l. 289-294 is not very convincing. The conclusion that "MsMDH does not contribute much to SA production in the presence of CgMDH or MsMDH" cannot be made on the presented results, as both the kinetic properties of the enzymes and their specific activities in the cells are not really clear. The substitution of MDH leads to the increase in SA production of 20%. That is not that high value to claim that the native enzyme does not contribute much.

The term "overexpression" is used in the manuscript inappropriately. It can be used only in case of homologous enzyme production, as only a gene that is present in the WT can be OVERexpressed.

Reviewer #3 (Remarks to the Author):

The revised manuscript entitled "Enhanced succinic acid production by Mannheimia employing optimal malate dehydrogenase" by Ahn et al has included numerous improvements in the text as well as additional experimentation and crystallographic refinement. My impression is that the findings are solid and that the authors have made a significant improvement in succinic acid production. On the flip side, the methodology used is not novel. Thus, while this is a better fit for Nat Comms
the question is whether the impact of this increase in succinate production in the absence of a methodological advance is sufficient, or whether this is suited for a more specialized journal.

I have two outstanding comments that could improve the manuscript.

First, the manuscript would benefit from a scientifically trained native-English speaking proof-reader to do a very deep and thorough proof-reading. The goals would be to ensure that sentence structures are consistent throughout, to help to clarify concepts, and to make the overall presentation more succinct. My impression is that the overall results are solid but that the presentation are somewhat obscuring a reasonably exciting advance. The logic currently feels a little bit winding throughout, and a significant look at the text would be helpful. In addition, the response to the comments about adding rationale for some of the experiments could be further improved. I had the sense that the authors included more information on what they tried, but didn't really expand on why they tried things. For example, they included a list of the 8 MDH enzymes and listed the biological roles of these in the supplement, but this does not explicitly explain to a reader why they tried certain things - it instead makes a reader infer why the authors tried certain things.

The second comment is following on the previous suggestion that the FRD could be a target of OAA inhibition. I recognize that this particular FRD has not been yet biochemically characterized, but all of the characterized FRD (and SDH) to date are very strongly inhibited by OAA, with Ki values in the nM range. It is reasonable to expect that this extends to other FRDs. For aerobic respiration, this feedback inhibition is a known regulator of the citric acid cycle and it may have a similar regulatory role during anaerobic respiration. The data presented here do not preclude the possibility that increasing MDH activity increases succinate production by multiple mechanisms. Or to put it another way, an increase in MDH activity can increase succinate production by increasing overall substrate availability for the latter enzymes in the process, removing local inhibition of MDH, and removing feedback inhibition of other enzymes. I agree that a detailed enzymological proof is beyond the scope of this paper, but mentioning the many possibilities is appropriate in the discussion.

Reviewers' comments:

Reviewer #1 (Remarks to the Author):

1. General comments:

The purpose of this work was to evaluate whether the step catalyzed by malate dehydrogenase could be rate limiting in the succinate production and productivity by Succinic producer *Mannheimia. succinogenes*. The authors reported unexpected data showing that the MDH of this bacteria (msMDH) is much less active that the one from *Corynebacterium glutamicum*, and more importantly, this msMDH is 10 to 20 times more sensitive to substrate inhibition (ie OAA used in the reduction sense as it is the way to produce succinate) than the that of *Corynbacterium*. It should be noticed that the Ki of msMDH is probably within the physiological concentration of OAA (although the authors were unable to measure it!), whereas cgMDH is likely not inhibited by physiological concentration of OAA. Thus, difference between these two MDHs is at two levels: higher activity at physiological pH (inset in figure 1) and strong inhibition of msMDH by OAA. The authors crystallized both MDH and identified Gly11 in the catalytic sites of msMDH as the equivalent of Q20 in cgMDH for the binding of the pyrophosphate moieties of the NADH. They showed that replacing G by Q at position 11 resulted in increased catalytic activity, and more importantly, the inhibition by OAA is dramatically released, yet still the msMDHG11Q showed 3 to 4 times higher sensitivity to OAA at physiological pH (around 7.0) than that of the cgMDH. The authors mentioned in the discussion that the inhibition was uncompetitive! The validation that higher activity MDH is critical in succinic acid production was shown by the finding that the replacement of msMDH by cgMDH in the engineered strain (PALK) increased this production by 10 -15%. As the engineered strain with the variant msMDHG11Q reached similar levels as with the cgMDH indicates. Is this later data could explained that the increased succinic production due to release of OAA inhibition or to higher activity of MDH per se. The authors should discuss on this point! Overall, this work has been carried out and contains a lot of data, but way it is written does not sufficiently show the key information and importance of this work. It is written in a somewhat confusing manner. As for instance, there is not a single word stating in the abstract that the msMDH is highly sensitive to OAA. Also, there are important data in the discussion, such as that OAA

inhibition is uncompetitive! Finally, this is a pretty good work that should find its place in dedicated journals like Metabolic Engineering rather than Nature Comm.

Response: Thank you very much for providing us with important comments, which were essential for improving our manuscript.

Even though we previously had difficulties in measuring the intracellular OAA concentrations in *Mannheimia succiniciproducen* PALK strain, we were finally able to experimentally determine the OAA concentrations. The results are now included in the revised manuscript (Supplementary Fig. 5). The intracellular OAA concentration was found to be high enough to inhibit *Ms*MDH, while the same OAA concentration inhibited *Cg*MDH to a lesser degree. Thus, utilization of *Cg*MDH showing higher activity and lower substrate inhibition at physiological conditions (pH 6.5-7.0 and OAA concentration at 26.11±4.47 µM) of *M. succiniciproducens* PALK strain made the difference for enhanced SA production compared to using *Ms*MDH.

Following the reviewer's comment, the manuscript was revised to enhance readability and to emphasize the importance of our work for the readers of *Nature Communications*. We agree that our finding on the existence of uncompetitive inhibition in *Ms*MDH is important and thus, moved its description to Results and discussion section (p. 10 line 202). Moreover, a description on strong substrate inhibition toward OAA by *Ms*MDH was added in the Abstract following the reviewer's comment (p. 3 line 33).

p. 3 line 33

Corynebacterium glutamicum MDH (*Cg*MDH) showed the highest specific activity and least substrate inhibition, while *M. succiniciproducens* MDH (*Ms*MDH) showed low specific activity at physiological pH and strong uncompetitive inhibition toward oxaloacetate (*ki* of 67.4 and 588.9 µM for *Ms*MDH and *Cg*MDH, respectively).

p. 10 line 202

A study on kinetic analysis of the MDH from *Phycomyces blakesleeanus*, which is known to have glycine residue at its 11th position, revealed that substrate inhibition prevents NADH binding[34]. This finding is similar to our result that the change in NADH binding motif (replacement of Gly11 *Ms*MDH with Gln) affected substrate inhibition. However, kinetic studies on *Ms*MDH and the *Ms*MDH^{G11Q} variant toward NADH revealed similar results with

those of mitochondrial MDH from *Cryptococcus neoformans*, which exhibited uncompetitive substrate inhibition (Supplementary Fig. 9)[35].

Specific comments

2. In the Abstract, it should be stated that OAA strongly inhibits MDH in uncompetitive manner with a Ki that is at least 10 times higher for msMdh than cgMDH

Response: A sentence in the Abstract section was modified as described above (p. 3 line 33).

3. In the introduction, it should be stated that there are two types of MDH, cytosolic and mitochondrial and that msMDH belongs to mitochondrial family whereas cgMDH to cytosolic one. Then this should bring additional arguments to search for a role of MDH in succinate production. This would simplify part of the results in 7 and 8, since this distinction is known

Response: Thank you for the great suggestion. Description on the existence of two types of MDH (cytosolic and mitochondrial), the categorization of *Ms*MDH (mitochondrial) and *Cg*MDH (cytosolic), and an additional argument to search for a role of MDH in SA production is now added in p. 5 line 91 of the Introduction section.

p. 5 line 91

Phylogenetic analysis of MDH from previous study classified this enzyme into two categories: cytosolic and mitochondrial MDHs, as they were found to be localized at different cellular compartments and showed distinctive characteristics[23]. In general, the cytosolic MDHs preferentially participate in malate-aspartate shuttle for NADH transfer while the mitochondrial MDHs participate in oxidative TCA cycle[24,25]. Thus, the cytosolic MDH seems to be more appropriate for OAA reduction compared with the mitochondrial MDH[26]. However, the MDHs from the major microorganisms employed for SA production, such as *A. succinogenes*, *E. coli*, *M. succiniciproducens*, *S. cerevisiae*, and *Y. lipolytica*, are clustered together with the mitochondrial MDH family, while the *C. glutamicum* MDH belongs to the cytosolic MDH family (Supplementary Fig. 2 and 3). These intriguing results led us to comparatively examine the structural and catalytic characteristics of MDHs.

4. Page 6, line 136 -139. It should be stressed that there is a great difference between the two enzymes with respect to OAA inhibition!

Response: A sentence is added in p. 8 line 162 to emphasize the difference between *Ms*MDH and *Cg*MDH with respect to OAA inhibition.

p. 8 line 162

Taken together, *Ms*MDH showing low *kcat* and *ki* values at pH 5.0~7.0 is not likely the best MDH for the production of SA by *M. succinicproducens* PALK strain having a physiological pH of 6.5~7.0 and an intracellular OAA concentration of 26.11±4.47 μM. Instead, *Cg*MDH showing high *kcat* and *ki* values at pH 5.0~7.0 is a better MDH for enhanced SA production in *M. succiniciproducens*.

5. Page 7, line 151: Please provide a reference at the end of this sentence.

Response: The sentence in p. 7 line 151 "The active sites of *Ms*MDH and *Cg*MDH are located between two distinct N- and C-terminal regions, and their NAD^+/NADH cofactor binding sites are mainly located at the N-terminal region (Fig. 2a-b)." was removed during the revision. Thus, no reference is needed.

6. Page 7, line 158 to the end, page 8, line 166 -169. A great part should go into the Introduction, and the data should be put in Supp. Data

Response: A great part of description from p. 7 line 158 ~ p. 8 line 169 was moved to the Introduction section (p. 5 line 91) and the corresponding data are shown in Supplementary Fig. 2 and 3.

p. 5 line 91

Phylogenetic analysis of MDH from previous study classified this enzyme into two categories: cytosolic and mitochondrial MDHs, as they were found to be localized at different cellular compartments and showed distinctive characteristics[23]. In general, the cytosolic MDHs preferentially participate in malate-aspartate shuttle for NADH transfer while the mitochondrial MDHs participate in oxidative TCA cycle[24,25]. Thus, the cytosolic MDH seems to be more appropriate for OAA reduction compared with the mitochondrial MDH[26].

However, the MDHs from the major microorganisms employed for SA production, such as *A. succinogenes, E. coli, M. succiniciproducens, S. cerevisiae*, and *Y. lipolytica,* are clustered together with the mitochondrial MDH family, while the *C. glutamicum* MDH belongs to the cytosolic MDH family (Supplementary Fig. 2 and 3). These intriguing results led us to comparatively examine the structural and catalytic characteristics of MDHs.

7. Page 10 to 13. I do not see the reason to expend so much on all these fermentation data. There should be only reported key fermentation showing that both higher activity and release of OAA inhibition have an impact in succinate production. Figures should be restricted to these key data, whereas a summary of all the conditions is clearly provided in Table 2

Response: As the results of *in vitro* and *in vivo* studies do not always coincide each other due to the difficulty of mimicking the complex nature of cellular system, it is very important (in strain development) to demonstrate step by step that the findings from *in vitro* study can be successfully applied to the actual strain for enhancing SA production. Hence, the reason for having the "SA production by employing *Cg*MDH and *Ms*MDH^{G11Q} variant" section in the Results and discussion was to describe the logic and process we took to successfully develop the final engineered strain. Nonetheless, according to your suggestion, descriptions on the fermentation results of PALKcgmdh (Supplementary Fig. 10e and Table 2) and PALKmsmdh^{G11Q} (Supplementary Fig. 10f and Table 2) in CDM containing glucose were moved to Supplementary Information. Also, a sentence in p. 13 line 282 was modified. Furthermore, the fermentation results presented previously in Figure 3 are moved to Supplementary Fig. 10 (also summarized in Table 2).

p. 13 line 282

The SA production performance indices obtained with fed-batch fermentation of the PALKcgmdh (Supplementary Fig. 10e and Table 2) and PALKmsmdh^{G11Q} (Supplementary Fig. 10f and Table 2) strains in CDM containing glucose were comparable to those achieved with the PALK (pMS3-cgmdh) (Supplementary Fig. 10b) and PALK (pMS3-msmdh^{G11Q}) (Supplementary Fig. 10d) strains, respectively, which express the *mdh* genes from the plasmids.

8. Page 17, line 391 to 395. This is clearly data that should go to 'results' section and in addition, data on NADH inhibition should be presented in Results section and these data would support the previous finding that OAA interacts at this region and the inhibition is probably due to prevention of NADH binding. In addition, this would support the finding that SA production is enhanced by co-cultivation with glucose and glycerol.

Response: Thank you. We moved it to Results (p. 10 line 202) following the reviewer's suggestion. In addition, our finding on enhanced SA production due to the increased flux of OAA to malate conversion through the elevated supply of NADH (effect of glucose and glycerol co-utilization) is now described in p. 14 line 311.

p. 10 line 202

A study on kinetic analysis of the MDH from *Phycomyces blakesleeanus*, which is known to have glycine residue at its 11th position, revealed that substrate inhibition prevents NADH binding[34]. This finding is similar to our result in that the change in NADH binding motif (replacement of Gly11 *Ms*MDH with Gln) affected substrate inhibition. However, kinetic studies on *Ms*MDH and the *Ms*MDH^{G11Q} variant toward NADH revealed similar results with those of mitochondrial MDH from *Cryptococcus neoformans,* which exhibited uncompetitive substrate inhibition (Supplementary Fig. 9)[35].

p. 14 line 311

In silico simulation (Supplementary Fig. 13) and kinetic studies on *Ms*MDH and the *Ms*MDH^{G11Q} variant (Supplementary Fig. 9) suggested that the OAA to malate conversion flux increased through the elevated supply of NADH.

9. Figure 2 should be splitted in two figures, one with the model structure, the other with the kinetic data

Response: The maximum likelihood phylogenetic tree of MDH-like proteins and the overall structures of *Ms*MDH and *Cg*MDH from the previous version of Fig. 2 are moved to Supplementary Fig. 2 and 6 following the reviewer's comment.

10. Figure 3: This figure is not necessary as the main results are given in Fig.4 and data in

figure 3 are nicely summarized in Table 2

Response: Fermentation results that were presented as Fig. 3 in the previous version are moved to Supplementary Fig. 10 (also summarized in Table 2).

Reviewer #2 (Remarks to the Author):

11. The manuscript of Ahn et al describes the engineering of *Mannheimia* strain to produce high amounts of succinic acid. It consists basically of three parts. First, it describes a kinetic characterization of several MDHs from different sources. Second, it describes the structures of MDHs from *M. succiniproducens* and *C. glutamicum* and kinetic analysis of mutants produced based on the structural analysis. Finally, it analyses succinate production with homo- and heterologously (over)produced MDH genes. The part with structural analysis is actually redundant for the main message of the manuscript, i.e. engineering of succinate producer, but it is interesting and makes the manuscript more compelling. Although the revision has significantly improved the manuscript, I have identified several points that must be addressed before the publication of the study.

Response: Thank you for your helpful comments and suggestions to improve our manuscript. The section on structural analysis was modified to remove redundancy and to emphasize the importance and meaning of our findings. Several important points identified by the reviewer was addressed below in the corresponding comments.

12. l. 78-84: how these enzymes were identified? Any experimental support for the identification, references?

Response: The enzymes described in line 78-84 were identified by *in silico* simulation based on genome-scale metabolic model (Supplementary Fig. 1) and was previously indicated in that sentence.

13. L. 84-86: was thought to be: may be better “was predicted to be”?

Response: Thank you. Reworded as suggested.

14. Ref. 18 is not correct (page numbers)

Response: Thank you. Corrected.

16. Suppl Note: reductive TCA cycle: reductive branch of the TCA cycle.

Response: Thank you. Corrected.

17. Suppl Note: “fumarase, which irreversibly oxidizes fumarate into malate”: this is not correct. First, fumarase reaction is fully reversible. Second, there is no oxidation or reduction in fumarase reaction.

Response: Thank you for your thorough reading of our manuscript and supplementary material. Also, thank you for identifying our mistake. Our apologies. Fumarase catalyzes the reversible hydration and dehydration of fumarate to malate. The information on fumarase is now correctly described in Supplementary Note 1.

Supplementary Note 1.

The wild type *S. cerevisiae* does not naturally produce SA as an end product, especially under anaerobic condition, due to the possession of a fumarase, which catalyzes the reversible hydration and dehydration between fumarate to malate, and fumarate reductase, which is only synthesized under anaerobic condition.

18. Suppl Note: *S. cerevisiae* does not possess the reductive TCA cycle.

Response: *S. cerevisiae* can operate reductive TCA cycle as has been reported by several research groups. Below are a few example papers proving the existence of reductive TCA cycle in *S. cerevisiae.*

[1] Yan, D., Wang, C., Zhou, J., Liu, Y., Yang, M., & Xing, J. Construction and fermentation control of reductive TCA pathway for malic acid production in *Saccharomyces cerevisiae*. *Chin. J. Biotechnol*. **29**, 1484-1493 (2013).

[2] Zelle, R.M. et al. Malic acid production by *Saccharomyces cerevisiae*: engineering of pyruvate carboxylation, oxaloacetate reduction, and malate export. *Appl. Environ. Microbiol*. **74**, 2766-2777 (2008).

[3] Xu, G., Chen, X., Liu, L., & Jiang, L. Fumaric acid production in *Saccharomyces cerevisiae* by simultaneous use of oxidative and reductive routes. *Bioresour. Technol*. **148**, 91-96 (2013).

19. Suppl. Note: "ScMDH1, which is [..] a member of TCA cycle": I am not sure that it's appropriate to call enzyme as a member of a cycle.

Response: We modified a sentence in Supplementary Note 1 to avoid confusion.

Supplementary Note 1

Three isozymes of *Sc*MDH are present in *S. cerevisiae*: *Sc*MDH1, a mitochondrial enzyme; *Sc*MDH2, a cytosolic enzyme known to possess higher OAA reduction activity than malate oxidation activity; *Sc*MDH3, a glyoxysomal enzyme involved with the gluconeogenesis pathway and reoxidation of NADH produced from fatty acid β-oxidation[13].

20. l. 216-218, 266-268: I do not believe that oxaloacetate intracellular concentration can reach the range of hundreds of microM. Mitochondrial concentration is actually in a low micromolar range. The papers cited in the manuscript for the intracellular oxaloacetate concentration (Refs. 35, 36) are not correct, as they do not report any value for oxaloacetate. Furthermore, high oxaloacetate concentration is highly unlikely, taking into account the kinetic properties (Km to OAA) of typical NAD-dependent malate dehydrogenase and high activity in most of bacteria. Please note that the specific activity of the enzyme in *M. succiniproducrens* is actually not reported as the values given in Suppl Fig. 9 are inappropriate (µM min-1). There is no indication here how much protein from the extracts was used for the assay. Altogether, I also doubt that the issue with substrate inhibition by OAA has any physiological sense. To discuss it, the authors must determine intracellular oxaloacetate concentration.

Response: Thank you very much for this important comment. The sentences in 216-218 and

266-268 describing our estimation of OAA concentration being in the range of hundreds of μM were deleted. In addition, the references [35 and 36] cited in the manuscript for intracellular concentration were removed as well.

It took us long time with much effort to establish a reliable method to measure the intracellular OAA concentrations, but we finally were able to measure the intracellular OAA concentration in *M. succiniciproducens*. The data/results and experimental protocol are now presented in Supplementary Fig. 5 and Online methods section, respectively. Moreover, the intracellular OAA concentrations in *M. succiniciproducens* are now provided in p. 8 line 155, and p. 8 line 162.

Regarding the reviewer's comment on cell extract experiment, we apologize for our mistake. Obviously, we measured the enzyme activities (uM·min^{-1}) in the cell extracts (Supplementary Fig. 12) rather than the specific activities. The total amounts of proteins in the PALK and PALKcgmdh cell lysates were measured using the Bradford assay and they are now included in the revised manuscript. The Supplementary Fig. 12 legend and the sentences in p. 30 line 683-687 are revised.

p. 8 line 155

The intracellular OAA concentration in *M. succiniciproducens* PALK strain was 26.11±4.47 μM confirming that substrate inhibition of *Ms*MDH occurred in the cell (Fig. 1h, Supplementary Fig. 5, and Table 1).

p. 8 line 162

Taken together, *Ms*MDH showing low *kcat* and *ki* values at pH 5.0~7.0 is not likely the best MDH for the production of SA by *M. succinicproducens* PALK strain having a physiological pH of 6.5~7.0 and an intracellular OAA concentration of 26.11±4.47 μM.

Supplementary Fig. 12 legend

Supplementary Figure 12. Enzyme activities in the cell extracts of *M. succiniciproducens* PALK and PALKcgmdh strains. Enzyme activity of cell extract was determined using a reaction mixture containing 200 μM NADH and 100 μM OAA. The concentrations of the total proteins in the reaction mixtures containing the PALK and PALKcgmdh cell lysates were 8.8 and 7.6 μg·ml^{-1}, respectively. Standard deviations from experiments performed in triplicate are shown.

p. 30 line 683-687

The enzyme activity in cell extract was measured at pH 7.0 using 0.5 ml of reaction mixture containing 0.1 M Tris-HCl, 200 μM NADH, 100 μM OAA, and 10 μl of cell extract lysate. The concentrations of the total proteins in the reaction mixtures containing the PALK and PALKcgmdh cell lysates were 8.8 and 7.6 $\mu g \cdot ml^{-1}$, respectively.

21. Table 1: please add the information for which substrate(s) catalytic constants were measured (NADH and oxaloacetate for forward and malate/NAD for backward reaction?). This is actually not clear throughout the manuscript.

Response: Both OAA and NADH were used as substrates to measure kinetic parameters as we focused on the OAA reduction reaction, which is the reaction necessary for SA production. For clarity, the sentences in p. 7 line 121, p. 7 line 130, and Table 1 legend were revised.

p. 7 line 121

The OAA reduction activities of four MDHs at pH 7.0 were measured using the purified enzymes.

p. 7 line 130

The kinetic analyses were performed using OAA and NADH as substrates, to focus on the thermodynamically favored reaction, which is the reaction necessary for SA production.

Table 1 legend

Table 1 Kinetic parameters of *Ms*MDH, *Ms*MDH^{G11Q}, and *Cg*MDH toward OAA.

22. Please summarize the kinetic properties of enzymes in an understandable table (i.e., Km and Vmax values of different MDH to different (named!) substrates)

Response: As described in the response for comment 21 by reviewer #2, the kinetic parameters (*kcat*, *km*, *kcat*/*km*, and *ki*) of various MDHs (*Ms*MDH, *Cg*MDH, and

*Ms*MDH^{G11Q}) for OAA are summarized in Table 1. The sentences in p. 7 line 121, p. 7 line 129, and Table 1 legend are revised for clarity. The kinetic parameters of MDHs for NADH are summarized in Supplementary Fig. 9.

23. Figs. 1b, 2f: relative activity: which specific activity corresponds to 100%?

Response: Fig. 1b and 2f (now changed to Fig. 2c) represent the relative activities of various MDHs with respect to the activity of *Ms*MDH. For clarity, the sentences in p. 10 line 194 and p. 29 line 671 and figure legends for Fig. 1b and 2c are revised.

p. 10 line 194

The relative activities of OAA reduction by six *Ms*MDH and *Cg*MDH variants, except for the *Ms*MDH^{A224S} variant, which formed inclusion bodies in *E. coli*, were analyzed in comparison with the activity of *Ms*MDH at pH 7.0 and compared with those of the wild type enzymes.

p. 29 line 671

The relative activities of MDHs in comparison with the activity of *Ms*MDH were measured using 0.5 ml of reaction mixture containing 0.1 M Tris-HCl at pH 7.0, 200 μM NADH, 100 μM oxaloacetate, and 3 nM of various MDHs.

Fig. 1b legend

(b) The relative activities of four MDHs from various SA producers, including *M. succiniciproducens*, *C. glutamicum*, *E. coli*, and *Y. lipolytica*, in comparison with the activity of *Ms*MDH.

Fig. 2c legend

(c) Site-directed mutagenesis and the relative activities of the *Ms*MDH and *Cg*MDH variants in comparison with the activity of *Ms*MDH

24. Figs. 1c, d, 2g, h: Rate: mkM min-1: please provide the activity in proper units (e.g., in mkmoles/min/mg protein). The values do not have much sense without the information on the enzyme amount.

Response: Thank you. The unit for the specific activities presented in Fig. 1c, 1d, 2g (now

changed to 2d), and 2h (now changed to 2e) is now changed to $\mu mol \cdot min^{-1} \cdot mg^{-1}$ protein.

25. Malate dehydrogenase assays (l. 683-701): NADH extinction coefficient?

Response: The NADH extinction coefficient is now added in the manuscript (p. 29 line 667).

p. 29 line 667

The activity of MDH was measured using a spectrophotometer at 340 nm by analyzing residual concentration of NADH (extinction coefficient of 6.22 $mM^{-1} \cdot cm^{-1}$)[51].

26. Discussion at l. 289-294 is not very convincing. The conclusion that "MsMDH does not contribute much to SA production in the presence of CgMDH or MsMDH" cannot be made on the presented results, as both the kinetic properties of the enzymes and their specific activities in the cells are not really clear. The substitution of MDH leads to the increase in SA production of 20%. That is not that high value to claim that the native enzyme does not contribute much.

Response: Thank you for bringing this up, and we apologize for the confusion. It was not our intention to state that *Ms*MDH has no contribution for SA production. In this study, similar SA production performance indices (titer, yield, and productivity) were observed between the PALK (pMS3-cgmdh) and PALKcgmdh strains and between the PALK (pMS3-msmdh^{G11Q}) and PALKmsmdh^{G11Q} strains. The PALK (pMS3-cgmdh) and PALK (pMS3-msmdh^{G11Q}) strains, which additionally harbor *msmdh* gene in the chromosome, use plasmid-based gene expression, while the PALKcgmdh and PALKmsmdh^{G11Q} strains, which do not harbor *msmdh* gene in the chromosome, express *cgmdh* or *msmdh^{G11Q}* gene through genome integration. Based on these comparisons, we can deduce that similar SA production performance between the PALK (pMS3-cgmdh) and PALKcgmdh strains as well as the PALK (pMS3-msmdh^{G11Q}) and PALKmsmdh^{G11Q} strains were mainly resulted from the activity of *Cg*MDH or *Ms*MDH^{G11Q}, respectively, rather than from the activity of *Ms*MDH. Thus, we want to claim that the enhanced SA production by the PALK (pMS3-cgmdh) and PALK (pMS3-msmdh^{G11Q}) strains were primarily due to the introduction of *Cg*MDH or *Ms*MDH^{G11Q}, which exhibit higher *kcat* and *ki* values than *Ms*MDH. The sentences from p. 13 line 282-292 are now modified for better understanding in the revised manuscript.

p. 13 line 282-292

The SA production performance indices obtained with fed-batch fermentation of the PALKcgmdh (Supplementary Fig. 10e and Table 2) and PALKmsmdh^{G11Q} (Supplementary Fig. 10f and Table 2) strains in CDM containing glucose were comparable to those achieved with the PALK (pMS3-cgmdh) (Supplementary Fig. 10b) and PALK (pMS3-msmdh^{G11Q}) (Supplementary Fig. 10d) strains, respectively, which express the *mdh* genes from the plasmids. It is interesting to observe that chromosomal replacement of the *msmdh* with *cgmdh* or *msmdh*^{G11Q} allowed high SA production similar to those achieved by the PALK (pMS3-cgmdh) and PALK (pMS3-msmdh^{G11Q}) strains, even though these latter strains additionally harbor *msmdh* gene in the chromosome. Thus, enhanced SA production by the PALK (pMS3-cgmdh) and PALK (pMS3-msmdh^{G11Q}) strains seems primarily due to the introduction of *Cg*MDH or *Ms*MDH^{G11Q}, both of which possess higher *kcat* and *ki* values than *Ms*MDH.

27. The term “overexpression” is used in the manuscript inappropriately. It can be used only in case of homologous enzyme production, as only a gene that is present in the WT can be OVERexpressed.

Response: Thank you. Corrected throughout the manuscript as the reviewer suggested.

Reviewer #3 (Remarks to the Author):

28. The revised manuscript entitled “Enhanced succinic acid production by Mannheimia employing optimal malate dehydrogenase” by Ahn et al has included numerous improvements in the text as well as additional experimentation and crystallographic refinement. My impression is that the findings are solid and that the authors have made a significant improvement in succinic acid production. On the flip side, the methodology used is not novel. Thus, while this is a better fit for Nat Comms the question is whether the impact of this increase in succinate production in the absence of a methodological advance is sufficient, or whether this is suited for a more specialized journal. I have two outstanding comments that could improve the manuscript.

Response: Thank you for your time and effort of reviewing our manuscript. We did our best to address the reviewer’s two helpful comments to improve our manuscript. The responses to

the reviewers' comments are shown below.

29. First, the manuscript would benefit from a scientifically trained native-English speaking proof-reader to do a very deep and thorough proof-reading. The goals would be to ensure that sentence structures are consistent throughout, to help to clarify concepts, and to make the overall presentation more succinct. My impression is that the overall results are solid but that the presentation are somewhat obscuring a reasonably exciting advance. The logic currently feels a little bit winding throughout, and a significant look at the text would be helpful. In addition, the response to the comments about adding rationale for some of the experiments could be further improved. I had the sense that the authors included more information on what they tried, but didn't really expand on why they tried things. For example, they included a list of the 8 MDH enzymes and listed the biological roles of these in the supplement, but this does not explicitly explain to a reader why they tried certain things - it instead makes a reader infer why the authors tried certain things.

Response: Again, thank you very much for your kind effort to improve our manuscript. Much appreciated. We would like to admit that English is not as good as native English speakers as all the authors are native Koreans. Also, we already have our manuscript reviewed by native English speaker, but this is what it is (in fact, the corresponding author had to redo many sentences as the English correction was wrong). So, we went over the entire manuscript again to improve the readability. The quality of writing might still be below your standard, but we did our best to improve it; based on the feedback from other metabolic engineers in the lab, the contents of the paper are clear, and can be understood without ambiguity and difficulty. The entire manuscript was revised carefully to have consistency in sentence structure and to present the overall work succinctly with logical flow. Especially, the sections in the manuscript describing the reason for selecting 8 different MDHs for evaluation (p. 6 line 111) and the physiological role of these MDHs (Supplementary Note 1) were revised.

A. succinogenes, *C. glutamicum*, *E. coli*, *M. succiniciproducens*, *S. cerevisiae*, and *Y. lipolytica* are the most studied and/or industrially employed strains for bio-based SA production. Thus, eight different MDHs (*As*MDH, *Cg*MDH, *Ec*MDH, *Ms*MDH, *Sc*MDH1, *Sc*MDH2, *Sc*MDH3, and *Yl*MDH) from these most representative SA producers were

evaluated. This was because there would be a better chance of finding an optimal MDH from these microorganisms, which potentially bears desirable properties for enhanced SA production in *M. succiniciproducens,* rather than from other organisms not capable of efficiently producing SA.

p. 6 line 111

In order to find an optimal MDH for OAA to malate conversion, eight different MDHs from the most studied and/or industrially employed natural and non-natural SA producers including *A. succinogenes* (*As*MDH), *C. glutamicum* (*Cg*MDH), *E. coli* (*Ec*MDH), *M. succiniciproducens* (*Ms*MDH), *S. cerevisiae* (mitochondrial *Sc*MDH1, cytosolic *Sc*MDH2, and glyoxysomal *Sc*MDH3), and *Y. lipolytica* (cytosolic *Yl*MDH)[27], were evaluated. This was because there would be a better chance of finding an optimal MDH from these microorganisms, which potentially bears desirable properties for enhanced SA production in *M. succiniciproducens,* rather than from other organisms not capable of efficiently producing SA. The physiological roles of these selected MDHs are summarized in Supplementary Note 1.

30. The second comment is following on the previous suggestion that the FRD could be a target of OAA inhibition. I recognize that this particular FRD has not been yet biochemically characterized, but all of the characterized FRD (and SDH) to date are very strongly inhibited by OAA, with Ki values in the nM range. It is reasonable to expect that this extends to other FRDs. For aerobic respiration, this feedback inhibition is a known regulator of the citric acid cycle and it may have a similar regulatory role during anaerobic respiration. The data presented here do not preclude the possibility that increasing MDH activity increases succinate production by multiple mechanisms. Or to put it another way, an increase in MDH activity can increase succinate production by increasing overall substrate availability for the latter enzymes in the process, removing local inhibition of MDH, and removing feedback inhibition of other enzymes. I agree that a detailed enzymological proof is beyond the scope of this paper, but mentioning the many possibilities is appropriate in the discussion.

Response: Thank you very much for this important comment. We acknowledge the existence of various possibilities that could have additionally contributed to enhanced SA production upon increasing the MDH activity. As the reviewer pointed out, the FRDs that have been

characterized to date were reported to be inhibited by OAA. In addition, those several previously studied FRDs, including *E. coli* FRD, were found to possess *ki* values in the nM range for OAA. Despite FRD having such nM *ki* value, a metabolically engineered *E. coli* strain was reported to produce a large amount of SA under anaerobic condition[1]. The intracellular OAA concentration in *E. coli* was reported to be 30 μM[2]. This means that the FRD responsible for SA production in *E. coli,* was not too much inhibited by the presence of 30 μM OAA. A couple of other studies have also reported that the intracellular OAA concentrations are in μM range. For example, the intracellular OAA concentrations of *C. glutamicum*[3] and *Saccharopolyspora erythraea*[4] were reported to be 7~88 μM and 75 nmol·gDCW^{-1}, respectively; for comparison, the intracellular OAA concentration in PALKcgmdh strain we measured is equivalent to 120.3±36.23 nmol·gDCW^{-1}. Furthermore, the *ki* values reported previously for other FRDs were all measured *in vitro* using the purified FRDs. Taken together, the actual *in vivo* inhibitory OAA concentration of FRD might be different from the inhibitory OAA concentration determined *in vitro*.

[References]
[1] Vemuri, G., Eiteman, M. & Altman, E. Succinate production in dual-phase *Escherichia coli* fermentations depends on the time of transition from aerobic to anaerobic conditions. *J. Ind. Microbiol. Biotechnol.* **28,** 325-332 (2002).
[2] Peng, L., Arauzo-Bravo, M.J. & Shimizu, K. Metabolic flux analysis for a ppc mutant *Escherichia coli* based on ^{13}C-labelling experiments together with enzyme activity assays and intracellular metabolite measurements. *FEMS Microbiol. Lett.* **235,** 17-23 (2004).
[3] Petersen, S. et al. Metabolic consequences of altered phosphoenolpyruvate carboxykinase activity in *Corynebacterium glutamicum* reveal anaplerotic regulation mechanisms *in vivo*. *Metab. Eng.* **3,** 344-361 (2001).
[4] Hong, M., Mou, H., Liu, X., Huang, M. & Chu, J. ^{13}C-assisted metabolomics analysis reveals the positive correlation between specific erythromycin production rate and intracellular propionyl-CoA pool size in *Saccharopolyspora erythraea*. *Bioproc. Biosyst. Eng.* **40,** 1337-1348 (2017).

Introduction of *Cg*MDH in the *M. succiniciproducens* PALK strain reduced the intracellular OAA concentrations from 26.11±4.47 (PALK) to 12.22±3.68 μM (PALKcgmdh) (Supplementary Fig. 5). Hence, if the actual *in vivo* inhibitory OAA concentration of *M. succiniciproducens* FRD is in the μM range (as observed in *E. coli*, which still efficiently produced SA even though *in vitro* inhibitory OAA concentration was in nM range), enhanced SA production by the PALKcgmdh strain would have also been contributed by reduced FRD inhibition by lower concentration of OAA by employing the *Cg*MDH. Further studies are needed to confirm this.

As identified from *in silico* genome-scale metabolic simulation (Supplementary Fig. 1), PCKA, FUMC, and FRD will be the next potential candidates to be studied for enhancing SA production using similar approaches reported in this study. Such structural and biochemical studies on these key enzymes will allow further improvement of SA production. The reviewer's suggestion to mention all these possibilities was truly valuable and thus we added the following paragraph in the revised manuscript (p. 18 line 401). We added the following paragraph in the revised manuscript.

p. 18 line 401

Although MDH was the focus of this study, it is possible that the introduction of more efficient *Cg*MDH into *M. succiniciproducens* might have affected the activities of other enzymes and corresponding reaction fluxes (see Supplementary Note 2 for detailed discussion). As identified from *in silico* genome-scale metabolic simulation (Supplementary Fig. 1), PCKA, FUMC, and FRD can be the next potential candidates to be studied for further enhancing SA production using similar approaches reported here.

In conclusion, we developed a highly efficient SA producing *M. succiniciproducens* strain by integrating the strategies of systems metabolic engineering with enzyme engineering. The use of the best MDH selected from various MDHs through detailed structural and kinetic studies significantly improved SA production. The strategy described in this paper will be useful for developing high performance strains for the production of industrially competitive bio-based chemicals.

Supplementary Note 2. Discussion on the possible effect of *Cg*MDH expression on the inhibition of FRD by OAA.

Several previously studied FRDs, including *E. coli* FRD, were found to possess *ki* values in the nM range for OAA. Despite FRD having such nM *ki* value, a metabolically engineered *E. coli* strain was reported to produce a large amount of SA under anaerobic condition[15]. The intracellular OAA concentration in *E. coli* was reported to be 30 μM[16]. This means that the FRD responsible in SA production in *E. coli* was not too much inhibited by the presence of 30 μM OAA. A couple of other studies have also reported that the intracellular OAA concentrations are in μM range. For example, the intracellular OAA concentrations of *C. glutamicum*[17] and *Saccharopolyspora erythraea*[18] were reported to be 7~88 μM and 75

nmol·gDCW^{-1}, respectively; for comparison, the intracellular OAA concentration in PALKcgmdh strain we measured is equivalent to 120.3±36.23 nmol·gDCW^{-1}. Furthermore, the *ki* values reported previously for other FRDs were all measured *in vitro* using the purified FRDs. Taken together, the actual *in vivo* inhibitory OAA concentration of FRD might be different from the inhibitory OAA concentration determined *in vitro*.

Introduction of *Cg*MDH in the *M. succiniciproducens* PALK strain reduced the intracellular OAA concentrations from 26.11±4.47 (PALK) to 12.22±3.68 μM (PALKcgmdh) (Supplementary Fig. 5). Hence, if the actual *in vivo* inhibitory OAA concentration of *M. succiniciproducens* FRD is in the μM range (as observed in *E. coli*, which still efficiently produced SA even though *in vitro* inhibitory OAA concentration was in nM range), enhanced SA production by the PALKcgmdh strain would have also been contributed by less inhibition of FRD by lower concentration of OAA. Further studies are needed to confirm this.

Reviewers' comments:

Reviewer #1 (Remarks to the Author):

The revised manuscript is now much more improved, more focused
The authors succeeded in measuring intracellular OAA using a very appropriate methode.Levels they found are in the range of the Ki of the MsMDH! This should be stated as well
there is some comments about other OAA-target such as Fumarase (Frd) or succinate oxidoreducatse (sdh), but the authors forgot to mention that these are membrane embedded enzyme and OAA might be very weakly accesible to them, otherwise at such concentration, they should be completely inhibited (Ki <0.01 μM according to literature)

Reviewer #2 (Remarks to the Author):

The authors have satisfactorily responded to all my comments, and I support the publication of the manuscript.

Nevertheless, I still have several very minor comments that are listed below.

l. 311-312: "using glycerol as a co-carbon source generates twice as much reducing equivalents per mole compared with glucose": I do not understand that. How glycerol (C3) can have twice as much reducing equivalents than glucose?

l. 318-321: what are glucose equivalents? How the yield (in mol) can be compared for the utilization of one versus two substrates?

Supplementary Figure 2. Title: "Detailed phylogenetic tree analyses": "Phylogenetic tree of MDH".

Fig 1a, Suppl Fig 1: please consider to insert ADP in PEP carboxykinase reaction (i.e., ADP+CO2)#

Suppl Note 1:
"which catalyzes the reversible hydration and dehydration between fumarate to malate": "which catalyzes the reversible hydration of fumarate to malate". Please also delete comma after S. cerevisiae. Please insert the reference for the sentence starting with "Thus, an E. coli strain deleted with the gene.."

Response to the Reviewer's comment:
"S. cerevisiae can operate reductive TCA cycle as has been reported by several research groups. Below are a few example papers proving the existence of reductive TCA cycle in S. cerevisiae.
[1] Yan, D., Wang, C., Zhou, J., Liu, Y., Yang, M., & Xing, J. Construction and fermentation control of reductive TCA pathway for malic acid production in Saccharomyces cerevisiae. Chin. J. Biotechnol. 29, 1484-1493 (2013).
[2] Zelle, R.M. et al. Malic acid production by Saccharomyces cerevisiae: engineering of pyruvate carboxylation, oxaloacetate reduction, and malate export. Appl. Environ. Microbiol. 74, 2766-2777 (2008).
[3] Xu, G., Chen, X., Liu, L., & Jiang, L. Fumaric acid production in Saccharomyces cerevisiae by simultaneous use of oxidative and reductive routes. Bioresour. Technol. 148, 91-96 (2013)."

S. cerevisiae cannot operate the reductive TCA cycle. For the rTCA cycle, please check the reviews on autotrophic CO2 fixation (e.g., Fuchs, 2011, Annu Rev Microbiol 65:631). I cannot read Ref [1], but I can hardly imagine functioning of ferredoxin-dependent caroxylation in yeasts. I believe, that here the reductive branch of the TCA cycle is meant (sometimes called "horseshoe TCA cycle")

Reviewer #3 (Remarks to the Author):

Revision addresses my comments

Reviewers' comments:

Reviewer #1 (Remarks to the Author):

Comment 1: The revised manuscript is now much more improved, more focused. The authors succeeded in measuring intracellular OAA using a very appropriate method. Levels they found are in the range of the Ki of the MsMDH! This should be stated as well.

Response:

Thank you.

Comment 2: There is some comments about other OAA-target such as Fumarase (Frd) or succinate oxidoreducatse (sdh), but the authors forgot to mention that these are membrane embedded enzyme and OAA might be very weakly accessible to them, otherwise at such concentration, they should be completely inhibited (Ki <0.01 µM according to literature).

Response:

Thank you very much. Also, we like your statement of "weakly accessible". A description on FRD was added in Supplementary Note 2 following the reviewer's comment.

Supplementary Note 2

It should also be noted that FRD is a membrane-bound protein[19], and thus OAA might be very weakly accessible to FRD.

Reviewer #2 (Remarks to the Author):

Comment 3: The authors have satisfactorily responded to all my comments, and I support the publication of the manuscript. Nevertheless, I still have several very minor comments that are listed below.

Response: Thank you for supporting the publication of our manuscript. The minor comments raised by the reviewer are addressed as shown below.

Comment 4: l. 311-312: "using glycerol as a co-carbon source generates twice as much reducing equivalents per mole compared with glucose": I do not understand that. How glycerol (C3) can have twice as much reducing equivalents than glucose?

Response: Sorry for the incomplete information. We should have written it as "per six carbon equivalent". The conversion of glycerol (C3) to phosphoenolpyruvate (C3) generates twice as much reducing equivalents per six-carbon equivalent mole compared with glucose (C6). The metabolic pathways (enzymes) responsible for NADH generation using glucose (glyceraldehyde 3-phosphate dehydrogenase) or glycerol (glyceraldehyde 3-phosphate dehydrogenase and glycerol 3-phosphate dehydrogenase) are shown in Supplementary Fig. 13. Two moles of NADH are generated during conversion of glucose to PEP. Also, two moles of NADH are generated during the conversion of glycerol to PEP. Since glycerol is a three carbon compound and glucose is a six carbon compound, the use of glycerol generates twice more NADH for the same six-carbon mole. A description on why utilizing glycerol generates more reducing equivalents per mole compared with glucose is added in p. 14 line 307.

p. 14 line 307

Based on our previous studies, glucose and glycerol were used as dual carbon sources to enhance SA production by taking full advantage of a highly efficient *Cg*MDH or *Ms*MDH^{G11Q} through providing more reducing equivalents[9,39]; conversion of glycerol (C3) to PEP (C3) generates twice as much reducing equivalents per six-carbon equivalent mole compared with glucose (C6)[40]. Metabolic fluxes of the PALK strain generated from glucose or glucose plus glycerol were compared using *in silico* simulation to verify enhanced NADH generation (glycerol 3-phosphate dehydrogenase and glyceraldehyde 3-phosphate dehydrogenase reactions).

40. Yazdani, S.S. & Gonzalez, R. Anaerobic fermentation of glycerol: a path to economic viability for the biofuels industry. *Curr. Opin. Biotech.* **18**, 213-219 (2007).

Comment 5: l. 318-321: what are glucose equivalents? How the yield (in mol) can be compared for the utilization of one versus two substrates?

Response: Due to the use of dual carbon sources (glucose and glycerol) it was necessary to convert the amount of glycerol consumed (mol) to glucose equivalent to calculate the total

amount of carbon sources consumed (mol glucose equivalent) for clear comparison with the case of utilizing glucose only. As the number of carbons in glucose and glycerol differ by a factor of 2, the amount of glycerol consumed (mol) was adjusted by the same factor (so that it is equivalent to glucose) and added with the amount of glucose consumed (mol) to calculate the total amount of carbon sources consumed (mol glucose equivalent) during the course of fed-batch fermentation. A sentence was added in p. 34 line 800 for clarity.

p. 34 line 800

As the number of carbons in glucose and glycerol differ by a factor of 2, the amount of glycerol consumed (mol) was converted to 'mol glucose equivalent' to calculate the total amount of carbon sources consumed during the course of fed-batch fermentation.

Comment 6: Supplementary Figure 2. Title: "Detailed phylogenetic tree analyses": "Phylogenetic tree of MDH".

Response: It is now changed to "Phylogenetic tree of MDH"

Comment 7: Fig 1a, Suppl Fig 1: please consider to insert ADP in PEP carboxykinase reaction (i.e., ADP+CO2)

Response: Fig. 1a and Supplementary Fig. 1 are now modified following the reviewer's comment.

Comment 8: Suppl Note 1:

"which catalyzes the reversible hydration and dehydration between fumarate to malate": "which catalyzes the reversible hydration of fumarate to malate". Please also delete comma after S. cerevisiae. Please insert the reference for the sentence starting with "Thus, an E. coli strain deleted with the gene.."

Response: Supplementary Note 1 is now modified.

Comment 9: Response to the Reviewer's comment:

"S. cerevisiae can operate reductive TCA cycle as has been reported by several research groups. Below are a few example papers proving the existence of reductive TCA cycle in S. cerevisiae.

[1] Yan, D., Wang, C., Zhou, J., Liu, Y., Yang, M., & Xing, J. Construction and fermentation control of reductive TCA pathway for malic acid production in Saccharomyces cerevisiae. Chin. J. Biotechnol. 29, 1484-1493 (2013).

[2] Zelle, R.M. et al. Malic acid production by Saccharomyces cerevisiae: engineering of pyruvate carboxylation, oxaloacetate reduction, and malate export. Appl. Environ. Microbiol. 74, 2766-2777 (2008).

[3] Xu, G., Chen, X., Liu, L., & Jiang, L. Fumaric acid production in Saccharomyces cerevisiae by simultaneous use of oxidative and reductive routes. Bioresour. Technol. 148, 91-96 (2013)."

S. cerevisiae cannot operate the reductive TCA cycle. For the rTCA cycle, please check the reviews on autotrophic CO2 fixation (e.g., Fuchs, 2011, Annu Rev Microbiol 65:631). I cannot read Ref [1], but I can hardly imagine functioning of ferredoxin-dependent caroxylation in yeasts. I believe, that here the reductive branch of the TCA cycle is meant (sometimes called "horseshoe TCA cycle")

Response: Thank you for your comment. We originally used the “reductive branch” term. One of the reviewers wanted us to change it to reductive TCA cycle. Thank you for agreeing with what we originally used. The term “reductive TCA cycle”, which was used in Supplementary Note 1, is changed back to reductive branch of the TCA cycle for clarity.

Supplementary Note 1

However, bio-based SA production using either reductive branch or oxidative TCA cycle has been achieved by metabolic engineering of *S. cerevisiae*[10].

Reviewer #3 (Remarks to the Author):

Comment 10: Revision addresses my comments.

Response: Thank you for reviewing our manuscript.

REVIEWERS' COMMENTS:

Reviewer #2 (Remarks to the Author):

The revision has addressed all my comments.
